# Synthesis and molecular modelling studies of pyrimidinones and pyrrolo[3,4-*d*]-pyrimidinodiones as new antiplasmodial compounds

**DOI:** 10.1590/0074-02760170452

**Published:** 2018-06-18

**Authors:** Kamilla Rodrigues Rogerio, Leonardo J M Carvalho, Luiza Helena Pinto Domingues, Bruno Junior Neves, José Teófilo Moreira, Rosane Nora Castro, Cesare Bianco, Claudio Tadeu Daniel-Ribeiro, Carolina Horta Andrade, Cedric Stephan Graebin

**Affiliations:** 1 Universidade Federal Rural do Rio de Janeiro, Departamento de Química, Laboratório de Diversidade Molecular e Química Medicinal, Seropédica, RJ, Brasil; 2 Fundação Oswaldo Cruz-Fiocruz, Instituto Oswaldo Cruz, Laboratório de Pesquisas em Malária, Rio de Janeiro, RJ, Brasil; 3 Universidade Federal de Goiás, Faculdade de Farmácia, Laboratório de Planejamento de Fármacos e Modelagem Molecular, Goiânia, GO, Brasil; 4 Universidade Federal Rural do Rio de Janeiro, Instituto de Ciências Exatas, Departamento de Química, Seropédica, RJ, Brasil; 5 Centro Universitário de Anápolis, UniEvangélica, Laboratório de Quimioinformática, Anápolis, GO, Brasil; 6 Universidade de Campinas, Instituto de Biologia, Departamento de Genética, Evolução e Bioagentes, Laboratório de Doenças Tropicais Professor Dr Luiz Jacintho da Silva, Campinas, SP, Brasil

**Keywords:** Malaria, Plasmodium falciparum, multicomponent reactions, Biginelli reaction, pyrimidinones, molecular modelling

## Abstract

**BACKGROUND:**

Malaria is responsible for 429,000 deaths per year worldwide, and more than 200 million cases were reported in 2015. Increasing parasite resistance has imposed restrictions to the currently available antimalarial drugs. Thus, the search for new, effective and safe antimalarial drugs is crucial. Heterocyclic compounds, such as dihydropyrimidinones (DHPM), synthesised via the Biginelli multicomponent reaction, as well as bicyclic compounds synthesised from DHPMs, have emerged as potential antimalarial candidates in the last few years.

**METHODS:**

Thirty compounds were synthesised employing the Biginelli multicomponent reaction and subsequent one-pot substitution/cyclisation protocol; the compounds were then evaluated *in vitro* against chloroquine-resistant *Plasmodium falciparum* parasites (W2 strain). Drug cytotoxicity in baseline kidney African Green Monkey cells (BGM) was also evaluated. The most active *in vitro* compounds were evaluated against *P. berghei* parasites in mice. Additionally, we performed an *in silico* target fishing approach with the most active compounds, aiming to shed some light into the mechanism at a molecular level.

**RESULTS:**

The synthetic route chosen was effective, leading to products with high purity and yields ranging from 10-84%. Three out of the 30 compounds tested were identified as active against the parasite and presented low toxicity. The *in silico* study suggested that among all the molecular targets identified by our target fishing approach, Protein Kinase 3 (PK5) and Glycogen Synthase Kinase 3β (GSK-3β) are the most likely molecular targets for the synthesised compounds.

**CONCLUSIONS:**

We were able to easily obtain a collection of heterocyclic compounds with *in vitro* anti-*P. falciparum* activity that can be used as scaffolds for the design and development of new antiplasmodial drugs.

Currently, malaria is considered the most important parasitic disease in the world. It is endemic in 91 countries, and 216 million new cases and 445,000 deaths were reported in 2016, being ninety percent of these deaths concentrated in Africa (WHO 2016).

There is a limited arsenal of drugs currently available to treat malaria. *Plasmodium falciparum*, the species responsible for the vast majority of severe cases and mortality, has become increasingly resistant to most of the available drugs. In Brazil, for instance, the widespread resistance of *P. falciparum* to chloroquine became prevalent in the 1980s, and this drug had to be replaced by other first-line drugs, such as the combination of quinine and doxycycline, and more recently by artemisinin combination therapies (ACTs) (de Andrade et al. 1992, Alecrim et al. 2006). Chloroquine is still in use to treat *P. vivax* infections, but reports of increased resistance are leading to arguments in favour of a chloroquine replacement by ACTs, as well (de Santana Filho et al. 2007, Pedro et al. 2012). ACTs are indeed becoming the first-choice treatment for malaria in many parts of the world. It combines the features of the potent and fast acting artemisinin derivatives, such as artesunate or artemether, with the extended action of drugs such as lumefantrine, amodiaquine or mefloquine, resulting in a higher efficacy of the treatment and limiting the risk for the emergence of resistant parasites (Angus et al. 2002, WHO 2010, Rosenthal 2013). However, great concern has arisen in the past few years, with reports of the emergence of artemisinin-resistant parasites, first in Southeast Asia and then in other parts of the world (Mita and Tanabe 2012, Rosenthal 2013). The spread of artemisinin resistance has the potential of reversing the gains in the fight against malaria observed in the past years, with serious consequences to affected populations. For this reason, the continuous search for novel antimalarial drugs is still at high priority to contain this threat. Thus, the research and development of new antimalarial drug candidates against *P. falciparum* and *P. vivax* infections are essential to achieve the goal of global malaria elimination.

Molecular targets validated in the literature as useful for malaria chemotherapy include Calcium-dependent targets (Calcium dependent ATPase PfATP6), Hexose transporter (pfHT) *Plasmodium* farnesyl transferase (PfPFT), glutathione reductase, thioredoxin reductase, glutathione-*S-*transferase, plasmepsins, falcipains, facilysins, protein kinases and glycolytic enzymes (Leite et al. 2013, Aguiar et al. 2017).

Dihydropyrimidinones (DHPMs), a class of heterocyclic compounds, can be easily synthesised from the Biginelli multicomponent reaction, which is a one-pot cyclocondensation reaction of ethyl acetoacetate (a *β*-dicarbonyl compound), an aldehyde and urea or thiourea, with a catalytic amount of acid (Kappe 2000). Diverse biological activities are reported in the literature for DHPMs, such as antiviral (Kim et al. 2012), calcium channel blocker (Kappe 2000), anticancer (Raju et al. 2011), and antifungal (Singh et al. 2008) activities, among others. Some DHPMs had their antiplasmodial activity against *P. falciparum* reported in the past few years (Chiang et al. 2009). Monastrol, a DHPM obtained from the Biginelli reaction, has become a prominent compound among synthetic nitrogenous heterocycles and paved the way for the creation of a prodigious collection of analogue compounds to find effective and selective drugs against various diseases (Pérez et al. 2002). The short number of steps and highly modular nature of the Biginelli reaction can be exploited to quickly generate a library of heterocyclic compounds, with a great molecular diversity, aiming to contribute to the discovery of new, biologically active antiparasitic compounds. Therefore, this study aims to synthesise and evaluate several DHPMs and closely related heterocycles towards new compounds with potential antiplasmodial activity.

## MATERIALS AND METHODS

**Fig. 1:**
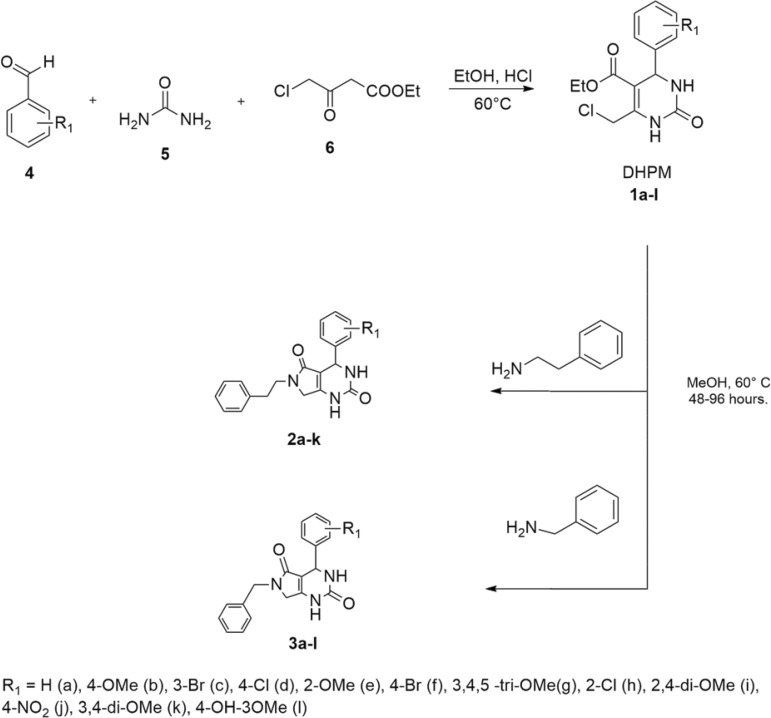
: Biginelli multicomponent synthesis of compound collections 1a-l, 2a-k and 3a-l.

*Chemical syntheses* - The collection of DHPMs **1a-1k** were obtained via the Biginelli reaction (Fig. 1), using aromatic aldehydes **4**, urea **5** and ethyl 4-chloroacetoacetate **6** as building blocks, as well as aqueous HCl as catalysts. The chloromethylene moiety present in DHPMs **1a-1k** was the starting point to the subsequent one-pot substitution/cyclisation protocol employing phenylethylamine or benzylamine, leading to the pyrrolo[3,4-*d*]-pyrimidinodiones **2a-k** and **3a-l** (Pérez et al. 2002). The synthesised compounds had their structures confirmed through the usual analytical techniques [FT-IR, ^1^H-NMR, ^13^C-NMR spectra, and mass spectrometry, as well as the HPLC purity assays, are available in (Supplementary data I).

*Antiplasmodial in vitro assay* - The cultivation of *P. falciparum* W2 strain (originating from Indochina and cultivated in the Malaria Research Laboratory, Oswaldo Cruz Foundation-Fiocruz), which is chloroquine-resistant, was made according to the protocol of Trager and Jensen (1976), using human erythrocytes (A^+^) in an RPMI 1640 medium supplemented with human serum. Parasites were synchronised to obtain the young (ring) parasite forms (Lambros and Vanderberg 1979). Parasitaemia was adjusted to 0.05% and haematocrit to 1.5%. Subsequently, the cultures were placed in microplates with the drugs, previously diluted in dimethyl sulphoxide and medium (DMSO 0.02% v/v), to be tested in serial dilutions (50 - 0.78 µM), as well as positive controls (chloroquine in serial dilutions; 10 - 0.07 µM) and negative (no drug) controls. All the microplates were incubated for 72 h. The microplates were then subjected to three cycles of freezing and thawing; the parasite growth was measured through the quantification of histidine-rich protein II (HRPII) using specific monoclonal antibodies (MPFM ICLLAB-55A®, USA and MPFG55P ICLLAB®, USA) in sandwich ELISA (Noedl et al. 2002). Absorbance was read at 450 nm in an Espectramax 190 spectrophotometer (Molecular Devices, USA), and the determination of the IC_50_ was made using dose-response curves.

*Cytotoxicity evaluation on mammalian cells* - BGM cells (originally acquired from ATCC (American Type Culture Collection), Manassas, VA, USA by the Federal University of Minas Gerais, Brazil, and donated by Dr Antoniana Krettli - Federal University of Minas Gerais) were cultured in 75 cm^2^ sterile ﬂasks containing RPMI 1640 medium supplemented with 10% foetal bovine serum (FBS) at a concentration of 5 x 10^3^ cells per millilitre in a 5% CO_2_ atmosphere at 37ºC. Cells were added with the test compounds in serial dilution. When the cell monolayer was confluent, it was trypsinised, washed with a culture medium distributed in a flat-bottomed 96-well plate (1 × 10^5^ cells/mL) and incubated for 24 h at 37ºC to ensure cell adherence. Then, the drugs were added in serial dilution (1000-15 µM) and incubated for 24 h at 37ºC. Subsequently, the MTT salt, in a concentration of 3 mg/mL, was added after 3 h; then, the supernatant was removed, and the dye present on the bottom of the plate wells was dissolved in DMSO in a volume of 100 µL/well DMSO. The microplates were then read in an Espectramax 190 (Molecular Devices, USA) spectrophotometer, using the 570 nm filter. The determination of MDL_50_ was performed using a dose response curves nonlinear regression function (Denizot and Lang 1986).

*In vivo activity evaluation against P. berghei* - Swiss mice (Institute of Science and Technology in Biomodels - Fiocruz) were inoculated with 10^5^ erythrocytes infected with *P. berghei* ANKA expressing green fluorescent protein (PbA-GFP, a donation from the malaria research and reference reagent resource centre -MR4, Manassas, VA; deposited by CJ Janse and AP Waters; MR4 number: MRA-865) intraperitoneally (day 1 of the experiment). Three hours after inoculation, the animals were randomly divided into five groups of mice. Two groups were used as controls: non-treated and treated with chloroquine; each of the other three groups received either compound **1b**, compound **1f** and compound **1h**, which showed the best efficacy against *P. falciparum in vitro*. All of the test compounds and chloroquine were administered at a dose of 30 mg/kg, orally by gavage for four consecutive days. On days 5 and 8 after infection, flow cytometry was used to detect and count the number of parasitised red blood cells (RBC) that expressed GFP in relation to 10,000 RBCs, thus assessing the evolution of parasitaemia. This PbA-GFP parasite has been shown to be a rapid, simple and sensitive tool for the examination of new antimalarial compounds *in vivo* (Sánchez et al. 2004). The antimalarial activity was determined by the percentage reduction in parasitaemia of treated animals compared to untreated controls (Peters et al. 1975).

*Hemolysis assay* - The haemolysis assay was performed according to the protocol described by Wang et al. (2010). The compounds were dissolved in DMSO (0.5% v/v). Suspensions of erythrocytes (1% haematocrit) were incubated with the compounds at serial dilutions ranging from 15 to 1000 µM, at 37ºC for 30 min with a constant stirring. A 0.05% solution of saponin (Sigma-Aldrich) was used as a positive control of the test as it generated 100% haemolysis, and the suspension of erythrocytes in the medium was used as a negative control (blank). Following incubation, suspensions were centrifuged at 1000 g for 10 min, and the absorbance of the supernatants was measured at 540 nm using an Espectramax 190 spectrophotometer. The haemolysis rate of the samples was calculated as follows: % haemolysis = (sample absorbance - blank absorbance) x 100 / absorbance of the control with saponin.

*Computational assays* - *In silico target fishing study - Structure-based pharmacophore screening* - The 3D structure of the most active compound **1f** (in Mol2 format) was used as the template and submitted to the PharmMapper web server (Liu et al. 2010, Wang et al. 2016). During the procedure, the maximum conformations were set, up to 300, and the number of reserved matched targets was 300. Other parameters were kept as a default.

*Homology modelling* - The amino acid sequence of the predicted targets were retrieved from the UniProt database (Apweiler 2004) and used as target for homology modelling in the SWISS-MODEL server (Bordoli et al. 2009, Biasini et al. 2014). Then, the built models were exported to the GalaxyWEB server (Ko et al. 2012), which refines loop or terminus regions by *ab initio* modelling. Lastly, the structure reliability of the built models were evaluated by using the MolProbity server (Chen et al. 2010). Models with the lowest Clashscore and MolProbity score were selected for further analysis. The Clashscore is the number of serious steric clashes per 1000 atoms. The MolProbity score is a log-weighted combination of the percentage of bad side-chain rotamers, percentage of Ramachandran outliers, and Clashscore, giving one number that reflects the resolution of the X-ray structures at which those values would be expected (Chen et al. 2010).

*Preparation of ligand structures* - The 3D structures of the most active 1,4-dihydropyrimidin-2(1H)-ones (DHPMs), compounds **1b**, **1d**, and **1f**, were imported into the Maestro workspace v.9.3 (Schrödinger, LCC, New York, 2012) and prepared using LigPrep v.2.5 (Schrödinger, LCC, New York, 2012). All possible ionisation and tautomeric states were generated at pH 7.0 ± 1.0 using Epik v.2.3 (Shelley et al. 2007). The lowest potential energy conformers and tautomers were retained as input for docking studies.

*Molecular docking* - The 3D structures of the predicted *P. falciparum* targets were imported into Maestro workspace and prepared using Protein Preparation Wizard workflow as follows: hydrogen atoms were added according to Epik v.2.3 (Shelley et al. 2007) calculation for pKa values (at pH 7.0 ± 1.0) and minimised using the OPLS-2005 force field (Banks et al. 2005). Then, grid boxes, with lengths ranging between 17-24 Å radius around the active sites, were created using the receptor grid generation module of the Glide v.5.8 (Friesner et al. 2004). The details of each grid are shown in Supplementary data II. Then, docking simulations were carried out in Glide v.5.8, using the “XP” resolution. Finally, the binding orientations of the ligands in the active site of the 3D model structures for the *P. falciparum* targets and their respective homologues in humans were analysed, and the most energetically favourable conformations were selected by GlideScore function (Eldridge et al. 1997).

*Similarity analysis* - A substructure searching of the DHPM scaffold was performed in the ChEMBL database (EMBL-EBI - Available from: https://www.ebi.ac.uk/chembl/) using the Sketch tool. The chemical similarity search yielded 470 compounds that were active (IC_50_, Ki, or Kd ≤ 10 µM) against at least one of 42 biological targets.

*Ethics approval and consent to participate* - The animal experiments performed in this study were approved by the Oswaldo Cruz Institute’ Commission for the Ethical Animal Use (CEUA/IOC), license number L-037/2015.

## RESULTS

**Fig. 2:**
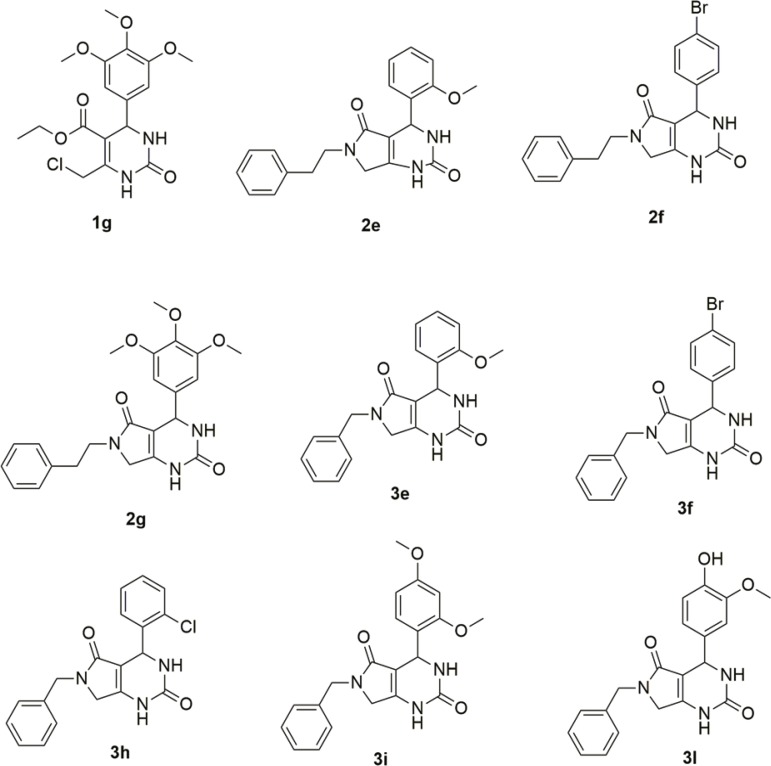
: novel compounds synthesised in this work.

*Chemistry* - The planned compounds were successfully synthesised via the Biginelli multicomponent reaction (Fig. 1) and the subsequent one-pot nucleophilic substitution/cyclisation, leading to pyrrolo[3,4-*d*]-pyrimidinodiones. The purity of the products was verified by high-performance liquid chromatography (Supplementary data I). For most (compounds **1a-k**, **2f-h**, **3b**, **3d**, **3f-i**, **3k** and **3l**), 100% purity was reported. Other compounds were in the 83-99% purity range. A total of 30 molecules were synthesised, 9 being novel compounds. The structures of those novel compounds are shown in Fig. 2.

The yields obtained in the syntheses ranged from low (10%) to good (up to 84.4%). Aniline was not employed in the cyclisation step since reports in the literature indicate that the cyclisation does not happen, even if the reaction temperature is raised to 250ºC (Pérez et al. 2002).

*Antiplasmodial activity and cytoxicity of the synthesised compounds* - The *in vitro* antiplasmodial activity of the compounds was evaluated using the chloroquine-resistant *P. falciparum* W2 strain (Trager and Jensen 1976). Of the 30 compounds assayed *in vitro*, 13 showed no activity against the W2 strain, even at the highest concentration employed (50 µM) in the assay (Table I). Seventeen compounds presented antiplasmodial activity, nine of them at concentrations below 10 µM. All of the compounds (**1a-d**, **1f**, **1h-l**) with IC_50_ values below 10 µM (6.38, 2.98, 6.17, 1.96, 1.76, 3.12, 6 and 3, respectively) belong to the monocyclic DHPM class.

**TABLE I:** Antiplasmodial and cytotoxicity assays

Compound	R_1_	n	IC_50_ (µM)^a^	MDL_50_ (µM)^a^	SI^b^
1a	H	-	6.38 ± 0.8	24.6 ± 0.03	3.9
1b	4-OMe	-	2.98 ± 0.2	57 ± 0.65	19
1c	3-Br	-	6.17 ± 0.02	21.7 ± 1.24	3.5
1d	4-Cl	-	1.96 ± 0.21	2.5 ± 0.93	1.3
1e	2-OMe	-	12.2 ± 0.8	23.8 ± 0.6	1.95
1f	4-Br	-	1.76 ± 0.27	22 ± 0.56	12.5
1g	3,4,5-tri-OMe	-	24.1 ± 1.2	21.7 ± 1.15	0.9
1h	2-Cl	-	3.12 ± 0.06	77 ± 0.87	24.6
1i	2,4-di-OMe	-	2.98 ± 0.18	9.7 ± 0.46	3.3
1j	3,4-di-OMe	-	6 ± 0.8	26.2 ± 1.1	4.4
1k	3-OMe, 4-OH	-	3 ± 0.25	24.5 ± 0.09	8.2
2a	H	2	> 50	> 100	-
2b	4-OMe	2	26.4 ± 0.26	24.6 ± 1.23	0.93
2c	3-Br	2	> 50	6.9 ± 0.97	0.1
2d	4-Cl	2	-	-	-
2e	2-OMe	2	22 ± 0.08	56 ± 1.83	2.5
2f	4-Br	2	> 50	18.6 ± 1.21	2.5
2g	3,4,5-tri-OMe	2	> 50	6.2 ± 0.73	< 0.1
2h	2-Cl	2	> 50	> 100	-
2i	2,4-di-OMe	2	-	-	-
2j	3,4-di-OMe	2	25.6 ± 0.09	8.9 ± 0.9	0.4
2k	3-OMe, 4-OH	2	-	-	-
3a	H	1	> 50	> 100	-
3b	4-OMe	1	> 50	> 100	-
3c	3-Br	1	-	-	-
3d	4-Cl	1	45 ± 0.79	86 ±1.27	1.9
3e	2-OMe	1	> 50	> 100	-
3f	4-Br	1	26 ± 1.15	> 100	> 3.8
3g	3,4,5-tri-OMe	1	> 50	> 100	-
3h	2-Cl	1	26.7 ± 0.56	> 100	> 3.8
3i	2,4-di-OMe	1	>50	> 100	-
3j	3,4-di-OMe	1	> 50	> 100	-
3k	3-OMe, 4-OH	1	> 50	> 100	-
Chloroquine	-	-	0.55 ± 0.09	-	-

a: results are expressed as a mean of three experiments; b: selectivity index (SI = MDL_50_ / IC_50_).

Cytotoxicity assays were completed using the BGM mammal cell line (Denizot and Lang 1986) (Table I). In general, compounds with weaker or no activity against *P. falciparum* showed lower toxicity and high MDL_50_ values. Conversely, compounds with higher activity against *P. falciparum* showed lower MDL_50_ values.

To further assess the potential toxicity of the compounds, a haemolysis assay (Wang et al. 2010) was also performed. Only five of the 30 compounds induced some degree of haemolysis at 1000 μM: **1f**, **1d**, **2b**, **2c** and **2f** (0.95%, 3.6%, 21.7%, 12.3% and 22.6% respectively). At 62 μM none of the compounds induced any degree of haemolysis.

The three compounds with the best antiplasmodial activity and selectivity indexes *in vitro* were selected for *in vivo* studies, using Swiss Webster mice infected with *Plasmodium berghei* (four-days) (Peters et al. 1975) (Table II). The three compounds most active in the in vitro assays also caused inhibition of *in vivo P. berghei* parasitaemia in mice, with 33-60% reduction of parasite burden at day eight post-inoculation.

**TABLE II:** *In vivo Plasmodium berghei* parasitaemia reduction after treatment

Compound	Concentration	Reduction (%)	

		Day five	Day eight
1b	30 mg/Kg	0	33.8
1f	30 mg/Kg	9.1	60.9
1h	30 mg/Kg	0	55.2
Chloroquine	30 mg/Kg	97.2	100

*In silico target fishing study* - *In silico* target fishing is an emerging approach that enables the prediction of biological targets of bioactive compounds, usually identified from phenotypical assays. This approach considers the similarity in chemical structure of the active compound with the chemical and target information from increasingly available databases (Jenkins et al. 2006, Cereto-Massagué et al. 2015). The prediction of the ability of small molecules to interact with biological targets is of interest for the rational design of more effective and less toxic drugs. Here, the biological targets of the synthesised DHPMs were predicted by integration of two *in silico* approaches: structure-based pharmacophore target fishing and molecular docking studies. The PharmMapper server predicted the top 300 potential protein targets for the selected DHPM. This tool predicted the best poses for the most active compound of the series, **1f**, against all the pharmacophore models present in the PharmTargetDB, and ranked the best-fitted biological targets with their respective scores (Liu et al. 2010, Wang et al. 2016). From the predicted targets in PharmMapper server, only two proteins were from *P. falciparum* (L-lactate dehydrogenase and protein kinase 5). This outcome probably occurred due to the small number of 3D protein structures of *Plasmodium* in this database. Therefore, the amino acid sequences of the predicted targets (except from *Plasmodium* proteins) were aligned with all *P. falciparum* proteins, assuming that proteins sharing similarity (homology) have enhanced the probability of sharing the same ligands (Klabunde 2007, Rognan 2007, Rose et al. 2015). Based on these alignments, homologues with sequential identity higher than 50% were prioritised for further analysis.

Considering the absence of X-ray structures for most of the P. *falciparum* proteins in the Protein Data Bank (PDB) database (Merritt et al. 2014), homology models were built. The details of the prioritised *P. falciparum* targets and the homology modelling statistical results are presented in Supplementary data II. Statistical analysis of the modelled protein structures showed that most parts of the amino acids are within the favoured Ramachandran regions (97.3-98.6%) and have good rotamers (97.3-98.6%), showing the good quality of the backbone dihedral angles (ψ against φ) and side-chain angles (χ) of the amino acids. In addition, acceptable Clashscores (5.98-7.46) and MolProbity scores (1.26-1.54) were obtained for these structures. Therefore, these characteristics suggest that the homology models can be useful for prospective molecular modelling investigations.

To further characterise the binding modes and the interaction scores of most active compounds with the predicted *P. falciparum* targets, molecular docking studies were performed using Glide software. As we can see in Table III, the GlideScore energies of the docking indicated that the studied DHPMs could act as antimalarial compounds due to their higher binding affinities with protein kinase 5 (PK5) and glycogen synthase kinase-3 beta (GSK-3β). Less pronounced binding energies were observed for other proteins. On the other hand, docking studies indicated that the DHPMs have considerable affinity to human homologues (see Supplementary data II).

**TABLE III:** Docking score results (kcal/mol) of selected 1,4-dihydropyrimidin-2(1H)-ones with *Plasmodium falciparum* targets

Predicted target	1a	1b	1c	1d	1f	1h	1i	1k	1l
Protein kinase 5	-8.76	-8.00	-8.87	-8.36	-8.33	-9.31	-6.25	-8.24	-9.10
Glycogen synthase kinase-3 β	-7.95	-7.92	-7.91	-7.89	-8.00	-6.27	-6.56	-8.06	-8.07
Methionine aminopeptidase	-6.38	-6.67	-6.64	-6.93	-5.64	-5.40	-6.55	-7.32	-6.74
cAMP-dependent protein kinase	-5.91	-5.71	-6.68	-6.20	-5.95	-6.60	-5.38	-5.55	-7.13
Casein kinase II	-6.33	-6.75	-7.06	-6.01	-5.59	-5.82	-5.20	-6.07	-7.94
Glyceraldehyde-3-phosphate dehydrogenase	-5.54	-6.15	-5.95	-5.92	-5.46	-6.26	-6.14	-5.87	-6.92
Heat shock protein 86	-5.38	-5.93	-5.48	-5.96	-5.20	-5.79	-5.48	-4.04	-5.84
Casein kinase I	-5.50	-7.10	-5.61	-4.78	-5.12	-5.86	-4.23	-4.77	-6.78
Actin I	-3.67	-3.71	-3.66	-3.75	-3.68	-3.89	-4.42	-4.12	-4.30
L-lactate dehydrogenase	-5.07	-3.79	-5.10	-4.36	-3.67	-4.55	-3.91	-3.49	-5.26

*: bold values indicate the potential targets according to docking score energies.

## DISCUSSION

In this study, we obtained 30 DHPMs and the related pyrrolo[3,4-*d*]-pyrimidinodiones, using a simple chemical strategy with a small number of synthetic steps.

During the synthesis, it was observed that the nature of the functional groups (R_1_, Scheme 1) in the aromatic aldehydes had no correlation with the yields obtained, leading us to believe that in the case of DHPM synthesis, the ring substituents do not affect the reaction, or the slow step is not the attack to the carbonyl group. The same effect was observed in the synthesis of the bicyclic compounds.

Regarding the *in vitro* assays for the monocyclic compound family (**1a-l**), it seems that there is not a clear relationship between the aromatic ring groups substituent nature (R_1_) and the antiplasmodial activity. Table I shows that both electron-donor and withdrawing groups at positions 2, 3 and/or 4 leads to IC_50_ values ranging from 1-10 µM, with the exception of compounds **1e** and **1g**. Comparing, for example, compounds **1b** and **1d** (with 4-Cl and 4-methoxy groups, respectively), we observed comparable IC_50_ values. Compounds **1h** and **1d**, presenting a chlorine atom at positions 2 and 4, respectively, also present similar activity in this assay. Regarding **1b** and **1e**, with a methoxy group in positions 2 and 4, there is a more pronounced IC_50_ difference (12.2 and 2.98 µM, respectively) that was not observed when comparing **1h** and **1d**. In both cases, the compound with a substituent group at position 2 was less active than the 4-substituted one. A tri-substituted derivative, **1g**, presented the highest IC_50_ value of this series (24.1 µM), comparable to the bicyclic compounds.

Noticeably, the cyclisation strategy employing primary amines (Compound series **2a-l** and **3a-l**) led to greater IC_50_ values when compared to the monocyclic compounds (**1**), indicating that this modification is not suitable for antiplasmodial activity.

**Fig. 3:**
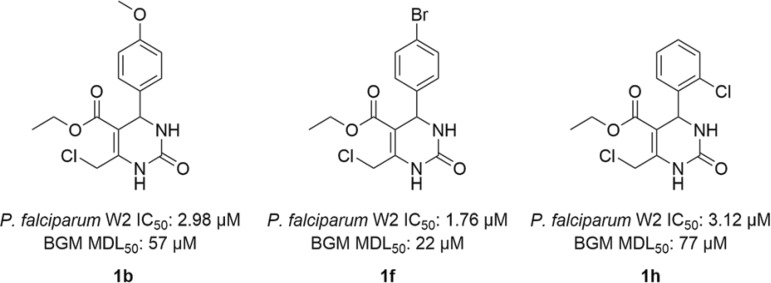
: synthesised compounds with promising results for future updates.

The cytotoxicity assay indicates that the most active compounds are also the most toxic, especially when comparing compound series **1** (monocyclic dihydropyrimidinones) and **2** (*N*-phenylethyl pyrrolo[3,4-*d*]-pyrimidinodiones). These findings indicate that compound modifications that decrease cytotoxicity also decrease antiplasmodial activity, suggesting that the mechanisms of antiplasmodial activity and toxicity are similar. Nevertheless, three compounds showed a combination of good antiplasmodial activity and lower cytotoxicity, resulting in good selectivity indexes (SI): **1b**, **1f** and **1h** (Fig. 3). There was initially a concern regarding the probable cytotoxicity of the monocyclic DHPMs, because of the electrophilic nature of the chloromethylene moiety present in all the compounds, but as Table I shows, it is safe to affirm that this moiety is not responsible for the cytotoxic effects observed in some of the derivatives, since it is easily observable that the modifications in this series are not related to the chloromethylene moiety, present in all DHPMs. Concerning the haemolysis assay, most compounds showed no hemolytic effect even at the high concentration of 1000 µM.

In terms of *in vivo* activity, although the activity (Table II) of the three compounds was not as good as the reference drug (chloroquine), these data indicate that optimisations in the DHPM structure may lead to more *in vivo* effective compounds. Although the *in vivo* activity of the compounds was not as good as the reference drug (chloroquine), these data indicate that optimisations in the DHPM structure may lead to more *in vivo* effective compounds.

**Fig. 4:**
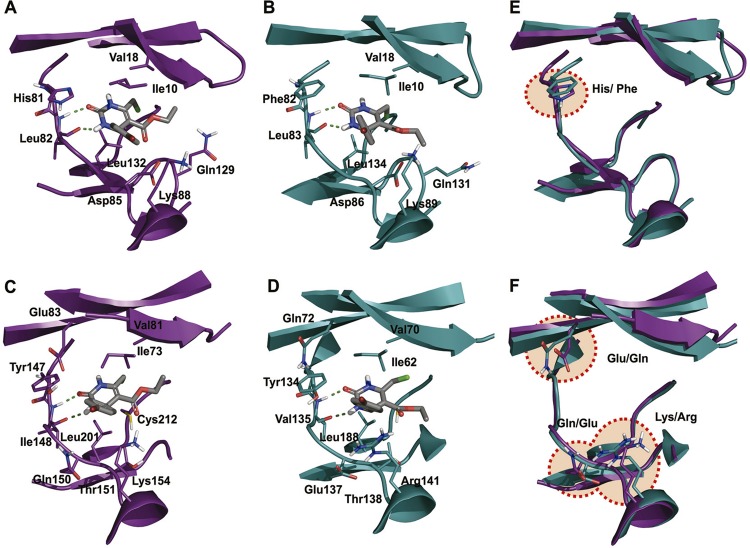
: predicted intermolecular interactions of 1f with ATP binding sites of *Plasmodium falciparum* PK5 (A, backbone in purple), human CDK2 (B, backbone in green), *P. falciparum* GSK-3β (C, backbone in purple) and human GSK-3β (D, backbone in green). Compound 1f is represented in stick models with carbon atoms coloured grey, nitrogen in blue, oxygen in red, hydrogen in white and bromine in dark red. (E) Structural alignment between *P. falciparum* PK5 (purple) and its human homologue CDK2 (green), and (F) between *P. falciparum* GSK-3β (purple) and its human homologue GSK-3β (green), highlighting the ATP binding sites differences.

Understanding the intermolecular interactions with predicted targets gives deeper insight into the inhibition mechanisms and builds a foundation for the rational design of more selective and potent DHPMs. The molecular modelling studies indicated that DHPMs could act as antimalarial agents because of their interaction with *P. falciparum* protein kinases. A common feature of protein kinase inhibitors is their ability to bind to the adenosine triphosphate (ATP) pocket, also called the hinge region. Most of the known small-molecule protein kinase inhibitors that are steady-state ATP competitive inhibitors also make hydrogen bonds with the backbone residues of the connecting hinge (Roskoski 2016). According to the GlideScore energies, investigated DHPMs may have antiplasmodial activity because of their affinity to *P. falciparum* PK5, a member of the family of cyclin-dependent protein kinases with important role in parasite DNA replication (Deshmukh et al. 2015, Deshmukh et al. 2016). The intermolecular interactions of **1f** in ATP binding site of PK5 (Fig. 4A) can be generalised as follows: the urea moiety can form hydrogen bonds (represented as green dashed lines) with the carbonyl/amine backbone of the hinge amino acid Leu82. In addition, the chloromethyl group of **1f** can interact with hydrophobic pocket formed by Val18, Leu132, and Ala142, while the phenyl group of **1f** can interact with the pocket formed by Ile10 and Lys86 of PK5. Similar interactions were also observed for **1f** in the ATP binding site of human homologue CDK2 (Fig. 4B).

The other predicted protein target, GSK-3β, is one of the eukaryotic protein kinases that was identified as essential for *P. falciparum*. Although its biological functions are not yet clarified, GSK-3β was demonstrated to be an essential enzyme for completion of the asexual erythrocytic cycle for the parasite (Droucheau et al. 2004, Masch et al. 2015). As seen in Fig. 4C, the urea moiety of **1f** can form hydrogen bonds (represented as dashed lines) with the carbonyl/amine backbone of the hinge Ile148. In addition, the chloromethyl group can interact with the hydrophobic pocket formed by Val81 and Cys212, while the phenyl group and ethyl acetate interact with the pocket formed by Ile73 and Val81. Compound **1f** also interacts in a similar manner in the ATP binding pocket of human homologue GSK-3β (Fig. 4D), but its aromatic ring is able to carry out an additional π-cation interaction with Arg141. All of these interactions are characteristic of known protein kinase inhibitors (Roskoski 2016).

Considering the similar binding modes and binding affinities, the predicted *P. falciparum* kinase structures were superimposed onto their corresponding human homologues. Both PK5 and GSK-3β are structurally similar with their counterparts (sequential identity of 64% and 55%, respectively). However, our analysis revealed notable electrostatic differences between the binding sites (see Fig. 4E-F). For instance, amino acid residues of the binding site for parasite PK5 (His81) and GSK-3β (Glu83, Gln150, and Lys154) were substituted in human CDK2 (Phe82) and GSK-3β (Gln72, Glu137, and Arg141) proteins, respectively. These structural differences may be useful to design more potent and selective anti-plasmodial lead candidates.

In addition to molecular docking studies, we performed a similarity search in ChEMBL database to explore the activity profile of known DHPMs. From this similarity search, we found that DHPM-based compounds are potent inhibitors of some human kinases, such as Rho-associated protein kinase 1 and rhodopsin kinase (IC_50s_ of 0.014 µM and 0.10 µM, respectively, see Supplementary data III). DHPMs are also reported as inhibitors of two *P. falciparum* proteins, i.e., heat shock protein (HSP) 70 (IC_50_ = 0.72 µM) (Chiang et al. 2009) and M18 aspartyl aminopeptidase (IC_50_ of 1.51 µM) (NCBI 2018). However, our molecular docking studies showed that the studied DHPMs present unsatisfactory GlideScore energies with these proteins (data not shown).

*In conclusion* - This study reports the quick and short-step synthesis of 30 heterocyclic compounds, with three of the compounds (**1b**, **1f** and **1h**) presenting good *in vitro* activity against the chloroquine-resistant *P. falciparum* W2 strain. Our *in silico* studies suggest that the observed antiplasmodial activity could be due to the inhibition of PK5 and GSK-3β, although further *in vitro* enzymatic studies are needed to confirm these results. The *in vivo* activity of those compounds against *P. berghei*-infected mice, unfortunately, was not as good as the standard drug chloroquine. However, in view of the widespread resistance of *P. falciparum* to the classic antimalarial drug classes, such as the aminoquinolines and sulphadoxine-pyrimethamine, and the emerging resistance to the artemisinins, the availability of a new class of chemicals with antiplasmodial activity inhibiting enzymes that are not currently the molecular target of the known standard antimalarial drugs and that can be obtained through a simple synthetic protocol is promising, as future enhancements can lead to improved biological activity of the compounds that will not be subjected to the same mechanisms of resistance acting against the currently available drugs.

*Availability of data and materials* - Synthetic protocols, FT-IR, ^1^H-NMR, ^13^C-NMR, mass spectrometry and HPLC purity assays concerning all the reported compounds are available as a supplementary data in DOCX format (Supplementary data I). Additionally, several tables, concerning the *in silico* studies (PharmMapper predictions, homology modelling details, statistical validation of the homology models, and docking scores of all docking calculations), are available as a supplementary data in XLSX format (Supplementary data II). A summary of the structures for the dihydropyrimidinone compounds found in the ChEMBL database is available as a supplementary data in DOCX format (Supplementary data III).
