# Supplementary material for: Synthesis and molecular modelling studies of pyrimidinones and pyrrolo[3,4-d]-pyrimidinodiones as new antiplasmodial compounds
Source: Mem Inst Oswaldo Cruz. 2018 Jun 18;113(8):e170452. doi: 10.1590/0074-02760170452 (PMC6001580; doi:10.1590/0074-02760170452)
Supplement: Supplementary file 1 [file 0074-0276-mioc-113-8-e170452-suppl01.pdf]

A. General methodology for synthesis of DHPMs 1a-1k:

The reactions were performed in vials with a screw cap so as to hold eight reactions at a time optimizing the time, which would not be possible with use of balloon were added to it the aromatic aldehyde, urea and 4-chloroacetoacetate ethyl in the ratio 1: 1.08: 1, respectively, 2 mL of ethanol and 2 drops of concentrated HCl. Reagents were purchased from commercial sources (Sigma-Aldrich and ACROS Organics) and were used without purification. The reaction was maintained at 60 °C for 48 hours using a IKA HS-7 heating plate with block and temperature control from a temperature sensor connected to this block.

Product isolation was accomplished by filtering the precipitate and washing with cold ethanol. When no precipitate formed spontaneously during cooling, the solution was poured into water-ice and then filtered.

The purification method employed for removal of impurities was added a small portion of ethanol and conducting the boiling compounds for at least 5 minutes, after cooling the solution was filtered to give the purified product.

6-Chloromethyl-2-oxo-4-phenyl-1,2,3,4-tetrahydro-pyrimidine-5-carboxylic acid ethyl ester **1a**

White solid; Melting point: 280 °C; Yield: 29.7%

IR (KBr): 3300 cm^{-1}, 1710 cm^{-1} e 1670 cm^{-1}.

^{1}H-NMR (500 MHz, DMSO-d_6): 1.12 (t, J = 7.2 Hz, 3H), 4.05 (q, J = 7.2 Hz, 2H), 4.6 (d, J=10.7Hz, 1H) e 4.78 (d, J=10.4Hz, 1H), 5.2 (sl, 1H), 7.26-7.36 (m, 5H), 7.87 (s, 1H), 9.87 (s, 1H). ^{13}C-NMR (125MHz, DMSO-d_6): δ, 14, 54.3, 60.4, 102, 126.8, 128, 129, 144, 146.5, 152.5.

Mass spectrometry $C_{14}H_{15}ClN_2O_3$ Na^+: 317.066341, calculated: 317.0669.

6-Chloromethyl-4-(4-methoxy-phenyl)-2-oxo-1,2,3,4-tetrahydro-pyrimidine-5-carboxylic acid ethyl ester **1b**.

Physical State: White solid; Melting point: 280 ° C; Yield: 22%

IR: 3300 cm^{-1}, 1710 cm^{-1} e 1670 cm^{-1}

^{1}H NMR (500MHz, DMSO-d_6):δ, 1.13 (t, J=7.25 Hz, 3H), 3.73 (s, 3H), 4.04 (q, J= 6.9 Hz, 2H), 4.6 (d, J= 10.4 , 1H) e 4.76 (d, J= 10.7Hz, 2H), 5.14 (sl, 1H), 6.89 – 6.91 (2d, J=8.8Hz, 2H), 7.17-7.18 (2d, J=8.5Hz, 2H), 7.8 (s, 1H), 9.47 (s, 1H). ^{13}C NMR (125 MHz, DMSO-d_6): δ, 14.4, 53.7, 55.5, 60.4, 102, 114, 127, 136.5, 146.2, 152.5, 159, 164.7

Mass spectrometry $C_{15}H_{17}ClN_2O_4$ Na^+: 347.076905, calculated: 347.07745.

4-(3-Bromo-phenyl)-6-chloromethyl-2-oxo-1,2,3,4-tetrahydro-pyrimidine-5-carboxylic acid ethyl ester **1c**.

Physical State: Solid yellowish white; Melting point: 172 ° C; Yield: 38%

IR: 3300 cm^{-1}, 1710 cm^{-1} e 1670 cm^{-1}

^{1}H NMR (500MHz, DMSO-d_6): δ, 1.13 (t, J=6.94 Hz, 3H), 4.05 (m, J= 7.25 Hz, 2H), 4.6 (d, J= 10.7Hz, 1H), 4.77 (d, J= 10.4Hz, 1H), 5.2 (sl, 1H), 7.26 (d, J=7.57, 1H), 7.34 (t,

CC BY

J=7.8Hz, 1H), 7.43 (s, 1H), 7.49 (d, J=8.2, 1H), 7.92 (s, 1H), 9.6 (s, 1H). ^{13}C NMR (125 MHz, DMSO-d_6): δ, 14.3, 53.9, 60.5, 101, 122, 125, 129.7, 130, 131, 147, 147.1, 152.2, 164.4
Mass spectrometry $C_{14}H_{14}ClN_2O_3$ Na^+: 394.976853, calculated: 394.97740.

6-Chloromethyl-4-(4-chloro-phenyl)-2-oxo-1,2,3,4-tetrahydro-pyrimidine-5-carboxylic acid ethyl ester **1d**
Physical State: White solid; Melting point: 178 ° C; Yield: 24%
IR: 3300 cm^{-1}, 1710 cm^{-1} e 1670 cm^{-1}
^{1}H NMR (500MHz, DMSO-d_6): δ, 1.12 (t, J=7.25 Hz, 3H), 4.05 (q, J= 7.25 Hz, 2H), 4.58 (d, J= 10.7Hz, 1H), 4.78 (d, J= 10.7Hz, 1H), 5.2 (sl, 1H), 7.27 (2d, J=8.2Hz, 2H), 7.42 (d, J=8.5Hz, 2H), 7.91 (s, 1H), 9.57 (s, 1H). ^{13}C NMR (125 MHz, DMSO-d_6): δ 14.3, 53.7, 60.5, 101.7, 128, 129, 132.6, 143, 146.8, 152.3, 164.2
Mass spectrometry $C_{14}H_{14}ClN_2O_3$ Na^+: 351.027368, calculated: 351.02792

6-Chloromethyl-4-(2-methoxy-phenyl)-2-oxo-1,2,3,4-tetrahydro-pyrimidine-5-carboxylic acid ethyl ester **1e**
Physical State: White solid; Melting point: 190 ° C; Yield: 66%
IR: 3300 cm^{-1}, 1710 cm^{-1} e 1670 cm^{-1}
^{1}H NMR (500MHz, DMSO-d_6): δ, 1.06 (t, J=7.25 Hz, 3H), 3.73 (s, 3H), 3.93 (m, J= 7.26 Hz, 2H), 4.66 (d, J= 10.7Hz, 1H), 4.76 (d, J= 10.7Hz, 1H), 5.53 (sl, 1H), 6.9 (t, J=6.95Hz, 1H), 7 (d, J=8.2, 1H), 7.12 (2d, J=6Hz, 1H), 7.24-7.28 (m, 1H), 7.45 (s, 1H), 9.41 (s, 1H). ^{13}C RMN (125 MHz, DMSO-d_6): δ, 13.7, 53.7, 53.77, 60.5, 101.7, 128.7, 128.9, 129, 129.2, 132, 143.3, 146.8, 152.3, 164.3
Mass spectrometry $C_{15}H_{17}ClN_2O_4$ Na^+: 347.076905, calculated: 347.07745.

4-(4-Bromo-phenyl)-6-chloromethyl-2-oxo-1,2,3,4-tetrahydro-pyrimidine-5-carboxylic acid ethyl ester **1f**
Physical State: White solid; Melting point: 177 ° C; Yield: 17%
IR: 3300 cm^{-1}, 1710 cm^{-1} e 1670 cm^{-1}
^{1}H NMR (500MHz, DMSO-d_6): δ, 1.12 (t, J=7.25 Hz, 3H), 4.05 (q, J= 7.25 Hz, 2H), 4.58 (d, J= 10.7Hz, 1H), 4.78 (d, J= 10.7Hz, 1H), 5.19 (sl, 1H), 7.21 (2d, J=8.2, 2H), 7.56 (2d, J=8.5Hz, 2H), 7.90 (s, 1H), 9.57 (s, 1H). ^{13}C RMN (125 MHz, DMSO-d_6): δ, 14.3, 52.7, 60.5, 101.7, 121, 129, 131, 143.7, 146.8 152.3, 164.5
Mass spectrometry $C_{14}H_{14}ClN_2O_3$ Na^+: 394.976853, calculated: 394.97740.

6-Chloromethyl-2-oxo-4-(3,4,5-trimethoxy-phenyl)-1,2,3,4-tetrahydro-pyrimidine-5-carboxylic acid ethyl ester **1g**
Physical State: White solid; Melting point: 190 ° C; Yield: 63.2%
IR: 3300 cm^{-1}, 1710 cm^{-1} e 1670 cm^{-1}
^{1} H NMR (500MHz, DMSO-d_6): δ, 1.14 (t, J=7.25 Hz, 3H), 3.64 (s, 3H), 3.73 (s, 6H), 4.05 (q, J= 7.25 Hz, 2H), 4.64 (d, J= 10.4Hz, 1H), 4.81 (d, J= 10.7Hz, 1H), 5.15 (sl, 1H), 6.57 (sl, 2H), 7.84 (s, 1H), 9.52 (s, 1H). ^{13}C NMR (125 MHz, DMSO-d_6): δ, 14.4, 54.1, 56.2, 60.4, 60.5, 101, 103, 137, 139.8, 152.5, 153.3, 164.3
Mass spectrometry $C_{17}H_{21}ClN_2O_6$ Na^+: 407.096687, calculated: 407.09858

6-Chloromethyl-4-(2-chloro-phenyl)-2-oxo-1,2,3,4-tetrahydro-pyrimidine-5-carboxylic acid ethyl ester **1h**
Physical State: White solid; Melting point: 190 ° C; Yield: 39%
IR: 3300 cm^{-1}, 1710 cm^{-1} e 1670 cm^{-1}
^{1}H NMR (500MHz, DMSO-d_6): δ, 1.03 (t, J=7.25 Hz, 3H), 3.96 (q, J= 7.25 Hz, 2H), 4.70 (d, J=10.4Hz, 1H), 4.77 (d, J=10.7Hz, 1H), 5.69 (sl, 1H), 7.29-7.45 (m, 4H), 7.86 (s, 1H), 9.59 (s, 1H). ^{13}C NMR (125 MHz, DMSO-d_6): δ, 14.2, 51.8, 60.3, 60.5, 100, 128.3, 129.1, 129.8, 129.9, 132, 141, 147, 151.7, 164.3
Mass spectrometry $C_{14}H_{14}ClN_2O_3$ Na^+: 351.027368, calculated: 351.02792

6-Chloromethyl-4-(2,4-dimethoxy-phenyl)-2-oxo-1,2,3,4-tetrahydro-pyrimidine-5-carboxylic acid ethyl ester **1i**
Physical State: White solid; Melting point: 175 ° C; Yield: 50%
IR: 3300 cm^{-1}, 1710 cm^{-1} e 1670 cm^{-1}
^{1}H NMR (500MHz, DMSO-d_6):δ, 1.08 (t, J=7.25 Hz, 3H), 3.74 (s, 3H), 3.78 (s, 3H), 3.98 (q, J= 7.25 Hz, 2H), 4.67 (d, J= 10.4Hz, 1H), 4.74 (d, J= 10.7Hz, 1H), 5.44 (sl, 1H), 6.45 (2d, J=8.5Hz, 2H), 6.55 (sl, 1H), 7 (d, J=8.5Hz, 1H), 7.39 (s, 1H), 9.37 (s, 1H). ^{13}C NMR (125 MHz, DMSO-d_6): δ, 14.4, 53.86, 55.83, 60.4, 102.2, 110.7, 112.2, 118.6, 136.8, 146.3, 148.7, 149, 152.5, 164.7
Mass spectrometry $C_{16}H_{19}ClN_2O_5$ Na^+: 377.087470, calculated: 377.08802

6-Chloromethyl-4-(3,4-dimethoxy-phenyl)-2-oxo-1,2,3,4-tetrahydro-pyrimidine-5-carboxylic acid ethyl ester **1j**
Physical State: White solid; Melting point: 155 ° C; Yield: 53%
IR: 3300 cm^{-1}, 1710 cm^{-1} e 1670 cm^{-1}
^{1}H NMR (500MHz, DMSO-d_6): δ, 1.08 (t, J=7.25 Hz, 3H), 3.74 (s, 3H), 3.78 (s, 3H), 3.98 (q, J= 7.25 Hz, 2H), 4.67 (d, J= 10.4Hz, 1H), 4.74 (d, J= 10.7Hz, 1H), 5.44 (sl, 1H), 6.45 (2d, J=8.5Hz, 2H), 6.55 (sl, 1H), 7 (d, J=8.5Hz, 1H), 7.39 (s, 1H), 9.37 (s, 1H). ^{13}C NMR (125 MHz, DMSO-d_6): δ, 14.4, 53.86, 55.83, 60.4, 102.2, 110.7, 112.2, 118.6, 136.8, 146.3, 148.7, 149, 152.5, 164.7
Mass spectrometry $C_{16}H_{19}ClN_2O_5$ Na^+: 377.087470, calculated: 377.08802

6-Chloromethyl-4-(4-hydroxy-3-methoxy-phenyl)-2-oxo-1,2,3,4-tetrahydro-pyrimidine-5-carboxylic acid ethyl ester **1k**
Physical State: White solid; Melting point: 159 ° C; Yield: 60%
IR: 3300 cm^{-1}, 1710 cm^{-1} e 1670 cm^{-1}
^{1}H NMR (500MHz, DMSO-d_6): δ, 1.14 (t, J=6.94Hz, 3H), 3.73 (s, 3H), 4.05 (q, J= 6.94 Hz, 2H), 4.7 (q, J= 10.4Hz, 2H), 5.1 (sl, 1H), 6.7 (d, J= 7.8Hz, 1H), 6.81 (d, J=1.58Hz, 1H), 7.7 (s, 1H), 8.99 (s, 1H), 9.43 (s, 1H). ^{13}C NMR (125 MHz, DMSO-d_6): δ, 14.4, 53.96, 55.96, 60.4, 102.4, 111.1, 115.8, 118.9, 135.4, 146.1, 146.5, 147.8, 152.5, 164.7
Mass spectrometry $C_{15}H_{17}ClN_2O_5$ Na^+: 363.071820, calculated: 363.07237

B. General methodology for synthesis of pyrrolo-[3,4-*d*]-pyrimidinodiones 2a-k and

3a-k:
The reaction was performed in vials with a screw cap (identical to the previous reaction), in which was added the DHPMs, amine (benzylamine or phenylethyamine) and triethylamine in the ratio 1:1:3, respectively, and 1 ml of methanol. The reaction was maintained at 60 ° C for 48-96 hours depending on the starting DHPM. The reaction was cooled and the precipitate was filtered. When no precipitate formed spontaneously, the solution was poured into water-ice and then filtered. The solid obtained was washed with cold methanol.

6-Phenethyl-4-phenyl-3,4,6,7-tetrahydro-1*H*-pyrrolo[3,4-*d*]pyrimidine-2,5-dione **2a**
Reaction time: 48 hours
Physical State: White solid; Melting point: 290 ° C; Yield: 64%
IR: 3300 cm^{-1}, 1650 cm^{-1} e 1670 cm^{-1}
^{1}H NMR (500MHz, DMSO-d_6): δ, 2.74 (t, J= 7.25Hz, 2H), 3.45 (m, 2H), 3.84 (s, 2H), 5.15 (sl, 1H), 7.18-7.37 (m, 10H), 7.53 (s, 1H), 9.52 (s, 1H). ^{13}C NMR (125 MHz, DMSO-d_6): δ, 34.7, 43, 47.7, 53.5, 103, 126.6, 126.9, 127.8, 128.8, 128.84, 129.1. 139.7, 144, 150.2, 152.3, 168.4.
Mass spectrometry $C_{20}H_{19}N_3O_2$ Na^+: 356.136948, calculated: 356.13750

4-(4-Methoxy-phenyl)-6-phenethyl-3,4,6,7-tetrahydro-1*H*-pyrrolo[3,4-*d*]pyrimidine-2,5-dione **2b**
Reaction time: 96 hours
Physical State: White solid; Melting point: 260 ° C; Yield: 68.8%
IR: 3300 cm^{-1}, 1650 cm^{-1} e 1670 cm^{-1}
^{1}H NMR (500MHz, DMSO-d_6): δ, 2.7 (t, J= 7.25Hz, 2H), 3.4 (s, 2H), 3.7 (s, 3H), 3.8 (m, 2H), 5.11 (sl, 1H), 6.9-7.3 (m, 9H), 7.48 (s, 1H), 9.49 (s, 1H). ^{13}C NMR (125 MHz, DMSO-d_6): δ, 3.7, 43.1, 47.7, 52.9, 55.5, 103.9, 114.1, 126.6, 128, 128.5, 129.1, 136.2, 139.7, 149.9, 152.3, 159, 168.4
Mass spectrometry $C_{21}H_{21}N_3O_3$ Na^+: 386.147512, calculated: 386.14806

4-(3-Bromo-phenyl)-6-phenethyl-3,4,6,7-tetrahydro-1*H*-pyrrolo[3,4-*d*]pyrimidine-2,5-dione **2c**
Reaction time: 48 hours
Physical State: White solid; Melting point: 278 ° C; Yield: 54%
IR: 3300 cm^{-1}, 1650 cm^{-1} e 1670 cm^{-1}
^{1}H NMR (500MHz, DMSO-d_6): δ, 2.75 (t, J= 7.25Hz, 2H), 3.48(m, 2H), 3.8 (sl, 3H), 5.21 (sl, 1H), 7.18-7.47(m, 10H), 9.6 (s, 1H). ^{13}C NMR (125 MHz, DMSO-d_6): δ, 34.7, 43.1, 47.8, 53, 103, 122, 126, 126.6, 128.8, 129.6, 130.7, 131.1, 139.6, 146.6, 150.5, 152.1, 168.3
Mass spectrometry $C_{20}H_{18}BrN_3O_3$ Na^+: 434.047460, calculated: 434.04801

4-(2-Methoxy-phenyl)-6-phenethyl-3,4,6,7-tetrahydro-1*H*-pyrrolo[3,4-*d*]pyrimidine-2,5-dione **2e**
Reaction time: 96 hours
Physical state: Yellowish solid; Melting point: 210 ° C; Yield: 57.3%

IR: 3300 cm^{-1}, 1650 cm^{-1} e 1670 cm^{-1}

^{1}H NMR (500MHz, DMSO-d_6): δ, 2.77 (t, J= 7,25Hz, 2H), 3.79 (s, 3H), 3.8(sl, 2H), 5.43 (sl, 1H), 6.92-7.31(m, 9H), 8.74 (s, 1H), 9.74 (s, 1H). ^{13}C NMR (125 MHz, DMSO-d_6): δ, 34.7, 43, 47.7, 48.5, 56, 102, 103, 120.8, 126, 128.4, 128.8, 129.1, 129.4, 139.7, 151, 152, 157, 168.4

Mass spectrometry $C_{21}H_{21}N_3O_3$ Na^+: 386.147512, calculated: 386.14806

4-(4-Bromo-phenyl)-6-phenethyl-3,4,6,7-tetrahydro-1*H*-pyrrolo[3,4-*d*]pyrimidine-2,5-dione **2f**

Reaction time: 48 hours

Physical State: White solid; Melting point: 305 ° C; Yield: 70%

IR: 3300 cm^{-1}, 1650 cm^{-1} e 1670 cm^{-1}

^{1}H NMR (500MHz, DMSO-d_6): δ, 2.72 (t, J= 7.25Hz, 2H), 3.46(m, 2H), 3.86 (s, 2H), 5.18 (sl, 1H), 7.17-7.58 (m, 9H + 1H), 9.58 (s, 1H). ^{13}C NMR (125 MHz, DMSO-d_6): δ 34.7, 43.1, 47.7, 53, 103.1, 120.9, 126.6, 128.8, 129.1, 129.2, 131.7, 139.7, 143.3, 150.3, 152, 168

Mass spectrometry $C_{20}H_{18}BrN_3O_3$ Na^+: 434.047460, calculated: 434.04801

6-Phenethyl-4-(3,4,5-trimethoxy-phenyl)-3,4,6,7-tetrahydro-1*H*-pyrrolo[3,4-*d*]pyrimidine-2,5-dione **2g**

Reaction time: 48 hours

Physical State: White solid; Melting point: 240 ° C; Yield: 70%

IR: 3300 cm^{-1}, 1650 cm^{-1} e 1670 cm^{-1}

^{1}H NMR (500MHz, DMSO-d_6): δ 2.77 (t, J= 7.25Hz, 2H), 3.49(m, 2H), 3.64 (s, 3H), 3.74 (s, 6H), 3.86 (s, 2H), 5.16 (sl, 1H), 6.63 (sl, 2H), 7.19-7.28 (m, 5H), 7.53 (s, 1H), 9.52 (s, 1H). ^{13}C NMR (125 MHz, DMSO-d_6): δ, 34.7, 43.1, 47.7, 53.5, 56.3, 60.4, 103.3, 103.9, 126.6, 128.8, 129.1, 137.2, 139.6, 139.7, 150.3, 152.3, 153.2, 168.6

Mass spectrometry $C_{23}H_{25}N_3O_5$ Na^+: 446.168642, calculated: 446.16919

4-(2-Chloro-phenyl)-6-phenethyl-3,4,6,7-tetrahydro-1*H*-pyrrolo[3,4-*d*]pyrimidine-2,5-dione **2h**

Reaction time: 48 hours

Physical State: White solid; Melting point: 300-310 ° C; Yield: 55.6%

IR: 3300 cm^{-1}, 1650 cm^{-1} e 1670 cm^{-1}

^{1}H NMR (500MHz, DMSO-d_6): δ, 2.72 (t, J= 7.25Hz, 2H), 3.44(m, 2H), 3.87 (s, 2H), 5.58 (sl, 1H), 7.17-7.47 (m, 10H), 9.61 (s, 1H). ^{13}C NMR (125 MHz, DMSO-d_6): δ, 34.7, 43.1, 47.8, 51.7, 102, 126.6, 127, 128.8, 129, 130, 132.4, 139.7, 140.3, 150.1, 151.8, 168

Mass spectrometry $C_{20}H_{18}ClN_3O_2$ Na^+: 390.097975, calculated: 390.09852

4-(3,4-Dimethoxy-phenyl)-6-phenethyl-3,4,6,7-tetrahydro-1*H*-pyrrolo[3,4-*d*]pyrimidine-2,5-dione **2j**

Reaction time: 96 hours

Physical State: White solid; Melting point: 156 ° C; Yield: 54%

IR: 3300 cm^{-1}, 1650 cm^{-1} e 1670 cm^{-1}

^{1}H NMR (500MHz, DMSO-d_6): δ, 2.77 (t, J= 7.25Hz, 2H), 3.4(m, 2H), 3.73 (s, 3H), 3.74 (s, 3H), 3.8 (s, 2H), 5.12 (sl, 1H), 6.75-6.95 (m, 3H) 7.15-7.30 (m, 5H), 7.49 (s, 1H), 9.49 (s, 1H). ^{13}C NMR (125 MHz, DMSO-d_6): δ, 34.7, 43, 47.7, 53, 55.8, 56, 103, 111, 112, 118, 126, 128.8, 129, 136.5, 139.7, 148.6, 148.9, 150, 168.6
Mass spectrometry $C_{22}H_{23}N_3O_4$ Na^+: 416.158077, calculated: 416.15863

6-Benzyl-4-phenyl-3,4,6,7-tetrahydro-1*H*-pyrrolo[3,4-*d*]pyrimidine-2,5-dione **3a**
Reaction time: 96 hours
Physical State: White solid; Melting point: 270 ° C; Yield: 62%
IR: 3300 cm^{-1}, 1650 cm^{-1} e 1670 cm^{-1}
^{1}H NMR (500MHz, DMSO-d_6): δ, 3.80 (d, J=18Hz, 1H), 3.9 (d, J=18Hz, 1H), 4.34 (d, J=15Hz ,1H), 4.53 (d, J=15Hz ,1H), 5.23 (sl, 1H), 7.1-7.39 (m, 10H), 7.6 (s, 1H), 9.55 (s, 1H). ^{13}C NMR (125 MHz, DMSO-d_6): δ, 45.1, 47.2, 53.6, 103.4, 126.9, 128, 128.9, 129, 138.5, 144, 150.6, 152.2, 168.7
Mass spectrometry $C_{19}H_{17}N_3O_2$ Na^+: 342.121297, calculated: 342.12185

6-Benzyl-4-(4-methoxy-phenyl)-3,4,6,7-tetrahydro-1*H*-pyrrolo[3,4-*d*]pyrimidine-2,5-dione **3b**
Reaction time: 96 hours
Physical State: White solid; Melting point: 265 ° C; Yield: 40%
IR: 3300 cm^{-1}, 1650 cm^{-1} e 1670 cm^{-1}
^{1}H NMR (500MHz, DMSO-d_6): δ, 3.74(s, 3H), 3.77 (d, J=18Hz, 1H), 3.9 (d, J=18Hz, 1H), 4.34 (d, J=15Hz ,1H), 4.53 (d, J=15Hz ,1H), 5.18 (sl, 1H), 6.9 (d, J=8.5Hz, 2H), 7.18 (d, J=7Hz, 2H), 7.23-7.34 (m, 5H), 7.5 (s, 1H), 9.6 (s, 1H). ^{13}C NMR (125 MHz, DMSO-d_6): δ, 45.1, 47.2, 53, 55.5, 103.6, 114.2, 127.7, 128, 129, 136, 138.5, 150.4, 152.2, 159.1, 168.8
Mass spectrometry $C_{20}H_{19}N_3O_3$ Na^+: 372.131862, calculated: 372.13241

6-Benzyl-4-(4-chloro-phenyl)-3,4,6,7-tetrahydro-1*H*-pyrrolo[3,4-*d*]pyrimidine-2,5-dione **3d**
Reaction time: 96 hours
Physical State: White solid; Melting point: 215 ° C; Yield: 70%
IR: 3300 cm^{-1}, 1650 cm^{-1} e 1670 cm^{-1}
^{1}H NMR (400MHz, DMSO-d_6): δ, 3.82 (d, J=18Hz, 1H), 3.88 (d, J=18Hz, 1H), 4.36 (d, J=15Hz, 1H), 4.52 (d, J=15Hz, 1H), 5.27 (sl, 1H), 7.19-7.43 (m, 9H), 7.63 (s,1H), 9.58 (s, 1H). ^{13}C NMR (100MHz, DMSO-d_6): δ, 45.2, 47.2, 53, 102.9, 127.6, 127.9, 128.8, 129, 132.4, 138.4, 142.8, 150.7, 152, 168.6
Mass spectrometry $C_{19}H_{16}ClN_3O_2$ Na^+: 376.082325, calculated: 376.08287

6-Benzyl-4-(2-methoxy-phenyl)-3,4,6,7-tetrahydro-1H-pyrrolo[3,4-d]pyrimidine-2,5-dione **3e**
Reaction time: 48 hours
Physical state: Yellowish solid; Melting point: 142 ° C; Yield: 54%
IR: 3300 cm^{-1}, 1650 cm^{-1} e 1670 cm^{-1}

^{1}H NMR (500MHz, DMSO-d_6): δ, 3.8 (s, 3H), 3.88 (m, 2H), 4.34 (d, J=15Hz , 1H), 4.48 (d, J=15Hz , 1H), 5.47 (sl, 1H), 6.9-7.3 (m, 10H), 9.4 (s, 1H). ^{13}C NMR (125 MHz, DMSO-d_6): δ, 45.1, 47.2, 48.7, 56, 102.2, 111.9, 120.8, 127.6, 128.6, 129, 129.5, 131.1, 138.6, 151.5, 152.2, 157.2, 168.7

Mass spectrometry $C_{20}H_{19}N_3O_3$ Na^+: 372.131862, calculated: 372.13241

6-Benzyl-4-(4-bromo-phenyl)-3,4,6,7-tetrahydro-1*H*-pyrrolo[3,4-*d*]pyrimidine-2,5-dione **3f**

Reaction time: 96 hours

Physical State: White solid; Melting point: 280 ° C; Yield: 70%

IR: 3300 cm^{-1}, 1650 cm^{-1} e 1670 cm^{-1}

^{1}H NMR (500MHz, DMSO-d_6): δ, 3.8 (d, J=18Hz, 1H), 3.9 (d, J=18Hz, 1H), 4.34 (d, J=15Hz, 1H), 4.52 (d, J=15Hz, 1H), 5.25 (sl, 1H), 7.18 (d, J=7Hz, 2H), 7.27-7.35 (m, 5H), 7.58 (d, J= 8.5Hz, 2H), 7.6 (s, 1H), 9.6 (s, 1H). ^{13}C NMR (125 MHz, DMSO-d_6): δ, 45.1, 47.2, 53.1, 102.9, 121, 127.6, 127.9, 129, 131.7, 138.5, 143.3, 150.8, 152.1, 168.6

Mass spectrometry $C_{19}H_{16}BrN_3O_2$ Na^+: 420.031810, calculated: 420.03326

6-Benzyl-4-(3,4,5-trimethoxy-phenyl)-3,4,6,7-tetrahydro-1*H*-pyrrolo[3,4-*d*]pyrimidine-2,5-dione **3g**

Reaction time: 48 hours

Physical State: White solid; Melting point: 270 ° C; Yield: 40%

IR: 3300 cm^{-1}, 1650 cm^{-1} e 1670 cm^{-1}

^{1}H NMR (500MHz, DMSO-d_6): δ, 3.65 (s, 3H), 3.76 (s, 6H), 3.86 (m, 2H), 4.45 (d, J=15Hz, 1H), 4.49 (d, J=15Hz, 1H), 5.23 (sl, 1H), 6.86 (s, 2H), 7.19-7.34 (m, 9H), 7.58 (s, 1H), 9.54 (s, 1H). ^{13}C NMR (125 MHz, DMSO-d_6): δ, 45.1, 47.2, 53.5, 56.2, 60.4, 103, 104, 127.7, 127.9, 129, 129.2, 137.2, 138.6, 139.4, 150.9, 152.2, 153.2, 168.8

Mass spectrometry $C_{22}H_{23}N_3O_5$ Na^+: 432.152992, calculated: 432.15354

6-Benzyl-4-(2-chloro-phenyl)-3,4,6,7-tetrahydro-1*H*-pyrrolo[3,4-*d*]pyrimidine-2,5-dione **3h**

Reaction time: 96 hours

Physical State: White solid; Melting point: 258 ° C; Yield: 10%

IR: 3300 cm^{-1}, 1650 cm^{-1} e 1670 cm^{-1}

^{1}H NMR (500MHz, DMSO-d_6): δ, 3.8 (d, J=18Hz, 1H), 3.92 (d, J=18Hz, 1H), 4.33 (d, J=15Hz, 1H) 4.5 (d, J=15Hz, 1H), 5.64 (sl, 1H), 7.16-7.43 (m, 9H), 7.52 (s, 1H), 9.6 (s, 1H). ^{13}C NMR (125 MHz, DMSO-d_6): δ, 45, 47.2, 51.8, 102.9, 121, 127.6, 127.9, 129, 131.7, 138.5, 143.3, 150.8, 152.1, 168.6

Mass spectrometry $C_{19}H_{16}ClN_3O_2$ Na^+: 376.082325, calculated: 376.08287

6-Benzyl-4-(2,4-dimethoxy-phenyl)-3,4,6,7-tetrahydro-1*H*-pyrrolo[3,4-*d*]pyrimidine-2,5-dione **3i**

Reaction time: 96 hours

Physical State: White solid; Melting point: 270 ° C; Yield: 84.4%

IR: 3300 cm^{-1}, 1650 cm^{-1} e 1670 cm^{-1}

^{1}H NMR (500MHz, DMSO-d_6): δ, 3.76(s, 3H), 3.78 (s, 3H), 3.82-3.92 (m, 2H), 4.37 (d, J=15Hz, 1H), 4.5 (d, J=15Hz, 1H), 5.4 (sl, 1H), 6.4 e 6.5 (2d, J= 8,5Hz, 2H), 6.57 (sl, 1H), 6.9-7.34 (m, 6H), 9.4 (s, 1H). ^{13}C NMR (125 MHz, DMSO-d_6): δ, 45.1, 47.2, 48.4, 55.7, 56, 99.1, 102.3, 105.1, 123.7, 127.6, 127.9, 129, 129.2, 138.6, 151.2, 152.2, 158.3, 160.3, 168.8

Mass spectrometry $C_{21}H_{21}N_3O_4$ Na^+: 402.142427, calculated: 402.14298

6-Benzyl-4-(3,4-dimethoxy-phenyl)-3,4,6,7-tetrahydro-1*H*-pyrrolo[3,4-*d*]pyrimidine-2,5-dione **3j**
Reaction time: 96 hours
Physical State: White solid; Melting point: 278 ° C; Yield: 64.2%
IR: 3300 cm^{-1}, 1650 cm^{-1} e 1670 cm^{-1}
^1H NMR (500MHz, DMSO-d_6): δ, 3.74(s, 6H), 3.82 (d, J=18Hz, 1H), 3.88 (d, J=18Hz, 1H), 4.41(d, J=15Hz, 1H), 4.5 (d, J=15Hz, 1H), 5.2 (sl, 1H), 6.81 e 6.82 (2d, J= 8.5Hz, 2H), 6.93-6.98 (m, 2H), 7.18 (d, J=7Hz, 2H), 7.2-7.35(m, 3H), 7.54 (s,1H), 9.5 (s, 1H). ^{13}C NMR (125 MHz, DMSO-d_6): δ, 45.2, 47.2, 53.1, 56, 103.5, 110.9, 112.1, 118.6, 127.6, 127.9, 129, 136.4, 138.6, 148.6, 149, 150.5, 152.2, 168.8
Mass spectrometry $C_{21}H_{21}N_3O_4$ Na^+: 402.142427, calculated: 402.14298

6-Benzyl-4-(4-hydroxy-3-methoxy-phenyl)-3,4,6,7-tetrahydro-1*H*-pyrrolo[3,4-*d*]pyrimidine-2,5-dione **3k**
Reaction time: 96 hours
Physical State: White solid; Melting point: 228 ° C; Yield: 50%
IR: 3300 cm^{-1}, 1650 cm^{-1} e 1670 cm^{-1}
^1H NMR (500MHz, DMSO-d_6): δ, 3.75(s, 3H), 3.8 (d, J=18Hz, 1H), 3.9 (d, J=18Hz, 1H), 4.4 (d, J=15Hz , 1H), 4.51 (d, J=15Hz , 1H), 5.14 (sl, 1H), 6.7 e 6.73 (2d, J=8.2Hz, 2H), 6.92 (s, 1H), 7.18-7.35 (m, 5H), 7.5 (s, 1H), 8.9 (s, 1H), 9.47 (s, 1H). ^{13}C NMR (125 MHz, DMSO-d_6): δ, 45.1, 47.1, 53.2, 56, 103.6, 111.4, 115.4, 119, 127.6, 127.9, 129, 135, 138.6, 146.4, 147.8, 150.4, 152.2, 168.9
Mass spectrometry $C_{20}H_{19}N_3O_4$ Na^+: 388.126777, calculated: 388.12733

C. HPLC chromatograms, FT-IR, ^1H NMR and ^{13}C NMR

HPLC assays were obtained using a Shimadzu LC- 20AT system with SPD-M20A DAD type detector, and a Merck Chromolith C18 column. The samples were solubilized in acetonitrile and an isocratic method was used. Mobile phase: 60 % (acetonitrile) and 40 % (H_2O + TFA 0.01%) with a flow of 2 mL/min.
FT-IR spectra were obtained in a Bruker Vertex 70 FT-IR apparatus. ^1H and ^{13}C NMR spectra (using the DEPT 135 technique) were obtained in a Bruker Avance 400/500 NMR spectrometer, using TMS as internal standard.
High resolution mass spectrometry results were obtained using a Bruker Daltonics LC-MS-microTOF apparatus (Oswaldo Cruz Foundation, Rio de Janeiro, RJ).

Compound 1a

C h r o m a t o g r a m

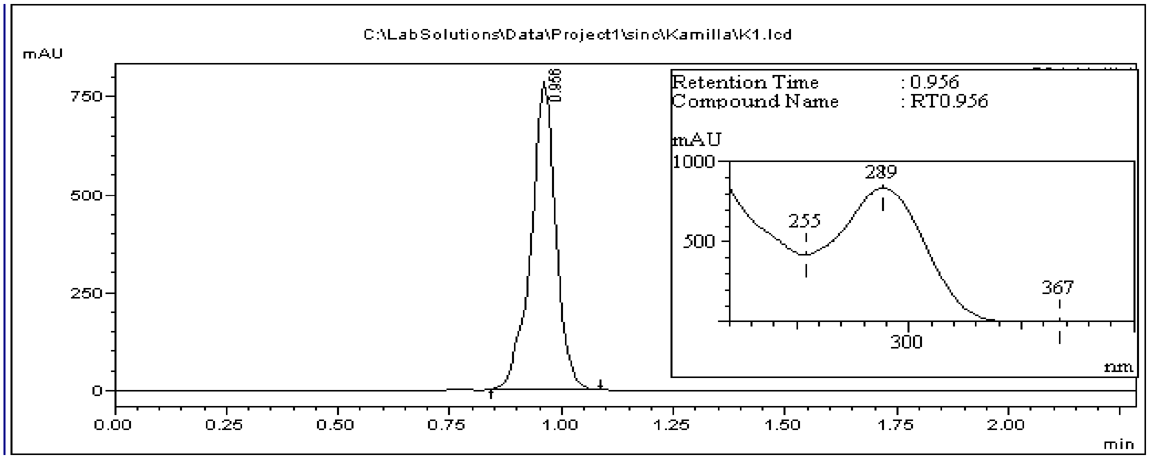

^{1}H NMR

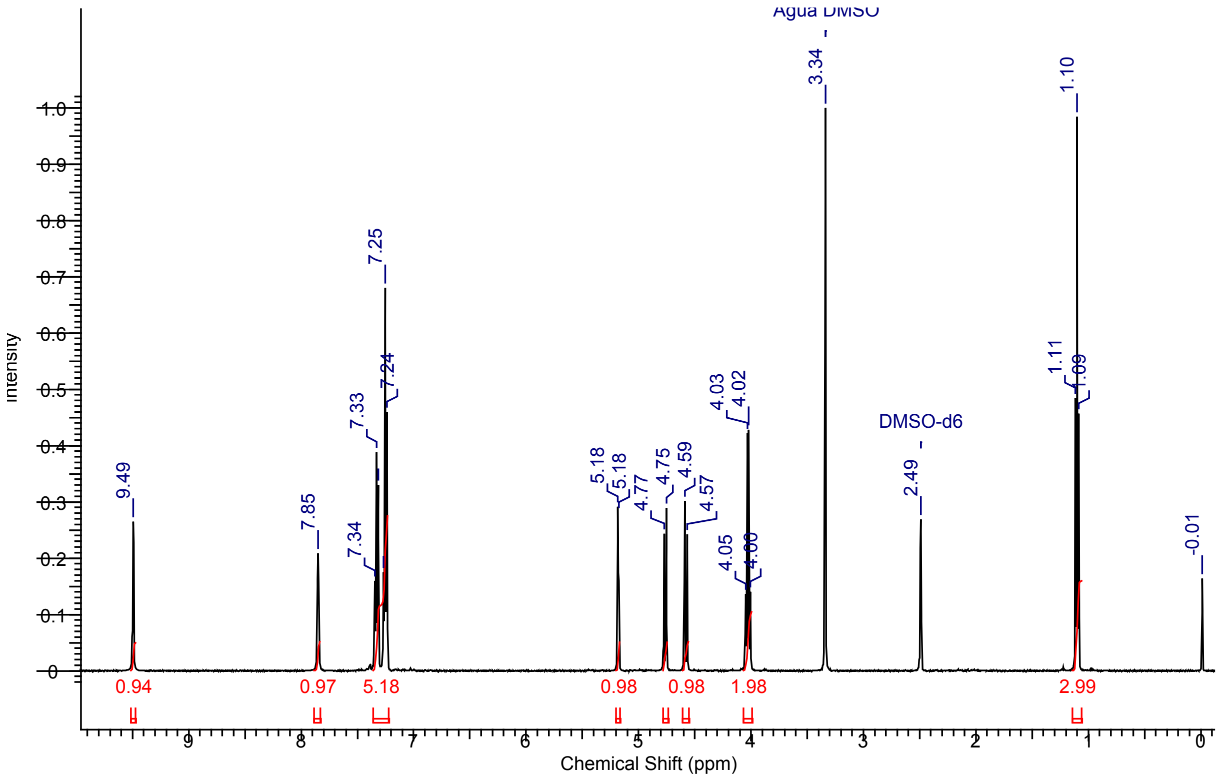

^{13}C NMR

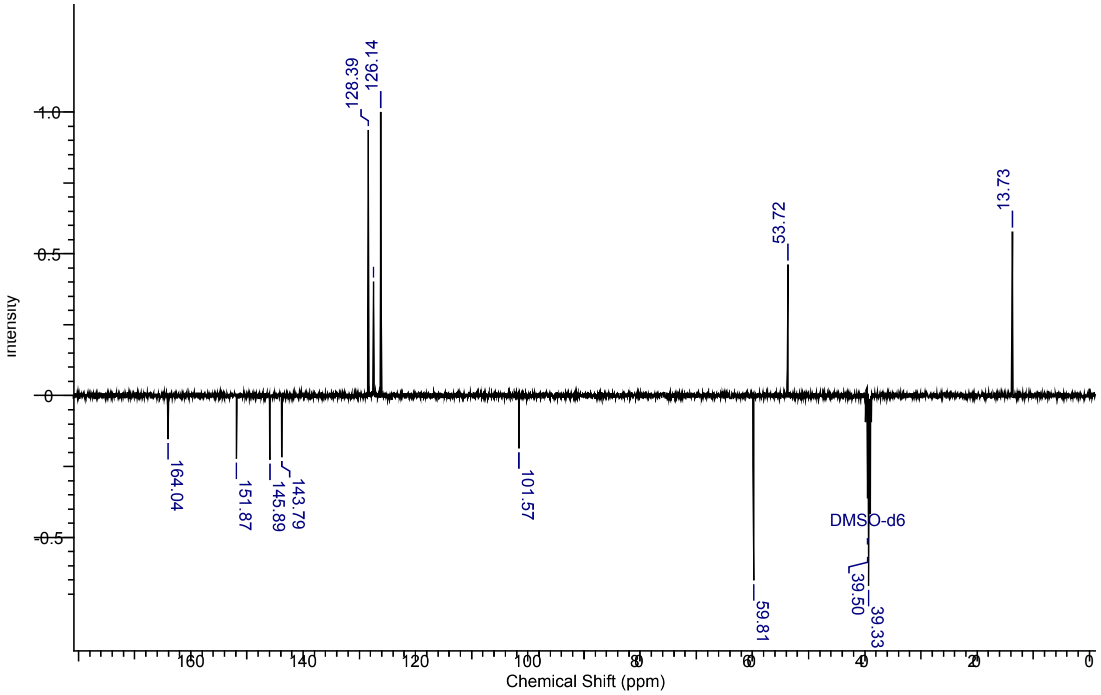

FT-IR

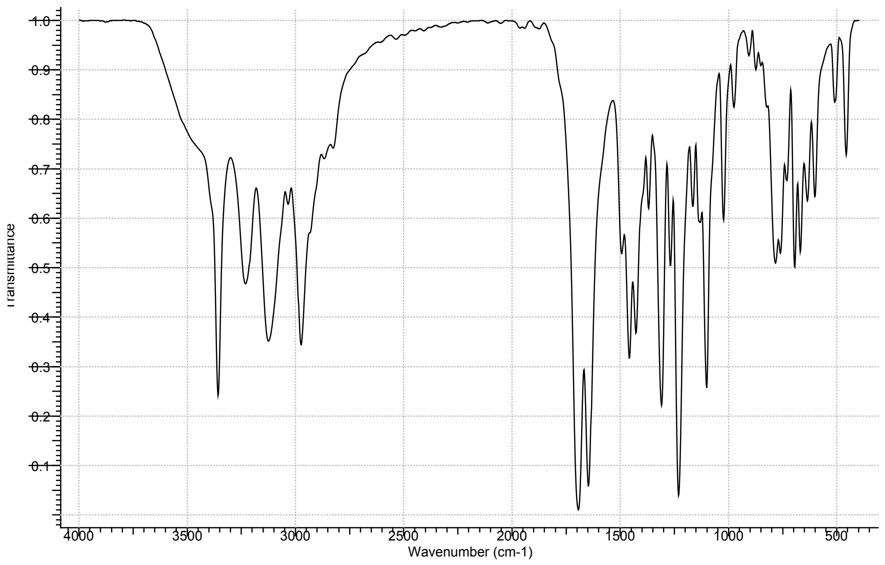

Mass Spectrometry

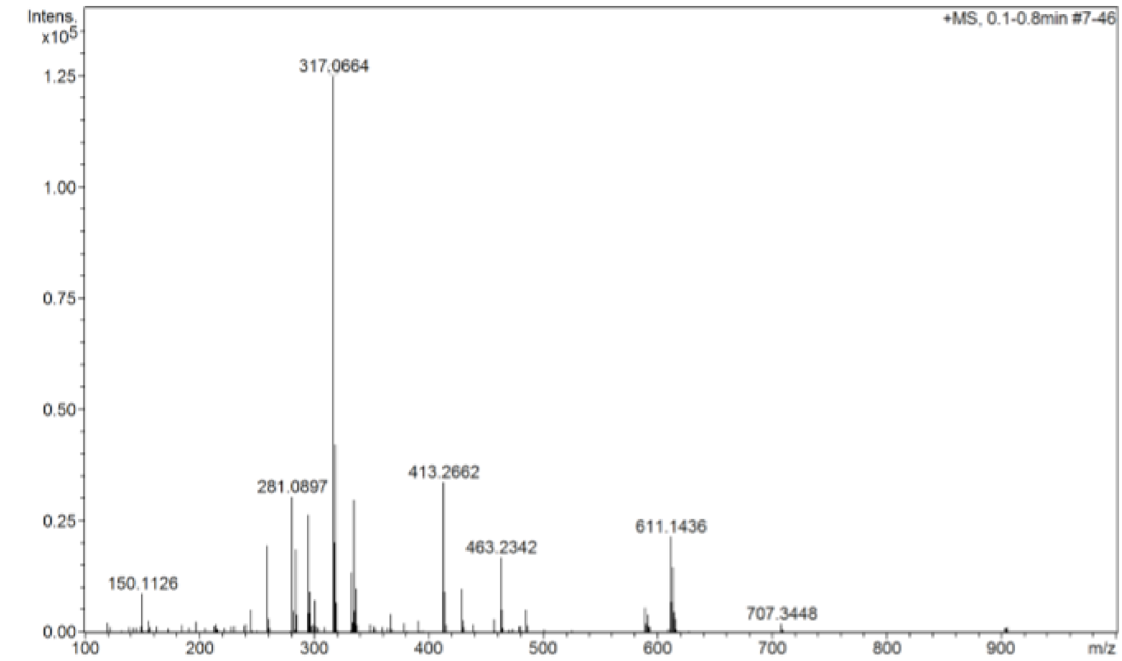

Meas. m/z	#	Ion Formula	m/z	err [ppm]	Mean err [ppm]	rdb	N-Rule	e⁻ Conf	mSigma	Std I	Std Mean m/z	Std I VarNorm	Std m/z Diff	Std Comb Dev
317.066441	1	C14H15ClN2NaO3	317.066341	-0.3	0.1	7.5	ok	even	0.6	0.8	n.a.	n.a.	n.a.	n.a.
	2	C10H11ClN8NaO	317.063655	-8.8	-9.7	8.5	ok	even	12.3	20.5	n.a.	n.a.	n.a.	n.a.
	3	C19H15ClNaO	317.070363	12.4	13.2	11.5	ok	even	24.4	37.1	n.a.	n.a.	n.a.	n.a.

Compound 1b

Chromatogram

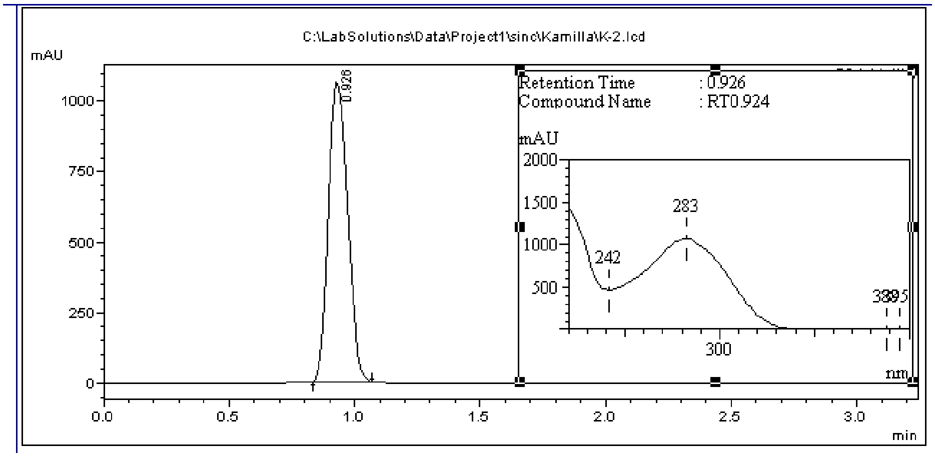

^{1}H NMR

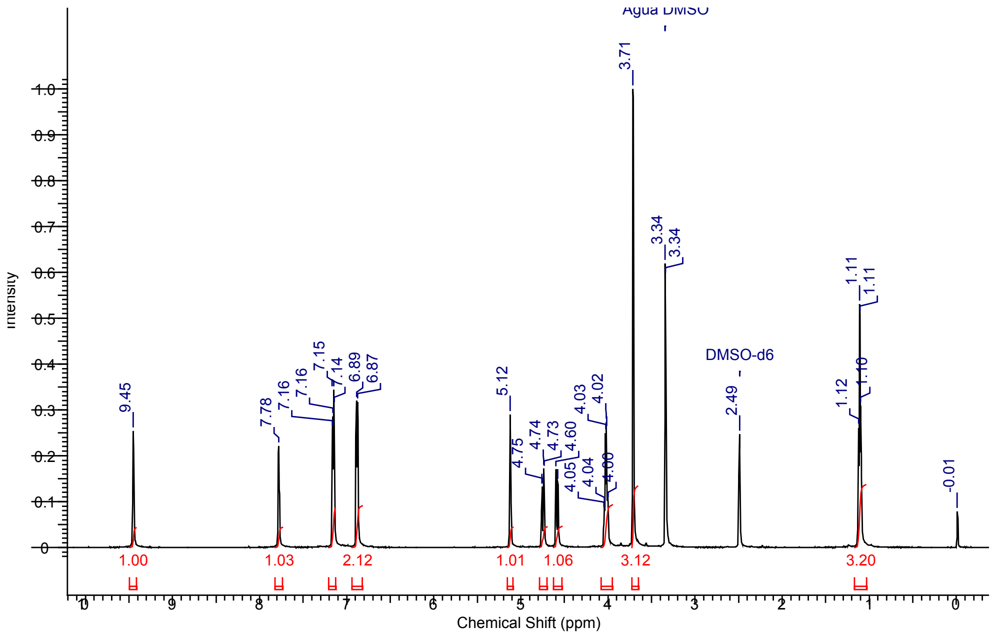
Agua DMSO
DMSO-d6
Intensity
Chemical Shift (ppm)

^{13}C NMR

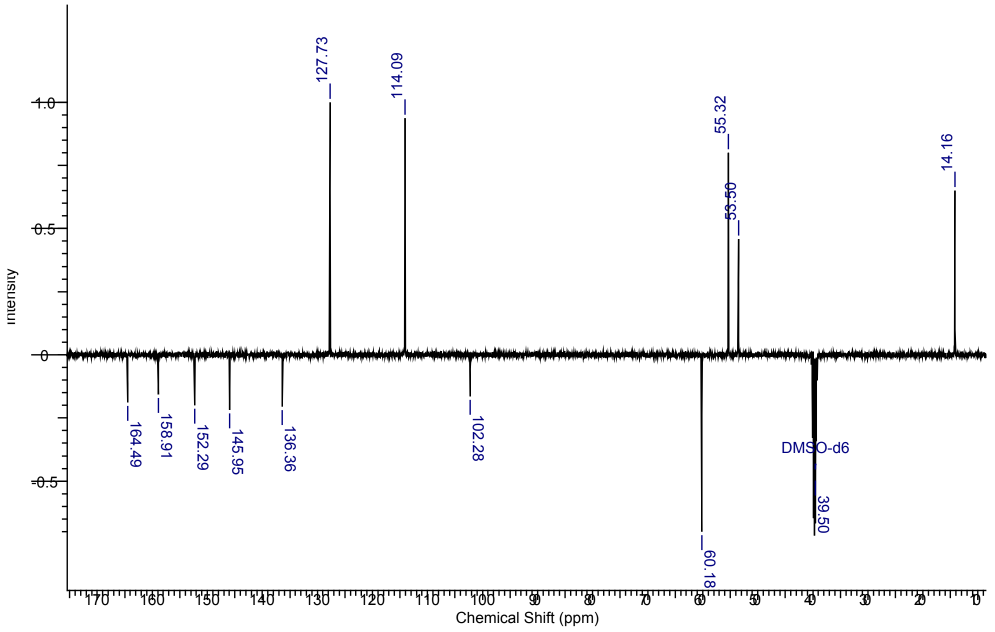
DMSO-d6
Intensity
Chemical Shift (ppm)

FT-IR

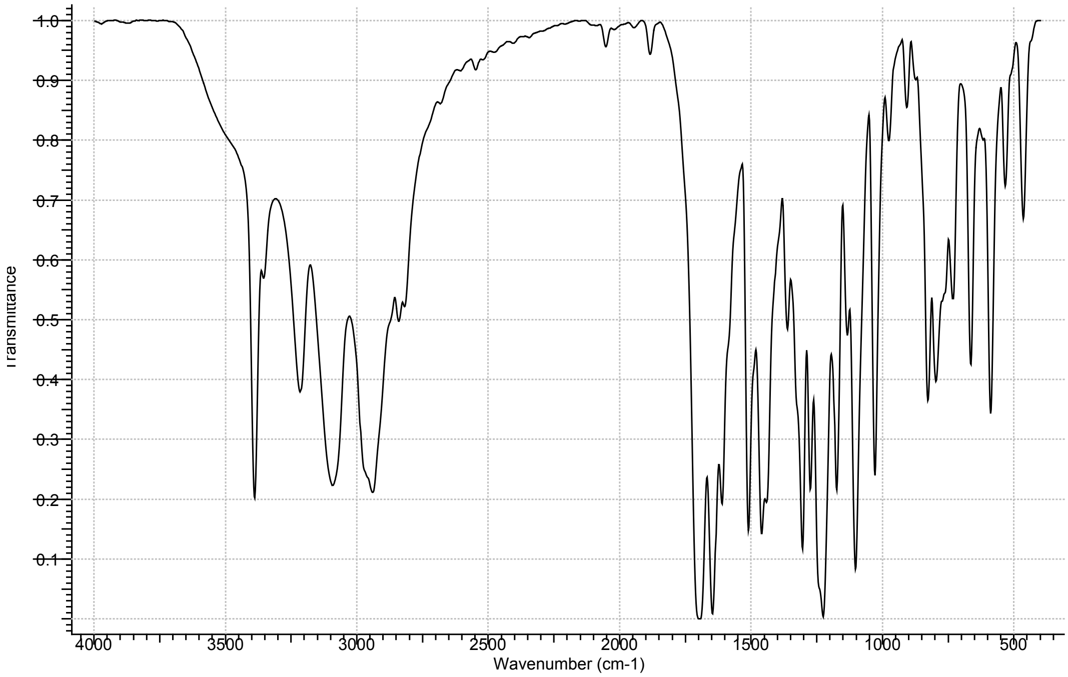

Mass Spectrometry

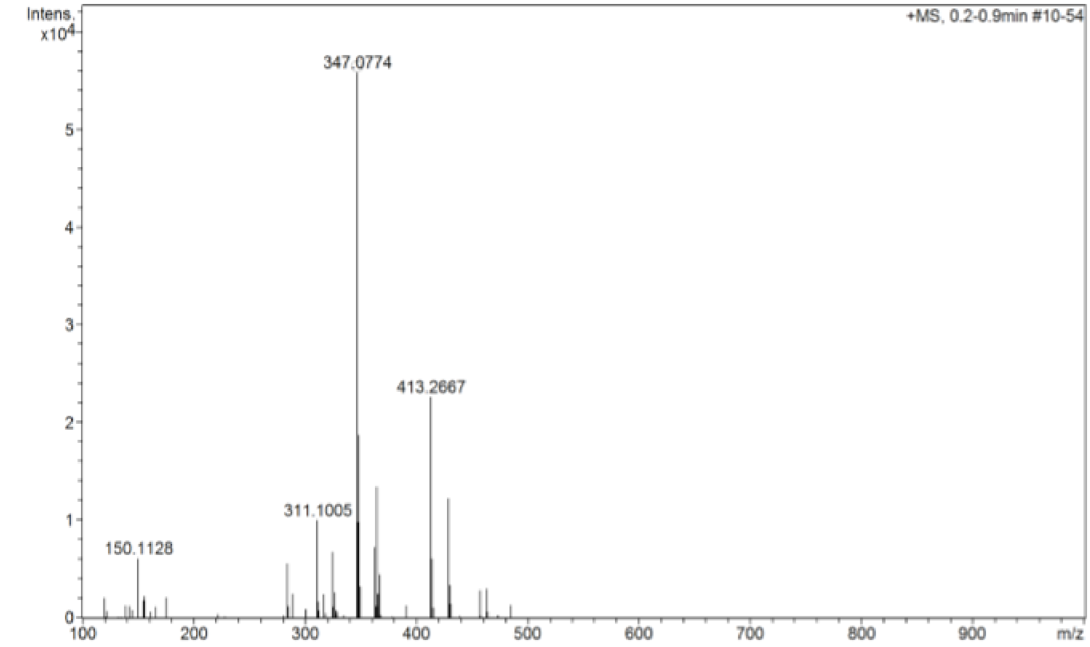

Meas. m/z	#	Ion Formula	m/z	err [ppm]	Mean err [ppm]	rdb	N-Rule	e⁻ Conf	mSigma	Std I	Std Mean m/z	Std I VarNorm	Std m/z Diff	Std Comb Dev
347.077357	1	C15H17ClN2NaO4	347.076905	-1.3	-1.3	7.5	ok	even	3.6	4.5	n.a.	n.a.	n.a.	n.a.
	2	C16H13ClN6Na	347.078243	2.6	2.0	12.5	ok	even	11.1	16.9	n.a.	n.a.	n.a.	n.a.
	3	C11H13ClN8NaO2	347.074220	-9.0	-10.2	8.5	ok	even	13.1	20.7	n.a.	n.a.	n.a.	n.a.
	4	C20H17ClNaO2	347.080928	10.3	1567.4	11.5	ok	even	21.6	38.4	n.a.	n.a.	n.a.	n.a.

Compound 1c

Chromatogram

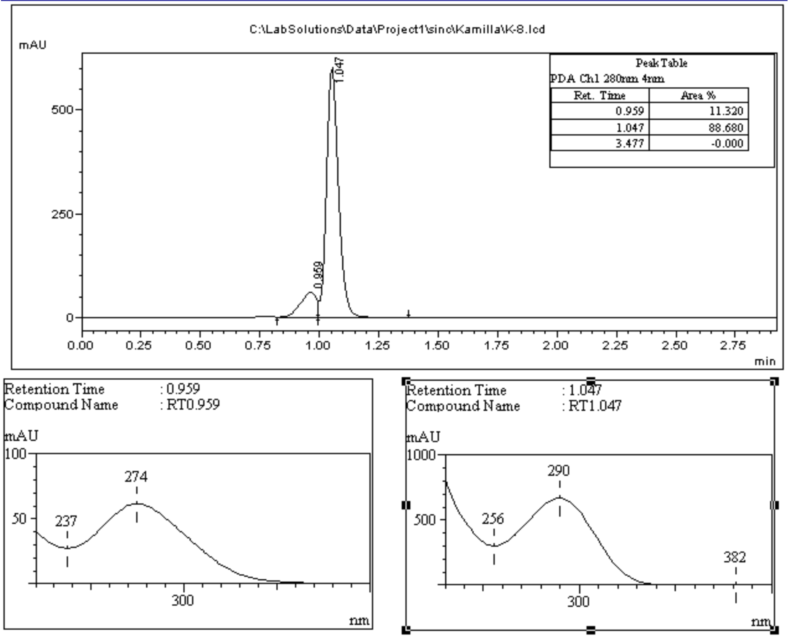

^{1}H NMR

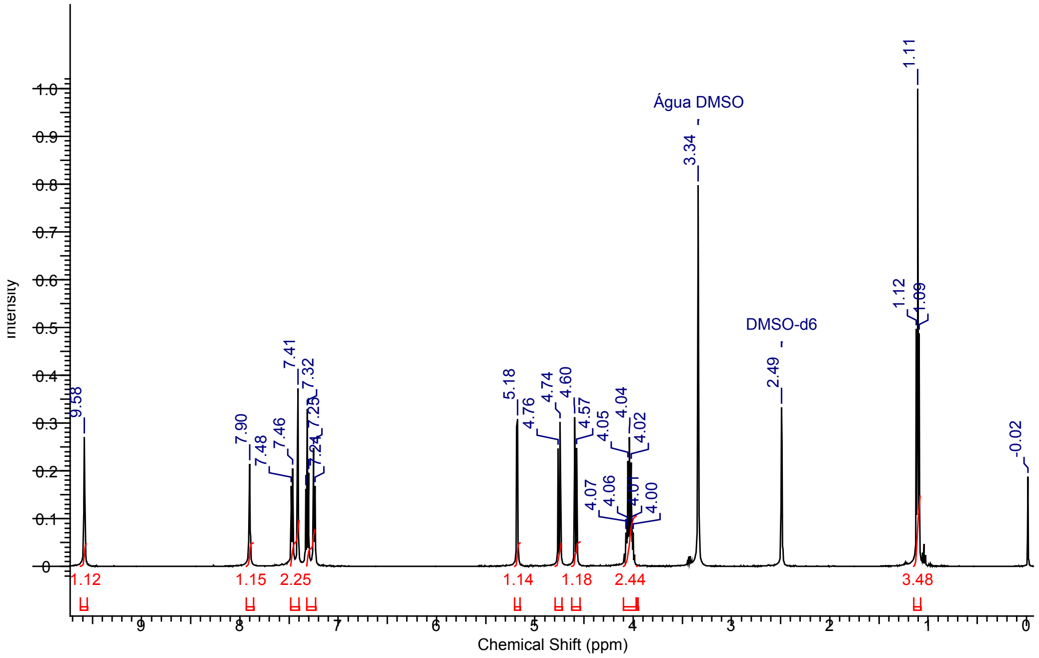

^{13}C NMR

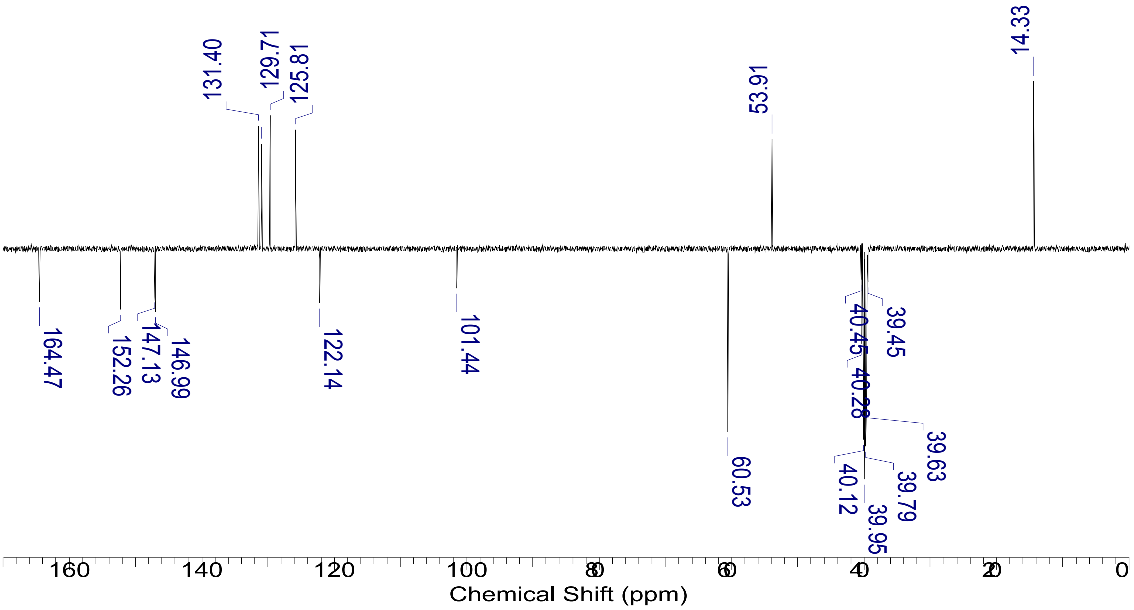

FT-IR

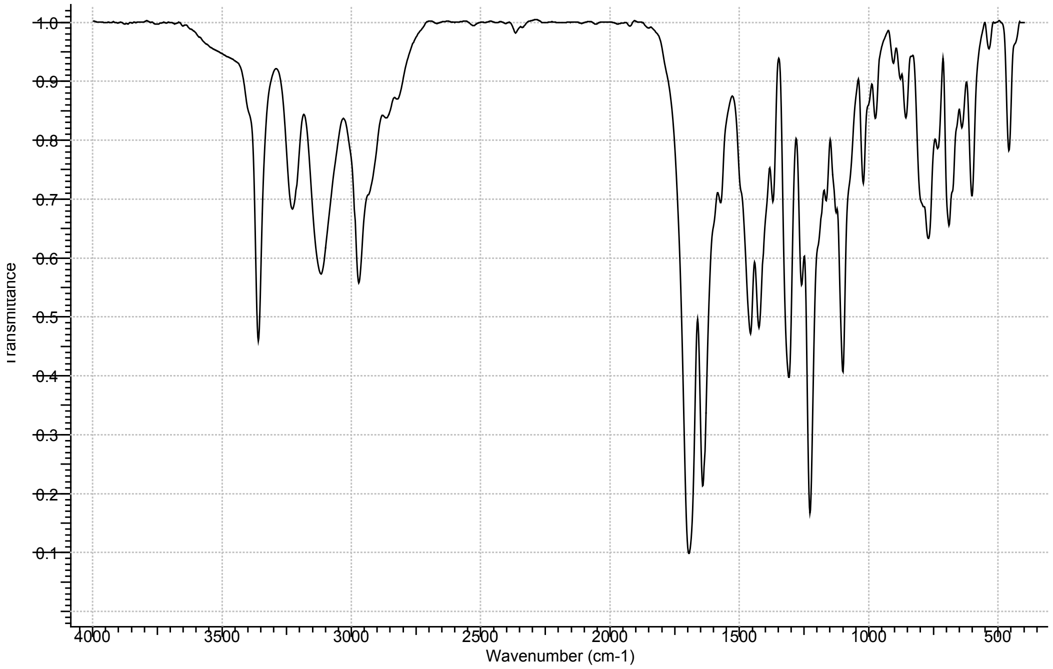

Mass Spectrometry

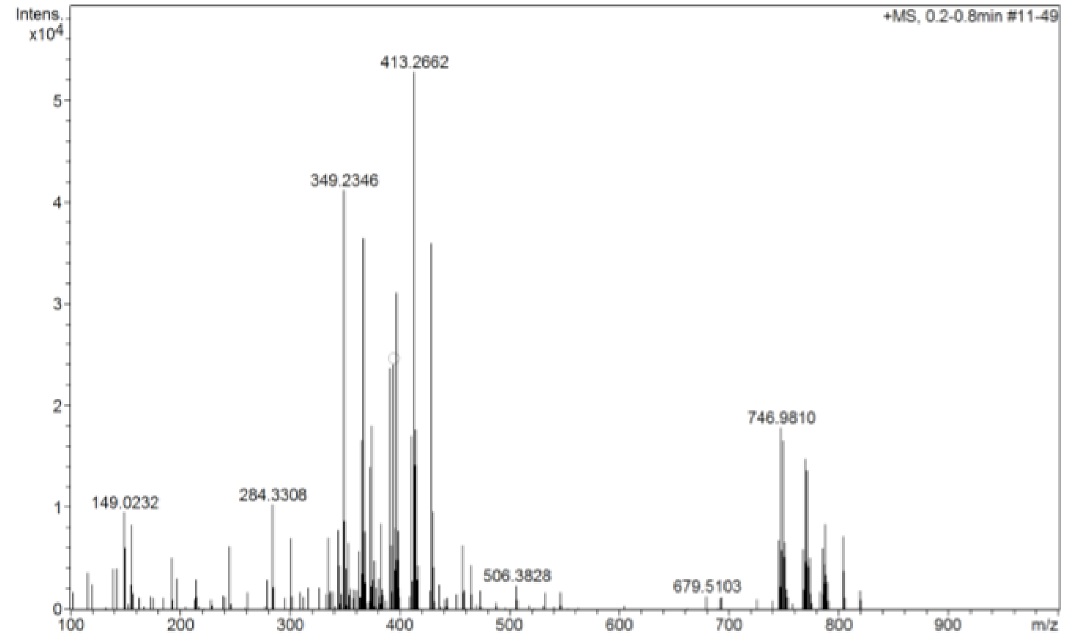

Meas. m/z	#	Ion Formula	m/z	err [ppm]	Mean err [ppm]	rdb	N-Rule	e⁻ Conf	mSigma	Std I	Std Mean m/z	Std I VarNorm	Std m/z Diff	Std Comb Dev
394.976583	1	C14H14BrClN2NaO3	394.976853	0.7	1.1	7.5	ok	even	6.1	6.4	n.a.	n.a.	n.a.	n.a.
	2	C10H10BrClN8NaO	394.974167	-6.1	-6.8	8.5	ok	even	11.1	13.8	n.a.	n.a.	n.a.	n.a.
	3	C9H14BrClN4NaO5	394.972830	-9.5	-9.6	3.5	ok	even	23.4	29.9	n.a.	n.a.	n.a.	n.a.
	4	C13H23Br2ClNa	394.974723	-4.7	3015.0	0.5	ok	even	207.3	242.9	n.a.	n.a.	n.a.	n.a.

Compound 1d

Chromatogram

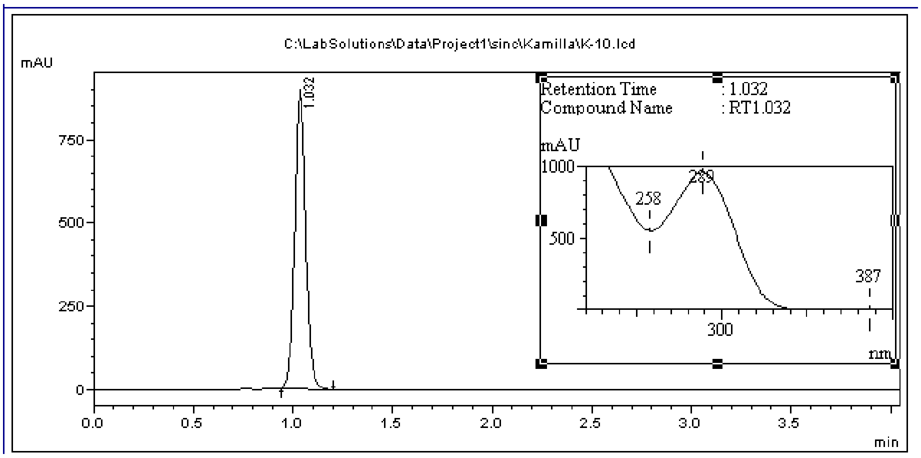

^{1}H NMR

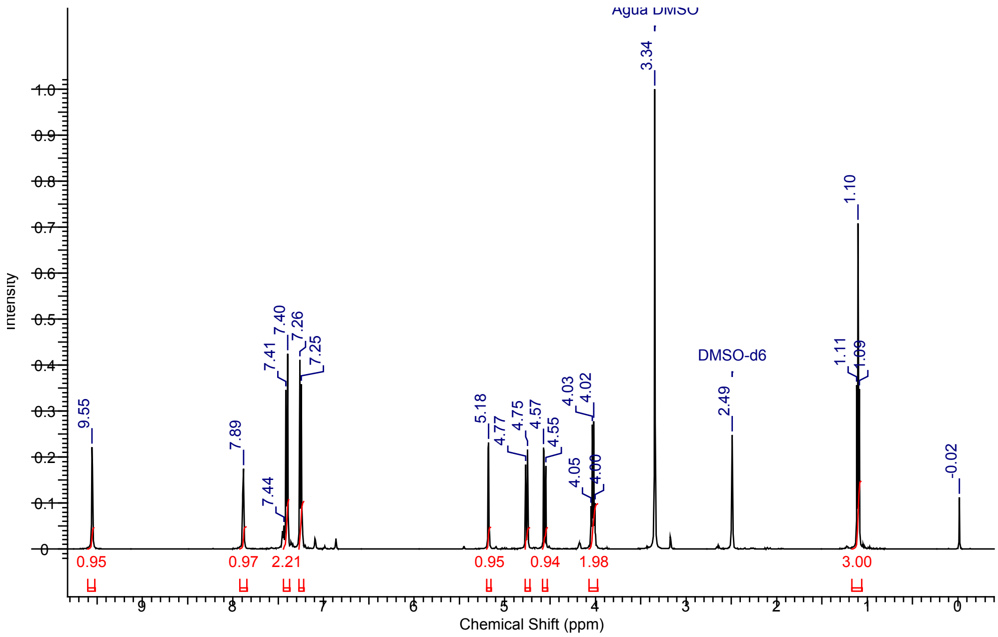
Agua DMSO
3.34
1.10
DMSO-d6
2.49
9.55
7.89
7.44
7.41
7.40
7.26
7.25
5.18
4.77
4.75
4.57
4.55
4.05
4.03
4.02
4.00
1.11
1.09
-0.02
0.95
0.97
2.21
0.95
0.94
1.98
3.00
Intensity
Chemical Shift (ppm)

^{13}C NMR

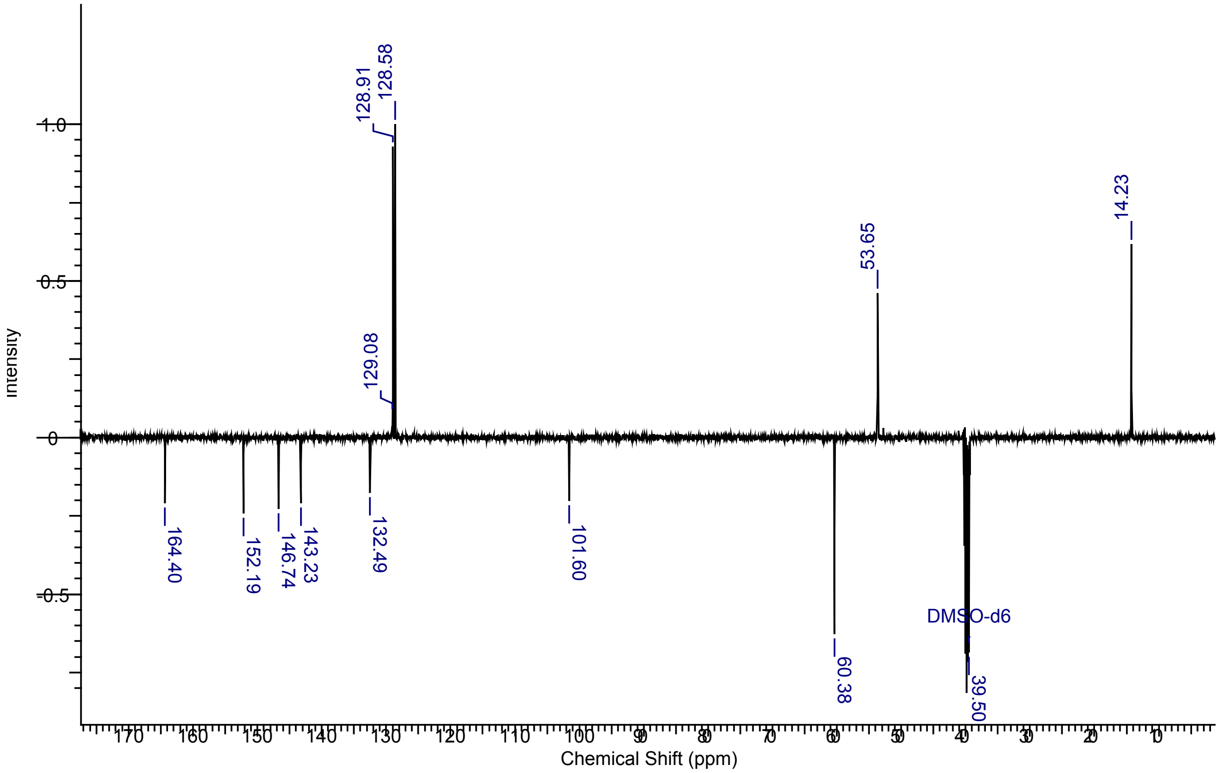
128.91
128.58
129.08
53.65
14.23
164.40
152.19
146.74
143.23
132.49
101.60
60.38
DMSO-d6
39.50
Intensity
Chemical Shift (ppm)

FT-IR

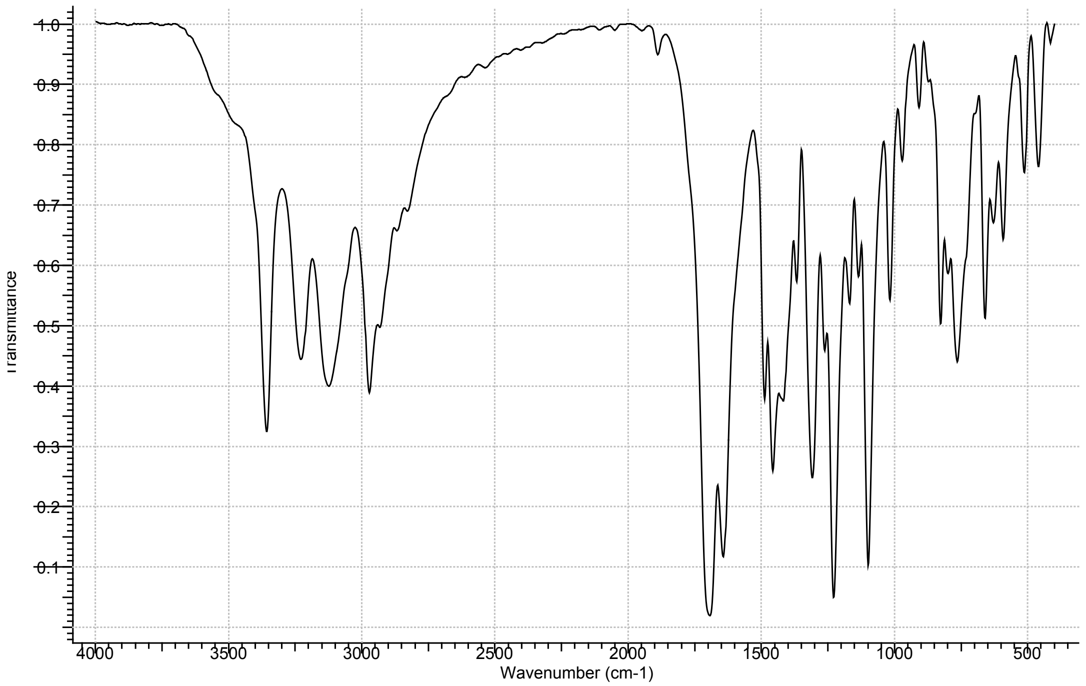

Mass Spectrometry

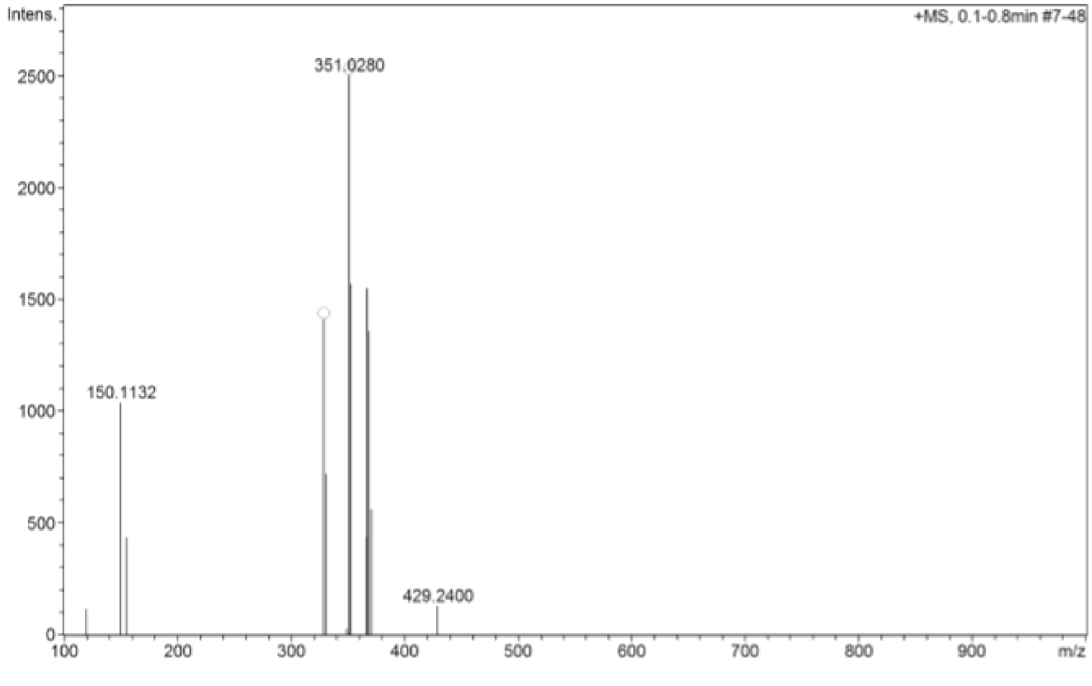

Meas. m/z	#	Ion Formula	m/z	err [ppm]	Mean err [ppm]	rdb	N-Rule	e⁻ Conf	mSigma	Std I	Std Mean m/z	Std I VarNorm	Std m/z Diff	Std Comb Dev
329.045832	1	C10H11Cl2N8O	329.042739	-9.4	4707.0	8.5	ok	even	99.3	191.1	n.a.	n.a.	n.a.	n.a.
	2	C14H15Cl2N2O3	329.045424	-1.2	4715.1	7.5	ok	even	109.7	207.6	n.a.	n.a.	n.a.	n.a.
	3	C19H15Cl2O	329.049447	11.0	4731.0	11.5	ok	even	128.0	235.4	n.a.	n.a.	n.a.	n.a.
351.028022	1	C4H10Cl2N12NaO2	351.031894	11.0	3154.2	4.5	ok	even	68.1	127.1	n.a.	n.a.	n.a.	n.a.
	2	C10H10Cl2N8NaO	351.024683	-9.5	4411.7	8.5	ok	even	81.7	163.8	n.a.	n.a.	n.a.	n.a.
	3	C14H14Cl2N2NaO3	351.027368	-1.9	4419.2	7.5	ok	even	92.6	180.7	n.a.	n.a.	n.a.	n.a.
	4	C19H14Cl2NaO	351.031391	9.6	4434.2	11.5	ok	even	112.9	210.1	n.a.	n.a.	n.a.	n.a.

Compound 1e

Chromatogram

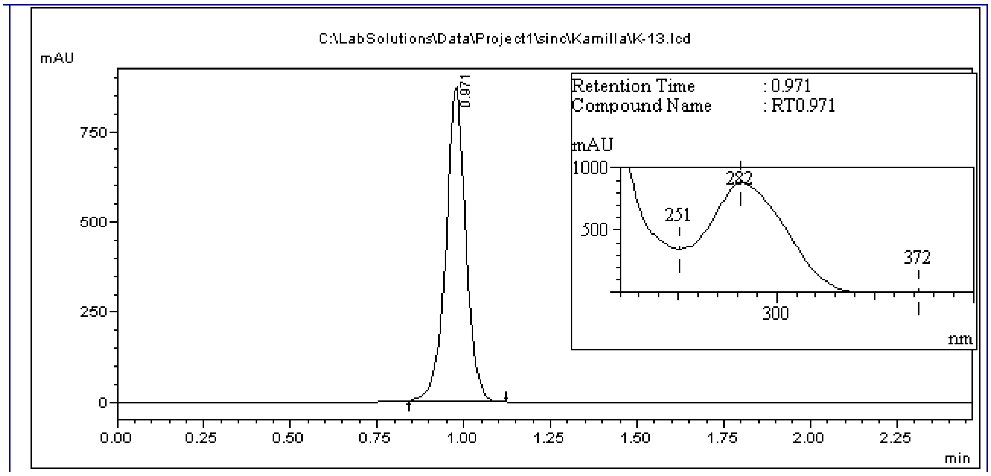

^{1}H NMR

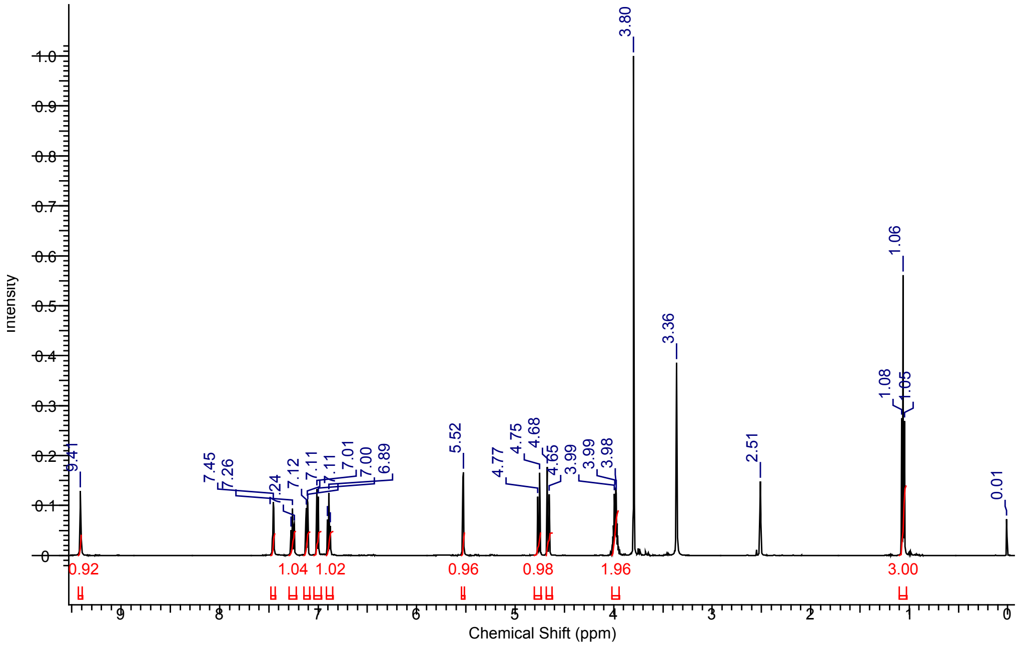

^{13}C NMR

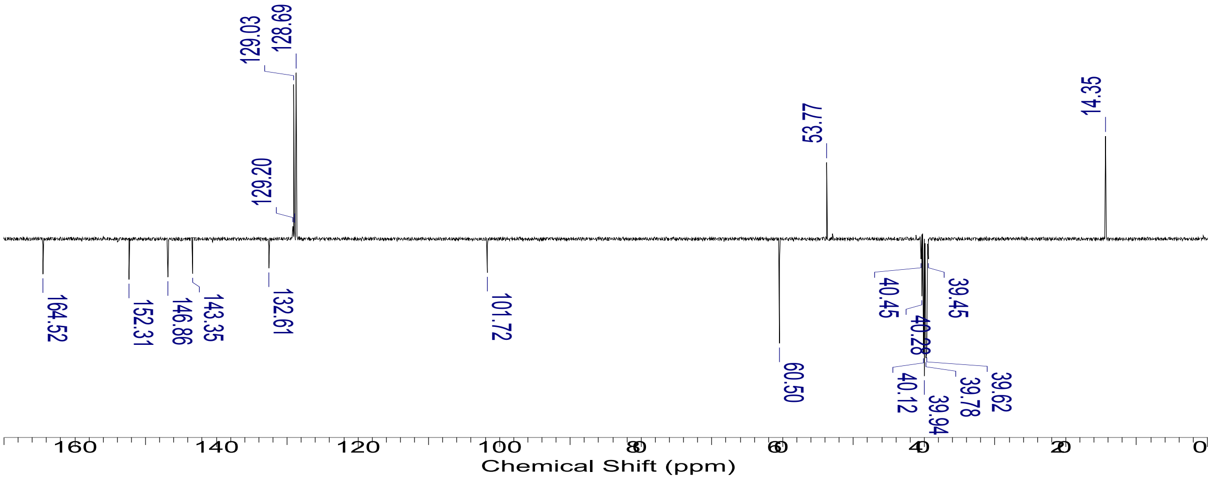

FT-IR

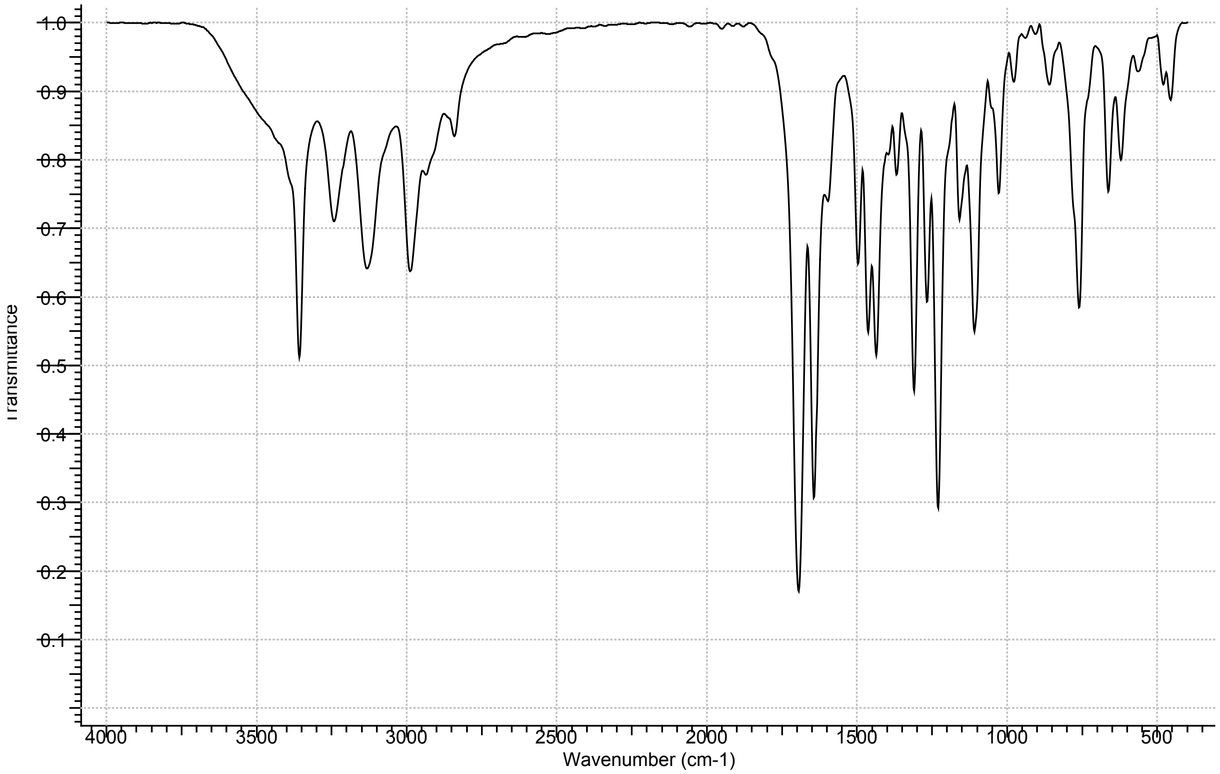

Mass Spectrometry

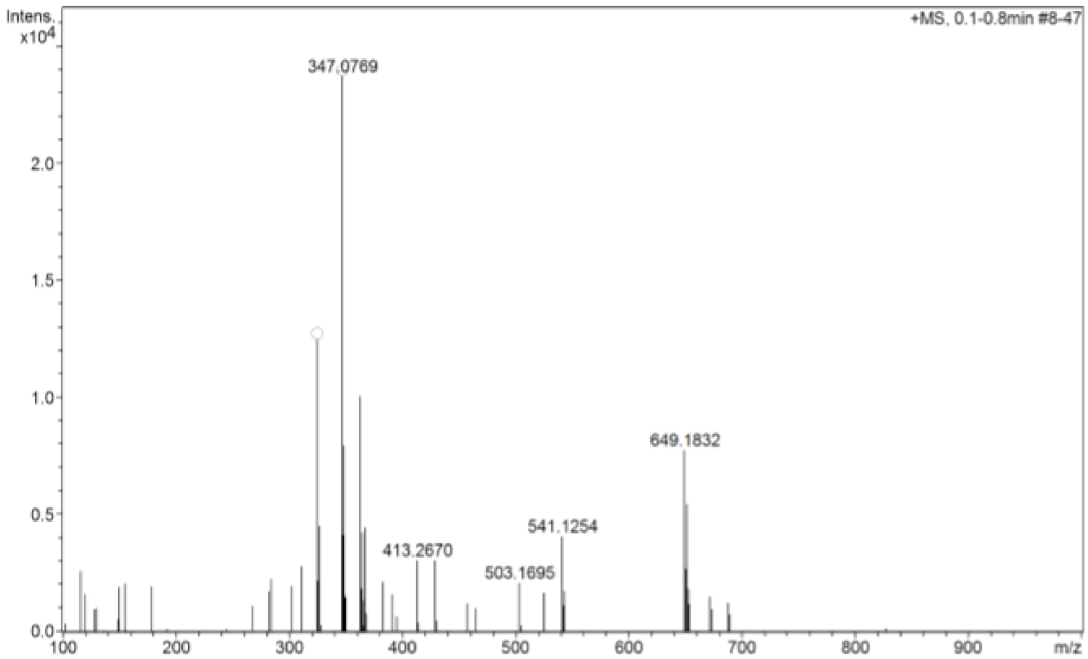

Meas. m/z	#	Ion Formula	m/z	err [ppm]	Mean err [ppm]	rdb	N-Rule	e⁻ Conf	mSigma	Std I	Std Mean m/z	Std I VarNorm	Std m/z Diff	Std Comb Dev
325.095071	1	C15H18ClN2O4	325.094961	-0.3	0.9	7.5	ok	even	20.6	28.3	n.a.	n.a.	n.a.	n.a.
	2	C11H14ClN8O2	325.092276	-8.6	-8.7	8.5	ok	even	22.4	28.2	n.a.	n.a.	n.a.	n.a.
	3	C16H14ClN6	325.096299	3.8	4.3	12.5	ok	even	27.0	39.6	n.a.	n.a.	n.a.	n.a.
	4	C20H18ClO2	325.098984	12.0	1675.2	11.5	ok	even	31.8	56.5	n.a.	n.a.	n.a.	n.a.
347.076945	1	C15H17ClN2NaO4	347.076905	-0.1	-0.3	7.5	ok	even	3.8	4.7	n.a.	n.a.	n.a.	n.a.
	2	C16H13ClN6Na	347.078243	3.7	3.0	12.5	ok	even	11.7	17.1	n.a.	n.a.	n.a.	n.a.
	3	C11H13ClN8NaO2	347.074220	-7.9	-9.2	8.5	ok	even	12.9	20.9	n.a.	n.a.	n.a.	n.a.
	4	C20H17ClNaO2	347.080928	11.5	1568.2	11.5	ok	even	22.0	38.3	n.a.	n.a.	n.a.	n.a.

Compound 1f

Chromatogram

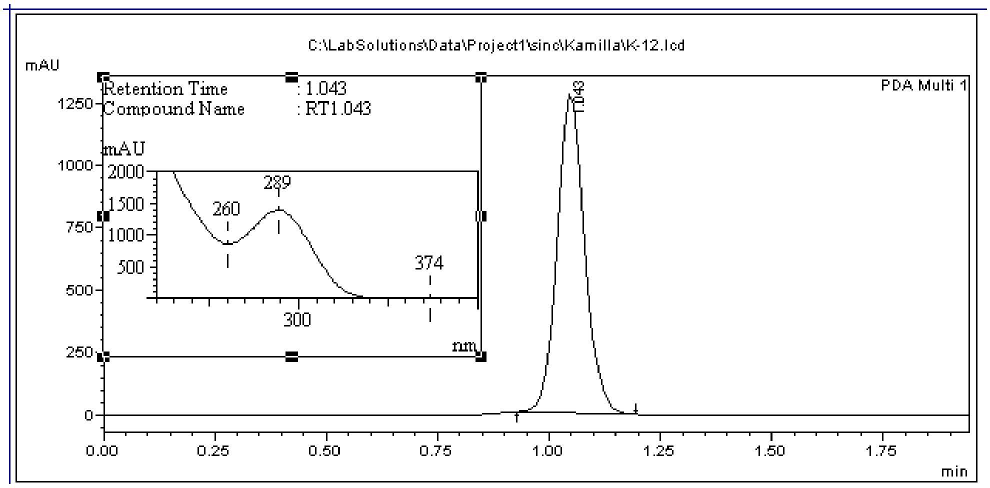

^{1}H NMR

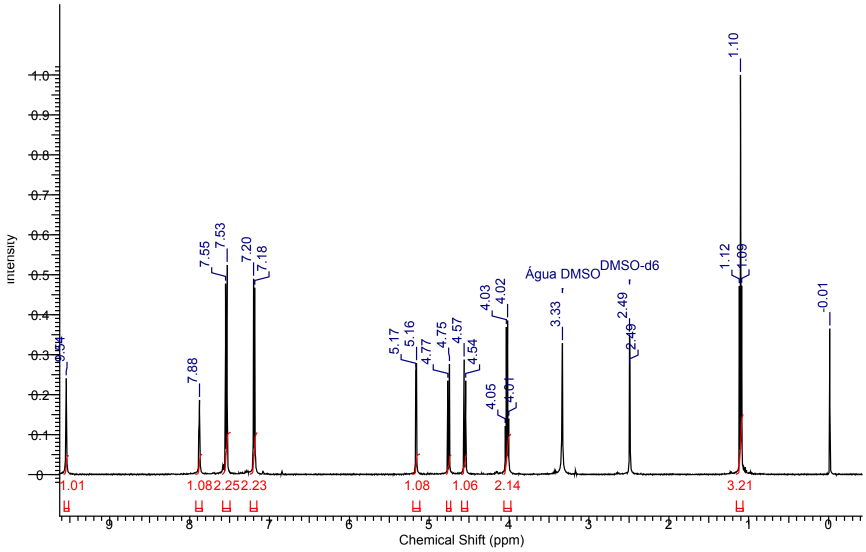
Intensity
7.88
7.55
7.53
7.20
7.18
5.17
5.16
4.77
4.75
4.57
4.54
4.05
4.03
4.02
4.01
3.33
Água DMSO
DMSO-d6
2.49
2.49
1.12
1.10
-0.01
1.01
1.08
2.25
2.23
1.08
1.06
2.14
3.21
Chemical Shift (ppm)

^{13}C NMR

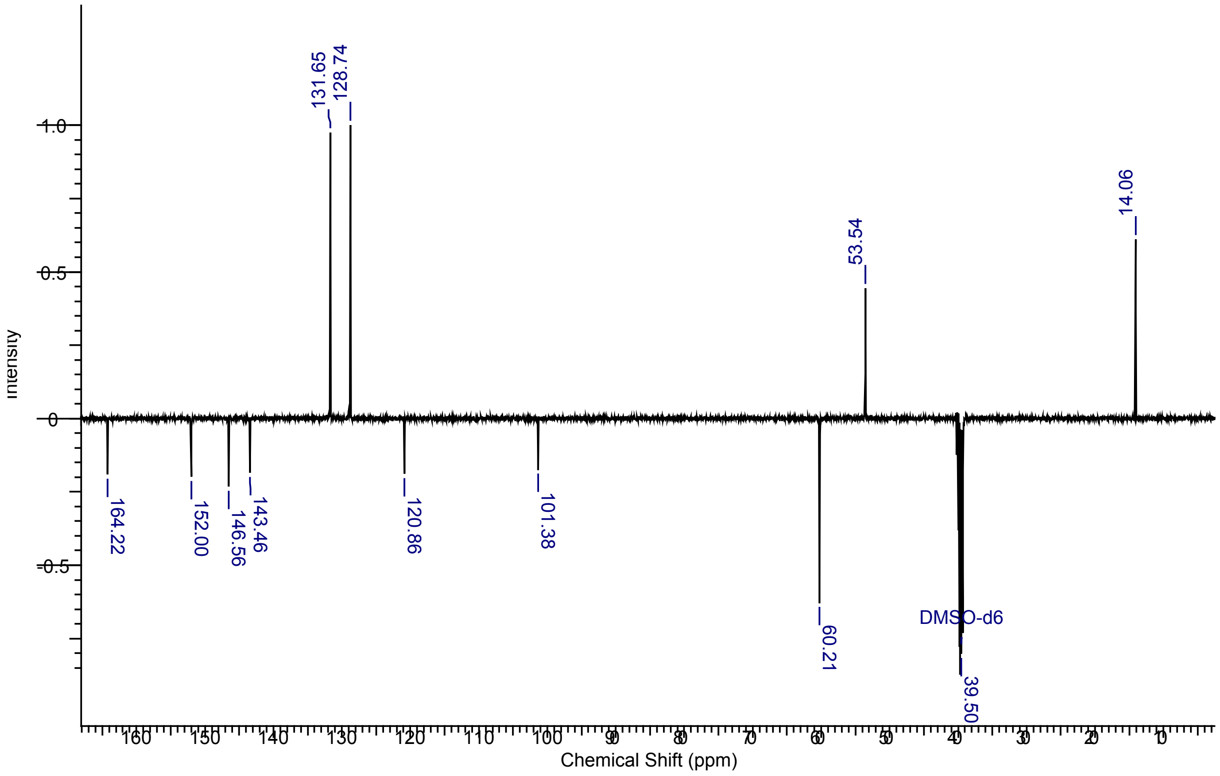
Intensity
131.65
128.74
53.54
14.06
164.22
152.00
146.56
143.46
120.86
101.38
60.21
DMSO-d6
39.50
Chemical Shift (ppm)

FT-IR

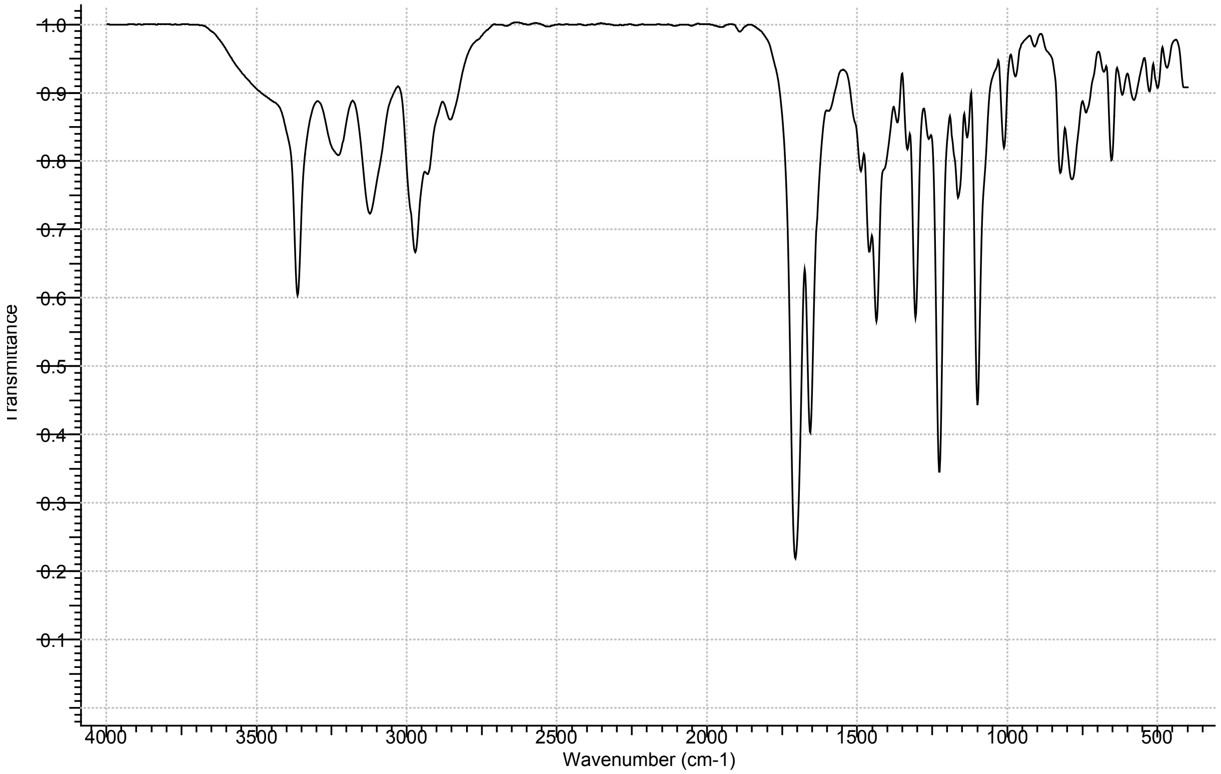

Mass Spectrometry

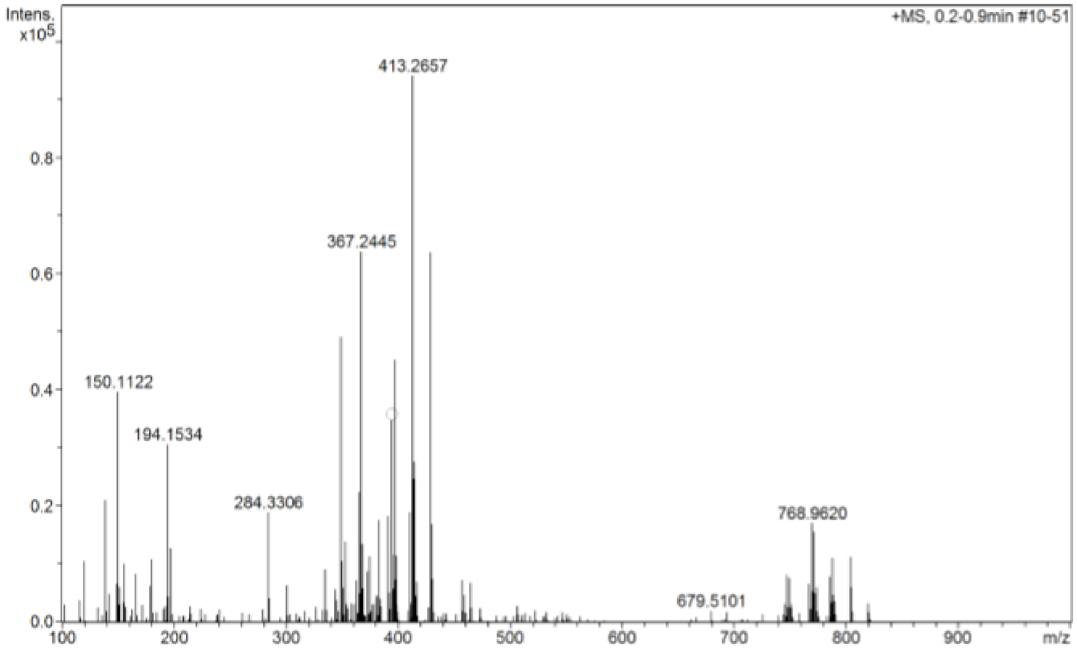

Meas. m/z	#	Ion Formula	m/z	err [ppm]	Mean err [ppm]	rdb	N-Rule	e⁻ Conf	mSigma	Std I	Std Mean m/z	Std I VarNorm	Std m/z Diff	Std Comb Dev
394.975983	1	C14H14BrClN2NaO3	394.976853	2.2	2.0	7.5	ok	even	2.5	2.3	n.a.	n.a.	n.a.	n.a.
	2	C10H10BrClN8NaO	394.974167	-4.6	-5.9	8.5	ok	even	12.1	15.9	n.a.	n.a.	n.a.	n.a.
	3	C9H14BrClN4NaO5	394.972830	-8.0	-8.7	3.5	ok	even	24.3	31.9	n.a.	n.a.	n.a.	n.a.
	4	C13H23Br2ClNa	394.974723	-3.2	3015.6	0.5	ok	even	204.4	239.0	n.a.	n.a.	n.a.	n.a.

Compound 1g

Chromatogram

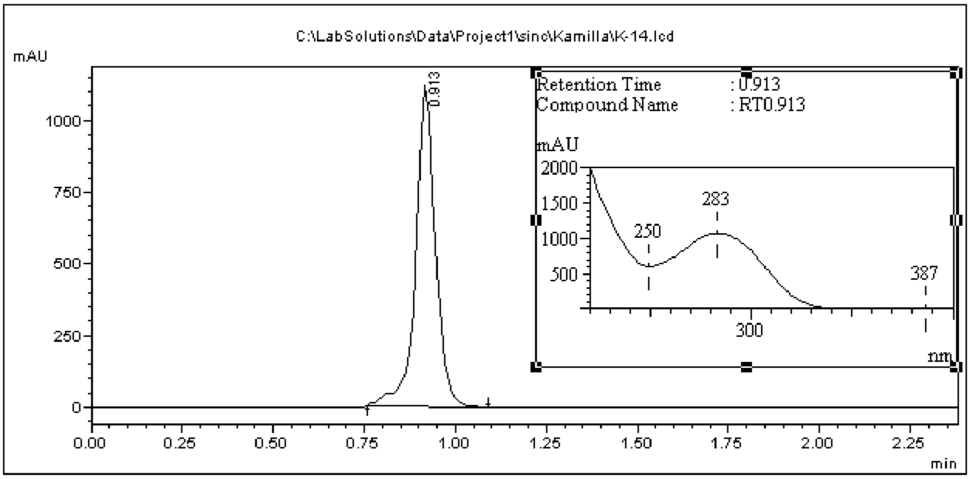

^{1}H NMR

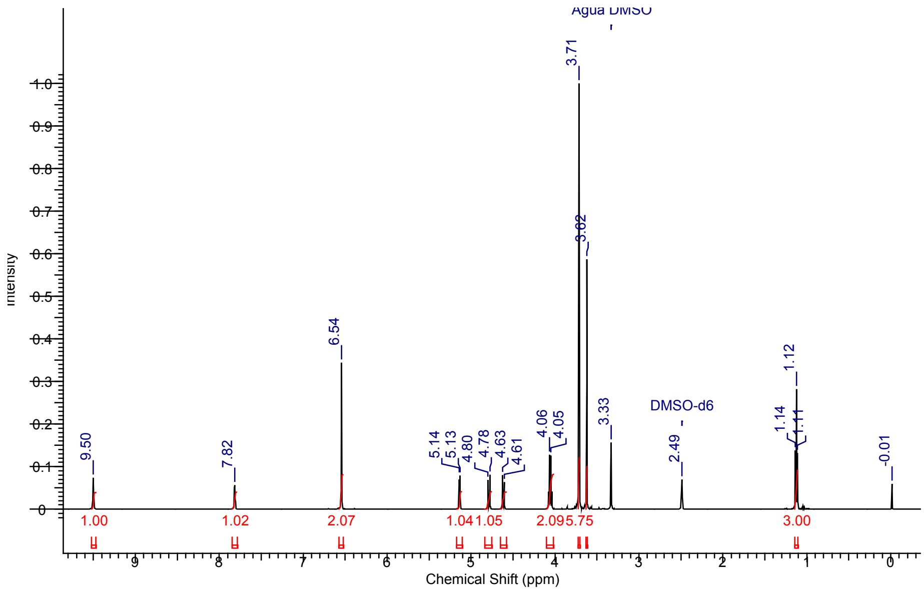

^{13}C NMR

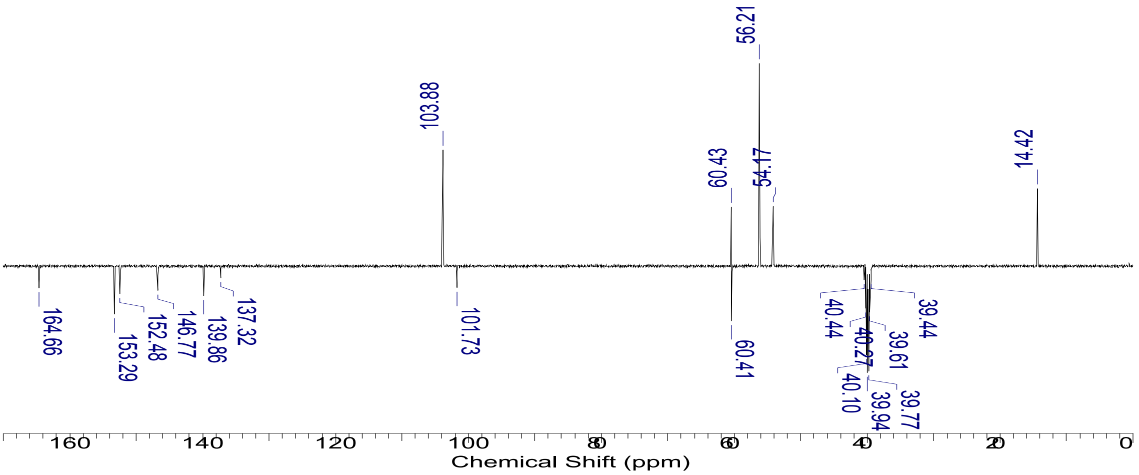

FT-IR

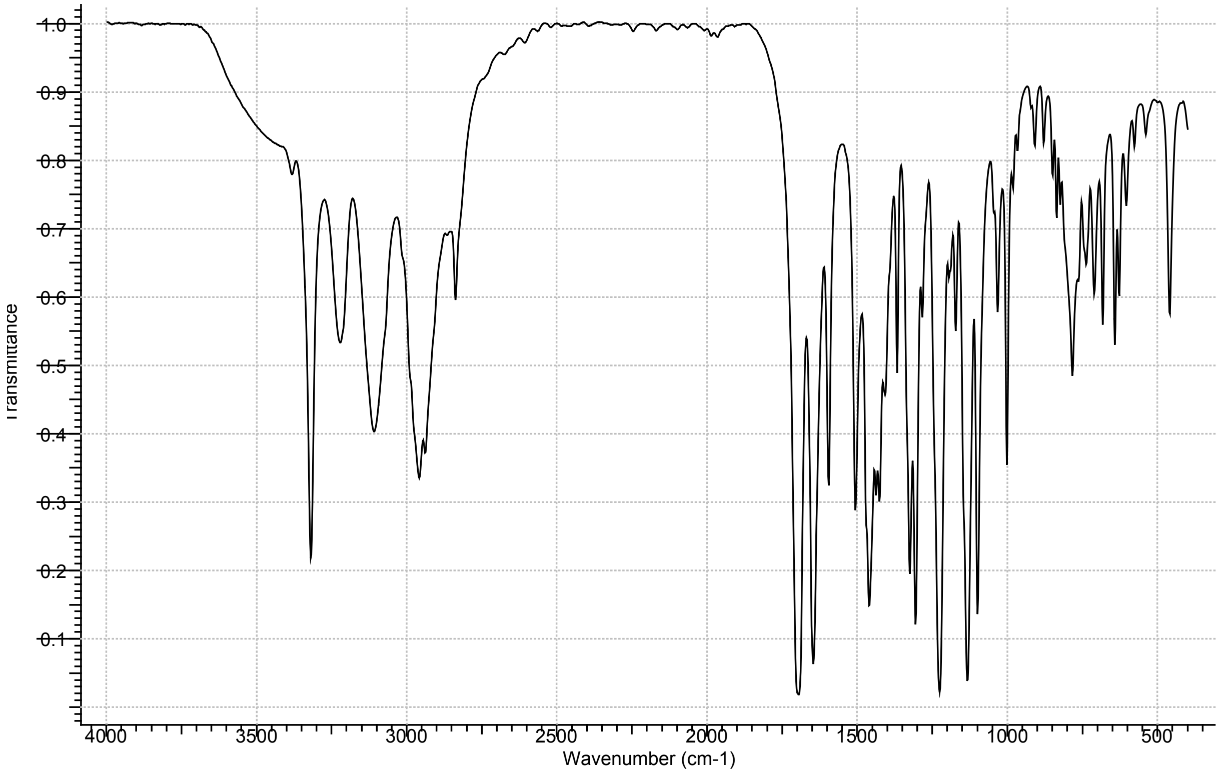

Mass Spectrometry

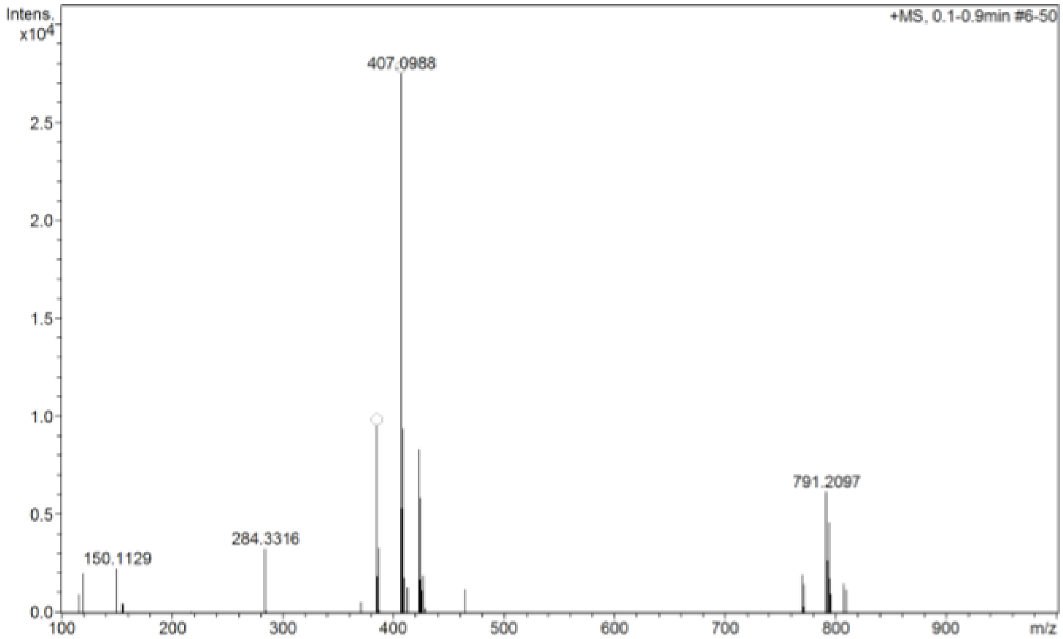

Meas. m/z	#	Ion Formula	m/z	err [ppm]	Mean err [ppm]	rdb	N-Rule	e⁻ Conf	mSigma	Std I	Std Mean m/z	Std I VarNorm	Std m/z Diff	Std Comb Dev
385.116845	1	C17H22ClN2O6	385.116091	-2.0	1410.7	7.5	ok	even	25.2	45.0	n.a.	n.a.	n.a.	n.a.
	2	C13H18ClN8O4	385.113405	-8.9	-8.9	8.5	ok	even	26.0	35.0	n.a.	n.a.	n.a.	n.a.
	3	C14H14ClN12	385.114743	-5.5	-5.9	13.5	ok	even	26.8	39.6	n.a.	n.a.	n.a.	n.a.
	4	C18H18ClN6O2	385.117428	1.5	1405.9	12.5	ok	even	29.7	54.6	n.a.	n.a.	n.a.	n.a.
	5	C22H22ClO4	385.120113	8.5	1406.9	11.5	ok	even	38.3	70.5	n.a.	n.a.	n.a.	n.a.
407.098822	1	C14H13ClN12Na	407.096687	-5.2	-6.6	13.5	ok	even	2.4	3.0	n.a.	n.a.	n.a.	n.a.
	2	C17H21ClN2NaO6	407.098035	-1.9	1333.7	7.5	ok	even	6.2	11.2	n.a.	n.a.	n.a.	n.a.
	3	C13H17ClN8NaO4	407.095349	-8.5	-9.4	8.5	ok	even	10.5	15.7	n.a.	n.a.	n.a.	n.a.
	4	C18H17ClN6NaO2	407.099372	1.4	1329.1	12.5	ok	even	13.1	23.3	n.a.	n.a.	n.a.	n.a.
	5	C22H21ClNaO4	407.102058	7.9	1330.1	11.5	ok	even	24.6	42.2	n.a.	n.a.	n.a.	n.a.

Compound 1h

Chromatogram

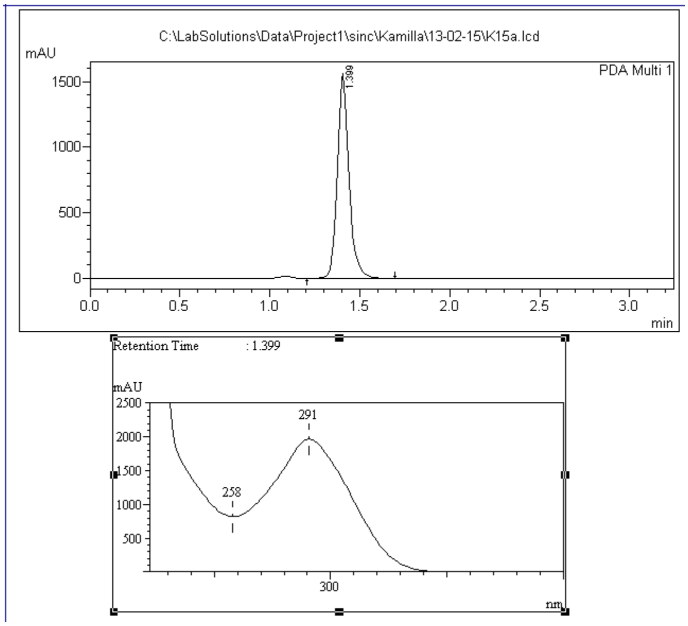

^{1}H NMR

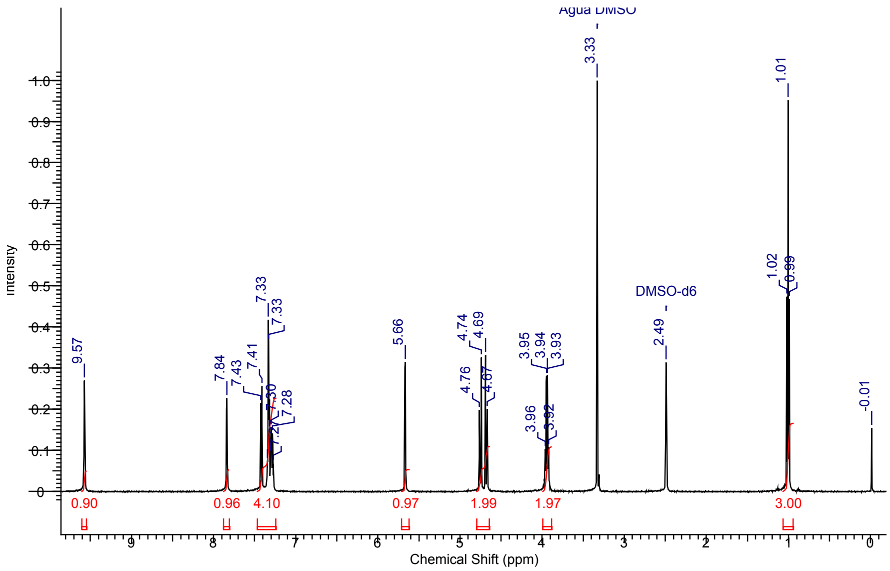
Agua DMSO
3.33
1.01
DMSO-d6
2.49
9.57
7.84
7.43
7.41
7.33
7.33
7.28
5.66
4.74
4.69
4.76
3.95
3.94
3.93
3.96
3.92
1.02
0.99
-0.01
0.90
0.96
4.10
0.97
1.99
1.97
3.00
Intensity
Chemical Shift (ppm)

^{13}C NMR

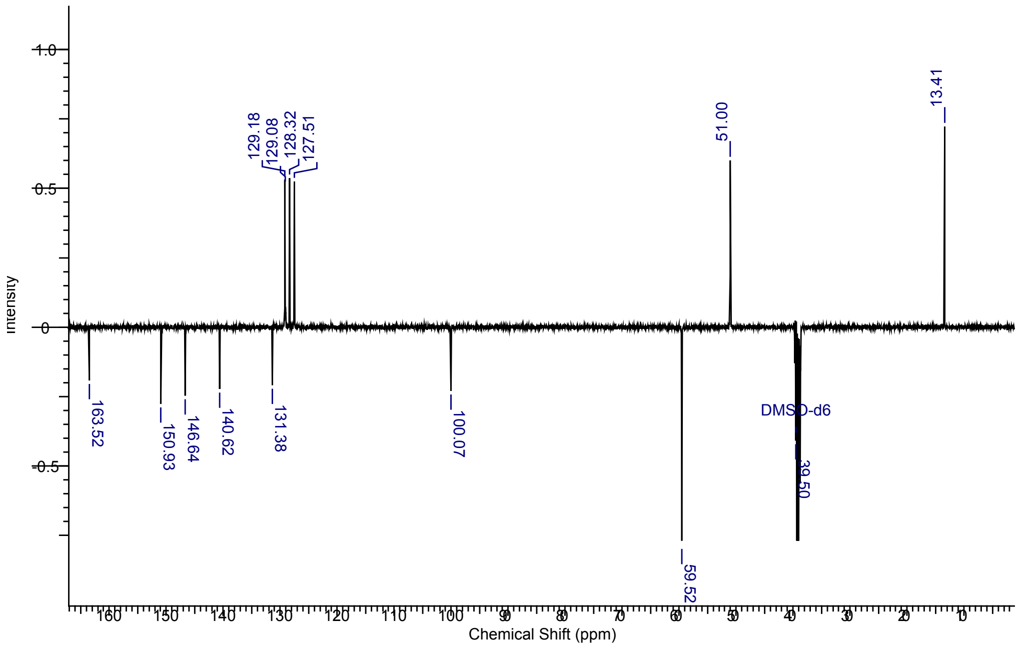
129.18
129.08
128.32
127.51
51.00
13.41
163.52
150.93
146.64
140.62
131.38
100.07
DMSO-d6
39.50
59.52
Intensity
Chemical Shift (ppm)

FT-IR

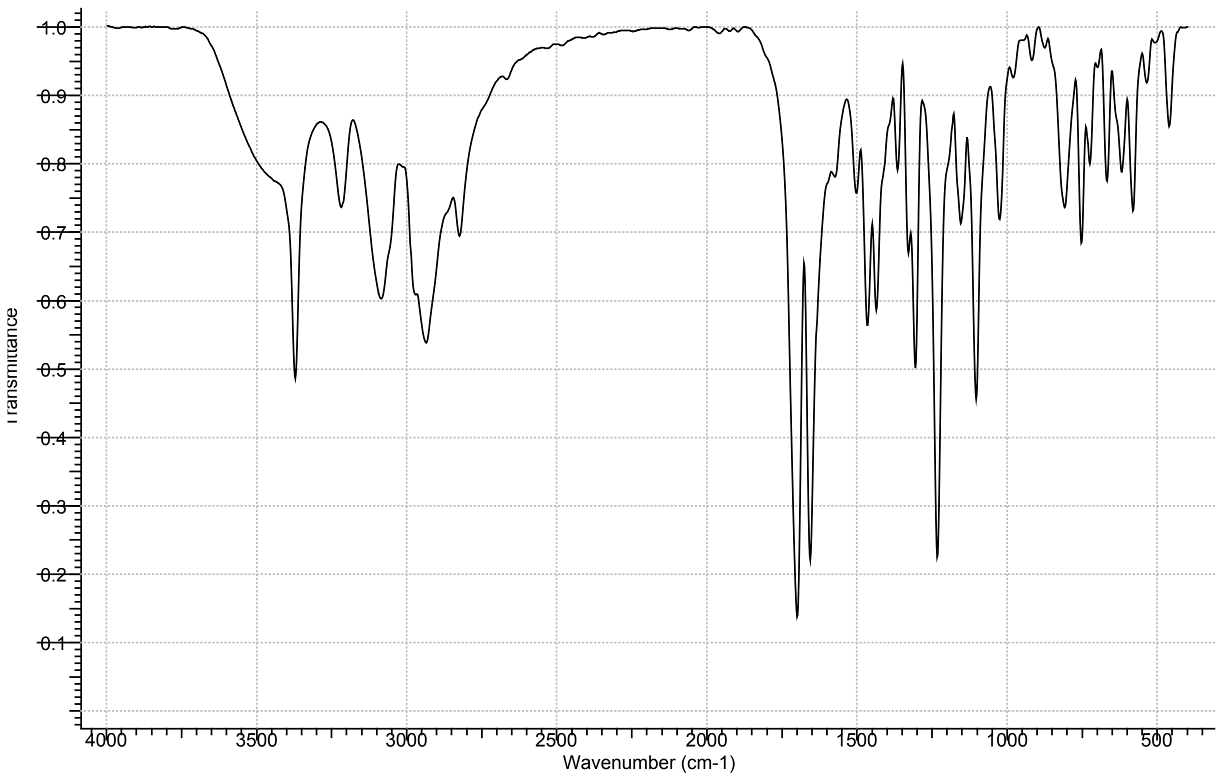

Mass Spectrometry

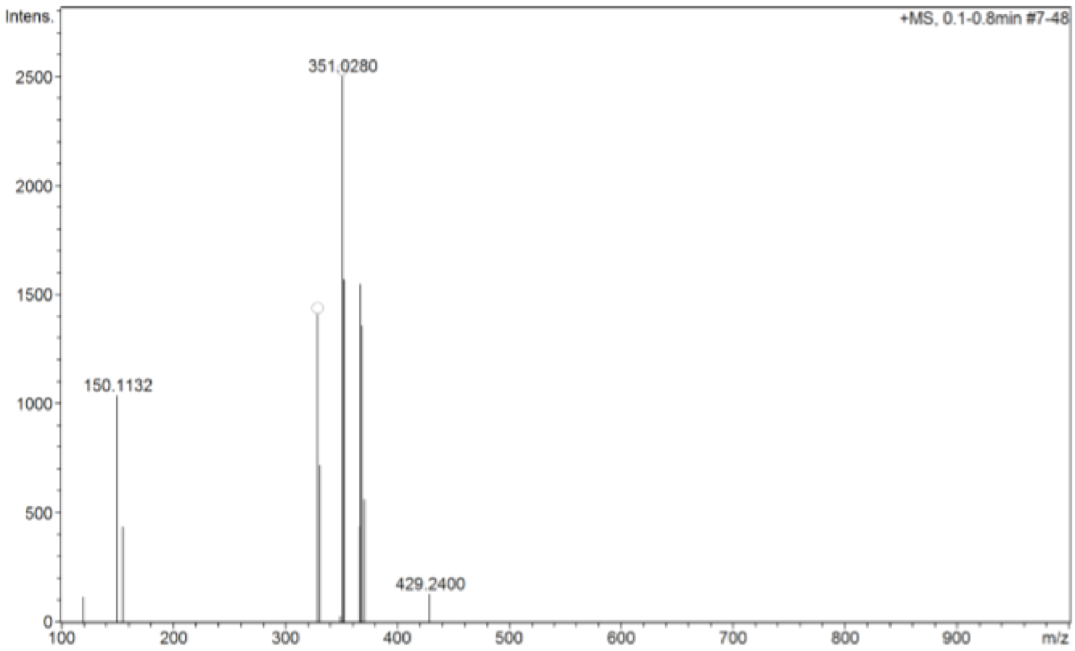

Meas. m/z	#	Ion Formula	m/z	err [ppm]	Mean err [ppm]	rdb	N-Rule	e⁻ Conf	mSigma	Std I	Std Mean m/z	Std I VarNorm	Std m/z Diff	Std Comb Dev
329.045832	1	C10H11Cl2N8O	329.042739	-9.4	4707.0	8.5	ok	even	99.3	191.1	n.a.	n.a.	n.a.	n.a.
	2	C14H15Cl2N2O3	329.045424	-1.2	4715.1	7.5	ok	even	109.7	207.6	n.a.	n.a.	n.a.	n.a.
	3	C19H15Cl2O	329.049447	11.0	4731.0	11.5	ok	even	128.0	235.4	n.a.	n.a.	n.a.	n.a.
351.028022	1	C4H10Cl2N12NaO2	351.031894	11.0	3154.2	4.5	ok	even	68.1	127.1	n.a.	n.a.	n.a.	n.a.
	2	C10H10Cl2N8NaO	351.024683	-9.5	4411.7	8.5	ok	even	81.7	163.8	n.a.	n.a.	n.a.	n.a.
	3	C14H14Cl2N2NaO3	351.027368	-1.9	4419.2	7.5	ok	even	92.6	180.7	n.a.	n.a.	n.a.	n.a.
	4	C19H14Cl2NaO	351.031391	9.6	4434.2	11.5	ok	even	112.9	210.1	n.a.	n.a.	n.a.	n.a.

Compound 1i

Chromatogram

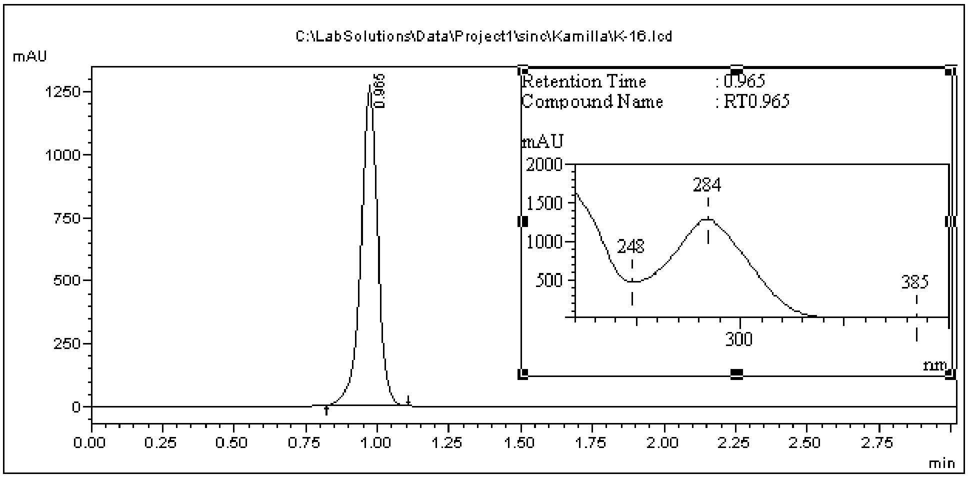

^{1}H NMR

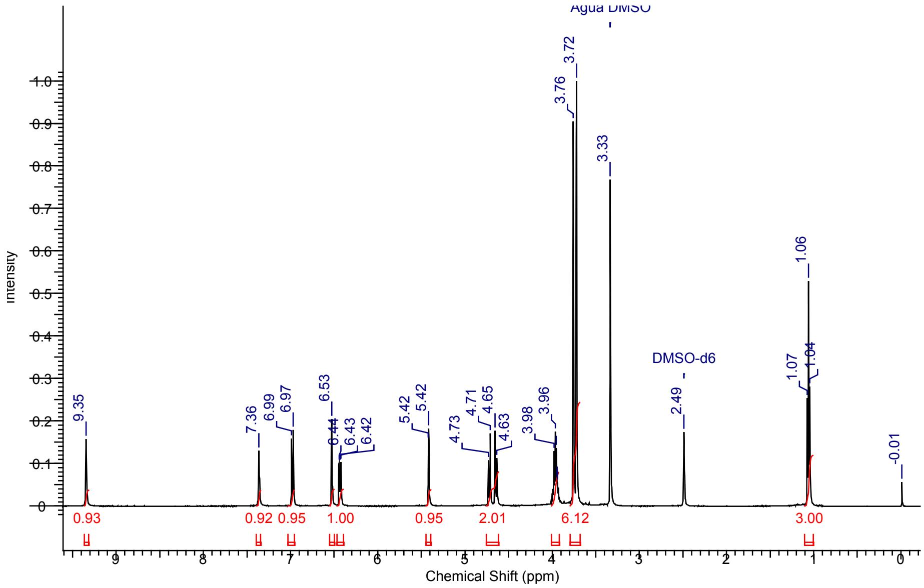

^{13}C NMR

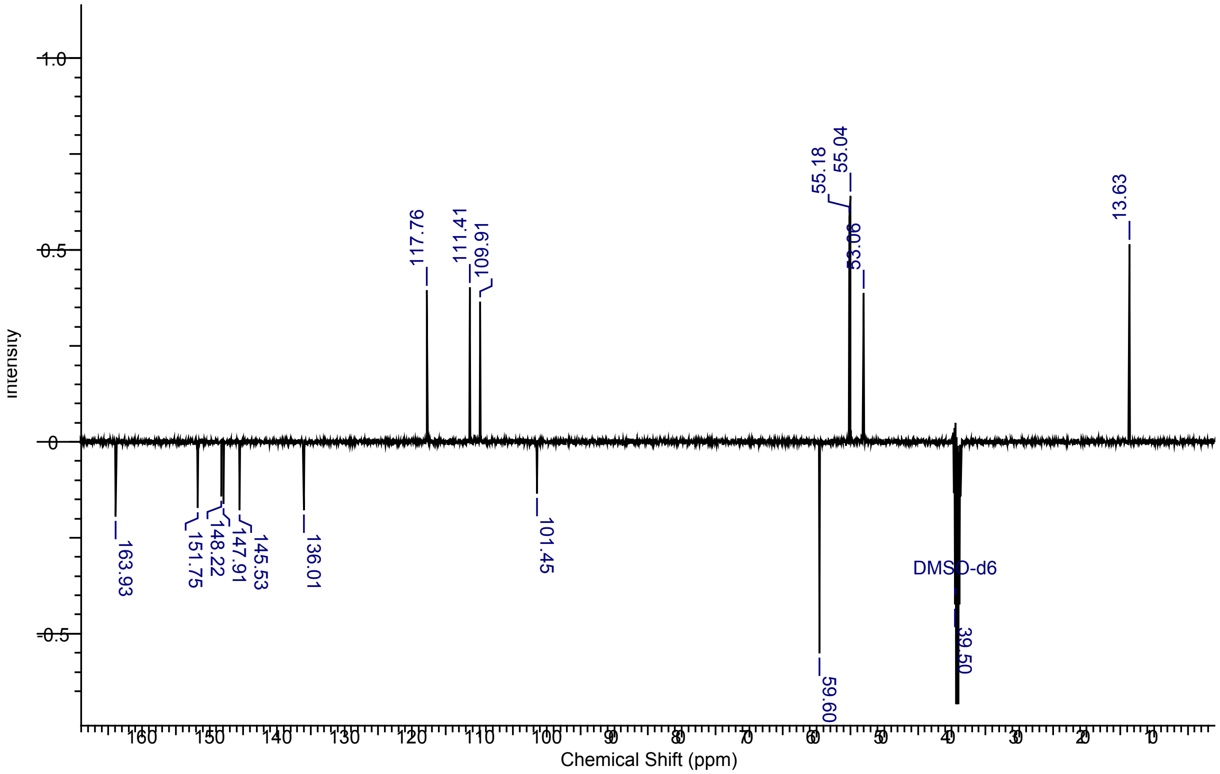

Mass Spectrometry

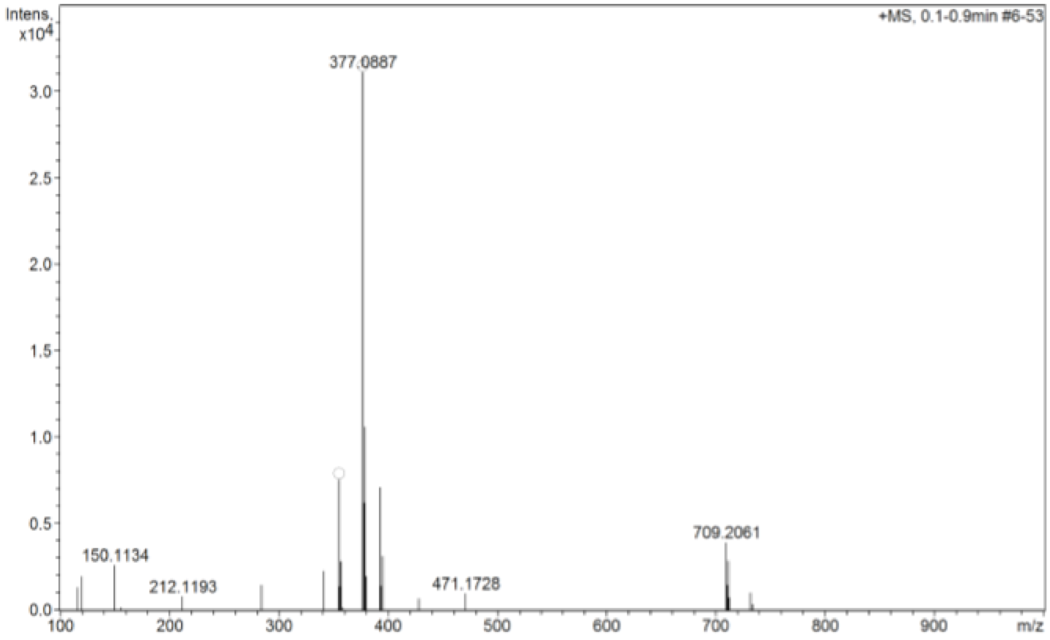

Meas. m/z	#	Ion Formula	m/z	err [ppm]	Mean err [ppm]	rdb	N-Rule	e⁻ Conf	mSigma	Std I	Std Mean m/z	Std I VarNorm	Std m/z Diff	Std Comb Dev
355.106672	1	C16H20ClN2O5	355.105526	-3.2	2745.8	7.5	ok	even	30.5	49.1	n.a.	n.a.	n.a.	n.a.
	2	C12H16ClN8O3	355.102841	-10.8	1384.5	8.5	ok	even	33.8	43.1	n.a.	n.a.	n.a.	n.a.
	3	C17H16ClN6O	355.106863	0.5	1408.2	12.5	ok	even	39.5	57.5	n.a.	n.a.	n.a.	n.a.
	4	C21H20ClO3	355.109549	8.1	2745.0	11.5	ok	even	41.8	72.9	n.a.	n.a.	n.a.	n.a.
377.088682	1	C17H15ClN6NaO	377.088808	0.3	-1.7	12.5	ok	even	4.9	7.5	n.a.	n.a.	n.a.	n.a.
	2	C16H19ClN2NaO5	377.087470	-3.2	1440.5	7.5	ok	even	8.1	11.3	n.a.	n.a.	n.a.	n.a.
	3	C21H19ClNaO3	377.091493	7.5	1436.6	11.5	ok	even	16.4	29.3	n.a.	n.a.	n.a.	n.a.
	4	C12H15ClN8NaO3	377.084785	-10.3	-12.9	8.5	ok	even	19.6	29.5	n.a.	n.a.	n.a.	n.a.

Compound 1j

Chromatogram

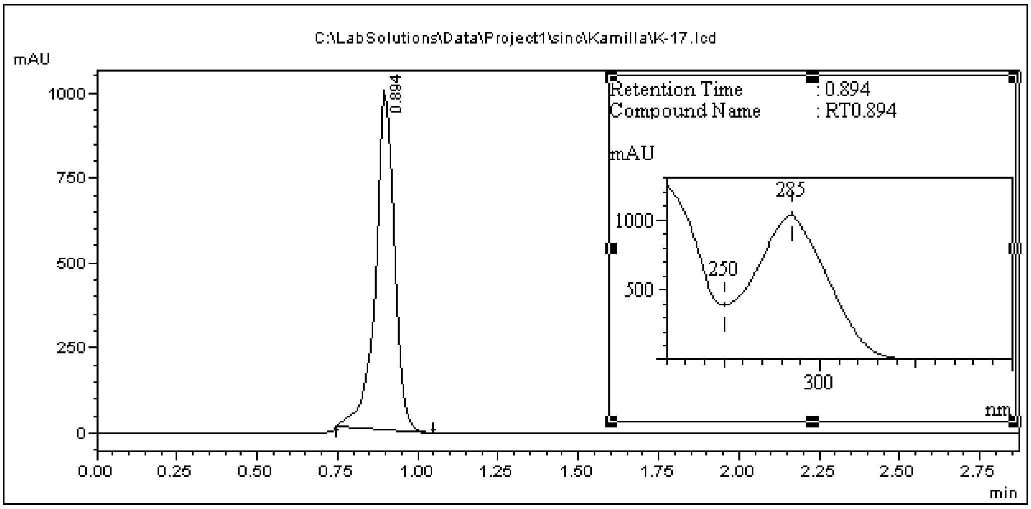

^{1}H NMR

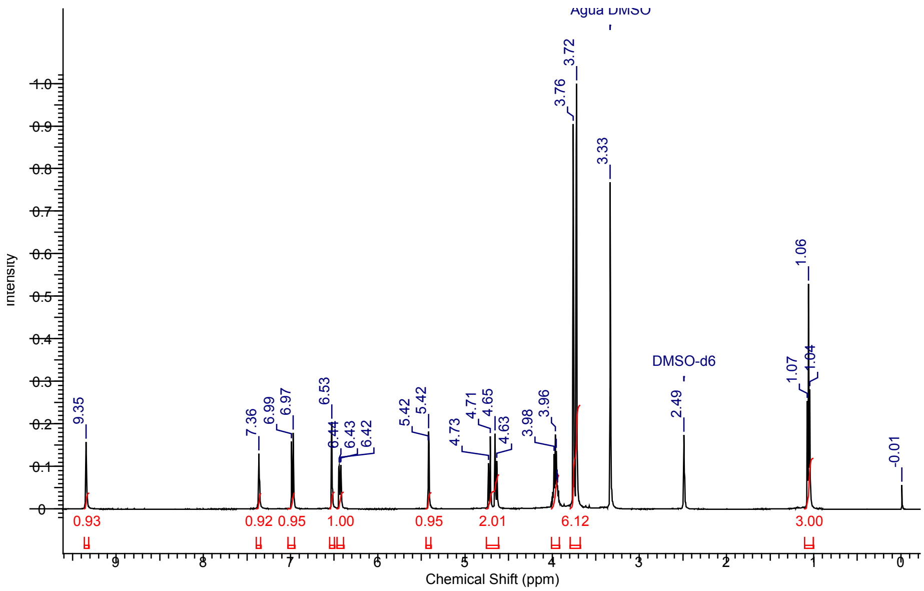

^{13}C NMR

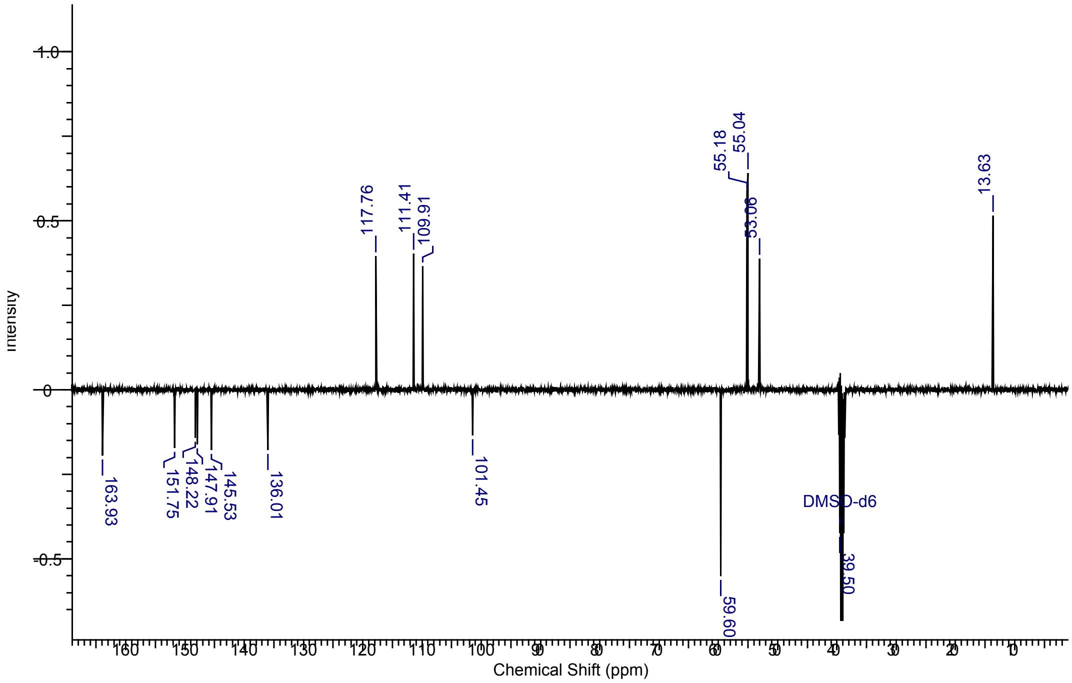

FT-IR

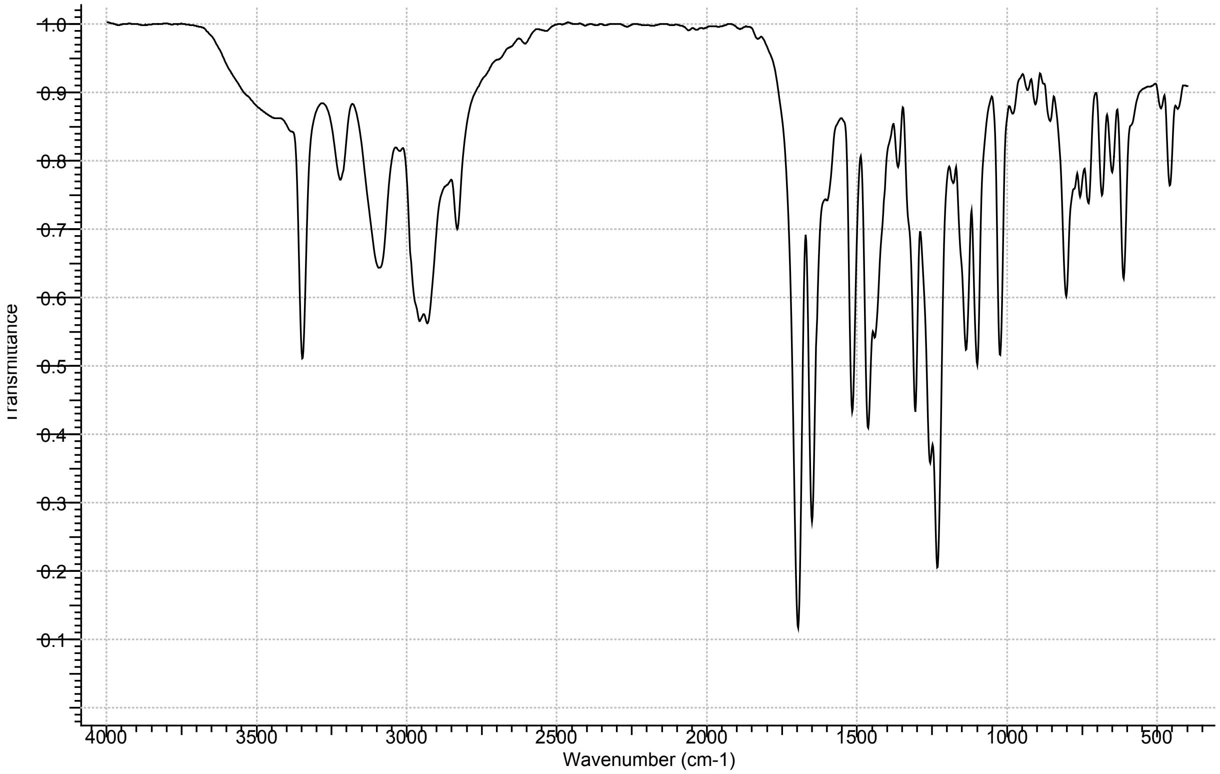

Mass Spectrometry

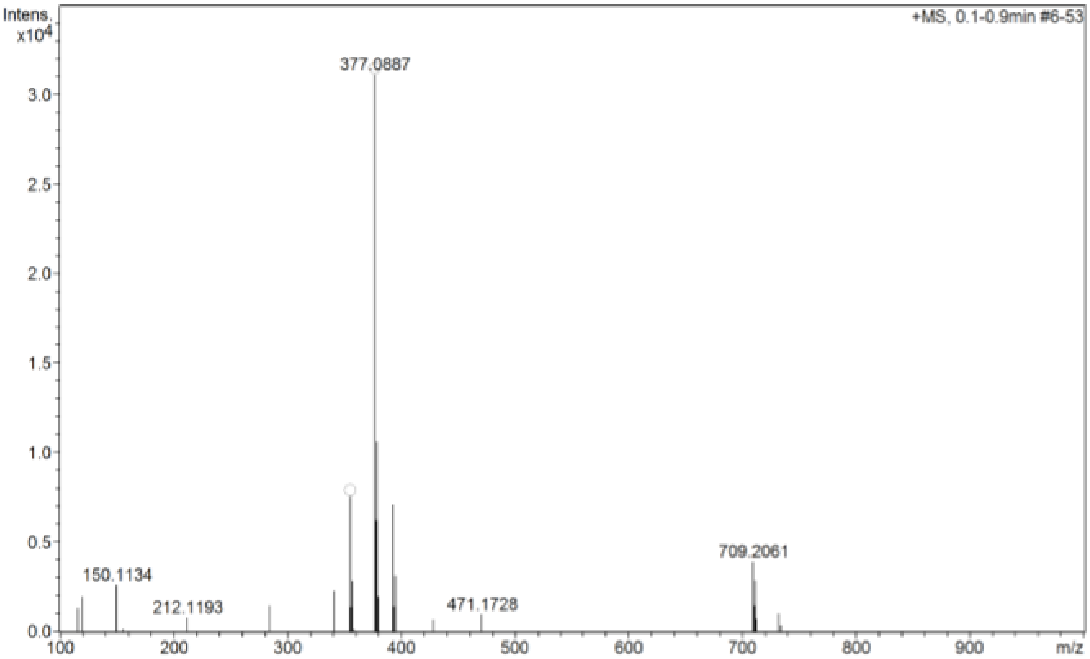

Meas. m/z	#	Ion Formula	m/z	err [ppm]	Mean err [ppm]	rdb	N-Rule	e⁻ Conf	mSigma	Std I	Std Mean m/z	Std I VarNorm	Std m/z Diff	Std Comb Dev
355.106672	1	C16H20ClN2O5	355.105526	-3.2	2745.8	7.5	ok	even	30.5	49.1	n.a.	n.a.	n.a.	n.a.
	2	C12H16ClN8O3	355.102841	-10.8	1394.5	8.5	ok	even	33.8	43.1	n.a.	n.a.	n.a.	n.a.
	3	C17H16ClN6O	355.106863	0.5	1408.2	12.5	ok	even	39.5	57.5	n.a.	n.a.	n.a.	n.a.
	4	C21H20ClO3	355.109549	8.1	2745.0	11.5	ok	even	41.8	72.9	n.a.	n.a.	n.a.	n.a.
377.088682	1	C17H15ClN6NaO	377.088808	0.3	-1.7	12.5	ok	even	4.9	7.5	n.a.	n.a.	n.a.	n.a.
	2	C16H19ClN2NaO5	377.087470	-3.2	1440.5	7.5	ok	even	8.1	11.3	n.a.	n.a.	n.a.	n.a.
	3	C21H19ClNaO3	377.091493	7.5	1436.6	11.5	ok	even	16.4	29.3	n.a.	n.a.	n.a.	n.a.
	4	C12H15ClN8NaO3	377.084785	-10.3	-12.9	8.5	ok	even	19.6	29.5	n.a.	n.a.	n.a.	n.a.

Compound 1k

Chromatogram

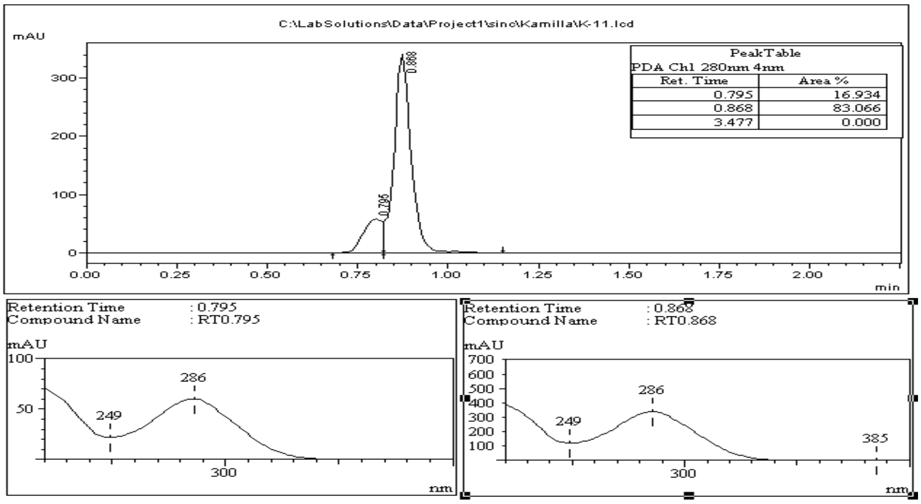

^{1}H NMR

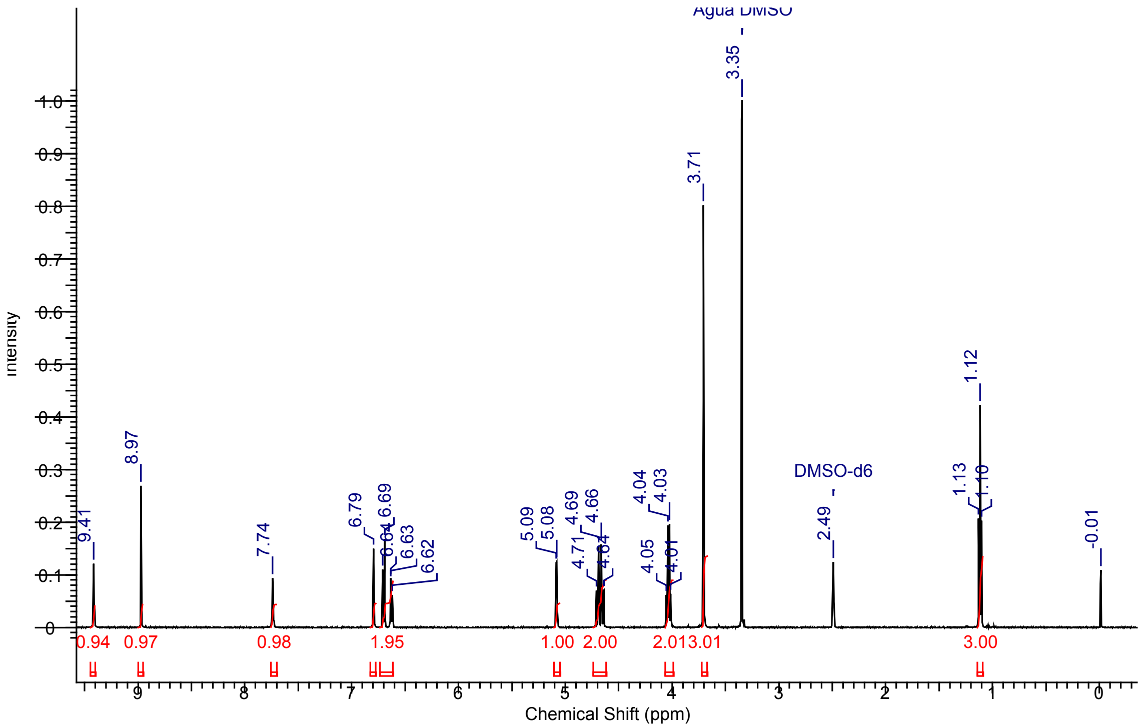

^{13}C NMR

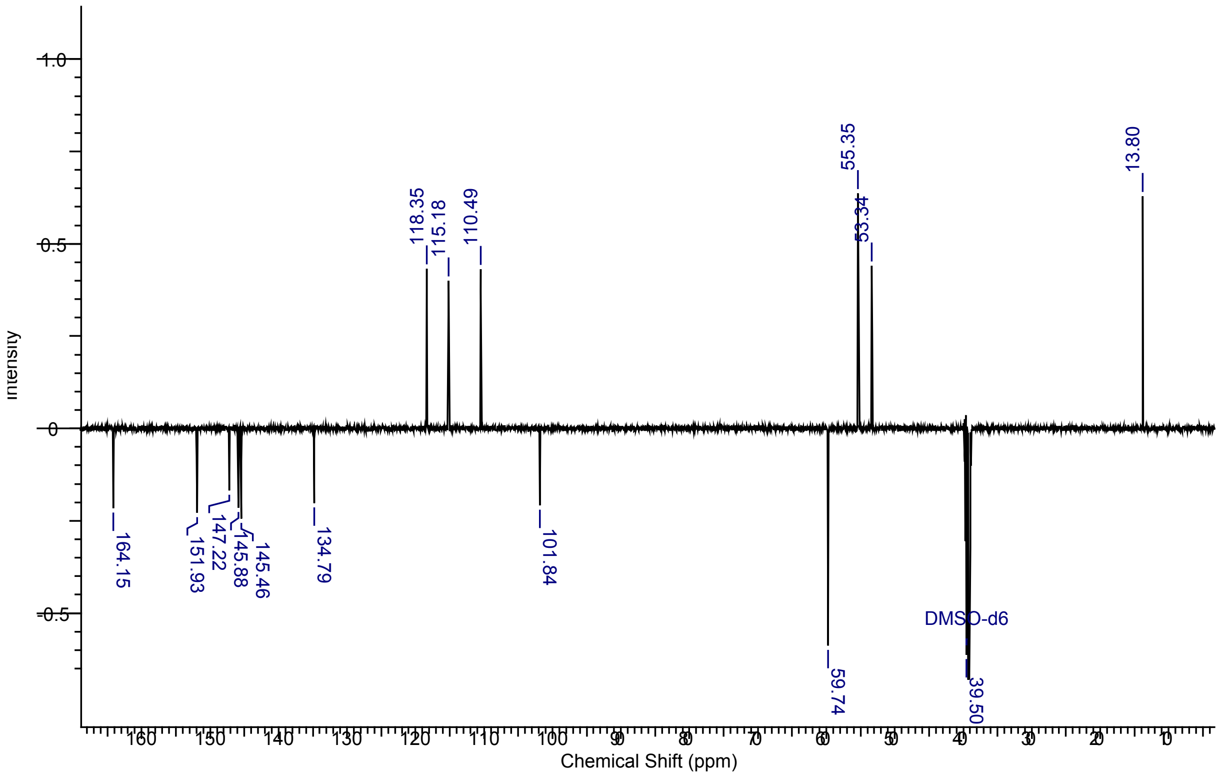

FT-IR

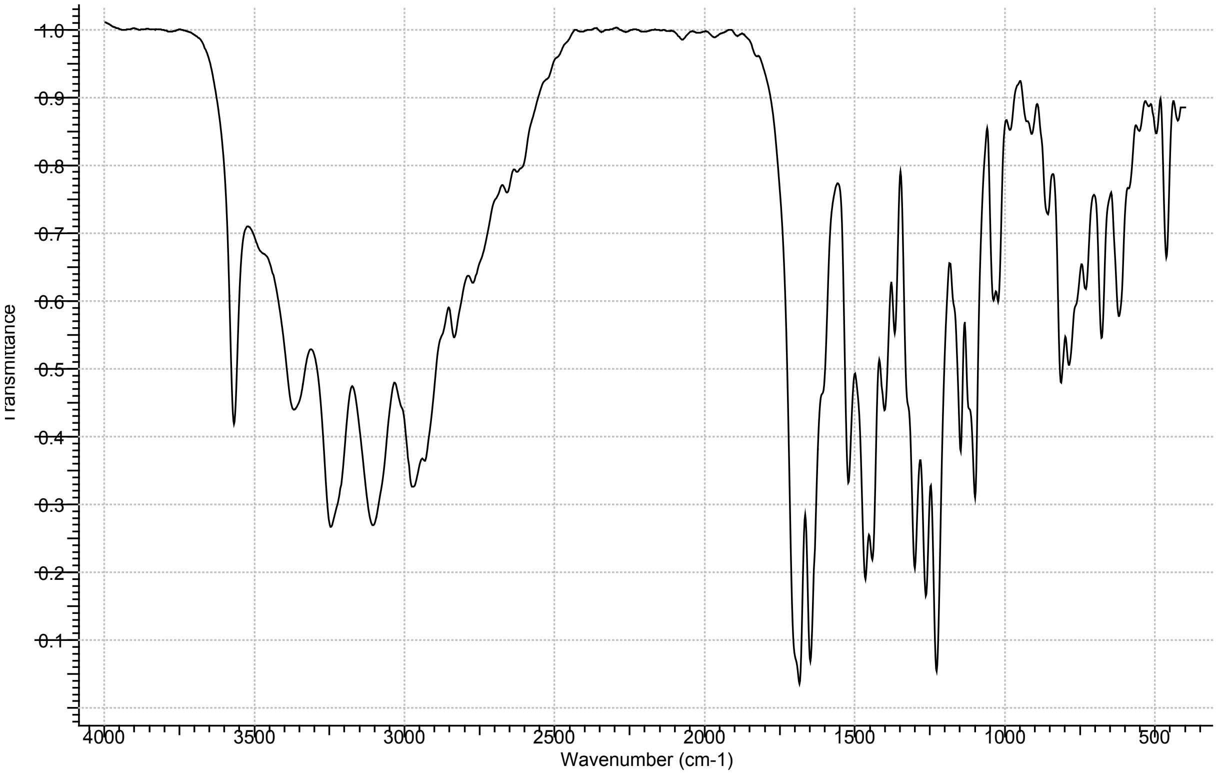

Mass Spectrometry

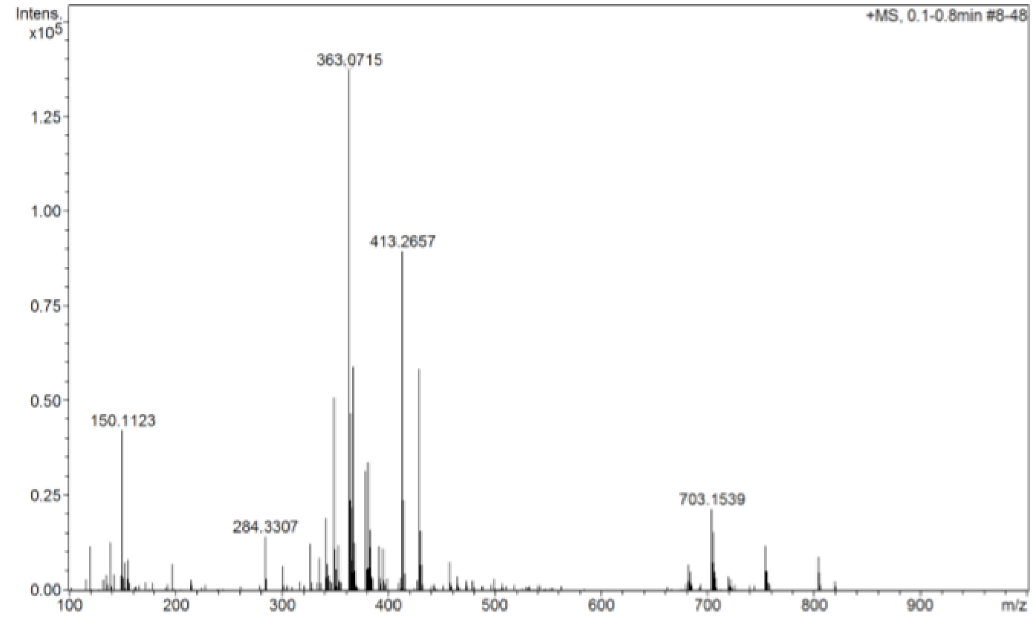

Meas. m/z	#	Ion Formula	m/z	err [ppm]	Mean err [ppm]	rdb	N-Rule	e⁻ Conf	mSigma	Std I	Std Mean m/z	Std I VarNorm	Std m/z Diff	Std Comb Dev
363.071493	1	C15H17ClN2NaO5	363.071820	0.9	-1.4	7.5	ok	even	1.9	2.6	n.a.	n.a.	n.a.	n.a.
	2	C11H13ClN8NaO3	363.069135	-6.5	-7.7	8.5	ok	even	12.0	19.3	n.a.	n.a.	n.a.	n.a.
	3	C16H13ClN6NaO	363.073157	4.6	4.0	12.5	ok	even	12.3	18.3	n.a.	n.a.	n.a.	n.a.
	4	C10H17ClN4NaO7	363.067797	-10.2	-10.7	3.5	ok	even	24.1	38.2	n.a.	n.a.	n.a.	n.a.

Compound 2a

Chromatogram

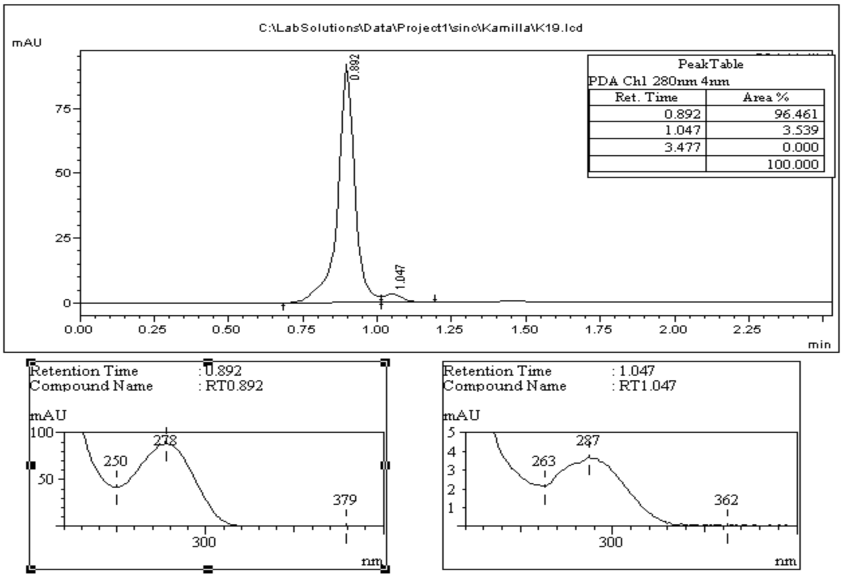

^{1}H NMR

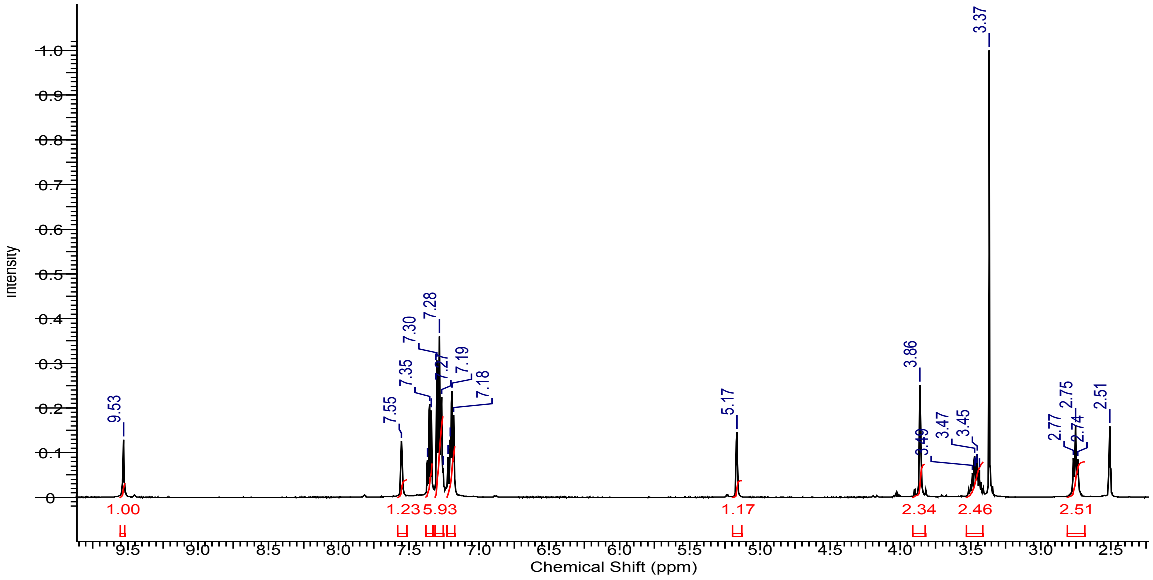

^{13}C NMR

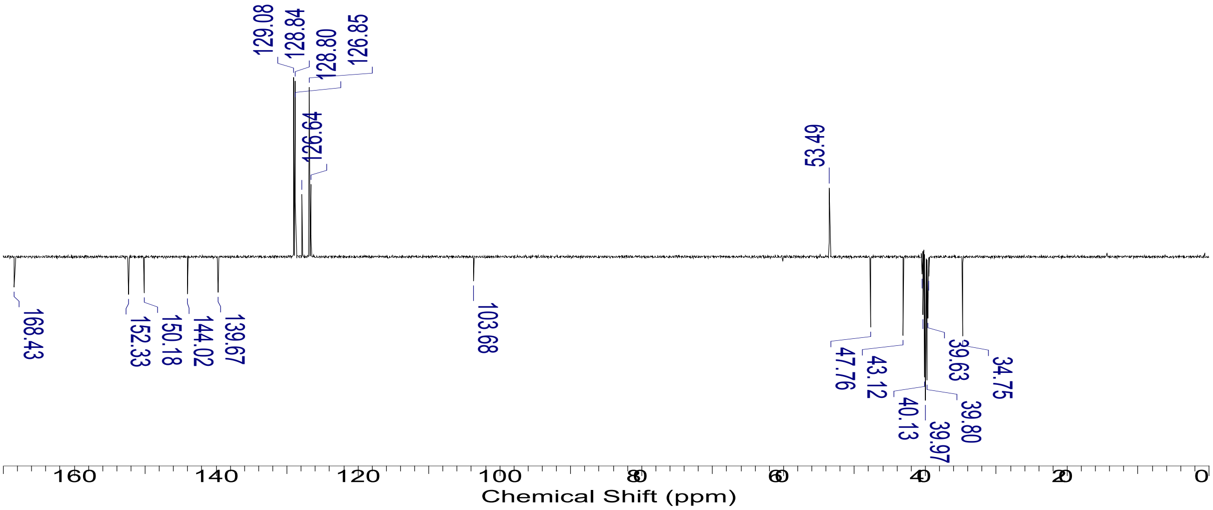

FT-IR

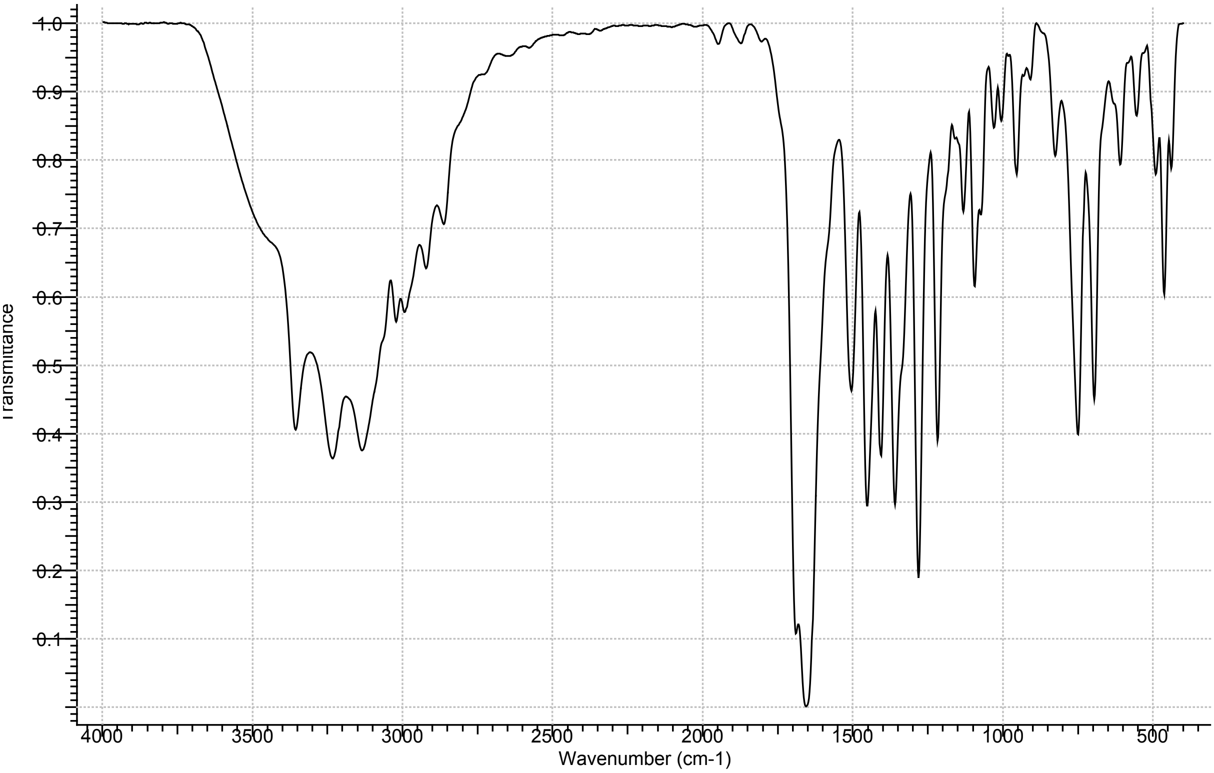

Mass Spectrometry

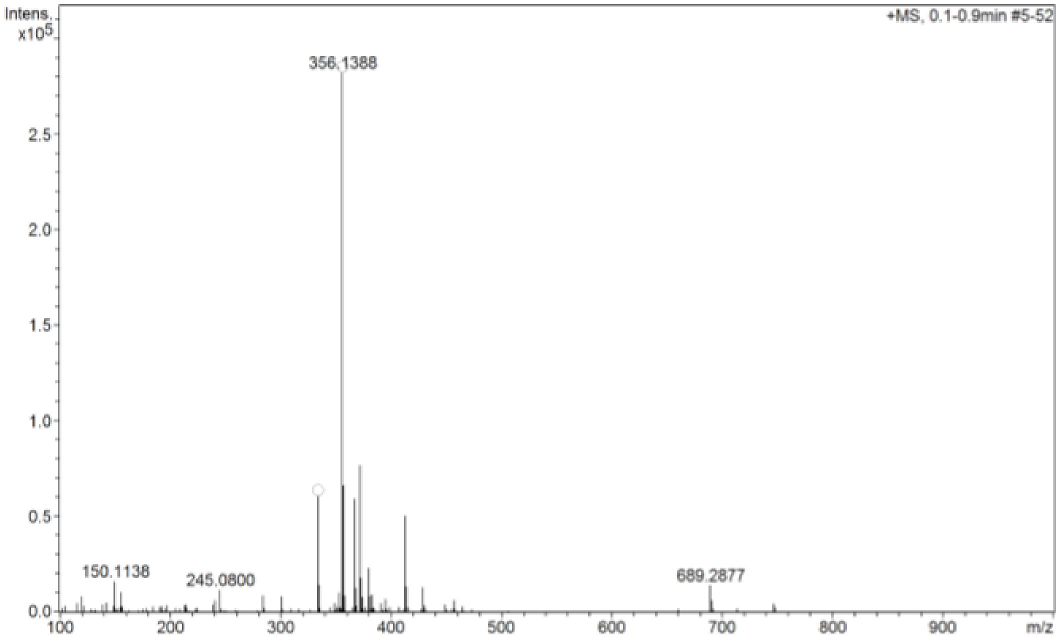

Meas. m/z	#	Ion Formula	m/z	err [ppm]	Mean err [ppm]	rdb	N-Rule	e⁻ Conf	mSigma	Std I	Std Mean m/z	Std I VarNorm	Std m/z Diff	Std Comb Dev
334.156621	1	C20H20N3O2	334.155003	-4.8	-4.8	12.5	ok	even	1.2	2.4	n.a.	n.a.	n.a.	n.a.
	2	C25H20N	334.159026	7.2	7.9	16.5	ok	even	26.0	40.7	n.a.	n.a.	n.a.	n.a.
356.138837	1	C20H19N3NaO2	356.136948	-5.3	-5.2	12.5	ok	even	2.6	4.2	n.a.	n.a.	n.a.	n.a.
	2	C25H19NNa	356.140970	6.0	6.7	16.5	ok	even	24.3	38.4	n.a.	n.a.	n.a.	n.a.

Compound 2b

Chromatogram

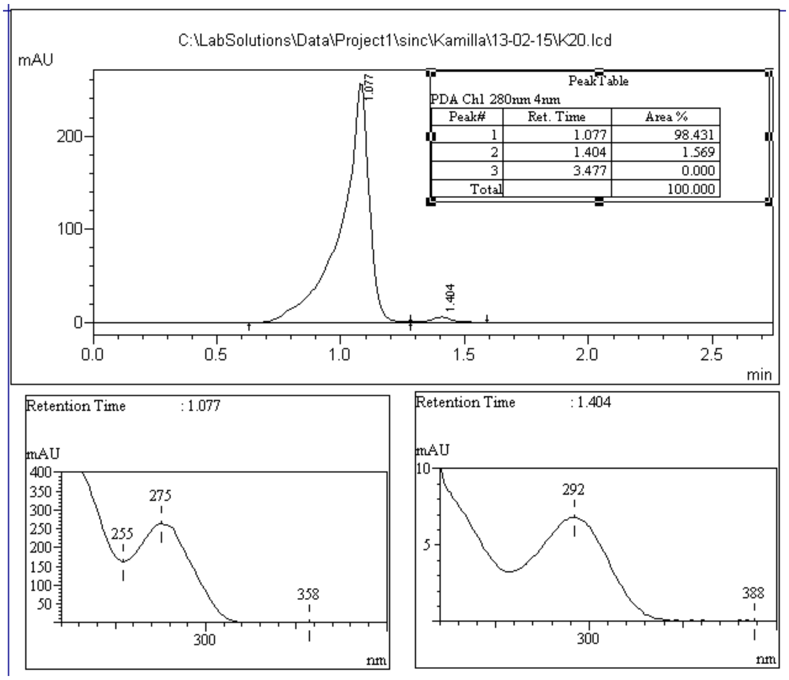

^{1}H NMR

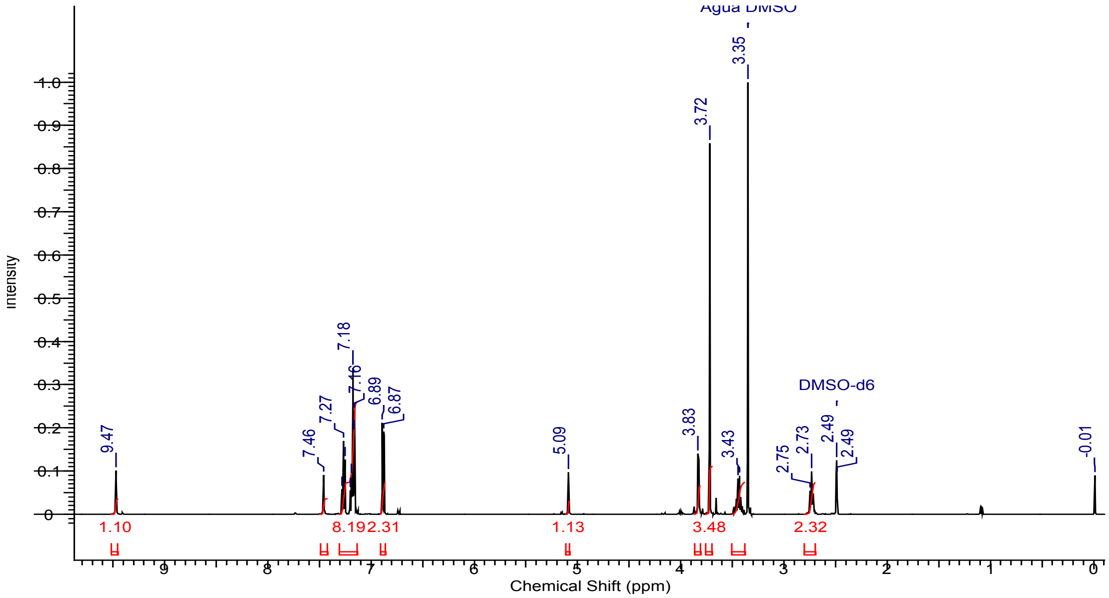
Agua DMSO
3.35
3.72
7.18
7.16
6.89
6.87
7.27
7.46
9.47
5.09
3.83
3.43
2.75
2.73
2.49
2.49
DMSO-d6
-0.01
1.10
8.19
2.31
1.13
3.48
2.32
Intensity
Chemical Shift (ppm)

^{13}C NMR

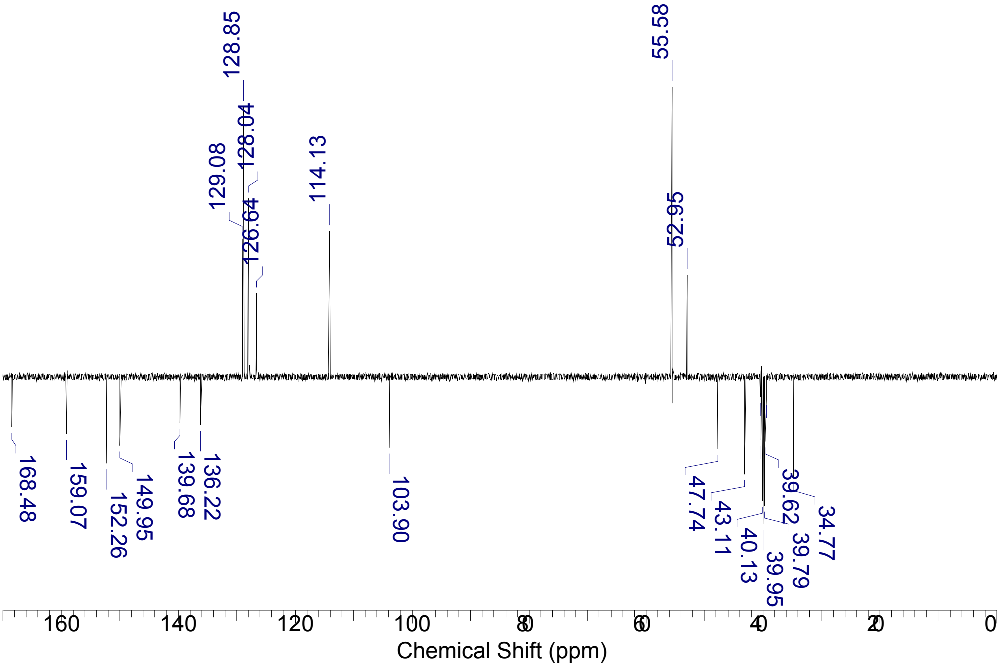
128.85
129.08
128.04
126.64
114.13
55.58
52.95
168.48
159.07
152.26
149.95
139.68
136.22
103.90
47.74
43.11
40.13
39.95
39.62
39.79
34.77
Chemical Shift (ppm)

FT-IR

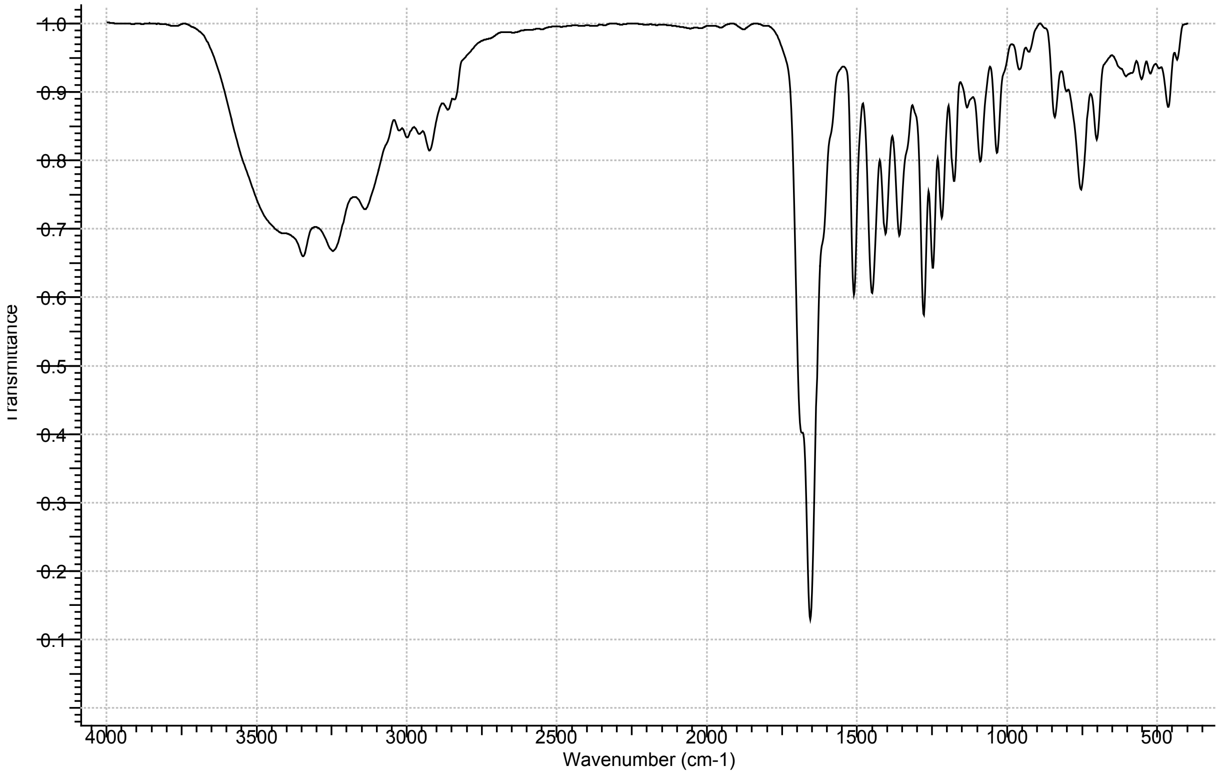

Mass Spectrometry

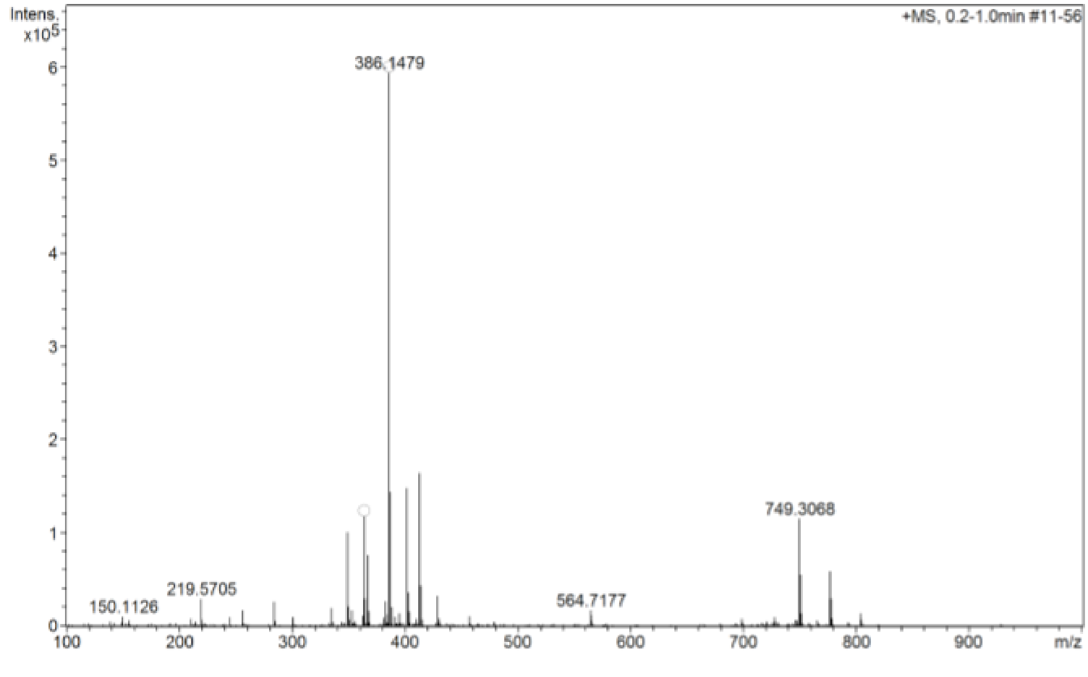

Meas. m/z	#	Ion Formula	m/z	err [ppm]	Mean err [ppm]	rdb	N-Rule	e^- Conf	mSigma	Std I	Std Mean m/z	Std I VarNorm	Std m/z Diff	Std Comb Dev
364.165821	1	$C_{21}H_{22}N_3O_3$	364.165568	-0.7	1126.5	12.5	ok	even	20.3	33.0	n.a.	n.a.	n.a.	n.a.
	2	$C_{17}H_{18}N_9O$	364.162883	-8.1	1118.4	13.5	ok	even	23.1	31.7	n.a.	n.a.	n.a.	n.a.
	3	$C_{26}H_{22}NO$	364.169591	10.4	1128.9	16.5	ok	even	32.4	59.3	n.a.	n.a.	n.a.	n.a.
386.147853	1	$C_{21}H_{21}N_3NaO_3$	386.147512	-0.9	-0.8	12.5	ok	even	0.5	0.9	n.a.	n.a.	n.a.	n.a.
	2	$C_{17}H_{17}N_9NaO$	386.144827	-7.8	-8.8	13.5	ok	even	14.5	25.8	n.a.	n.a.	n.a.	n.a.
	3	$C_{26}H_{21}NNaO$	386.151535	9.5	10.0	16.5	ok	even	26.5	41.0	n.a.	n.a.	n.a.	n.a.

Compound 2c

Chromatogram

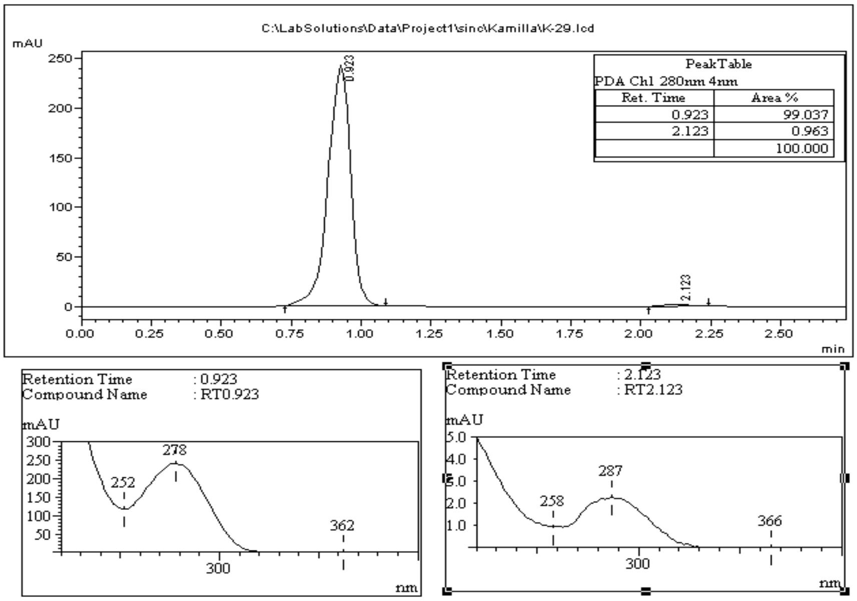

^{1}H NMR

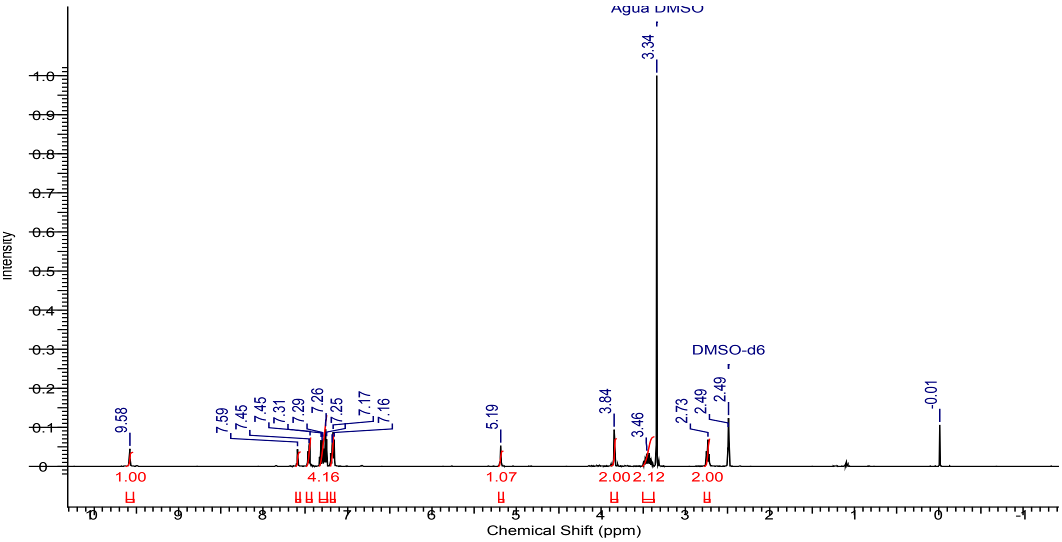

^{13}C NMR

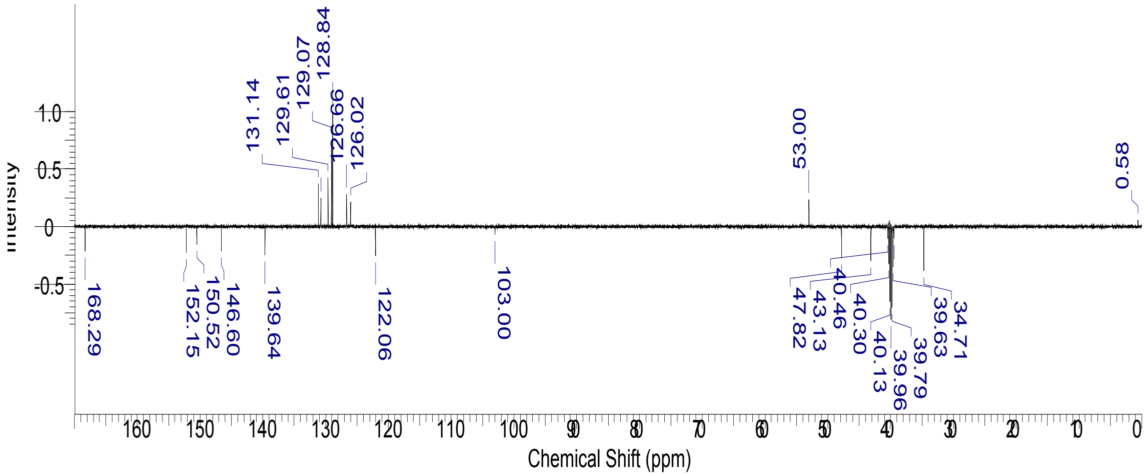

FT-IR

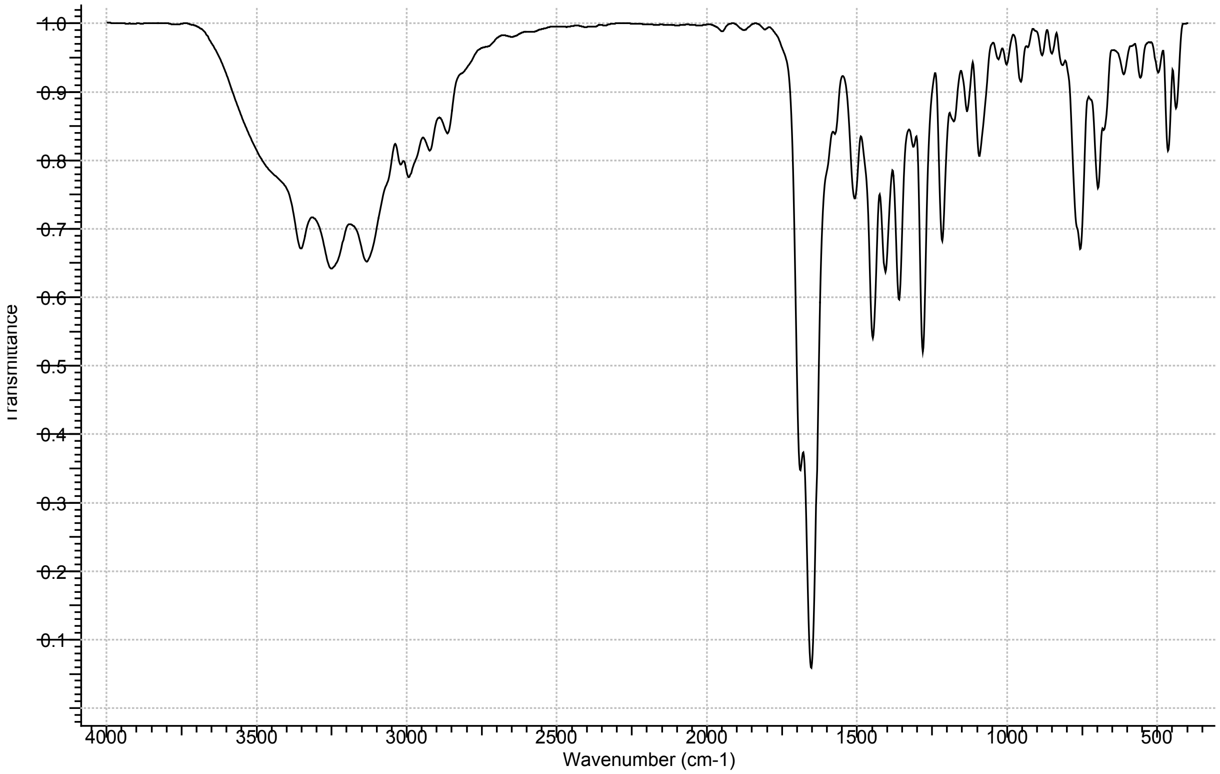

Mass Spectrometry

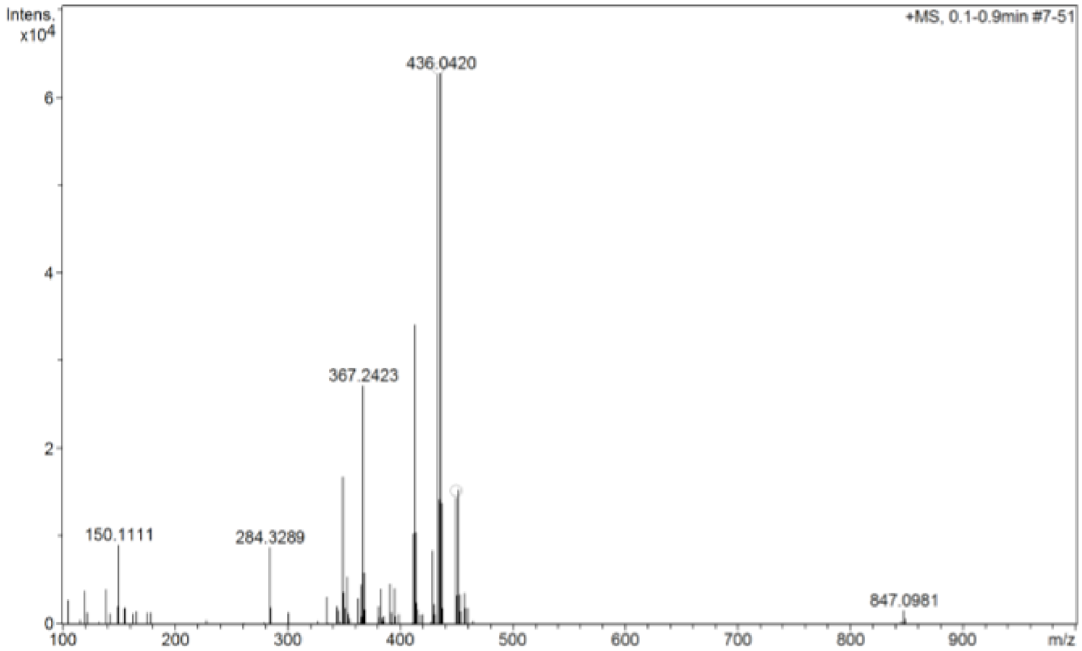

Meas. m/z	#	Ion Formula	m/z	err [ppm]	Mean err [ppm]	rdb	N-Rule	e⁻ Conf	mSigma	Std I	Std Mean m/z	Std I VarNorm	Std m/z Diff	Std Comb Dev
434.043746	1	C20H18BrN3NaO2	434.047460	8.6	8.4	12.5	ok	even	3.0	2.9	n.a.	n.a.	n.a.	n.a.
	2	C16H14BrN9Na	434.044774	2.4	1.4	13.5	ok	even	12.6	13.9	n.a.	n.a.	n.a.	n.a.
	3	C15H18BrN5NaO4	434.043437	-0.7	-1.3	8.5	ok	even	25.9	28.6	n.a.	n.a.	n.a.	n.a.
	4	C14H22BrNNaO8	434.042099	-3.8	-4.0	3.5	ok	even	40.3	44.4	n.a.	n.a.	n.a.	n.a.
450.017368	1	C20H18BrKN3O2	450.021397	9.0	1154.1	12.5	ok	even	9.4	11.5	n.a.	n.a.	n.a.	n.a.
	2	C16H14BrKN9	450.018712	3.0	1149.9	13.5	ok	even	10.8	10.6	n.a.	n.a.	n.a.	n.a.
	3	C15H18BrKN5O4	450.017374	0.0	1154.0	8.5	ok	even	22.6	24.1	n.a.	n.a.	n.a.	n.a.
	4	C14H22BrKNO8	450.016037	-3.0	1157.8	3.5	ok	even	35.4	39.2	n.a.	n.a.	n.a.	n.a.

Compound 2e

Chromatogram

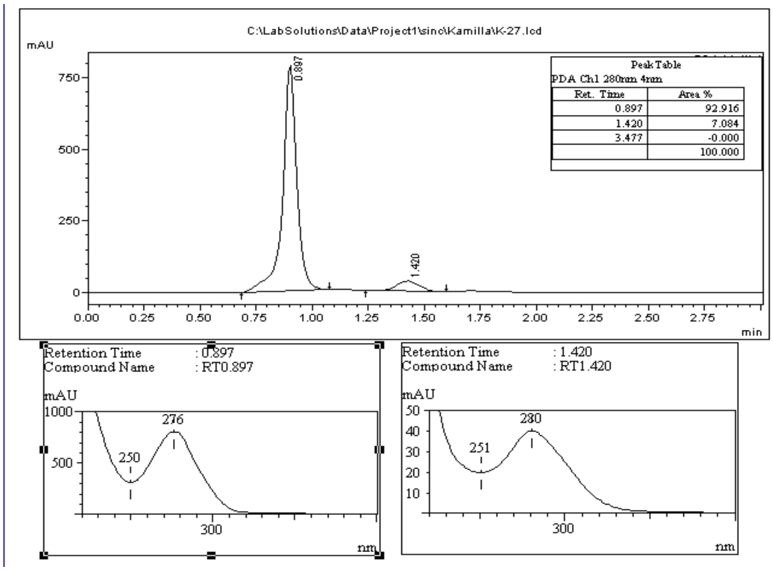

^{1}H NMR

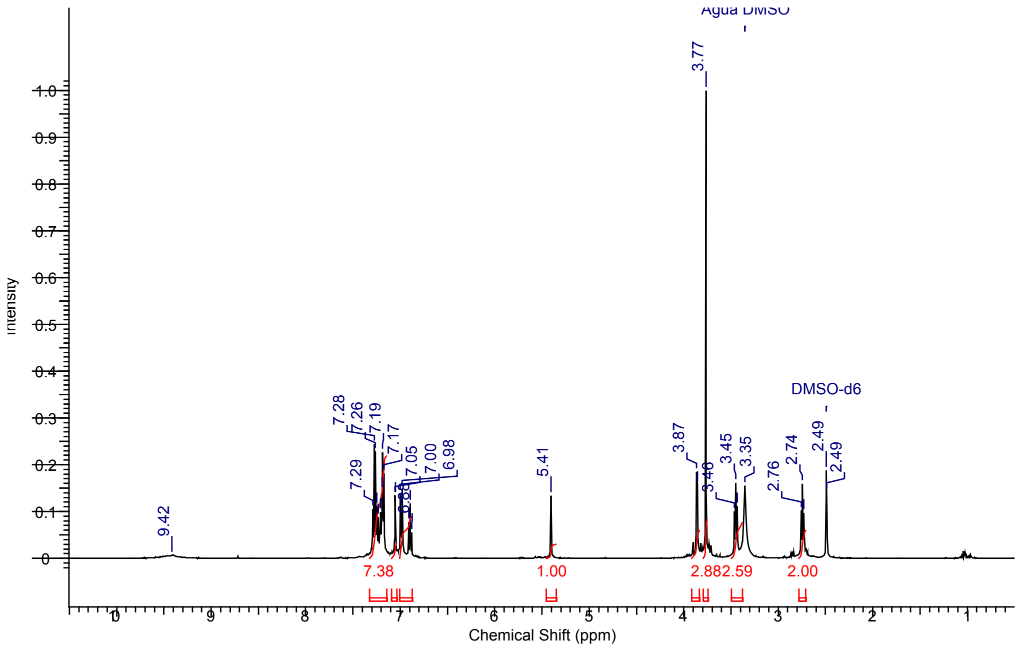
Agua DMSO
DMSO-d6
Intensity
Chemical Shift (ppm)
9.42
7.29
7.28
7.26
7.19
7.17
7.05
7.00
6.98
5.41
3.87
3.77
3.46
3.45
3.35
2.76
2.74
2.49
2.49
7.38
1.00
2.88
2.59
2.00

^{13}C NMR

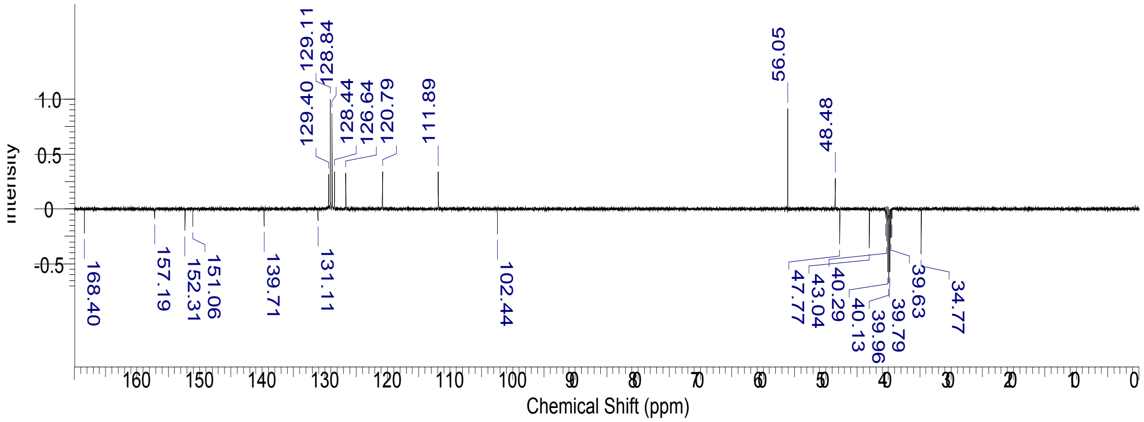
Intensity
Chemical Shift (ppm)
129.40
129.11
128.84
128.44
126.64
120.79
111.89
56.05
48.48
168.40
157.19
152.31
151.06
139.71
131.11
102.44
47.77
43.04
40.29
40.13
39.96
39.79
39.63
34.77

FT-IR

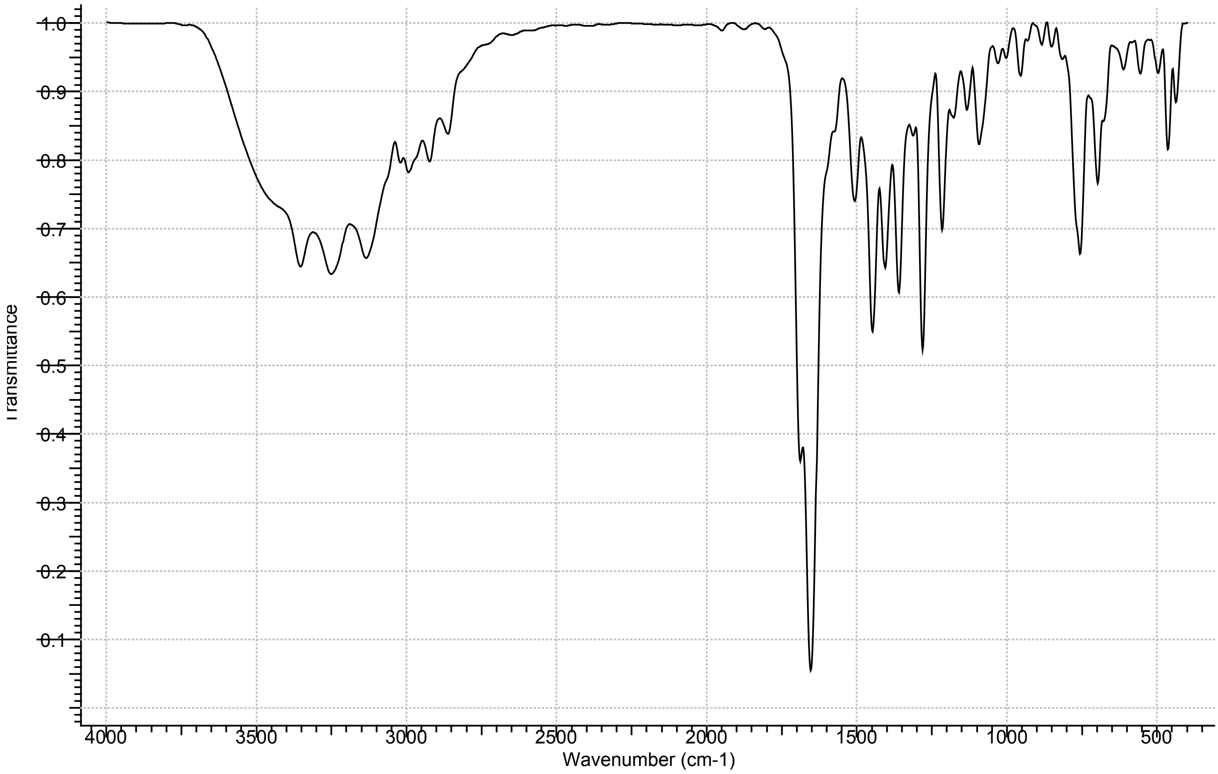

Mass Spectrometry

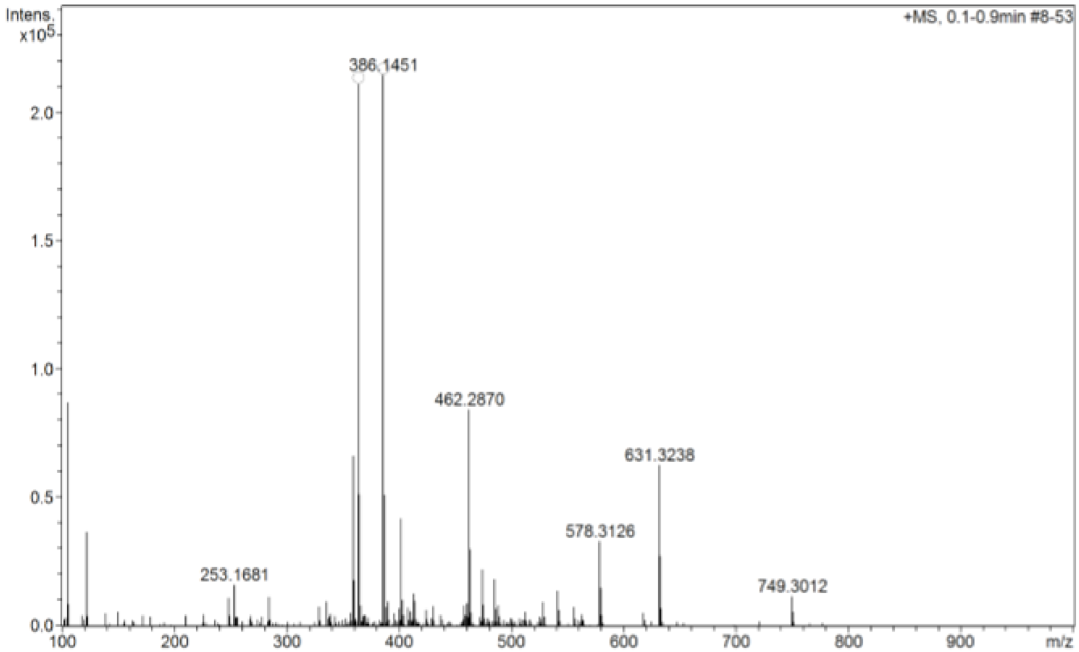

Meas. m/z	#	Ion Formula	m/z	err [ppm]	Mean err [ppm]	rdb	N-Rule	e⁻ Conf	mSigma	Std I	Std Mean m/z	Std I VarNorm	Std m/z Diff	Std Comb Dev
364.163322	1	C21H22N3O3	364.165588	6.2	5.7	12.5	ok	even	2.3	4.3	n.a.	n.a.	n.a.	n.a.
	2	C17H18N9O	364.162883	-1.2	-2.8	13.5	ok	even	15.9	28.7	n.a.	n.a.	n.a.	n.a.
	3	C16H22N5O5	364.161545	-4.9	-6.0	8.5	ok	even	28.2	47.8	n.a.	n.a.	n.a.	n.a.
386.145051	1	C21H21N3NaO3	386.147512	6.4	6.5	12.5	ok	even	1.7	2.6	n.a.	n.a.	n.a.	n.a.
	2	C17H17N9NaO	386.144827	-0.6	-1.4	13.5	ok	even	12.5	22.4	n.a.	n.a.	n.a.	n.a.
	3	C16H21N5NaO5	386.143489	-4.0	-4.5	8.5	ok	even	25.1	41.9	n.a.	n.a.	n.a.	n.a.
	4	C15H25NNaO9	386.142152	-7.5	-7.6	3.5	ok	even	38.4	63.0	n.a.	n.a.	n.a.	n.a.

Compound 2f
Chromatogram

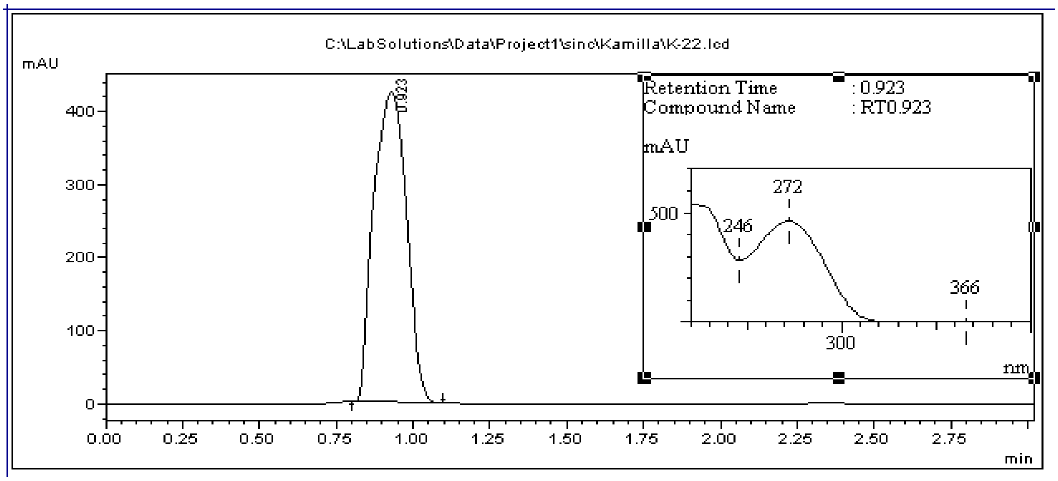

^{1}H NMR

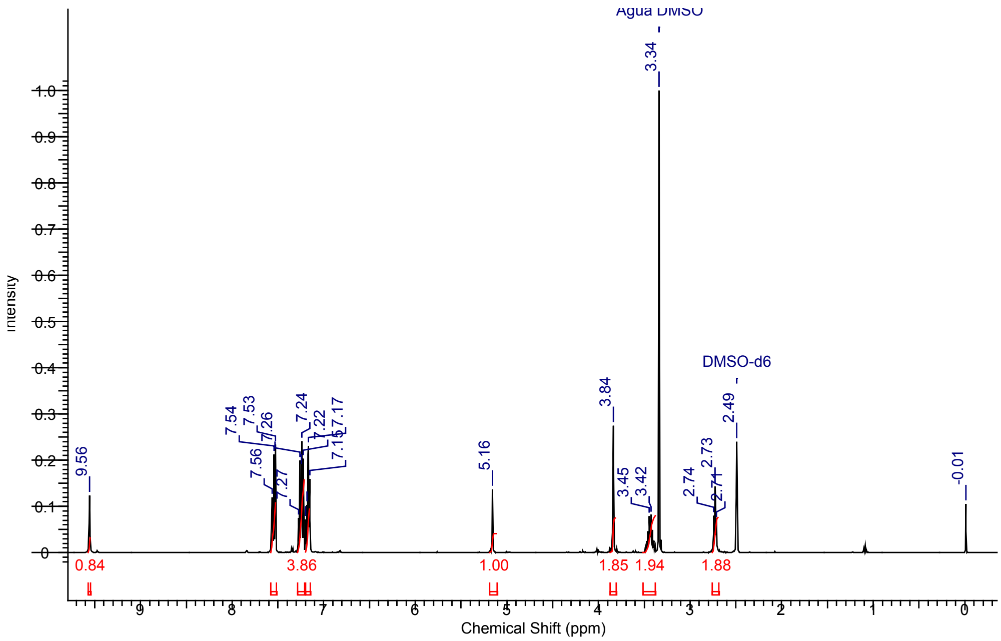

^{13}C NMR

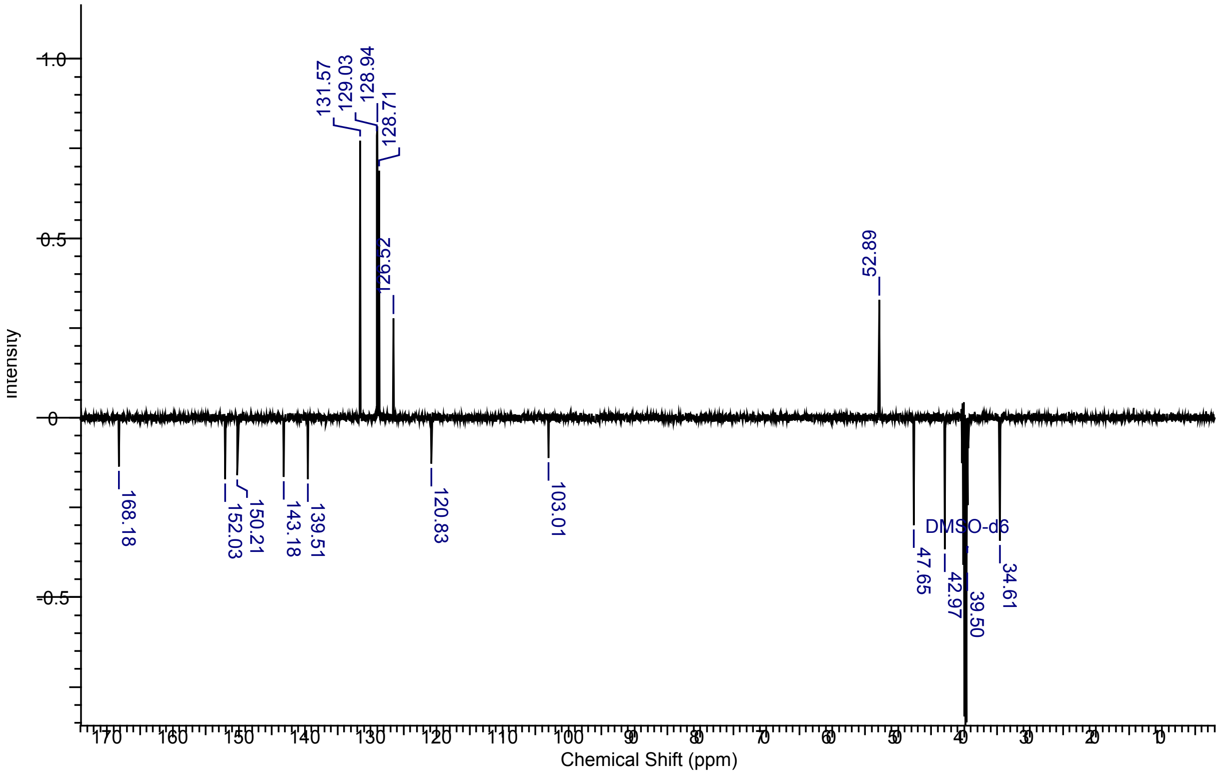

FT-IR

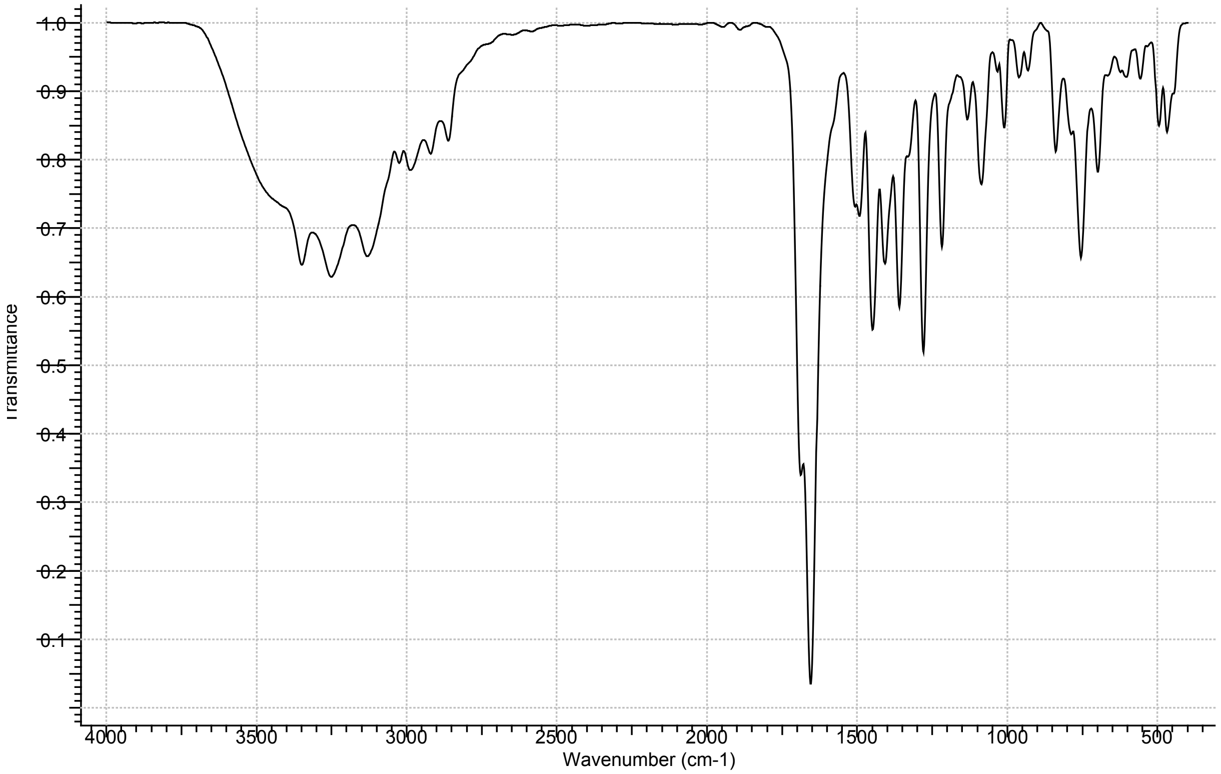

Mass Spectrometry

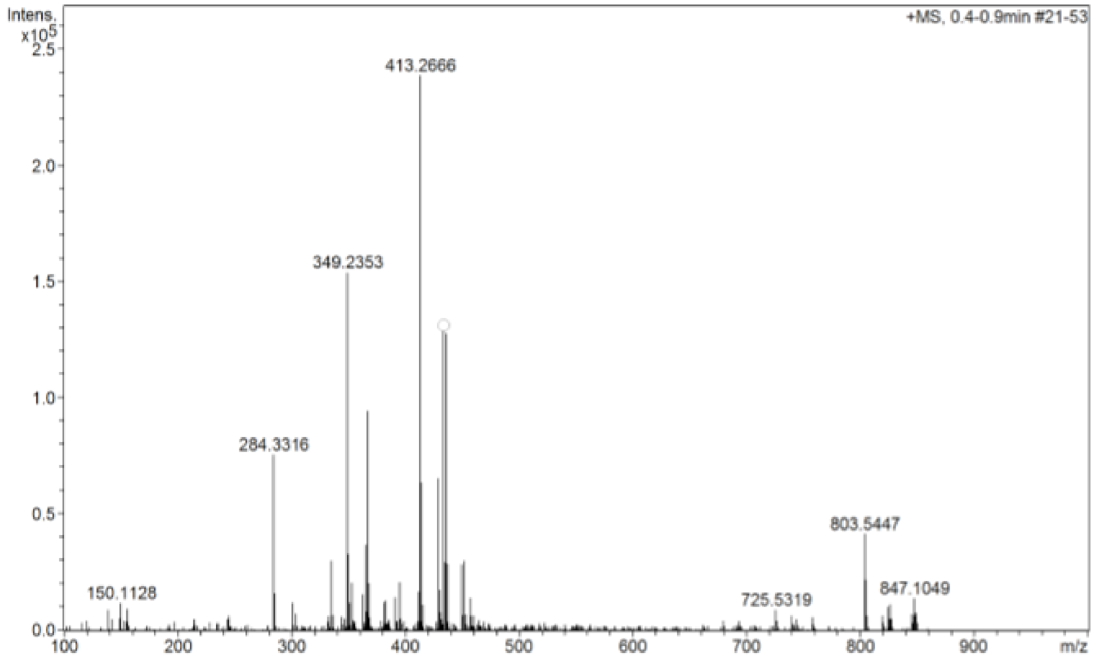

Meas. m/z	#	Ion Formula	m/z	err [ppm]	Mean err [ppm]	rdb	N-Rule	e⁻ Conf	mSigma	Std I	Std Mean m/z	Std I VarNorm	Std m/z Diff	Std Comb Dev
434.047519	1	C20H18BrN3NaO2	434.047460	-0.1	0.0	12.5	ok	even	4.1	3.3	n.a.	n.a.	n.a.	n.a.
	2	C16H14BrN9Na	434.044774	-6.3	-7.0	13.5	ok	even	13.2	15.0	n.a.	n.a.	n.a.	n.a.
	3	C25H18BrNNa	434.051482	9.1	9.7	16.5	ok	even	29.8	30.6	n.a.	n.a.	n.a.	n.a.

Compound 2g

Chromatogram

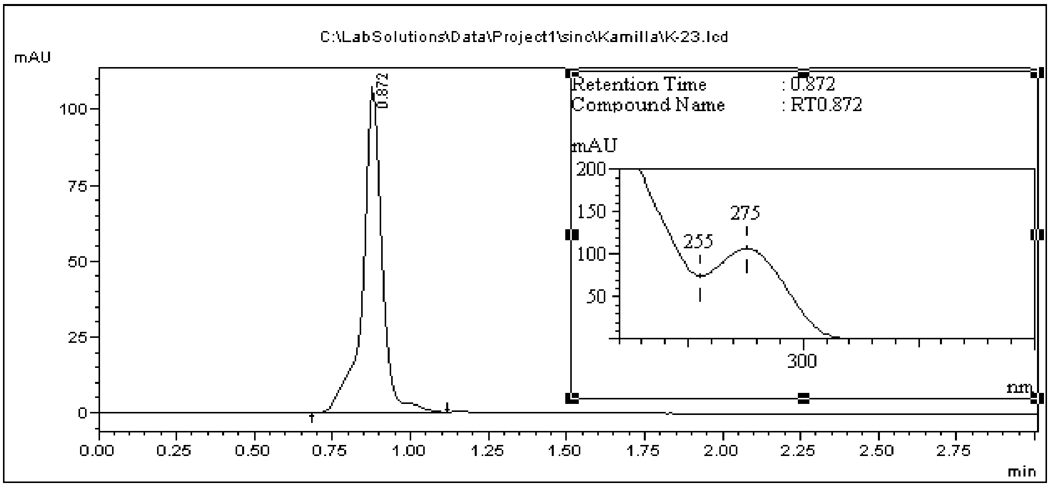

^{1}H NMR

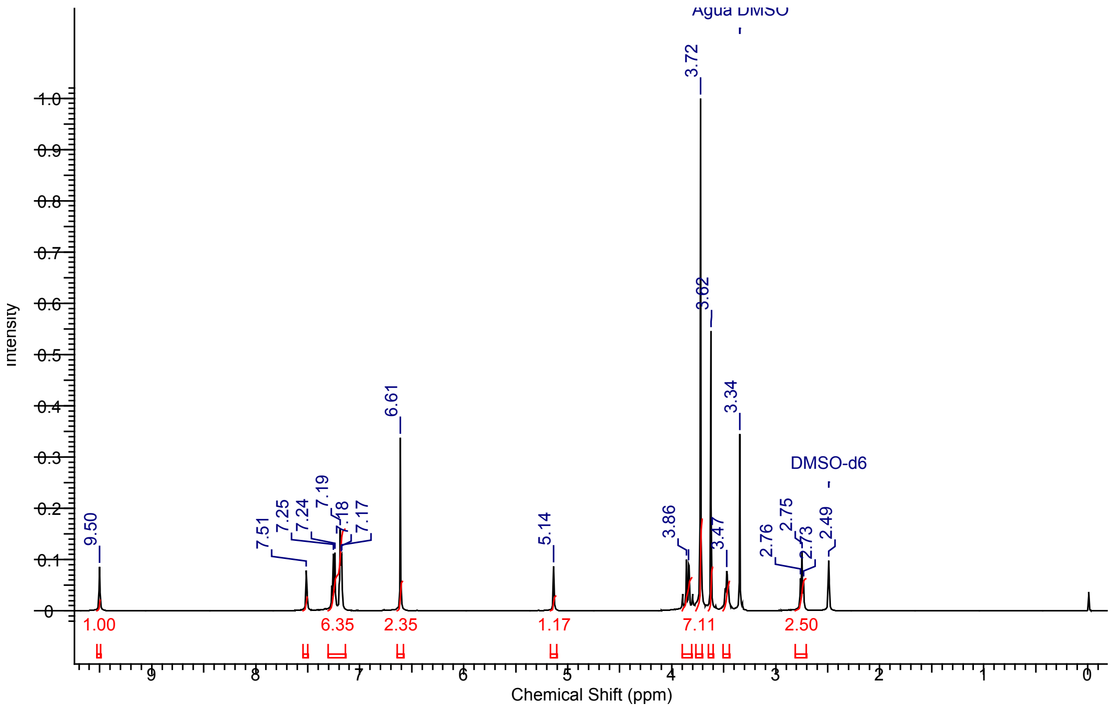
Agua DMSO
3.72
3.62
3.34
6.61
DMSO-d6
9.50
7.51
7.25
7.24
7.19
7.18
7.17
5.14
3.86
3.47
2.76
2.75
2.73
2.49
1.00
6.35
2.35
1.17
7.11
2.50
Intensity
Chemical Shift (ppm)

^{13}C NMR

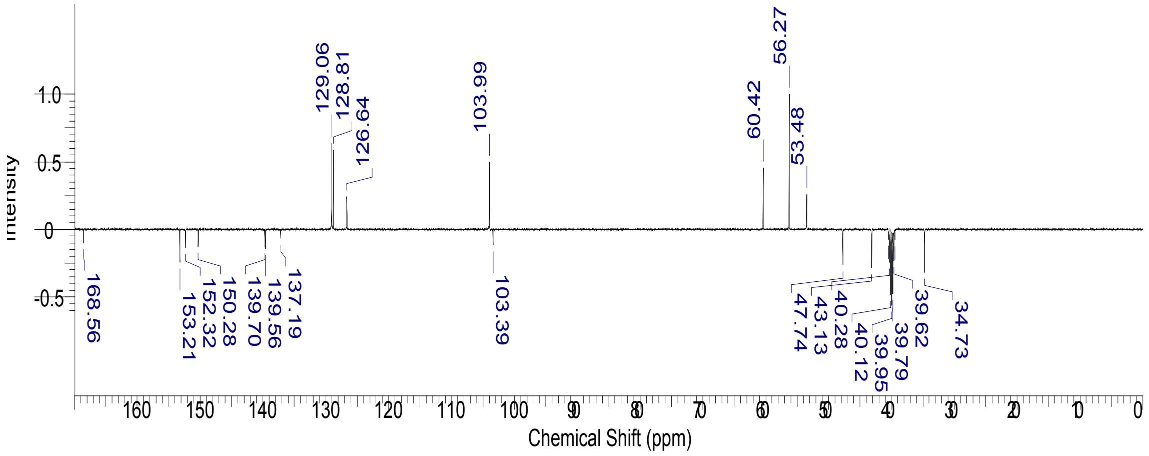
129.06
128.81
126.64
103.99
60.42
56.27
53.48
168.56
153.21
152.32
150.28
139.70
139.56
137.19
103.39
47.74
43.13
40.28
40.12
39.95
39.79
39.62
34.73
Intensity
Chemical Shift (ppm)

FT-IR

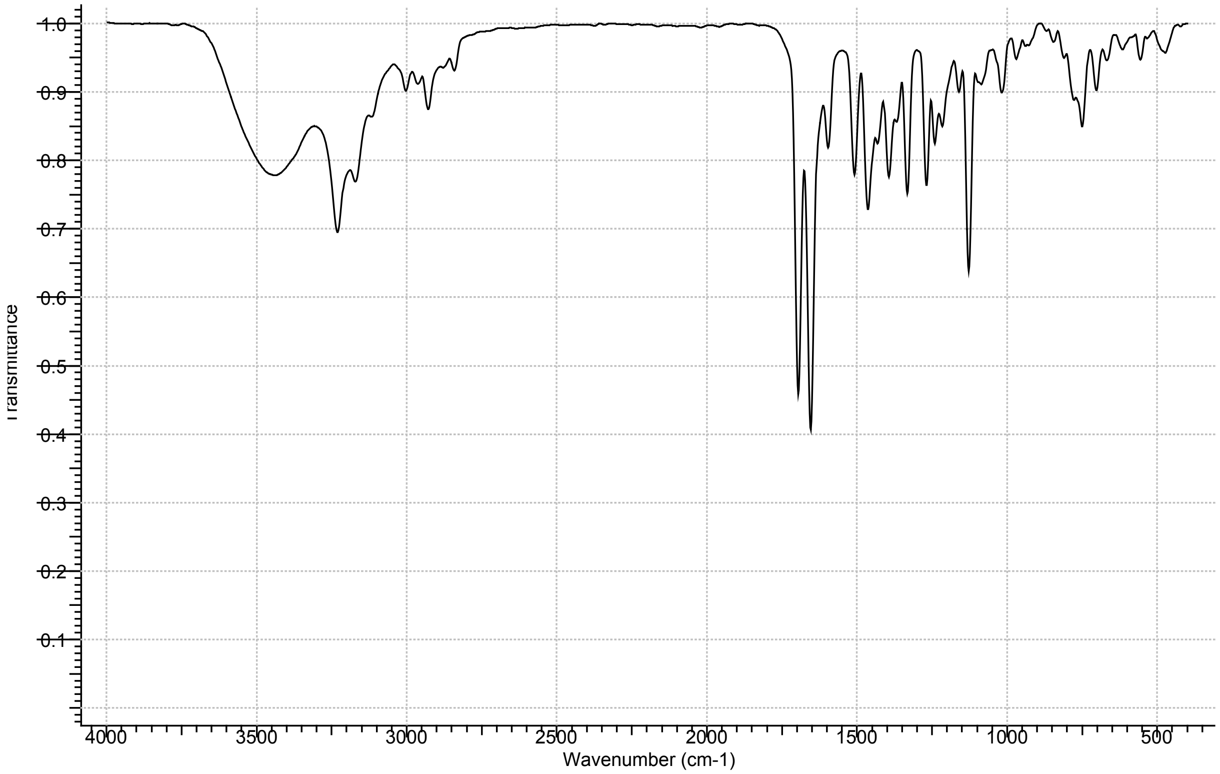

Mass Spectrometry

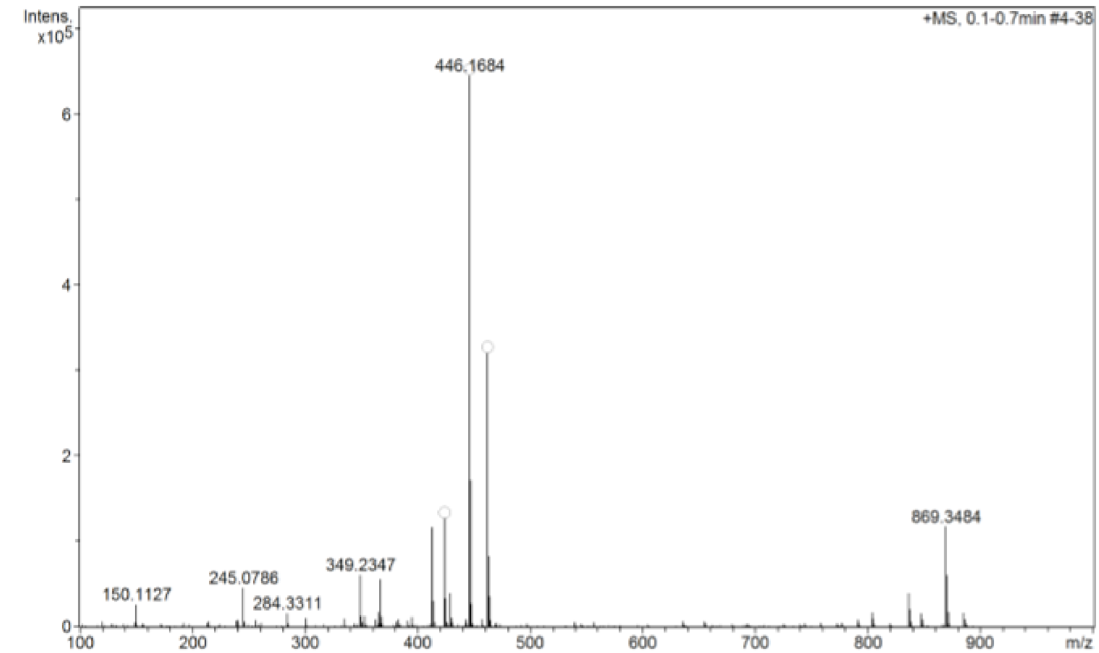

Meas. m/z	#	Ion Formula	m/z	err [ppm]	Mean err [ppm]	rdb	N-Rule	e⁻ Conf	mSigma	Std I	Std Mean m/z	Std I VarNorm	Std m/z Diff	Std Comb Dev
424.186458	1	C23H26N3O5	424.186697	0.6	0.8	12.5	ok	even	0.9	1.2	n.a.	n.a.	n.a.	n.a.
	2	C19H22N9O3	424.184012	-5.8	-6.3	13.5	ok	even	13.4	23.0	n.a.	n.a.	n.a.	n.a.
	3	C24H22N7O	424.188035	3.7	3.7	17.5	ok	even	14.5	20.3	n.a.	n.a.	n.a.	n.a.
	4	C18H26N5O7	424.182675	-8.9	-9.1	8.5	ok	even	26.0	41.7	n.a.	n.a.	n.a.	n.a.
446.168442	1	C23H25N3NaO5	446.168642	0.4	0.7	12.5	ok	even	1.2	1.6	n.a.	n.a.	n.a.	n.a.
	2	C24H21N7NaO	446.169979	3.4	3.4	17.5	ok	even	12.7	18.1	n.a.	n.a.	n.a.	n.a.
	3	C19H21N9NaO3	446.165956	-5.6	-6.1	13.5	ok	even	14.7	24.4	n.a.	n.a.	n.a.	n.a.
	4	C18H25N5NaO7	446.164619	-8.6	-8.7	8.5	ok	even	27.6	43.6	n.a.	n.a.	n.a.	n.a.
462.142292	1	C23H25KN3O5	462.142579	0.6	1.3	12.5	ok	even	3.4	6.3	n.a.	n.a.	n.a.	n.a.
	2	C19H21KN9O3	462.139894	-5.2	-5.5	13.5	ok	even	9.3	16.5	n.a.	n.a.	n.a.	n.a.
	3	C24H21KN7O	462.143916	3.5	3.7	17.5	ok	even	14.3	22.6	n.a.	n.a.	n.a.	n.a.
	4	C18H25KN5O7	462.138556	-8.1	-7.8	8.5	ok	even	20.6	34.6	n.a.	n.a.	n.a.	n.a.

Compound 2h
Chromatogram

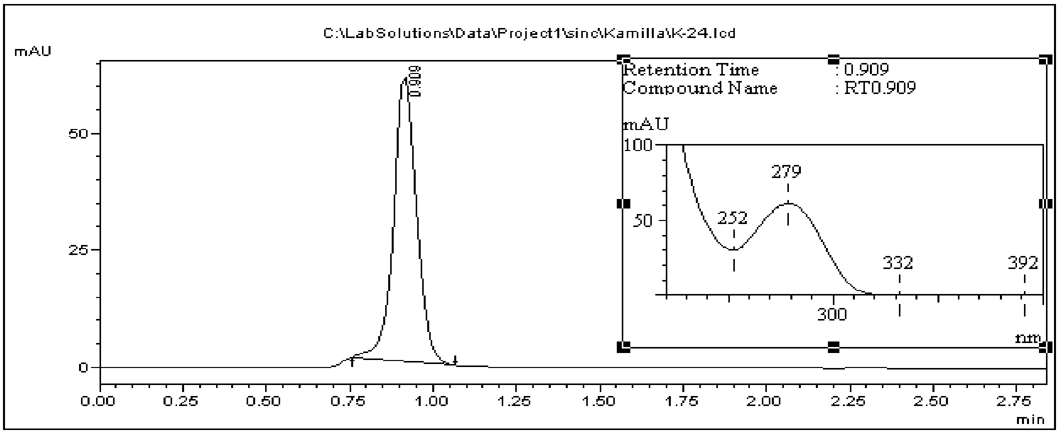

^{1}H NMR

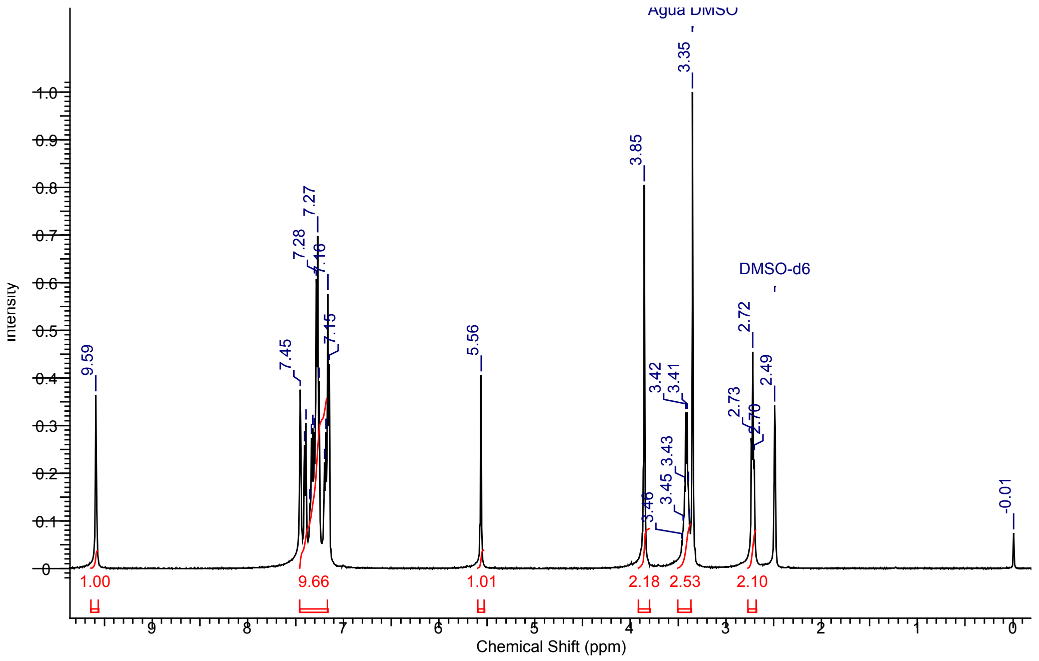
Agua DMSO
DMSO-d6
9.59
7.45
7.28
7.27
7.16
7.15
5.56
3.85
3.46
3.45
3.43
3.42
3.41
3.35
2.73
2.72
2.70
2.49
-0.01
1.00
9.66
1.01
2.18
2.53
2.10
Chemical Shift (ppm)

^{13}C NMR

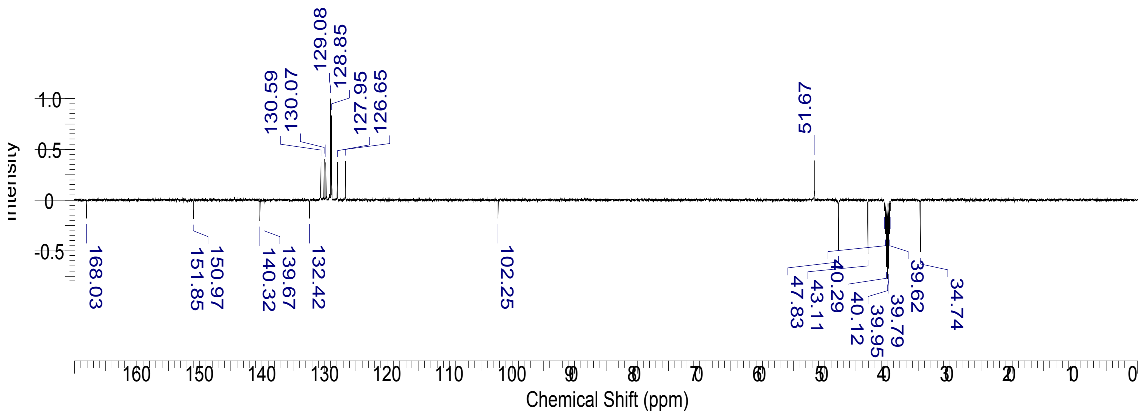
130.59
130.07
129.08
128.85
127.95
126.65
51.67
168.03
151.85
150.97
140.32
139.67
132.42
102.25
47.83
43.11
40.29
40.12
39.95
39.79
39.62
34.74
Chemical Shift (ppm)

FT-IR

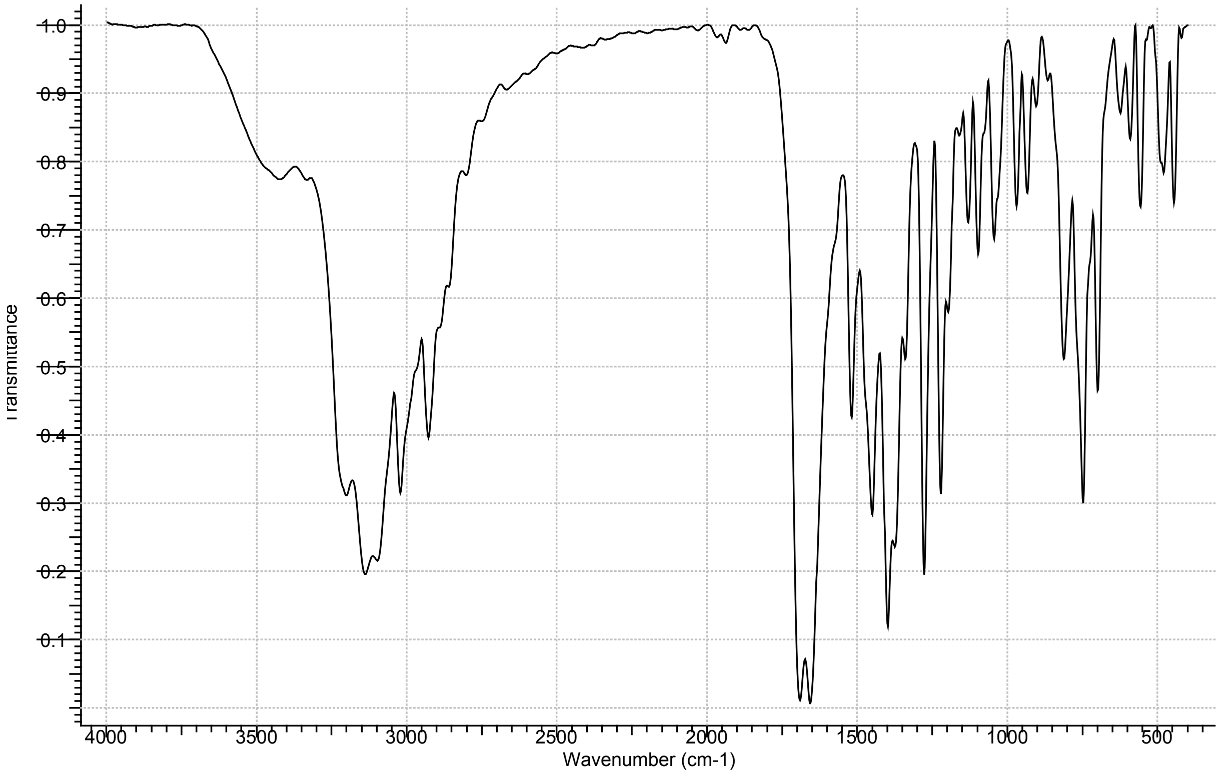

Mass Spectrometry

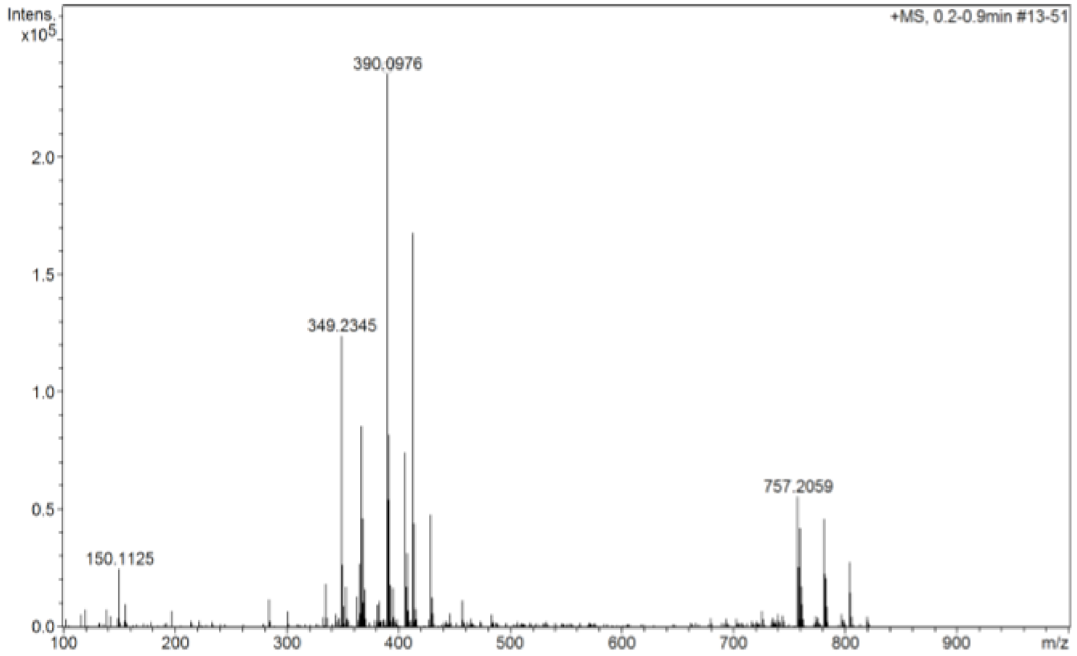

Meas. m/z	#	Ion Formula	m/z	err [ppm]	Mean err [ppm]	rdb	N-Rule	e⁻ Conf	mSigma	Std I	Std Mean m/z	Std I VarNorm	Std m/z Diff	Std Comb Dev
390.097591	1	C20H18ClN3NaO2	390.097975	1.0	1.4	12.5	ok	even	0.6	0.8	n.a.	n.a.	n.a.	n.a.
	2	C16H14ClN9Na	390.095290	-5.9	-6.3	13.5	ok	even	12.9	18.8	n.a.	n.a.	n.a.	n.a.
	3	C15H18ClN5NaO4	390.093952	-9.3	-9.2	8.5	ok	even	24.5	35.6	n.a.	n.a.	n.a.	n.a.

Compound 2j

Chromatogram

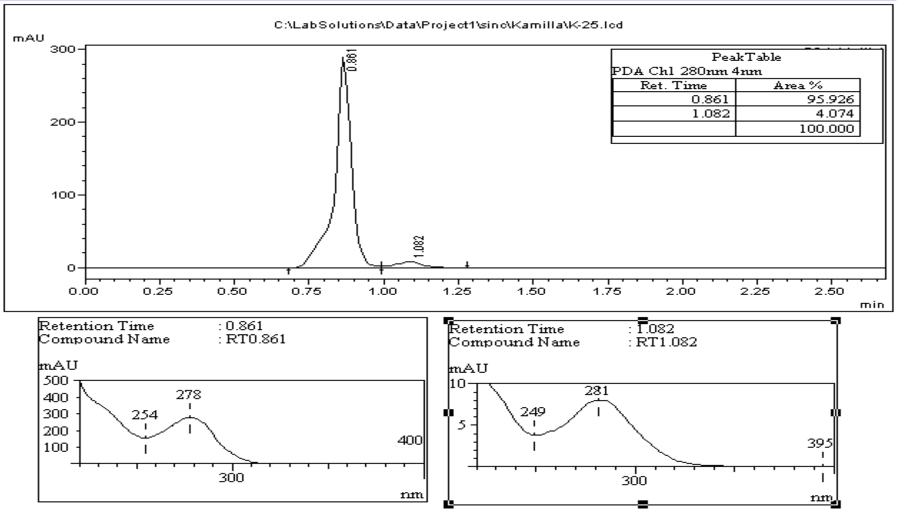

¹H NMR

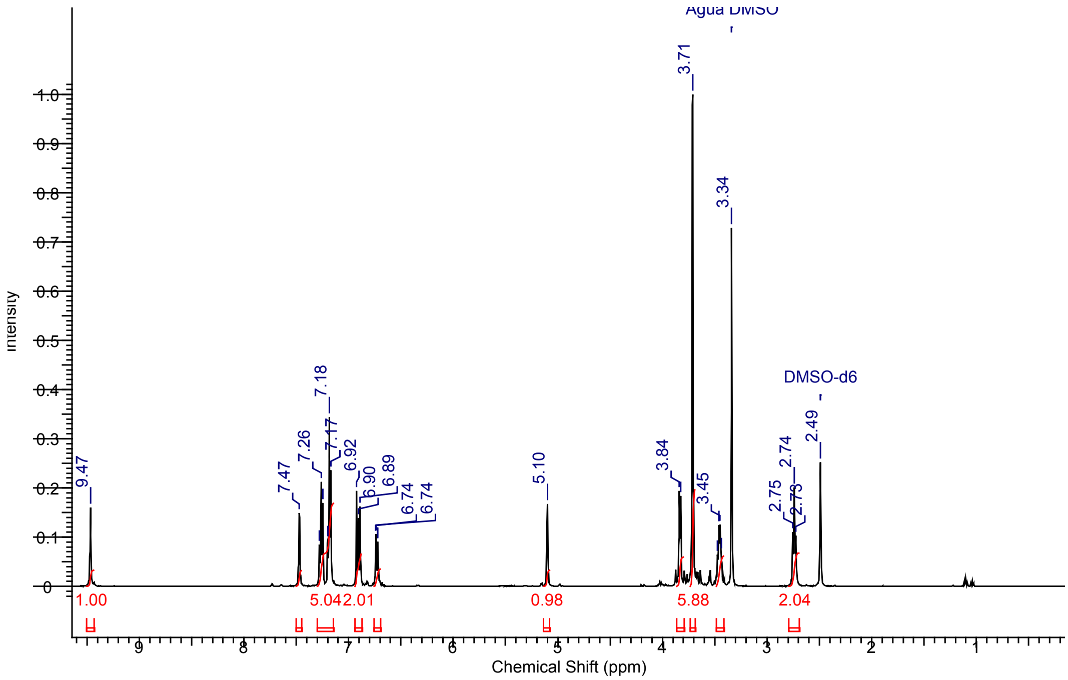

¹³C NMR

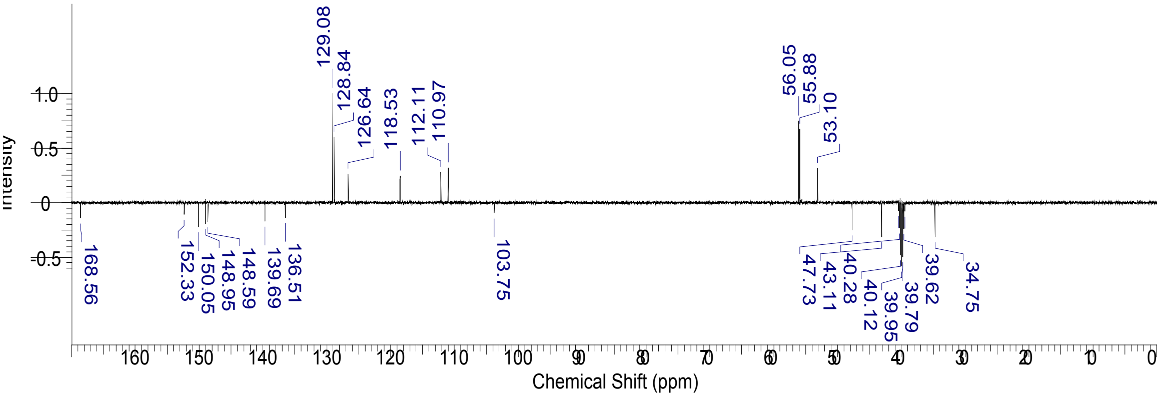

FT-IR

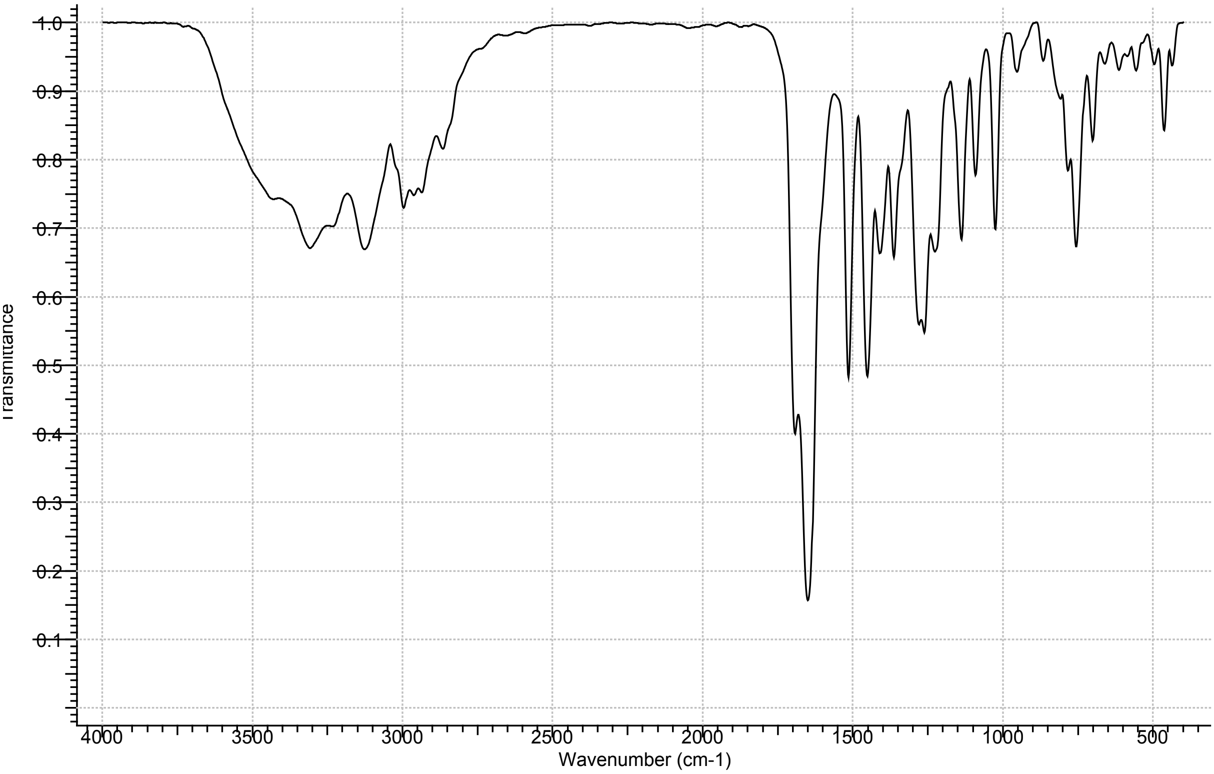

Mass Spectrometry

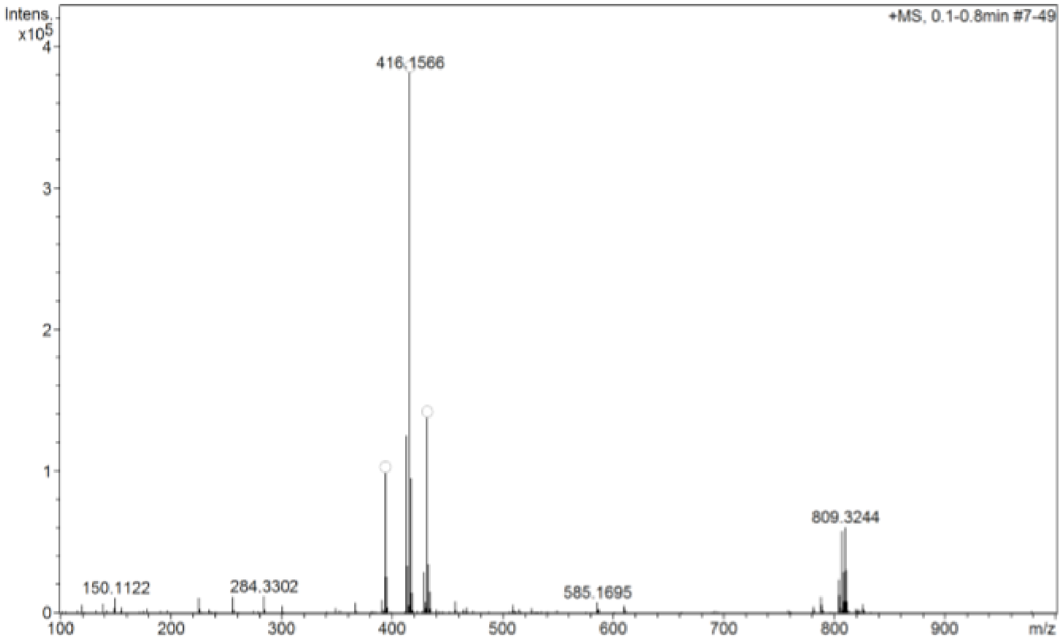

Meas. m/z	#	Ion Formula	m/z	err [ppm]	Mean err [ppm]	rdb	N-Rule	e⁻ Conf	mSigma	Std I	Std Mean m/z	Std I VarNorm	Std m/z Diff	Std Comb Dev
394.174746	1	$C_{22}H_{24}N_3O_4$	394.176133	3.5	3.8	12.5	ok	even	1.6	2.8	n.a.	n.a.	n.a.	n.a.
	2	$C_{23}H_{20}N_7$	394.177470	6.9	6.8	17.5	ok	even	12.1	17.0	n.a.	n.a.	n.a.	n.a.
	3	$C_{18}H_{20}N_9O_2$	394.173447	-3.3	-4.0	13.5	ok	even	15.8	27.2	n.a.	n.a.	n.a.	n.a.
	4	$C_{17}H_{24}N_5O_6$	394.172110	-6.7	-7.0	8.5	ok	even	28.4	46.4	n.a.	n.a.	n.a.	n.a.
416.156634	1	$C_{22}H_{23}N_3NaO_4$	416.158077	3.5	3.8	12.5	ok	even	2.0	3.3	n.a.	n.a.	n.a.	n.a.
	2	$C_{18}H_{19}N_9NaO_2$	416.155392	-3.0	-3.5	13.5	ok	even	12.1	21.1	n.a.	n.a.	n.a.	n.a.
	3	$C_{23}H_{19}N_7Na$	416.159414	6.7	6.7	17.5	ok	even	15.4	22.5	n.a.	n.a.	n.a.	n.a.
	4	$C_{17}H_{23}N_5NaO_6$	416.154054	-6.2	-6.3	8.5	ok	even	24.8	40.4	n.a.	n.a.	n.a.	n.a.
	5	$C_{16}H_{27}NNaO_{10}$	416.152717	-9.4	-9.2	3.5	ok	even	38.2	61.2	n.a.	n.a.	n.a.	n.a.
432.130329	1	$C_{22}H_{23}KN_3O_4$	432.132014	3.9	4.2	12.5	ok	even	1.9	3.3	n.a.	n.a.	n.a.	n.a.
	2	$C_{18}H_{19}KN_9O_2$	432.129329	-2.3	-3.1	13.5	ok	even	10.6	19.8	n.a.	n.a.	n.a.	n.a.
	3	$C_{23}H_{19}KN_7$	432.133352	7.0	6.8	17.5	ok	even	13.2	20.6	n.a.	n.a.	n.a.	n.a.

Compound 3a
Chromatogram

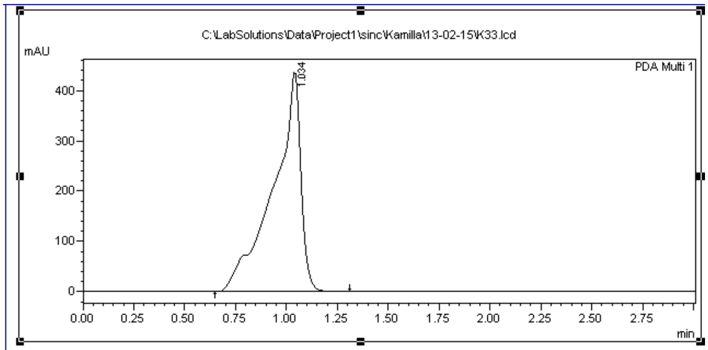

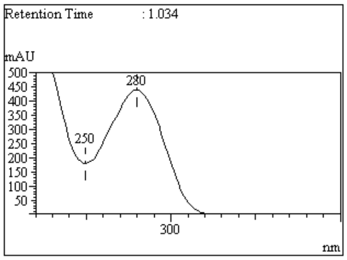

^{1}H NMR

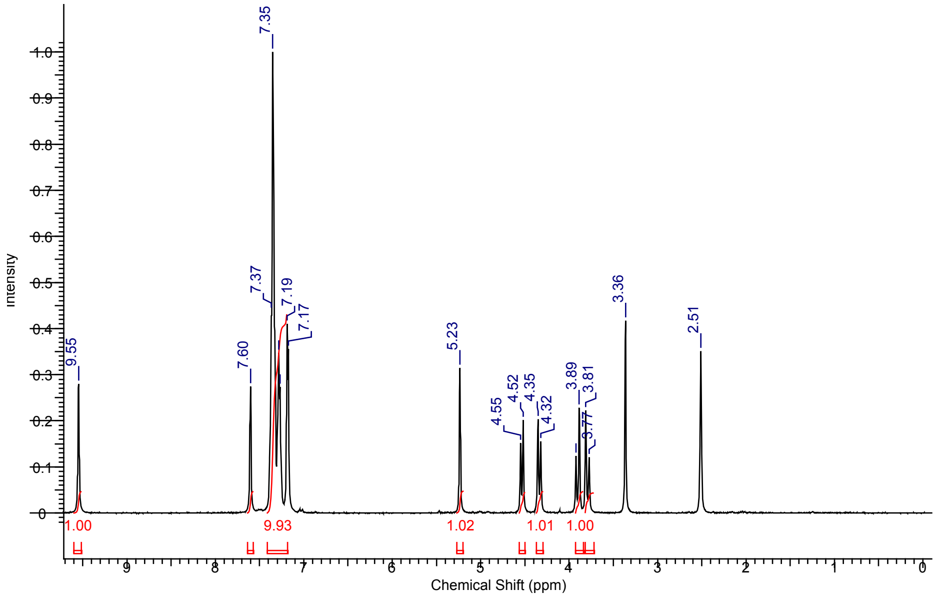

^{13}C NMR

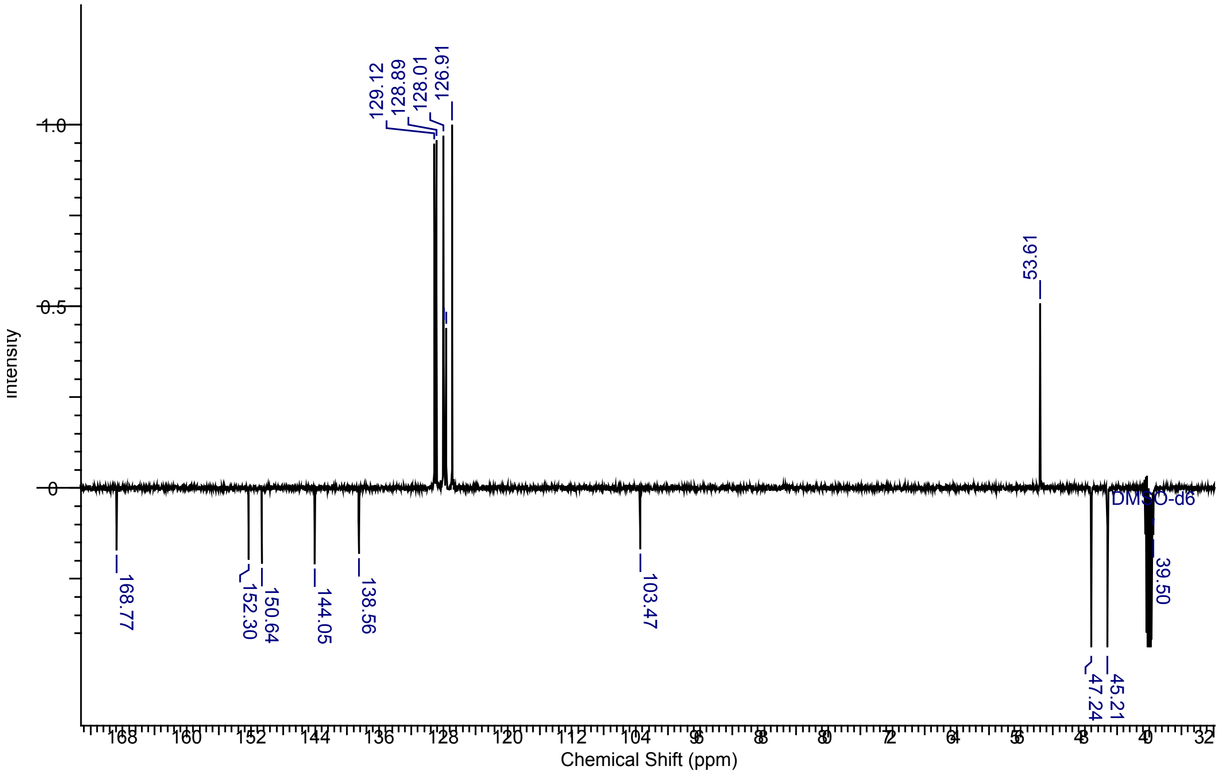

FT-IR

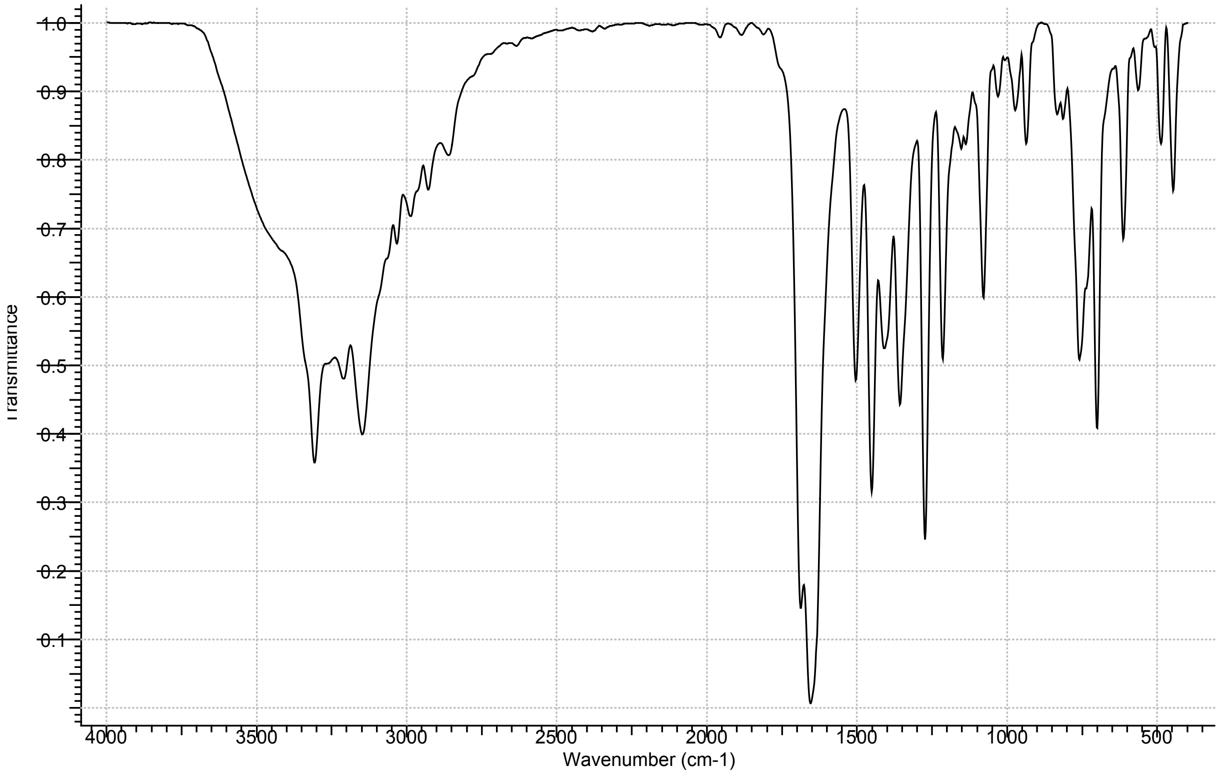

Mass Spectrometry

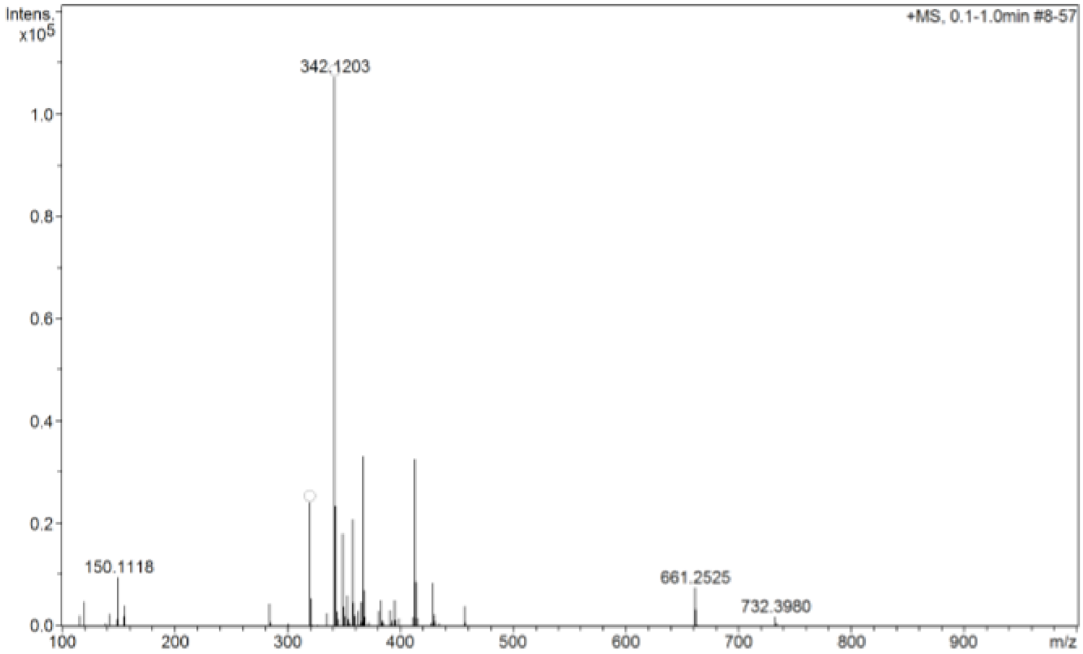

Meas. m/z	#	Ion Formula	m/z	err [ppm]	Mean err [ppm]	rdb	N-Rule	e⁻ Conf	mSigma	Std I	Std Mean m/z	Std I VarNorm	Std m/z Diff	Std Comb Dev
320.138251	1	C19H18N3O2	320.139353	3.4	3.3	12.5	ok	even	10.9	18.5	n.a.	n.a.	n.a.	n.a.
	2	C15H14N9	320.136668	-4.9	-6.5	13.5	ok	even	16.1	23.4	n.a.	n.a.	n.a.	n.a.
	3	C14H18N5O4	320.135330	-9.1	-10.1	8.5	ok	even	29.7	45.0	n.a.	n.a.	n.a.	n.a.
342.120324	1	C19H17N3NaO2	342.121297	2.8	3.0	12.5	ok	even	0.3	0.6	n.a.	n.a.	n.a.	n.a.
	2	C15H13N9Na	342.118612	-5.0	-6.2	13.5	ok	even	13.6	25.2	n.a.	n.a.	n.a.	n.a.
	3	C14H17N5NaO4	342.117275	-8.9	-9.6	8.5	ok	even	26.4	45.5	n.a.	n.a.	n.a.	n.a.

Compound 3b

Chromatogram

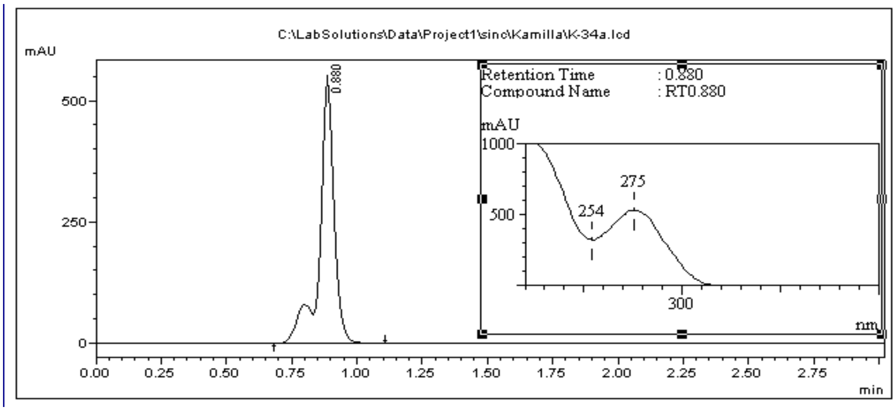

^{1}H NMR

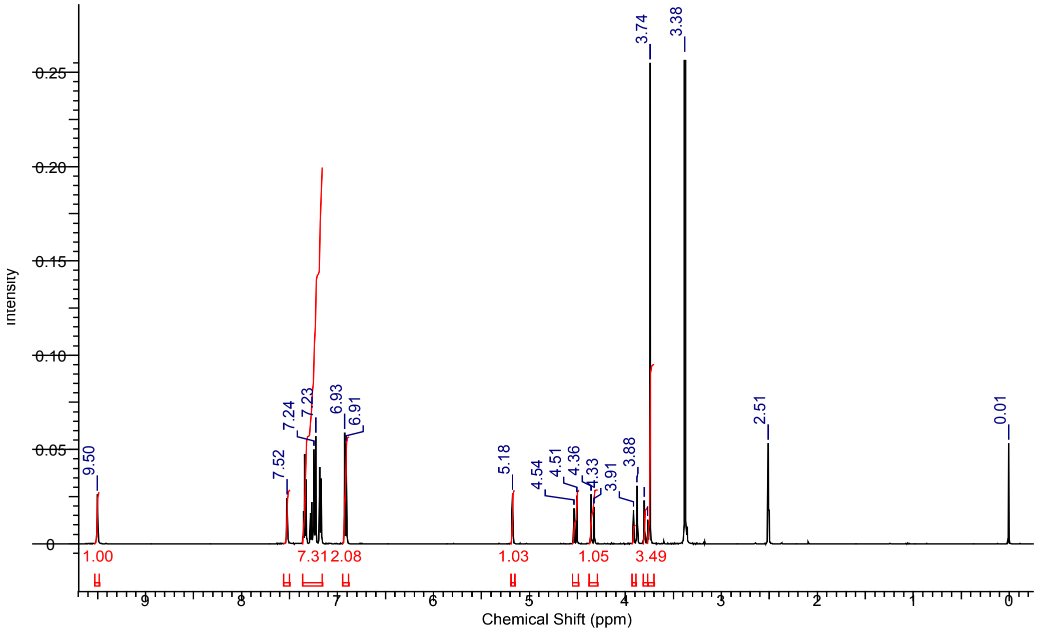

^{13}C NMR

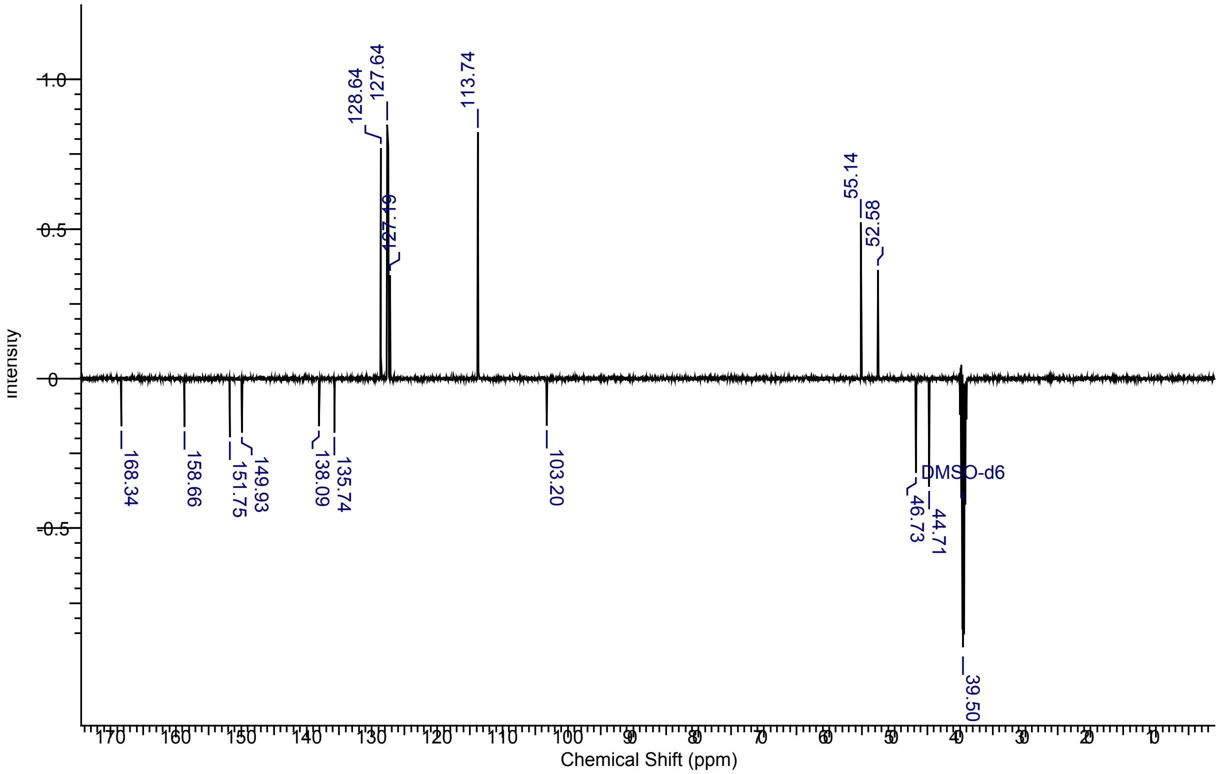

FT-IR

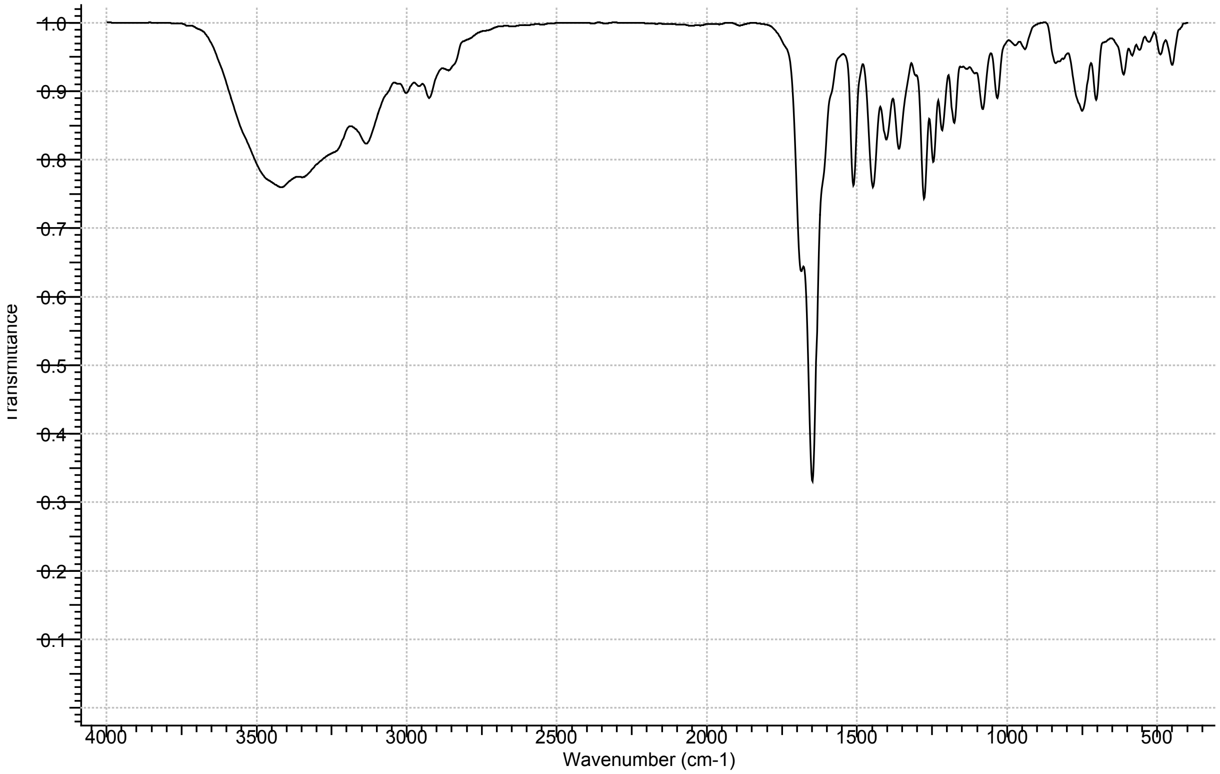

Mass Spectrometry

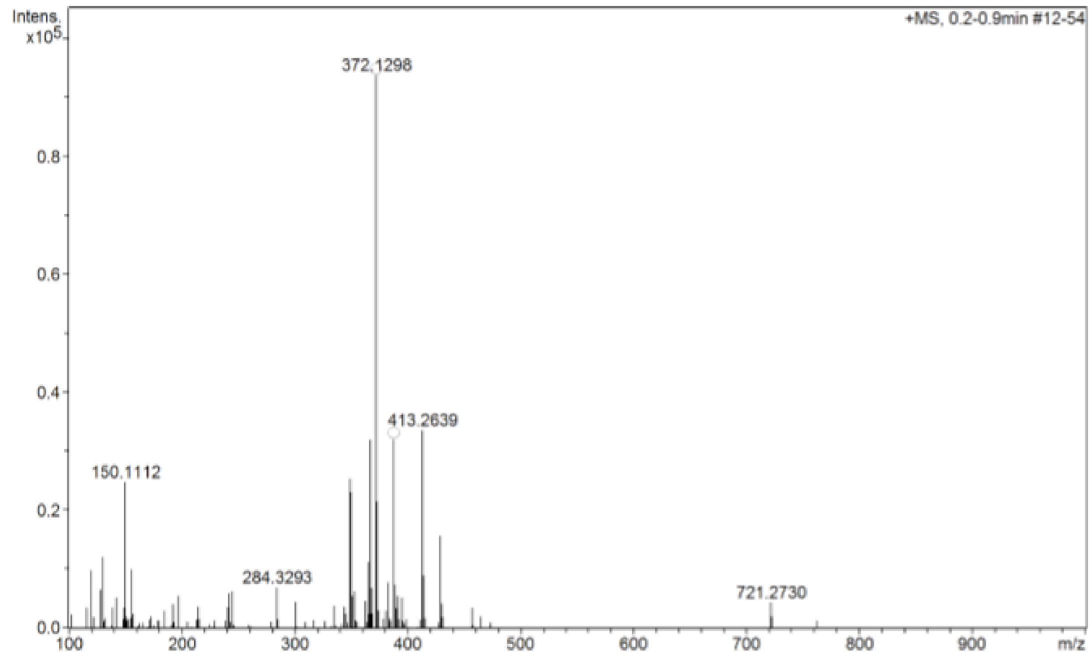

Meas. m/z	#	Ion Formula	m/z	err [ppm]	Mean err [ppm]	rdb	N-Rule	e⁻ Conf	mSigma	Std I	Std Mean m/z	Std I VarNorm	Std m/z Diff	Std Comb Dev
372.129840	1	C20H19N3NaO3	372.131862	5.4	5.0	12.5	ok	even	0.5	0.7	n.a.	n.a.	n.a.	n.a.
	2	C16H15N9NaO	372.129177	-1.8	-3.3	13.5	ok	even	13.9	25.3	n.a.	n.a.	n.a.	n.a.
	3	C15H19N5NaO5	372.127839	-5.4	-6.4	8.5	ok	even	26.5	45.1	n.a.	n.a.	n.a.	n.a.
388.103466	1	C20H19KN3O3	388.105800	6.0	4.1	12.5	ok	even	8.9	16.4	n.a.	n.a.	n.a.	n.a.
	2	C16H15KN9O	388.103114	-0.9	-4.1	13.5	ok	even	14.4	22.0	n.a.	n.a.	n.a.	n.a.
	3	C15H19KN5O5	388.101777	-4.4	-6.9	8.5	ok	even	24.3	39.1	n.a.	n.a.	n.a.	n.a.

Compound 3d

Chromatogram

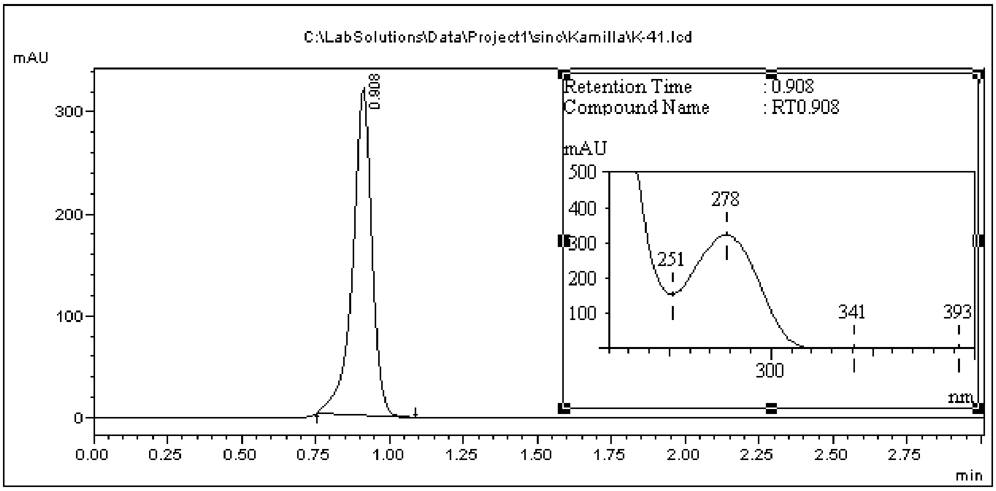

^{1}H NMR

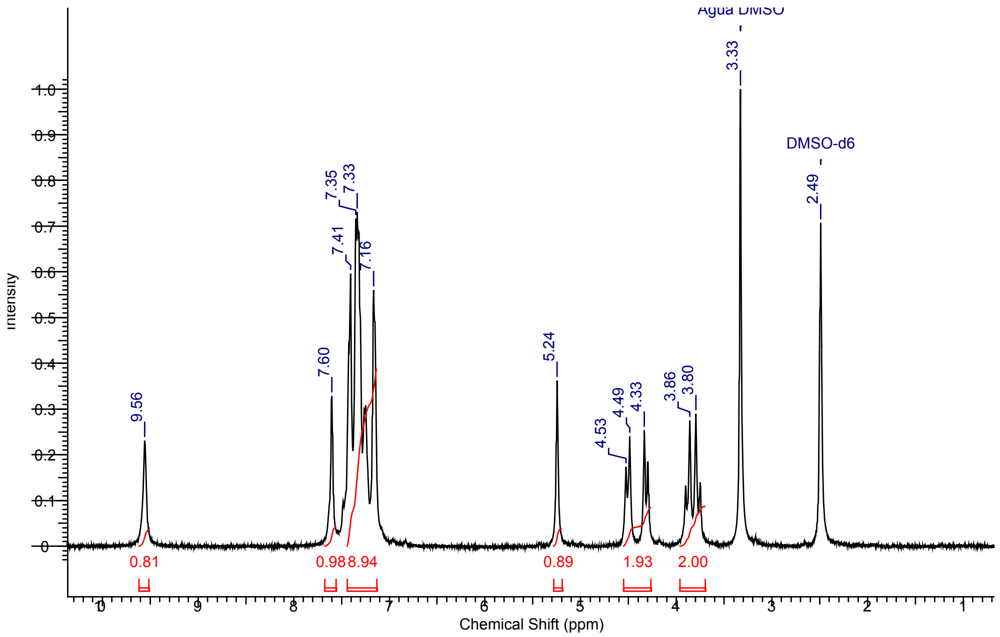
Agua DMSO
3.33
DMSO-d6
2.49
9.56
7.60
7.41
7.35
7.33
7.16
5.24
4.53
4.49
4.33
3.86
3.80
0.81
0.98
8.94
0.89
1.93
2.00
Intensity
Chemical Shift (ppm)

^{13}C NMR

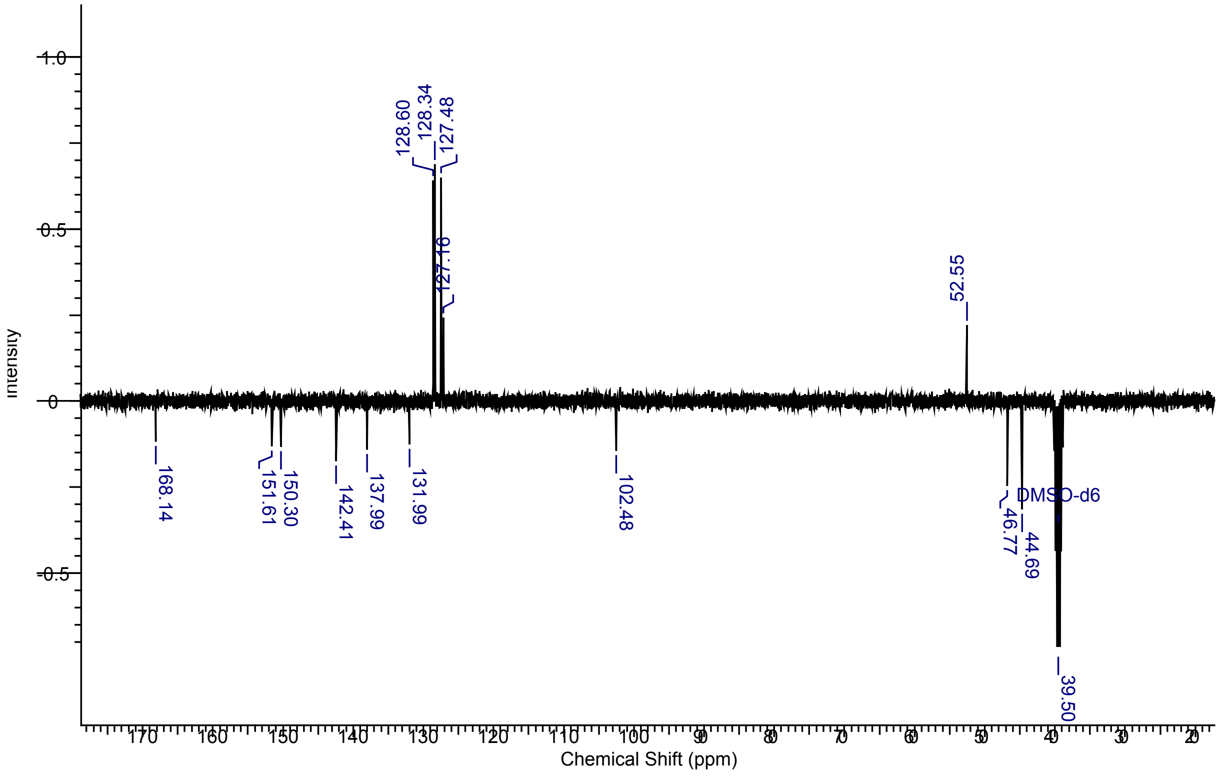
128.60
128.34
127.48
127.16
52.55
168.14
151.61
150.30
142.41
137.99
131.99
102.48
46.77
44.69
DMSO-d6
39.50
Intensity
Chemical Shift (ppm)

FT-IR

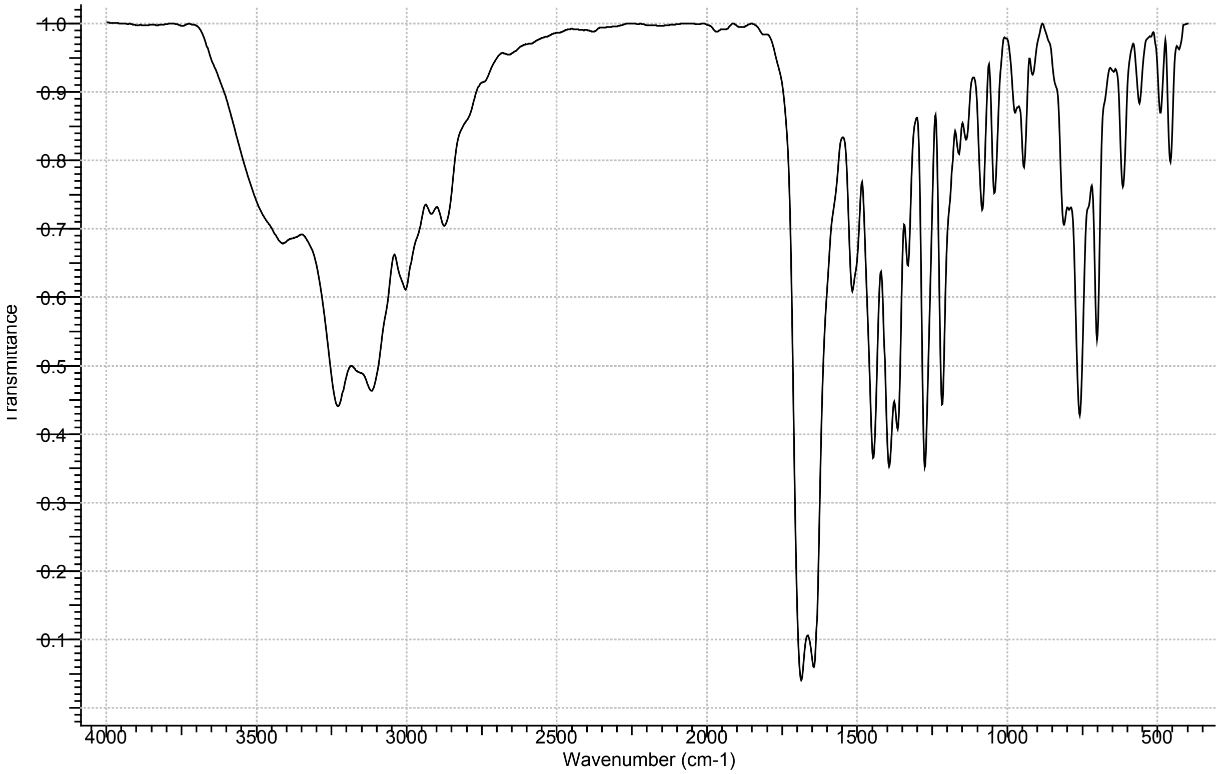

Mass Spectrometry

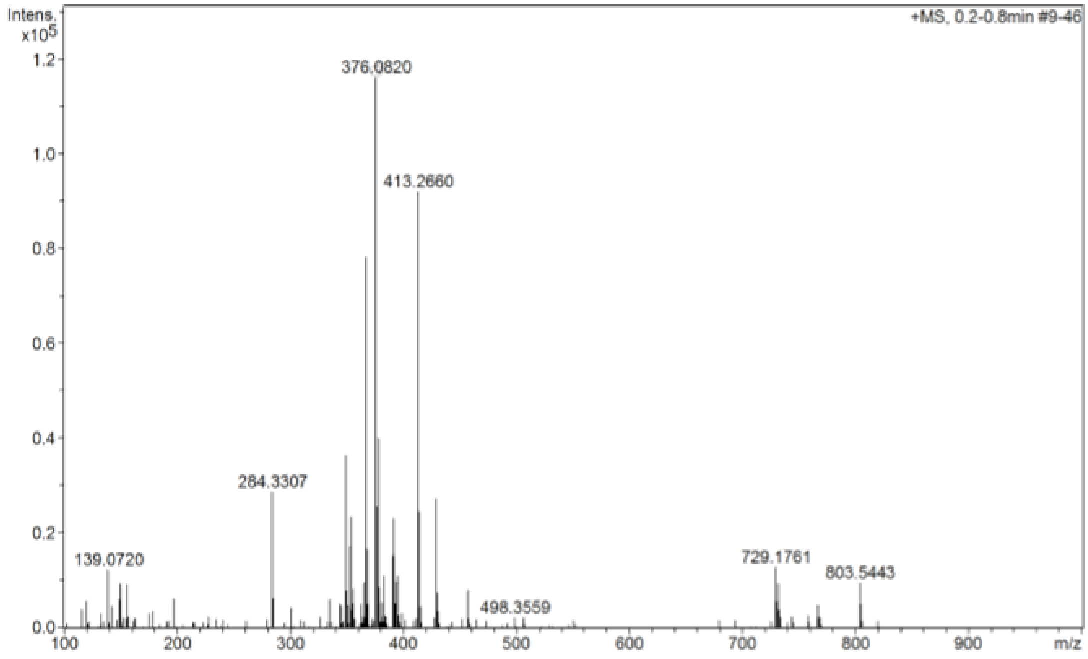

Meas. m/z	#	Ion Formula	m/z	err [ppm]	Mean err [ppm]	rdb	N-Rule	e⁻ Conf	mSigma	Std I	Std Mean m/z	Std I VarNorm	Std m/z Diff	Std Comb Dev
376.082021	1	C19H16ClN3NaO2	376.082325	0.8	-1.2	12.5	ok	even	1.4	1.8	n.a.	n.a.	n.a.	n.a.
	2	C15H12ClN9Na	376.079640	-6.3	-7.3	13.5	ok	even	13.0	19.3	n.a.	n.a.	n.a.	n.a.
	3	C14H16ClN5NaO4	376.078302	-9.9	-10.3	8.5	ok	even	24.8	36.6	n.a.	n.a.	n.a.	n.a.
	4	C14H17Cl2N7Na	376.081470	-1.5	2794.5	8.5	ok	even	137.2	182.5	n.a.	n.a.	n.a.	n.a.
	5	C18H21Cl2NNaO2	376.084155	5.7	2790.8	7.5	ok	even	141.2	188.0	n.a.	n.a.	n.a.	n.a.

Compound 3e

Chromatogram

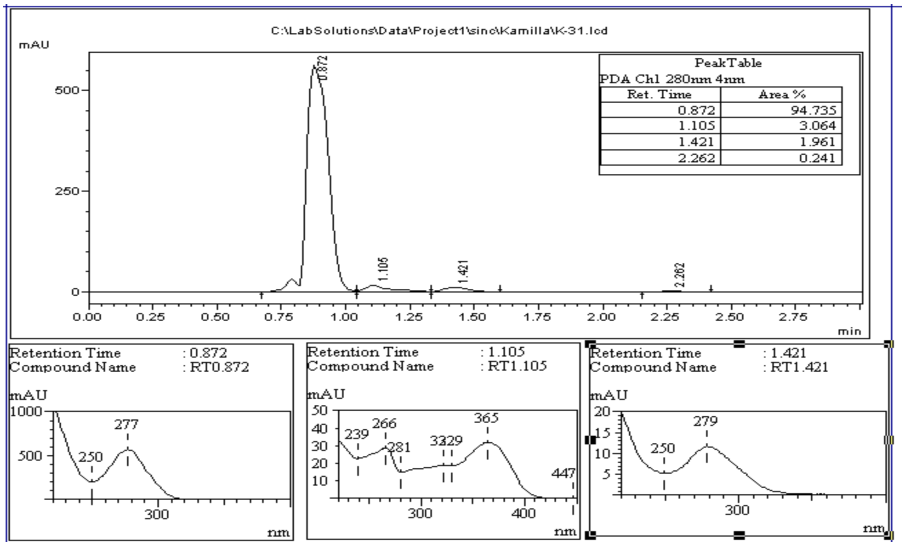

^{1}H NMR

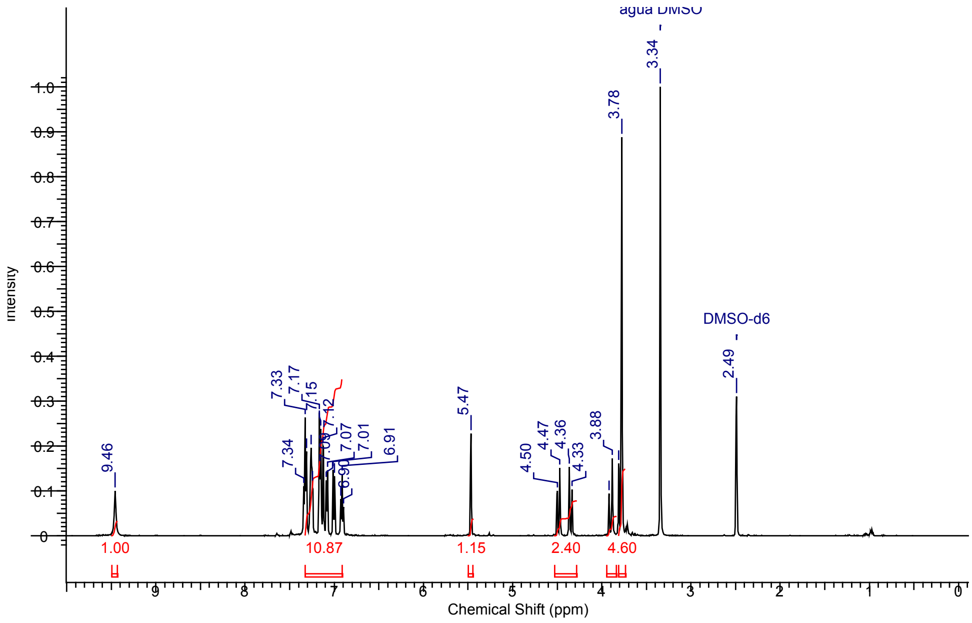

^{13}C NMR

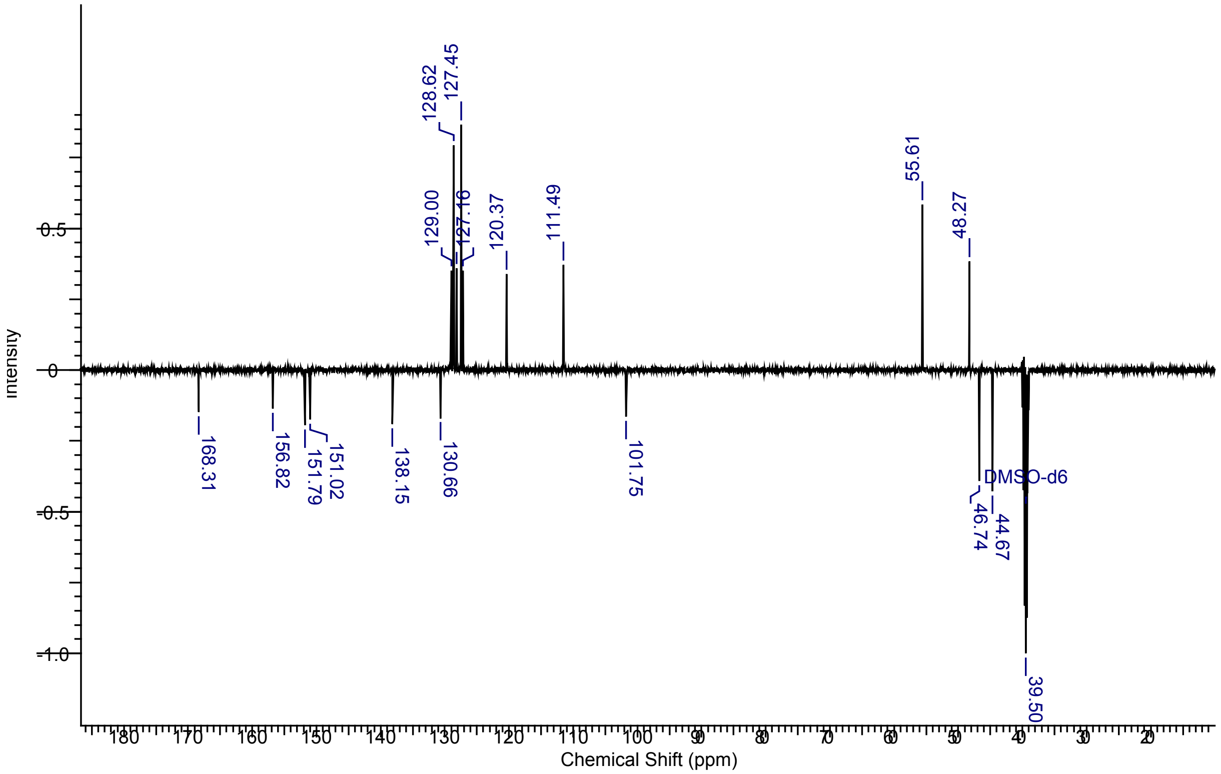

F T - I R

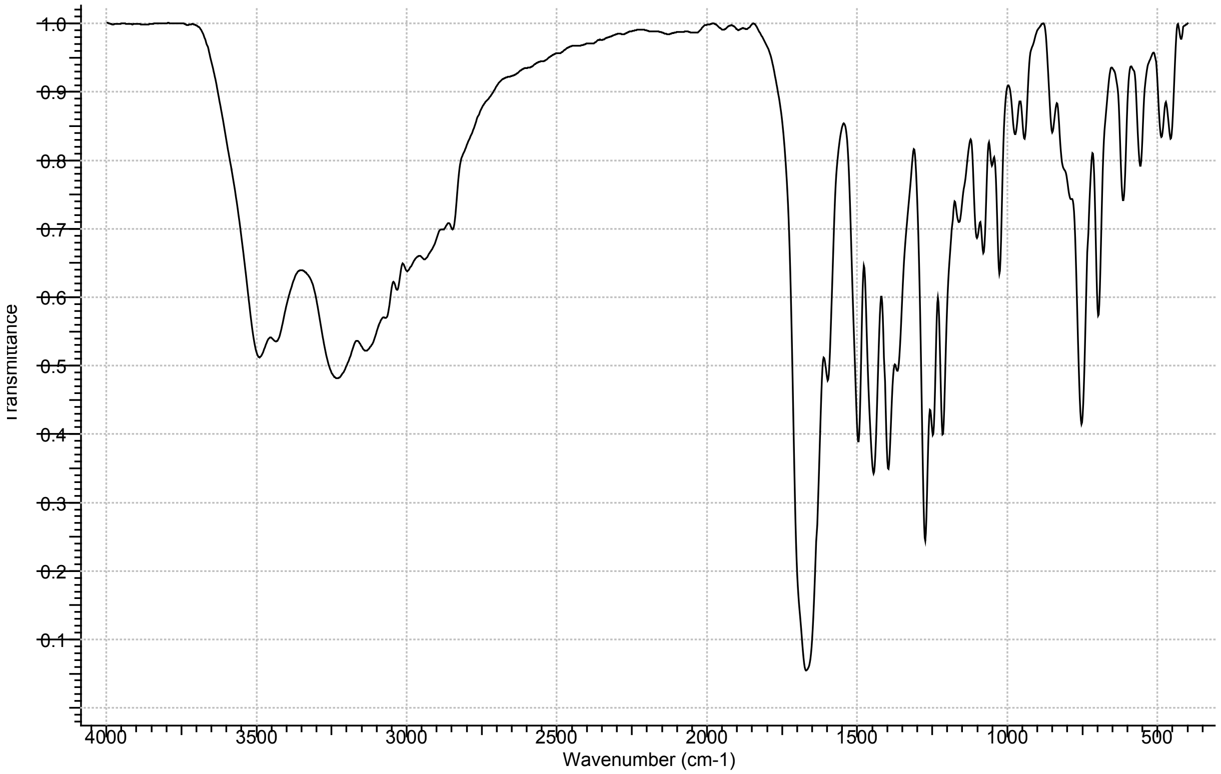

Mass Spectrometry

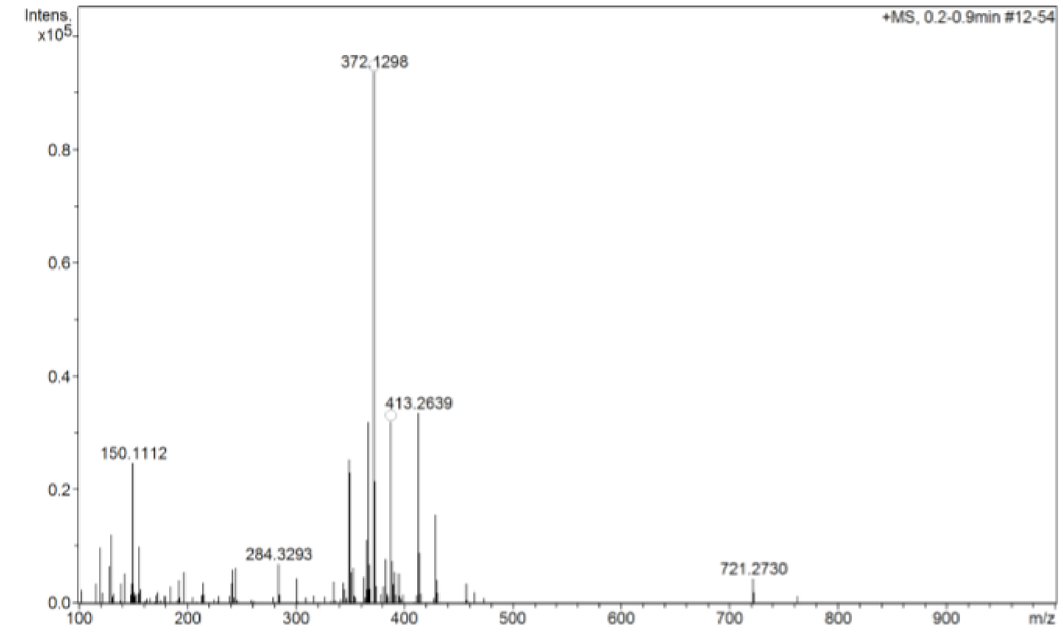

Meas. m/z	#	Ion Formula	m/z	err [ppm]	Mean err [ppm]	rdb	N-Rule	e⁻ Conf	mSigma	Std I	Std Mean m/z	Std I VarNorm	Std m/z Diff	Std Comb Dev
372.129840	1	$C_{20}H_{19}N_3NaO_3$	372.131862	5.4	5.0	12.5	ok	even	0.5	0.7	n.a.	n.a.	n.a.	n.a.
	2	$C_{16}H_{15}N_9NaO$	372.129177	-1.8	-3.3	13.5	ok	even	13.9	25.3	n.a.	n.a.	n.a.	n.a.
	3	$C_{15}H_{19}N_5NaO_5$	372.127839	-5.4	-6.4	8.5	ok	even	26.5	45.1	n.a.	n.a.	n.a.	n.a.
388.103466	1	$C_{20}H_{19}KN_3O_3$	388.105800	6.0	4.1	12.5	ok	even	8.9	16.4	n.a.	n.a.	n.a.	n.a.
	2	$C_{16}H_{15}KN_9O$	388.103114	-0.9	-4.1	13.5	ok	even	14.4	22.0	n.a.	n.a.	n.a.	n.a.
	3	$C_{15}H_{19}KN_5O_5$	388.101777	-4.4	-6.9	8.5	ok	even	24.3	39.1	n.a.	n.a.	n.a.	n.a.

Compound 3f

Chromatogram

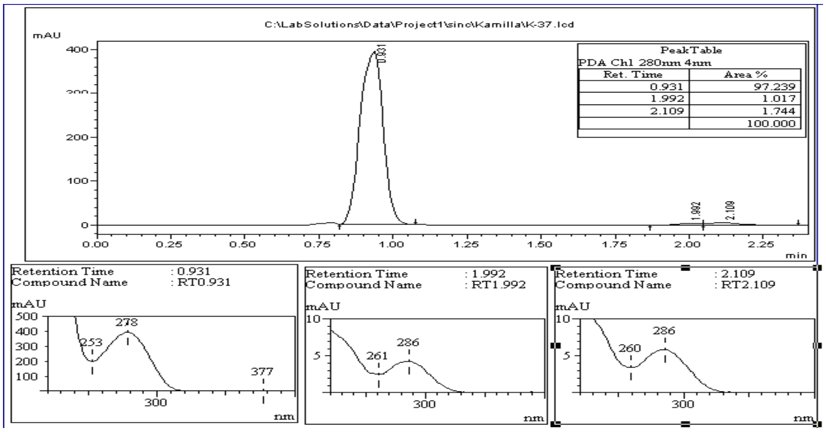

^{1}H NMR

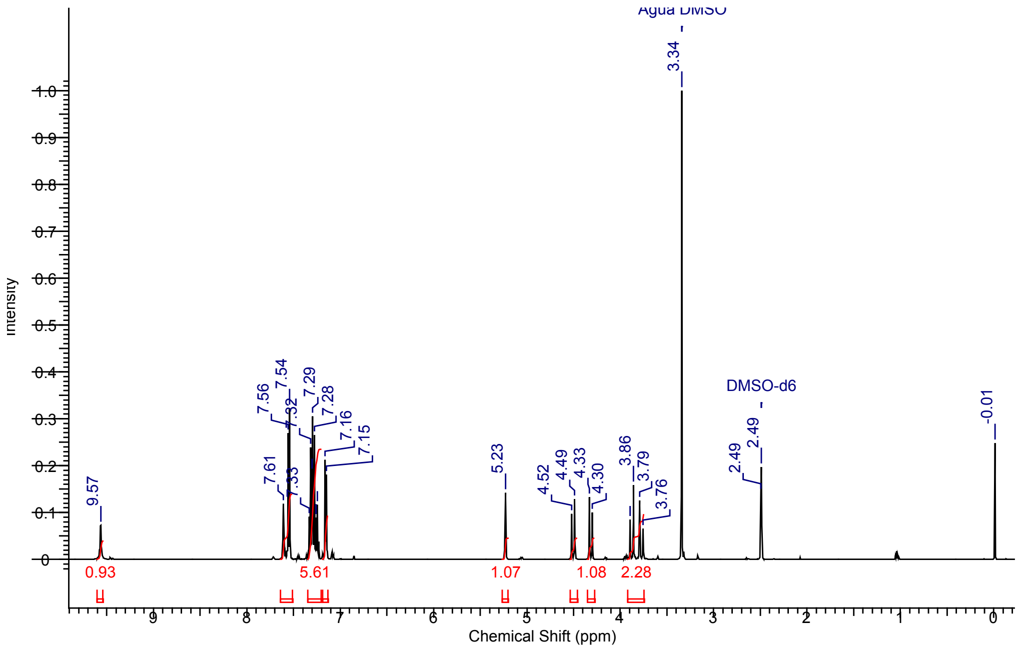
Intensity
Chemical Shift (ppm)
DMSO-d6
9.57
7.61
7.56
7.54
7.33
7.32
7.29
7.28
7.16
7.15
5.23
4.52
4.49
4.33
4.30
3.86
3.79
3.76
3.34
2.49
2.49
-0.01
0.93
5.61
1.07
1.08
2.28

^{13}C NMR

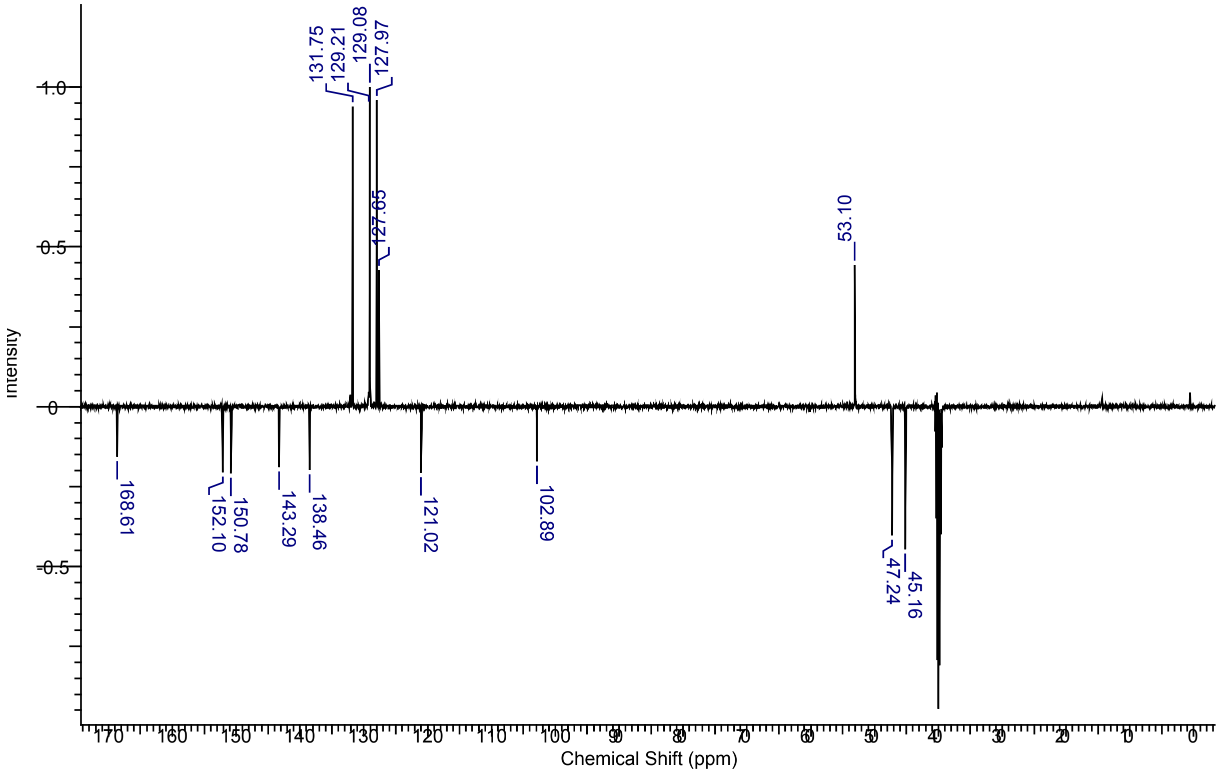
Intensity
Chemical Shift (ppm)
131.75
129.21
129.08
127.97
127.65
53.10
168.61
152.10
150.78
143.29
138.46
121.02
102.89
47.24
45.16

FT-IR

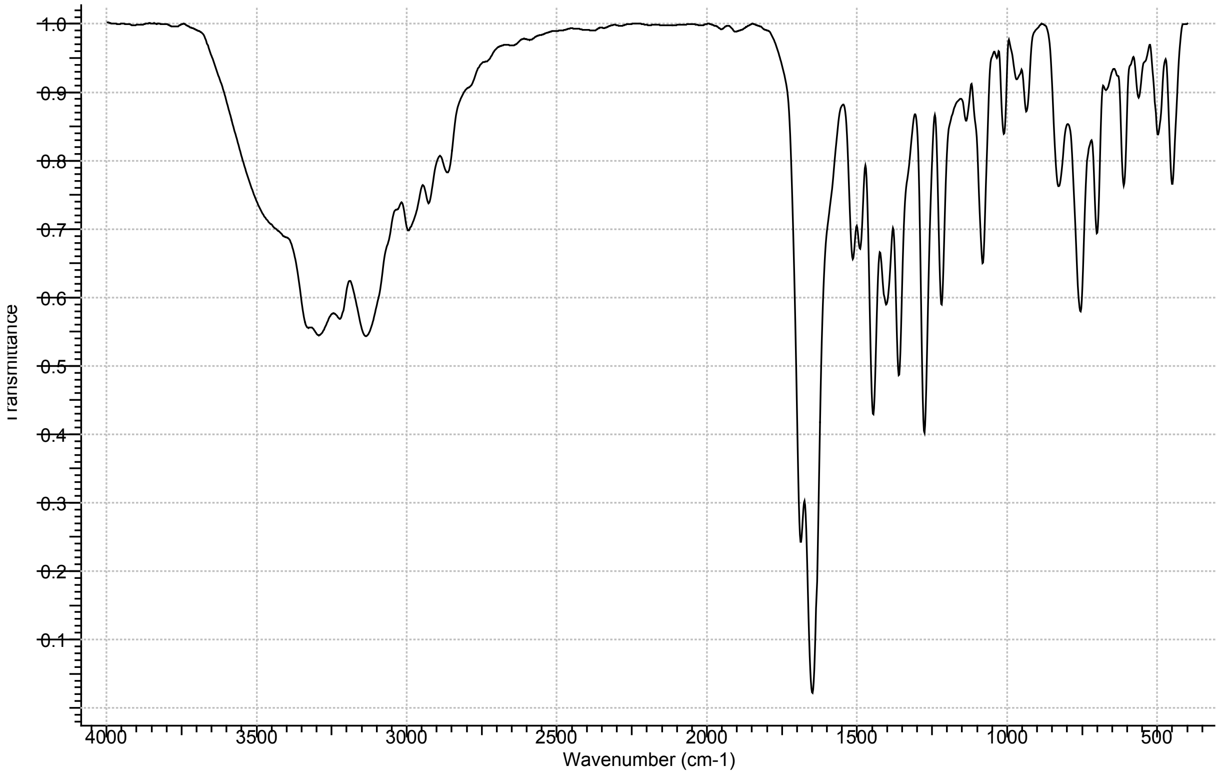

Mass Spectrometry

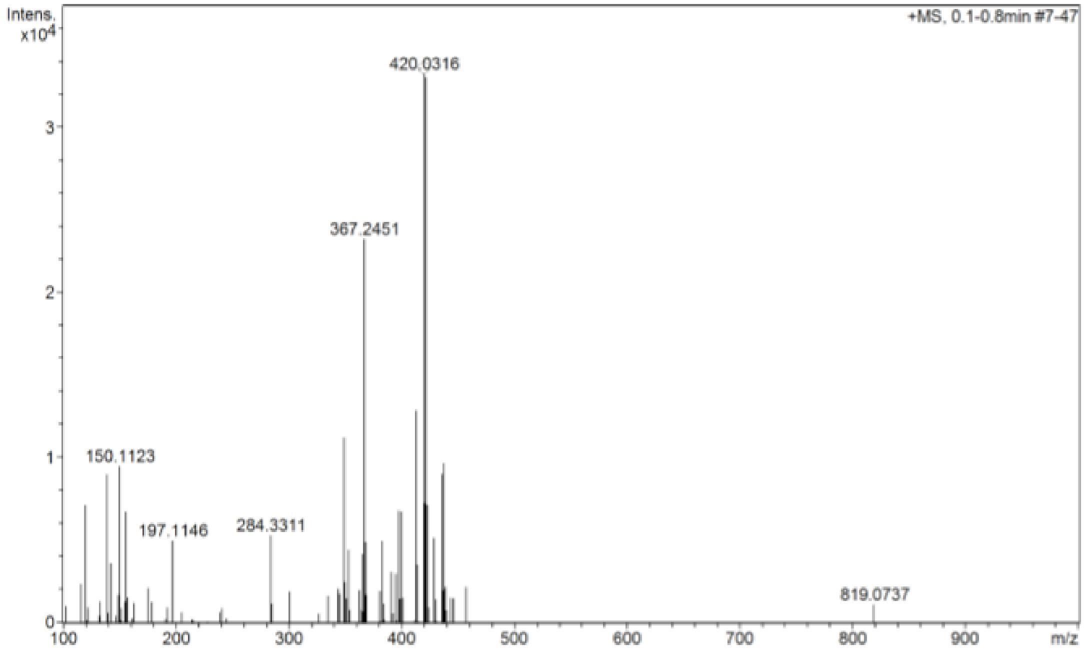

Meas. m/z	#	Ion Formula	m/z	err [ppm]	Mean err [ppm]	rdb	N-Rule	e⁻ Conf	mSigma	Std I	Std Mean m/z	Std I VarNorm	Std m/z Diff	Std Comb Dev
420.031638	1	C19H16BrN3NaO2	420.031810	0.4	-1.0	12.5	ok	even	2.7	1.8	n.a.	n.a.	n.a.	n.a.
	2	C15H12BrN9Na	420.029124	-6.0	-8.3	13.5	ok	even	14.6	16.6	n.a.	n.a.	n.a.	n.a.
	3	C14H16BrN5NaO4	420.027787	-9.2	-11.1	8.5	ok	even	28.3	31.9	n.a.	n.a.	n.a.	n.a.
	4	C8H16BrN9NaO5	420.034997	8.0	5.1	4.5	ok	even	59.8	69.7	n.a.	n.a.	n.a.	n.a.
	5	C7H20BrN5NaO9	420.033660	4.8	2.4	-0.5	ok	even	74.1	87.3	n.a.	n.a.	n.a.	n.a.
	6	C4H12BrN15NaO3	420.032312	1.6	-0.6	5.5	ok	even	82.8	87.2	n.a.	n.a.	n.a.	n.a.

Compound 3g

Chromatogram

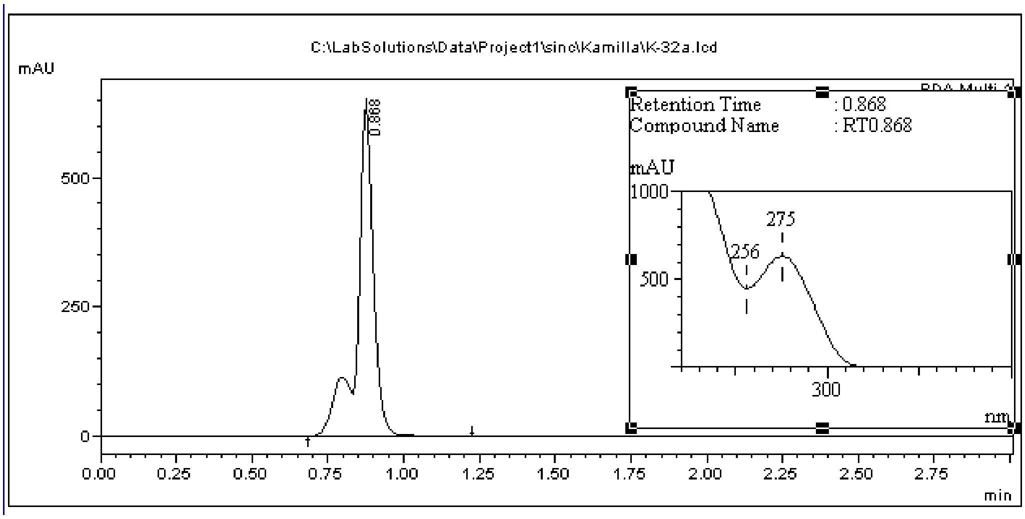

^{1}H NMR

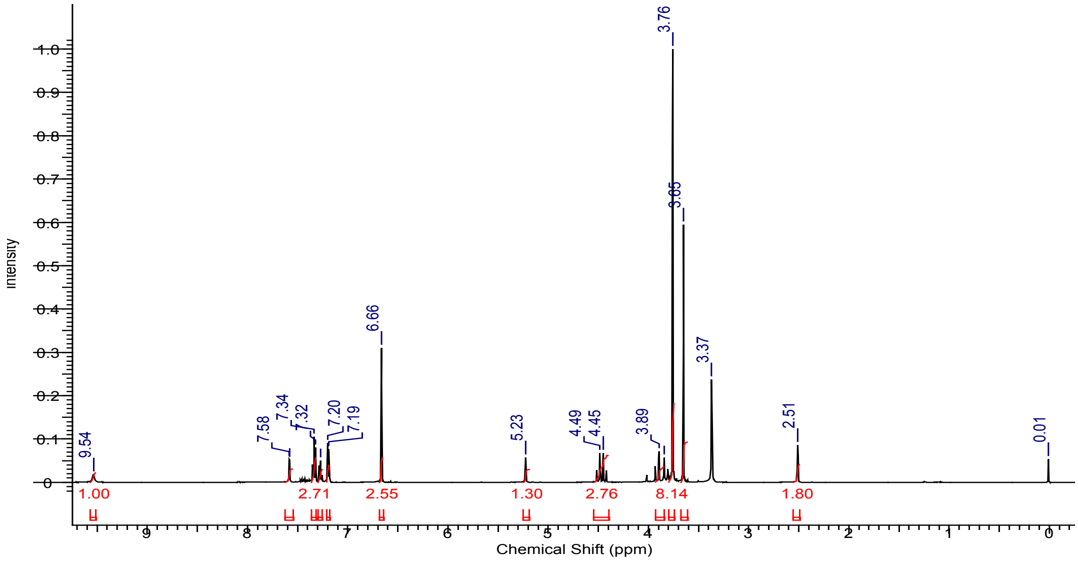

^{13}C NMR

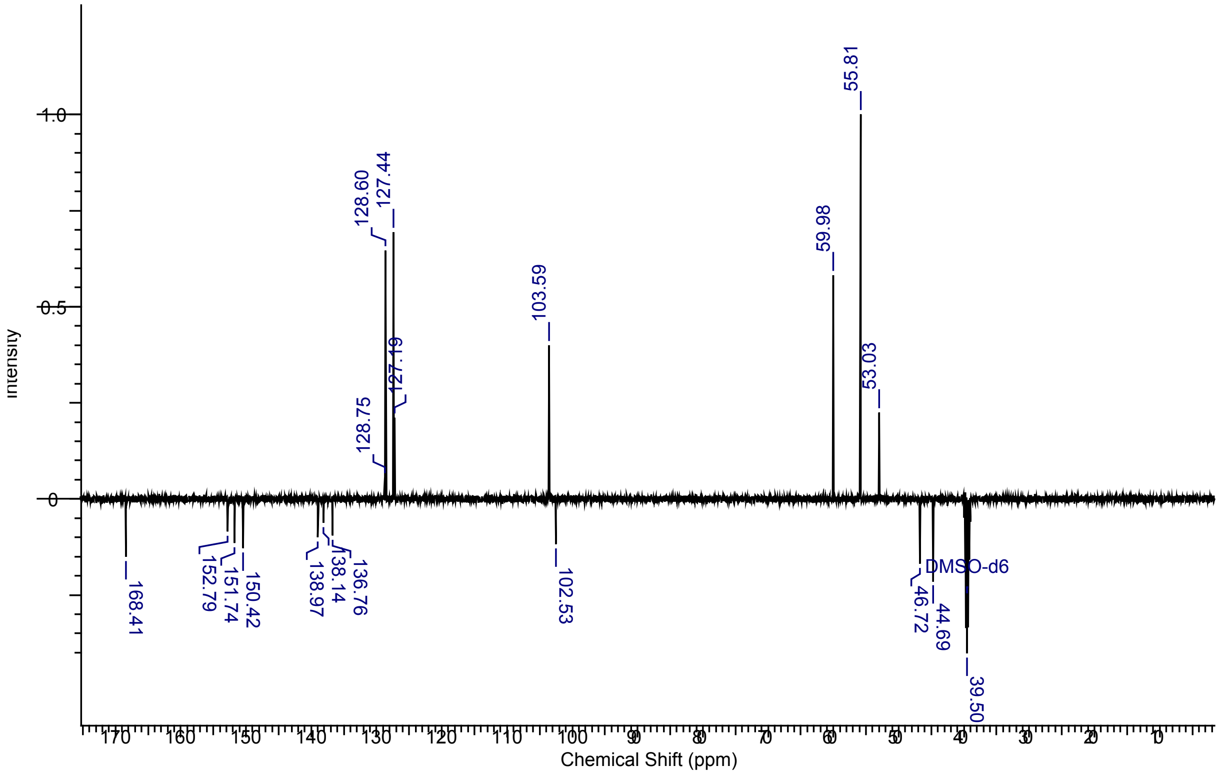

FT-IR

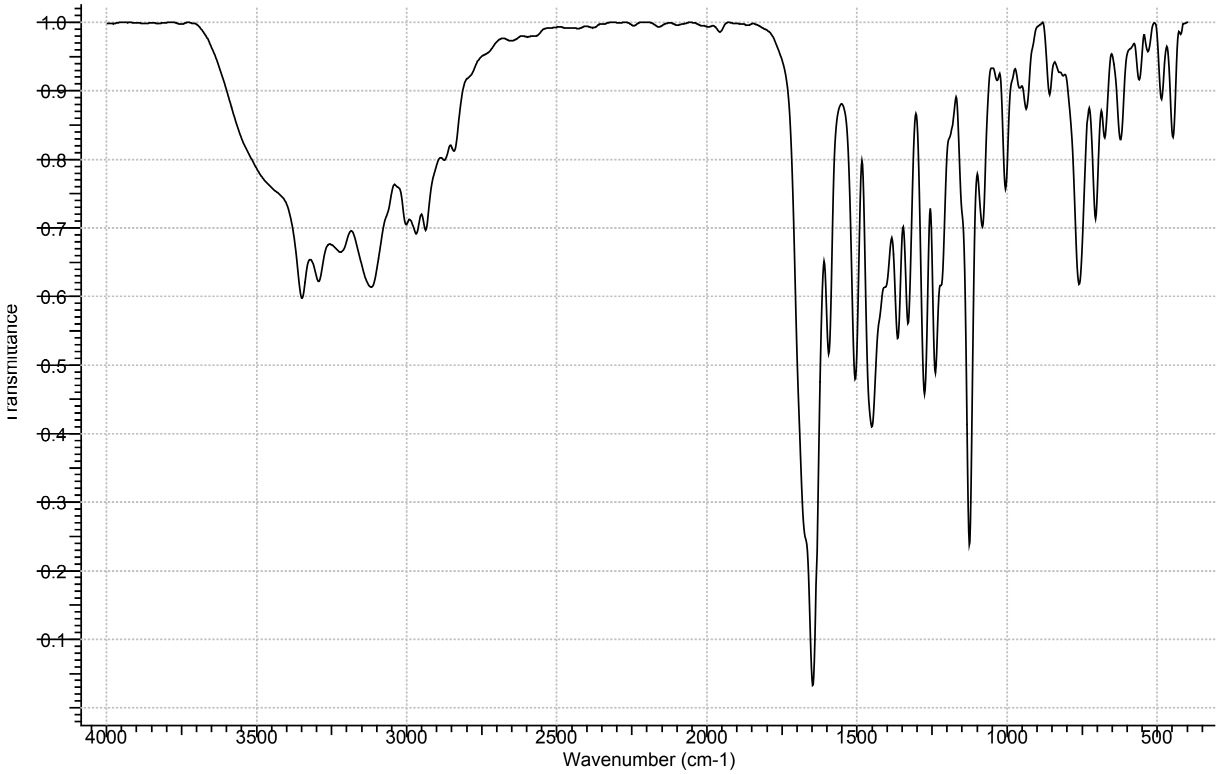

Mass Spectrometry

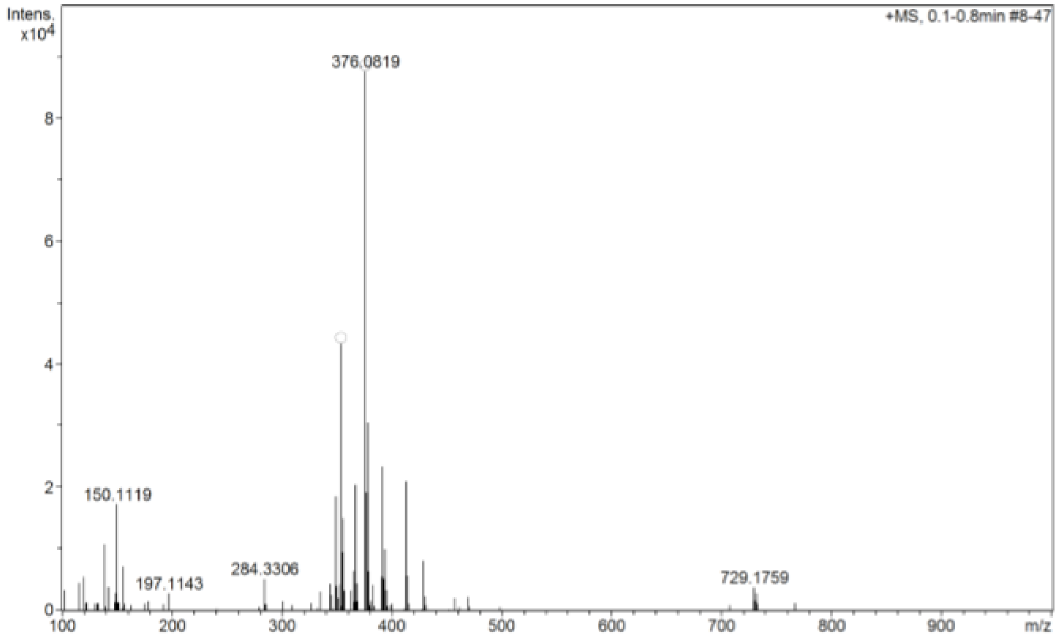

Meas. m/z	#	Ion Formula	m/z	err [ppm]	Mean err [ppm]	rdb	N-Rule	e⁻ Conf	mSigma	Std I	Std Mean m/z	Std I VarNorm	Std m/z Diff	Std Comb Dev
354.099765	1	C19H17ClN3O2	354.100381	1.7	1528.4	12.5	ok	even	4.2	7.0	n.a.	n.a.	n.a.	n.a.
	2	C15H13ClN9	354.097696	-5.8	-7.1	13.5	ok	even	11.7	17.7	n.a.	n.a.	n.a.	n.a.
	3	C14H17ClN5O4	354.096358	-9.6	-10.3	8.5	ok	even	23.3	34.8	n.a.	n.a.	n.a.	n.a.
376.081905	1	C19H16ClN3NaO2	376.082325	1.1	1438.7	12.5	ok	even	4.0	6.2	n.a.	n.a.	n.a.	n.a.
	2	C15H12ClN9Na	376.079640	-6.0	-7.2	13.5	ok	even	13.4	19.9	n.a.	n.a.	n.a.	n.a.
	3	C14H16ClN5NaO4	376.078302	-9.6	-10.3	8.5	ok	even	24.7	36.7	n.a.	n.a.	n.a.	n.a.

Compound 3h

Chromatogram

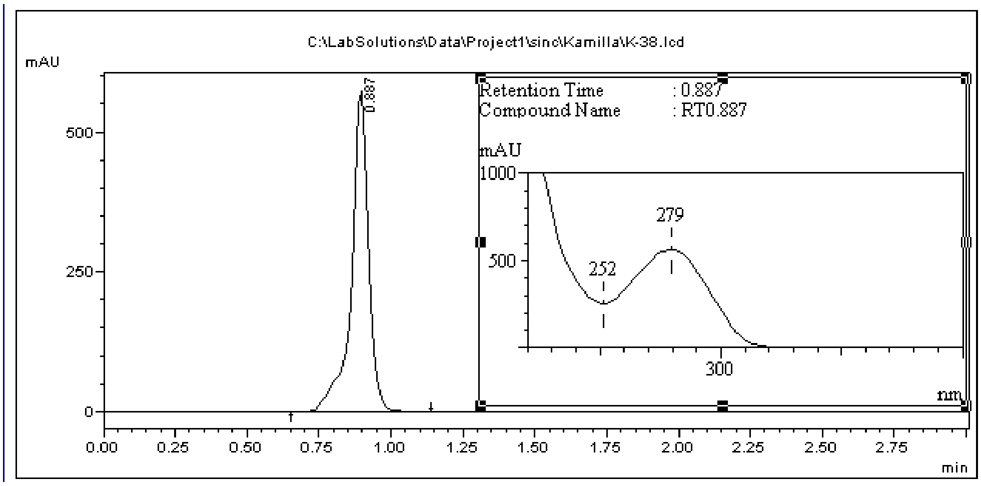

^{1}H NMR

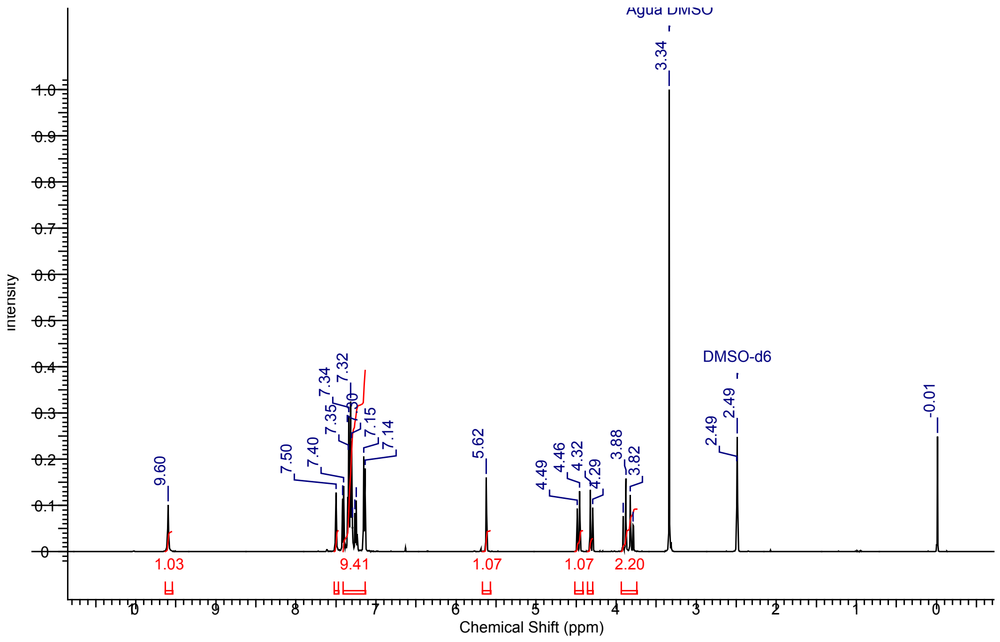
Agua DMSO
3.34
DMSO-d6
2.49
2.49
-0.01
9.60
7.50
7.40
7.35
7.34
7.32
7.30
7.15
7.14
5.62
4.49
4.46
4.32
4.29
3.88
3.82
1.03
9.41
1.07
1.07
2.20
Intensity
Chemical Shift (ppm)

^{13}C NMR

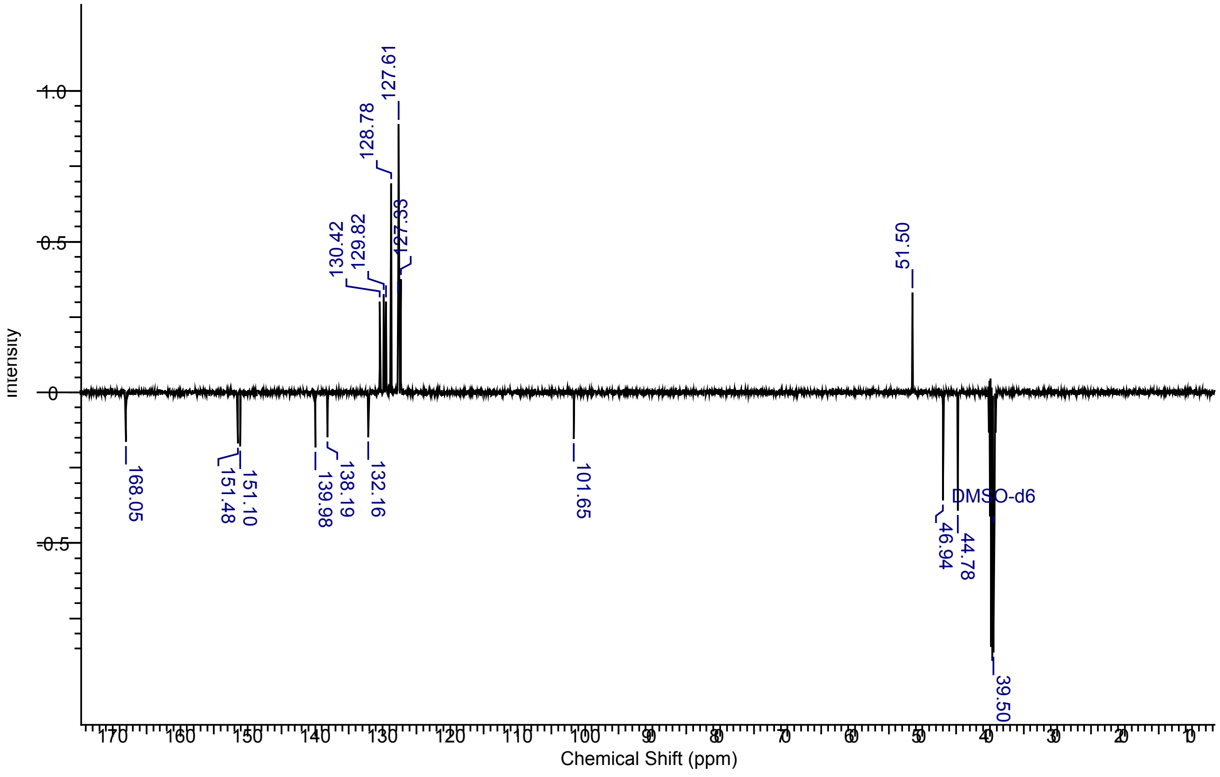
127.61
128.78
127.33
130.42
129.82
51.50
168.05
151.10
151.48
139.98
138.19
132.16
101.65
DMSO-d6
46.94
44.78
39.50
Intensity
Chemical Shift (ppm)

FT-IR

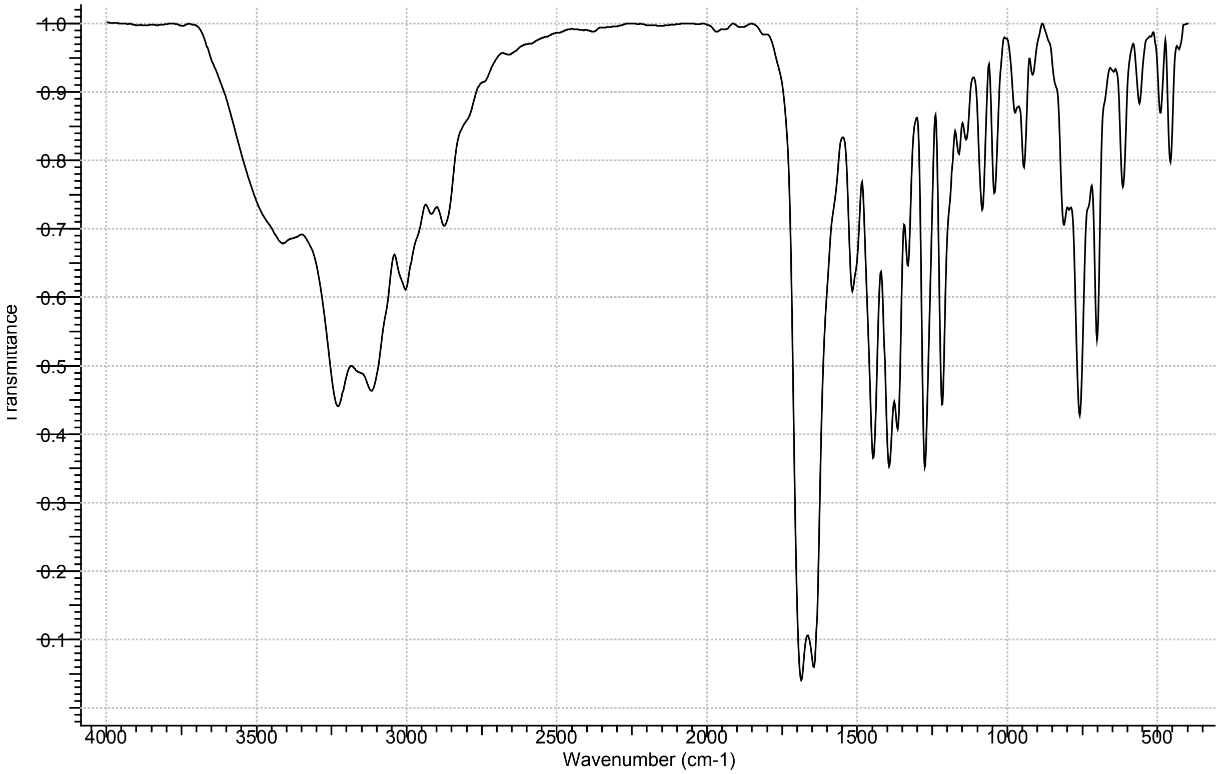

Mass Spectrometry

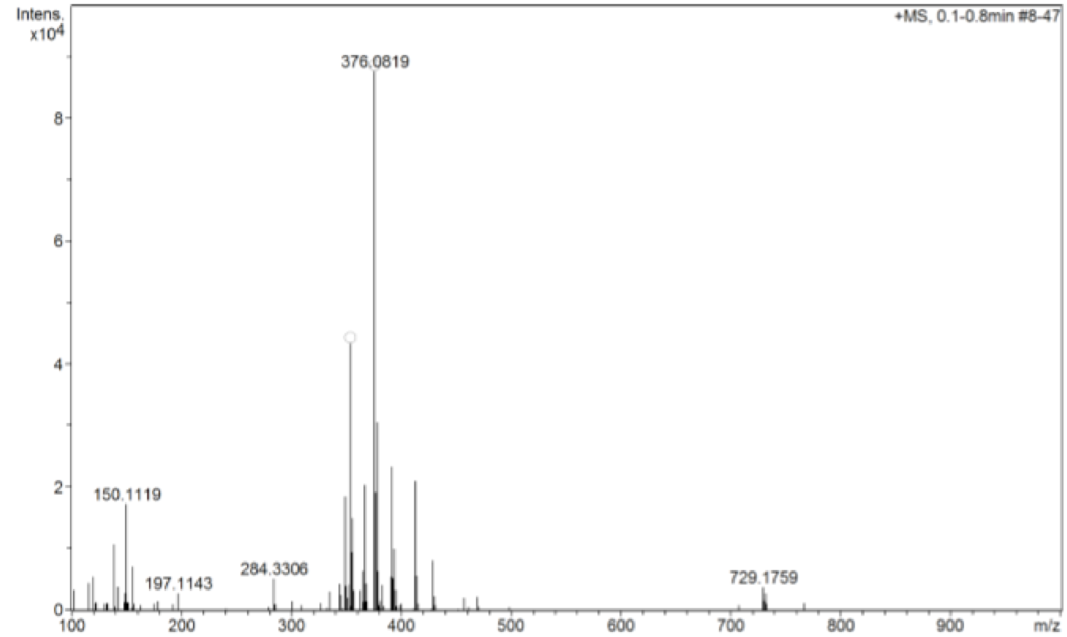

Meas. m/z	#	Ion Formula	m/z	err [ppm]	Mean err [ppm]	rdb	N-Rule	e⁻ Conf	mSigma	Std I	Std Mean m/z	Std I VarNorm	Std m/z Diff	Std Comb Dev
354.099765	1	C19H17ClN3O2	354.100381	1.7	1528.4	12.5	ok	even	4.2	7.0	n.a.	n.a.	n.a.	n.a.
	2	C15H13ClN9	354.097696	-5.8	-7.1	13.5	ok	even	11.7	17.7	n.a.	n.a.	n.a.	n.a.
	3	C14H17ClN5O4	354.096358	-9.6	-10.3	8.5	ok	even	23.3	34.8	n.a.	n.a.	n.a.	n.a.
376.081905	1	C19H16ClN3NaO2	376.082325	1.1	1438.7	12.5	ok	even	4.0	6.2	n.a.	n.a.	n.a.	n.a.
	2	C15H12ClN9Na	376.079640	-6.0	-7.2	13.5	ok	even	13.4	19.9	n.a.	n.a.	n.a.	n.a.
	3	C14H16ClN5NaO4	376.078302	-9.6	-10.3	8.5	ok	even	24.7	36.7	n.a.	n.a.	n.a.	n.a.

Compound 3i

Chromatogram

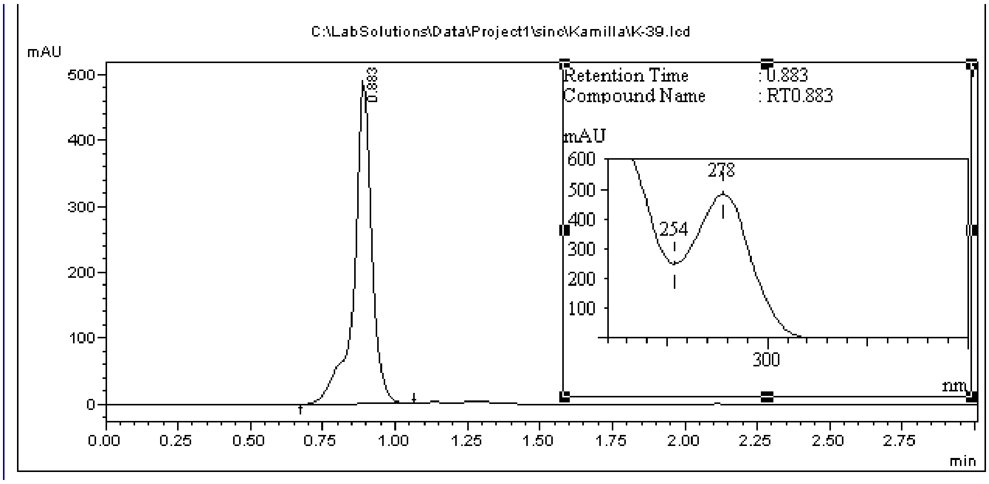

^{1}H NMR

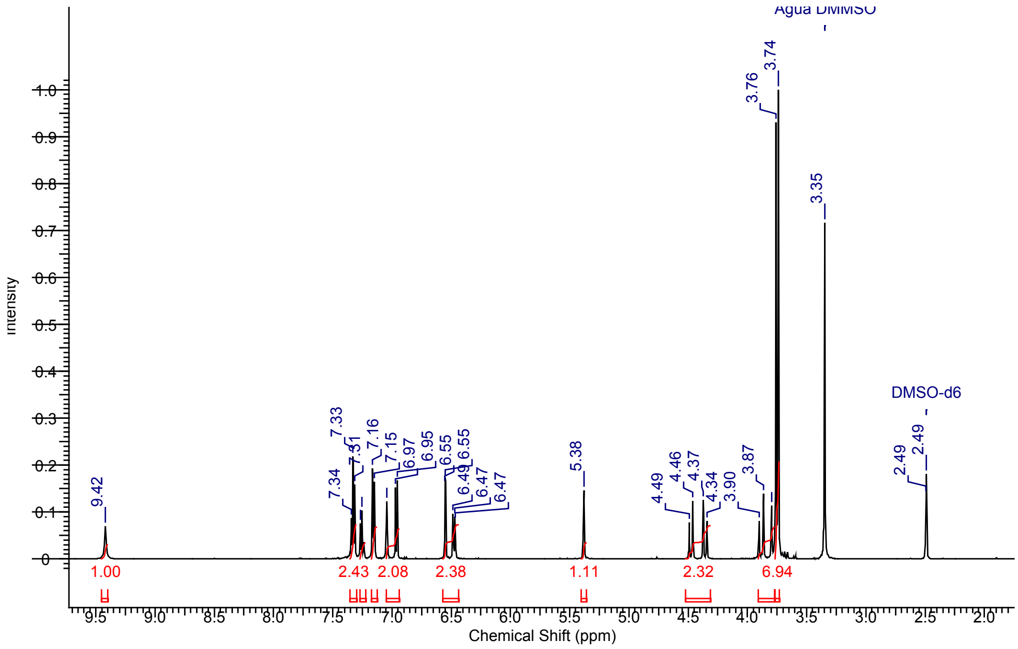

^{13}C NMR

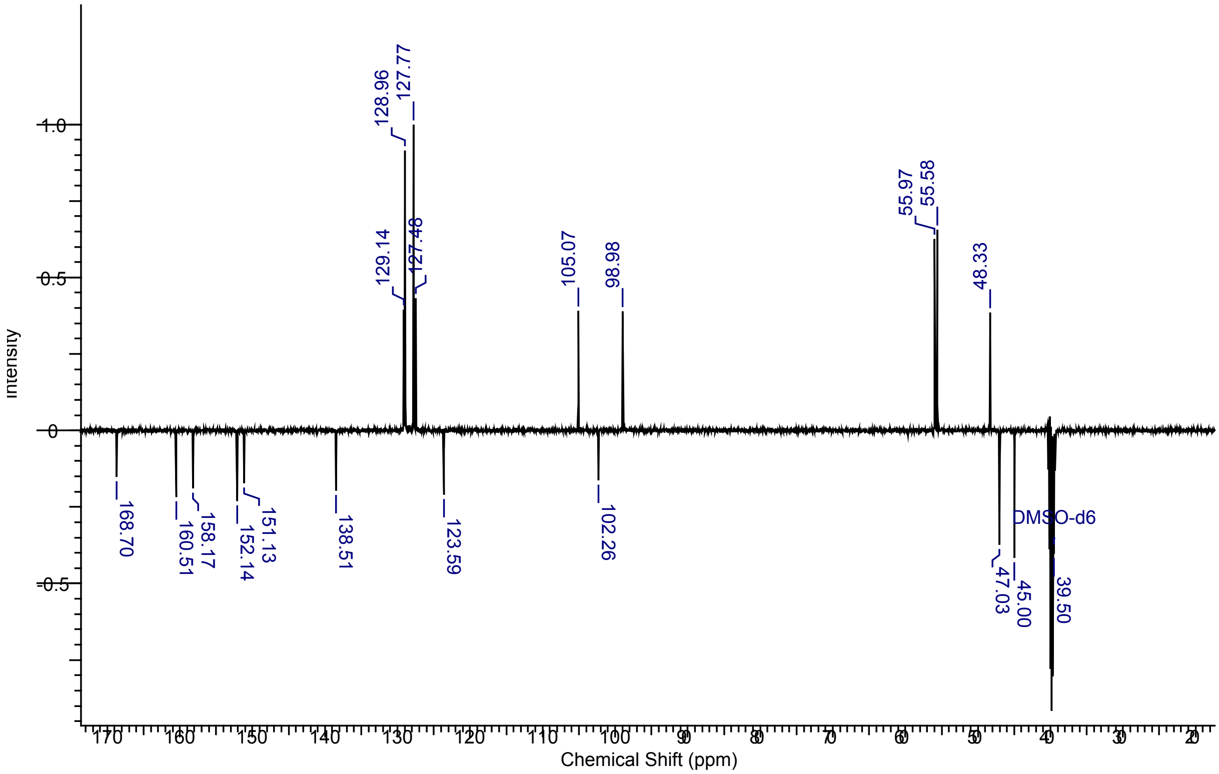

FT-IR

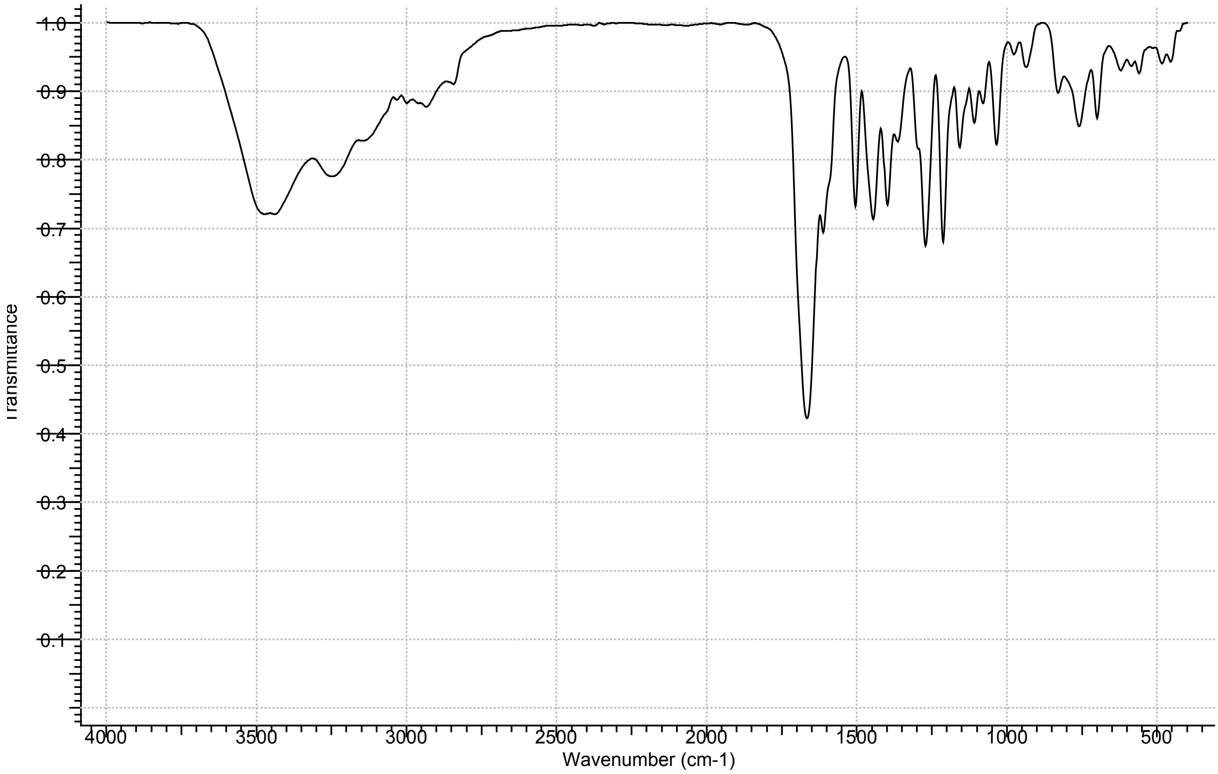

Mass Spectrometry

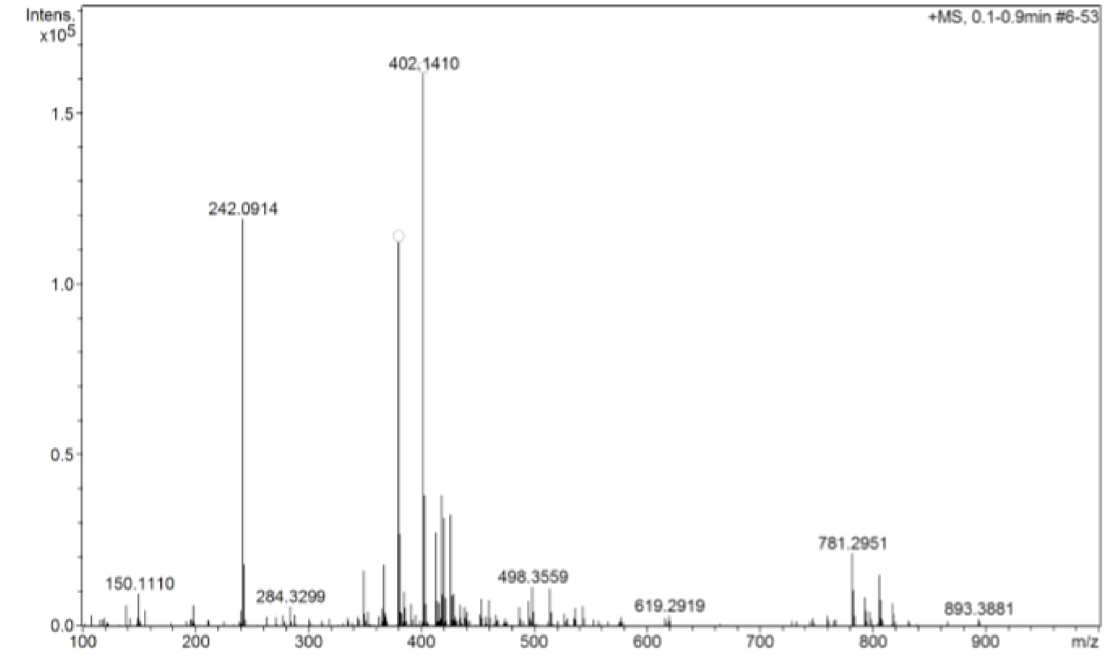

Meas. m/z	#	Ion Formula	m/z	err [ppm]	Mean err [ppm]	rdb	N-Rule	e⁻ Conf	mSigma	Std I	Std Mean m/z	Std I VarNorm	Std m/z Diff	Std Comb Dev
380.159100	1	C21H22N3O4	380.160483	3.6	3.0	12.5	ok	even	2.1	3.0	n.a.	n.a.	n.a.	n.a.
	2	C17H18N9O2	380.157797	-3.4	-5.0	13.5	ok	even	12.4	22.4	n.a.	n.a.	n.a.	n.a.
	3	C22H18N7	380.161820	7.2	6.2	17.5	ok	even	15.7	22.7	n.a.	n.a.	n.a.	n.a.
	4	C16H22N5O6	380.156460	-6.9	-8.1	8.5	ok	even	24.8	41.6	n.a.	n.a.	n.a.	n.a.
	5	C15H26NO10	380.155122	-10.5	-11.3	3.5	ok	even	38.1	62.5	n.a.	n.a.	n.a.	n.a.
402.141021	1	C21H21N3NaO4	402.142427	3.5	-0.1	12.5	ok	even	3.7	4.9	n.a.	n.a.	n.a.	n.a.
	2	C17H17N9NaO2	402.139742	-3.2	-7.7	13.5	ok	even	12.4	23.1	n.a.	n.a.	n.a.	n.a.
	3	C22H17N7Na	402.143764	6.8	3.0	17.5	ok	even	16.9	23.9	n.a.	n.a.	n.a.	n.a.
	4	C16H21N5NaO6	402.138404	-6.5	-10.6	8.5	ok	even	24.1	41.4	n.a.	n.a.	n.a.	n.a.
	5	C15H25NNaO10	402.137067	-9.8	-13.6	3.5	ok	even	37.2	62.0	n.a.	n.a.	n.a.	n.a.

Compound 3j

Chromatogram

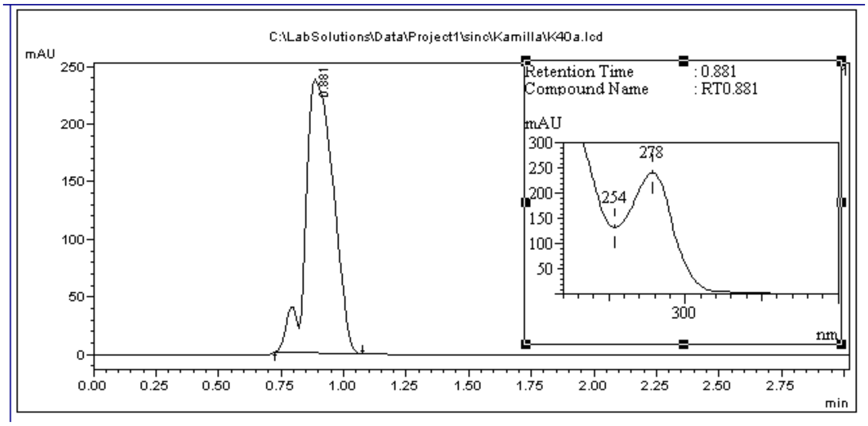

^{1}H NMR

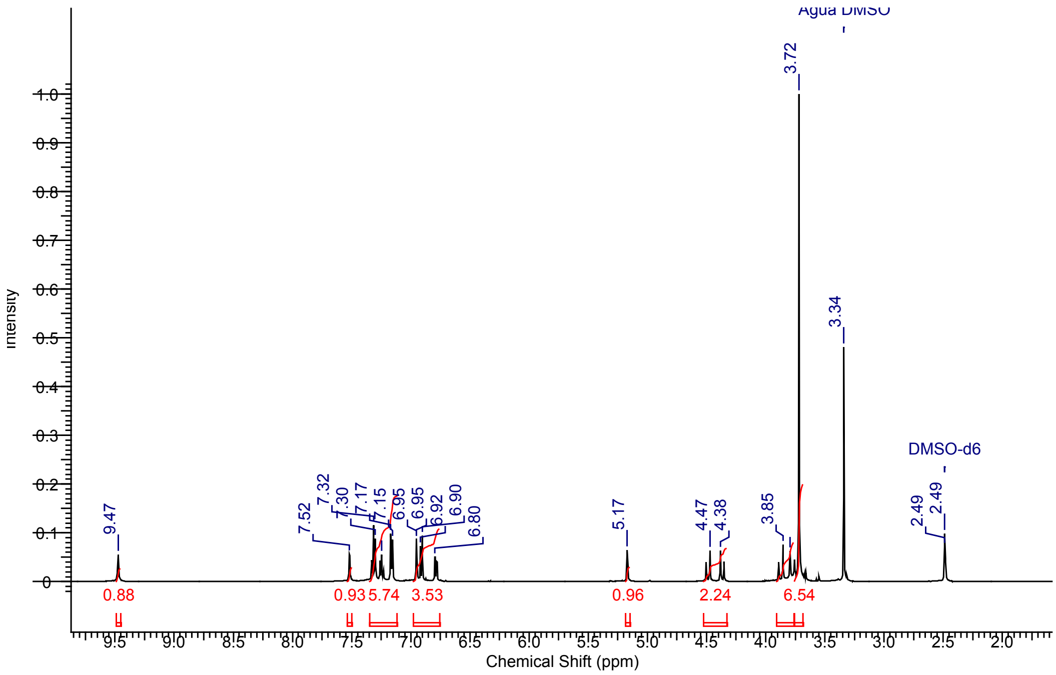

Agua DMSO
3.72
3.34
DMSO-d6
9.47
7.52
7.32
7.30
7.17
7.15
6.95
6.95
6.92
6.90
6.80
5.17
4.47
4.38
3.85
2.49
2.49
0.88
0.93
5.74
3.53
0.96
2.24
6.54
Intensity
Chemical Shift (ppm)

^{13}C NMR

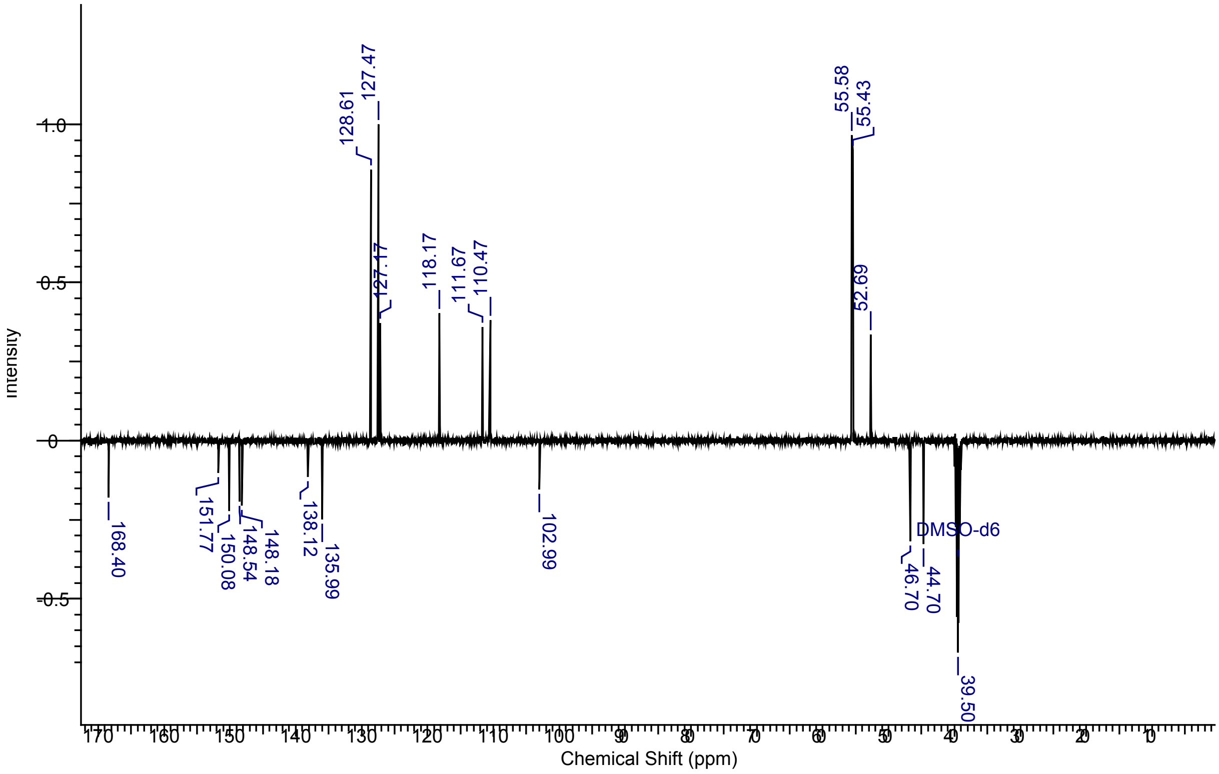

128.61
127.47
127.17
118.17
111.67
110.47
55.58
55.43
52.69
168.40
151.77
150.08
148.54
148.18
138.12
135.99
102.99
DMSO-d6
46.70
44.70
39.50
Intensity
Chemical Shift (ppm)

FT-IR

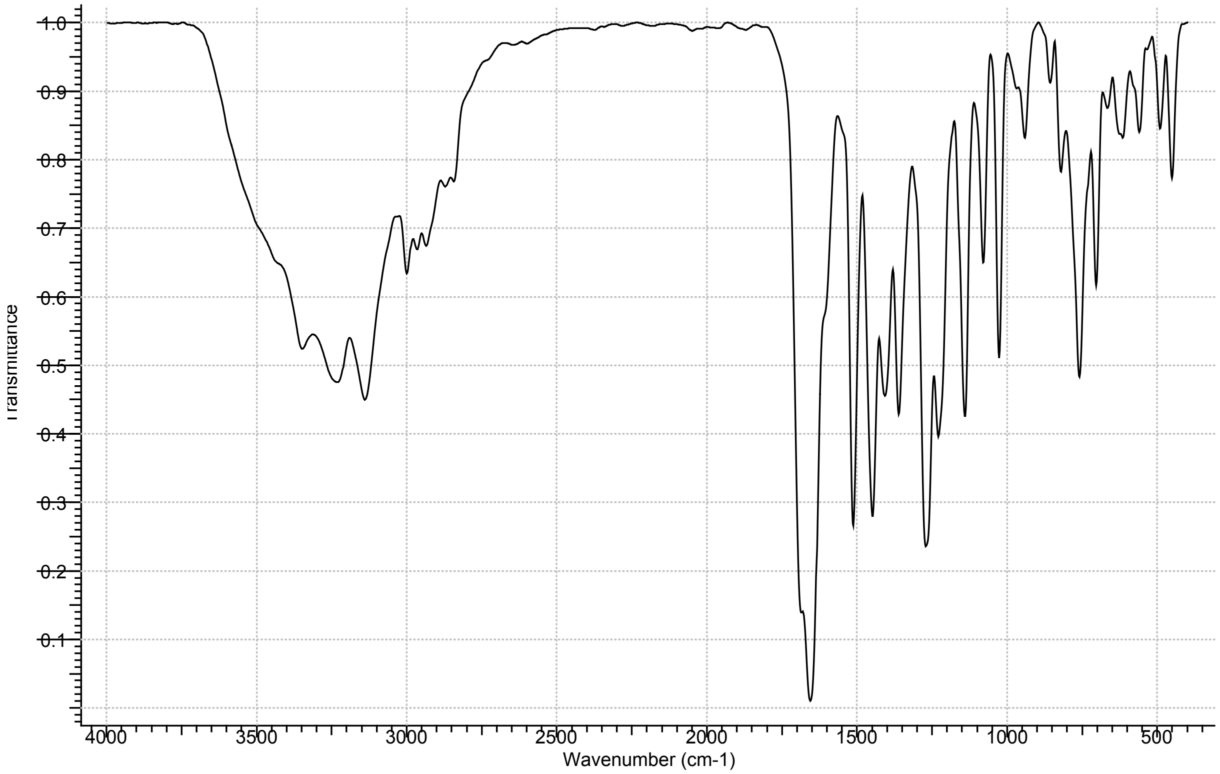

Mass Spectrometry

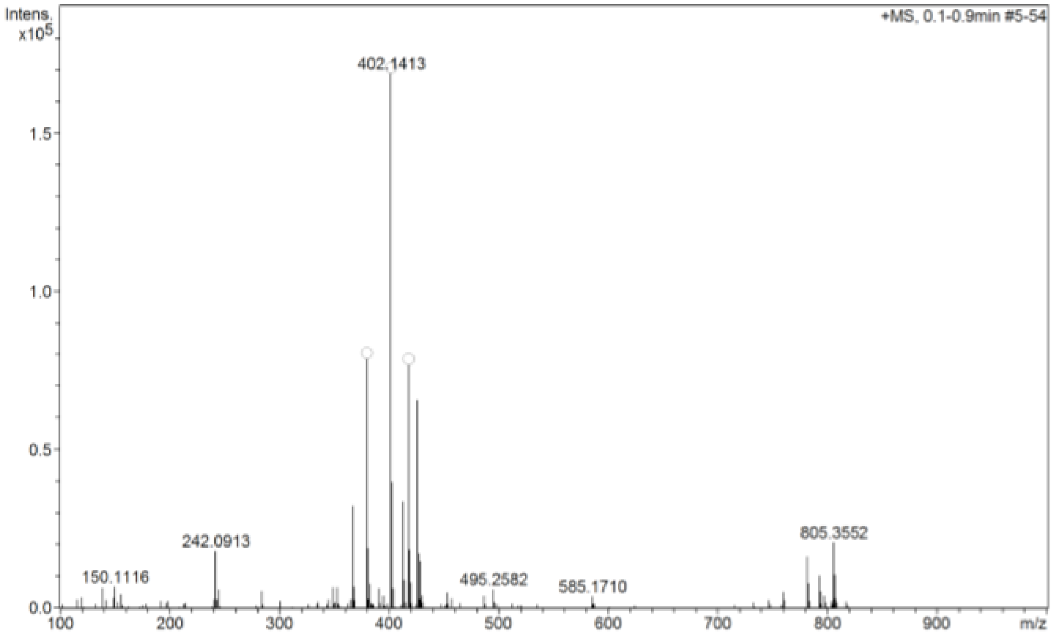

Meas. m/z	#	Ion Formula	m/z	err [ppm]	Mean err [ppm]	rdb	N-Rule	e⁻ Conf	mSigma	Std I	Std Mean m/z	Std I VarNorm	Std m/z Diff	Std Comb Dev
380.159340	1	C21H22N3O4	380.160483	3.0	3.0	12.5	ok	even	0.6	1.0	n.a.	n.a.	n.a.	n.a.
	2	C17H18N9O2	380.157797	-4.1	-5.0	13.5	ok	even	13.5	23.9	n.a.	n.a.	n.a.	n.a.
	3	C22H18N7	380.161820	6.5	6.2	17.5	ok	even	14.2	20.7	n.a.	n.a.	n.a.	n.a.
	4	C16H22N5O6	380.156460	-7.6	-8.1	8.5	ok	even	26.1	43.4	n.a.	n.a.	n.a.	n.a.
402.141323	1	C21H21N3NaO4	402.142427	2.7	1.0	12.5	ok	even	3.2	4.6	n.a.	n.a.	n.a.	n.a.
	2	C17H17N9NaO2	402.139742	-3.9	-6.6	13.5	ok	even	11.5	21.0	n.a.	n.a.	n.a.	n.a.
	3	C22H17N7Na	402.143764	6.1	4.1	17.5	ok	even	16.8	24.3	n.a.	n.a.	n.a.	n.a.
	4	C16H21N5NaO6	402.138404	-7.3	-9.5	8.5	ok	even	23.8	40.1	n.a.	n.a.	n.a.	n.a.
418.115139	1	C21H21KN3O4	418.116364	2.9	3.2	12.5	ok	even	2.1	3.9	n.a.	n.a.	n.a.	n.a.
	2	C17H17KN9O2	418.113679	-3.5	-4.3	13.5	ok	even	10.6	19.8	n.a.	n.a.	n.a.	n.a.
	3	C22H17KN7	418.117702	6.1	5.8	17.5	ok	even	13.0	20.9	n.a.	n.a.	n.a.	n.a.
	4	C16H21KN5O6	418.112342	-6.7	-6.9	8.5	ok	even	21.9	38.4	n.a.	n.a.	n.a.	n.a.

Compound 3k
Chromatogram

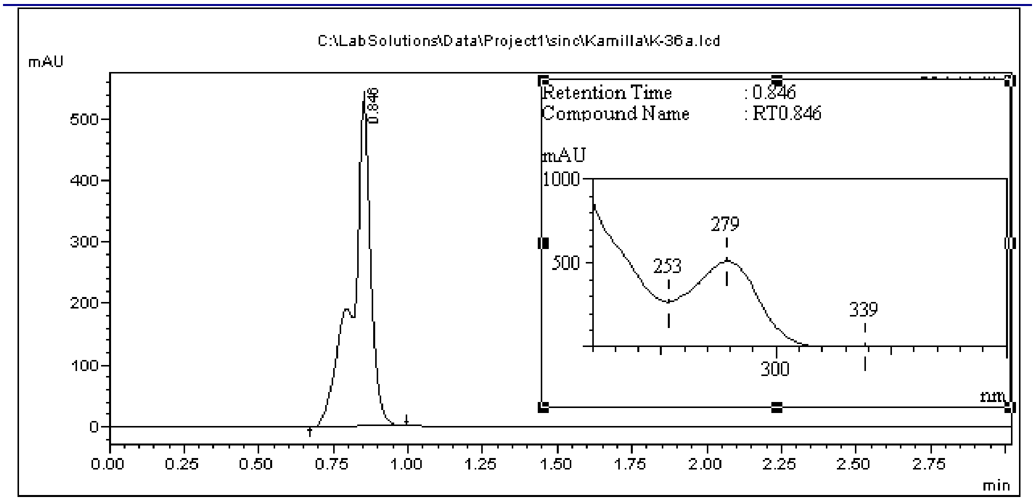

^{1}H NMR

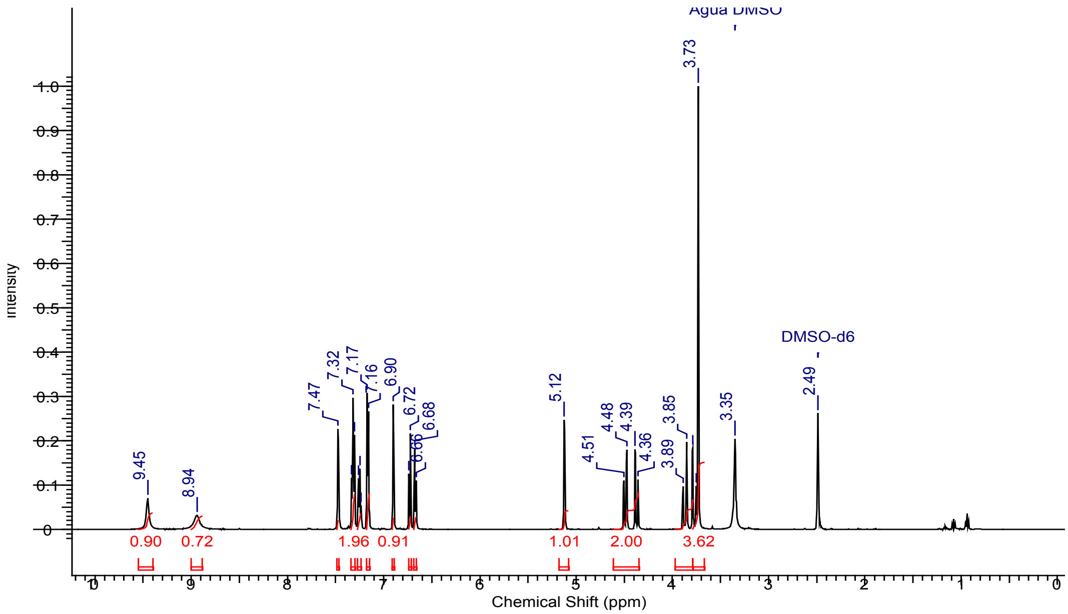
Agua DMSO
3.73
DMSO-d6
2.49
3.35
3.85
3.89
4.36
4.39
4.48
4.51
5.12
6.68
6.66
6.72
6.90
7.16
7.17
7.32
7.47
8.94
9.45
0.90
0.72
1.96
0.91
1.01
2.00
3.62
Intensity
Chemical Shift (ppm)

^{13}C NMR

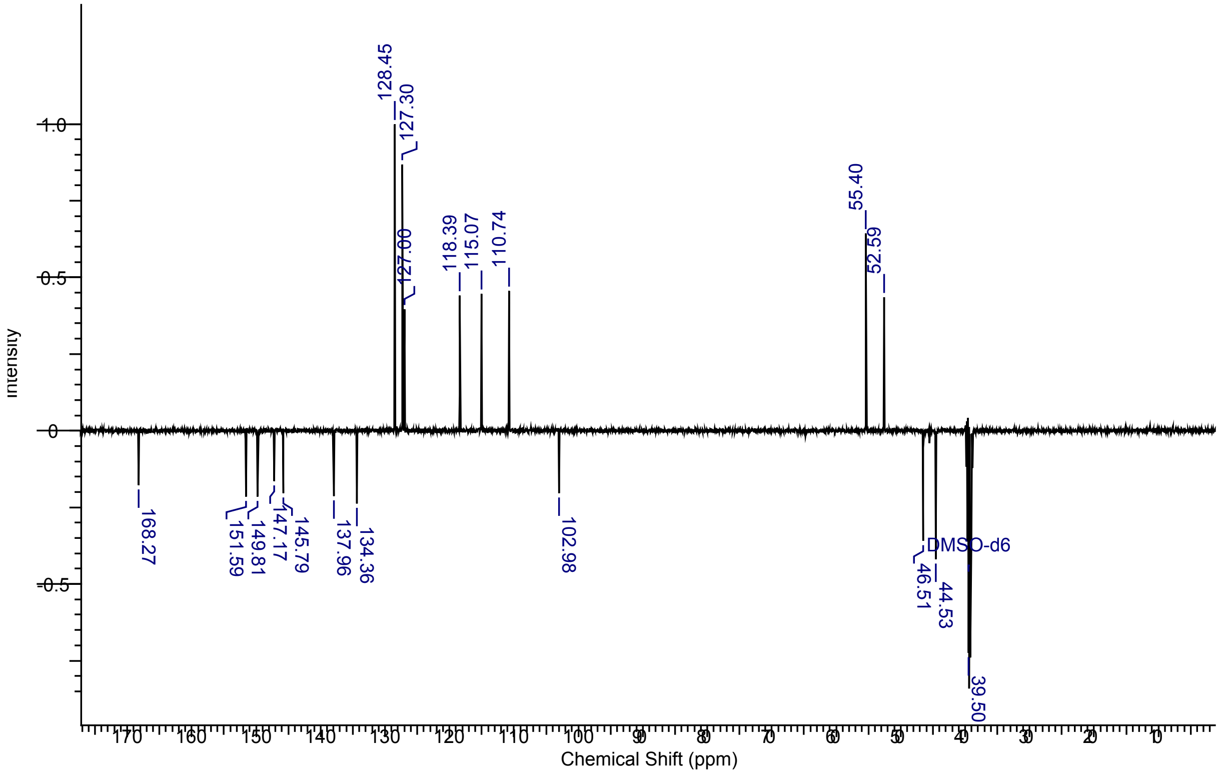
128.45
127.30
127.00
118.39
115.07
110.74
55.40
52.59
168.27
151.59
149.81
147.17
145.79
137.96
134.36
102.98
DMSO-d6
46.51
44.53
39.50
Intensity
Chemical Shift (ppm)

FT-IR

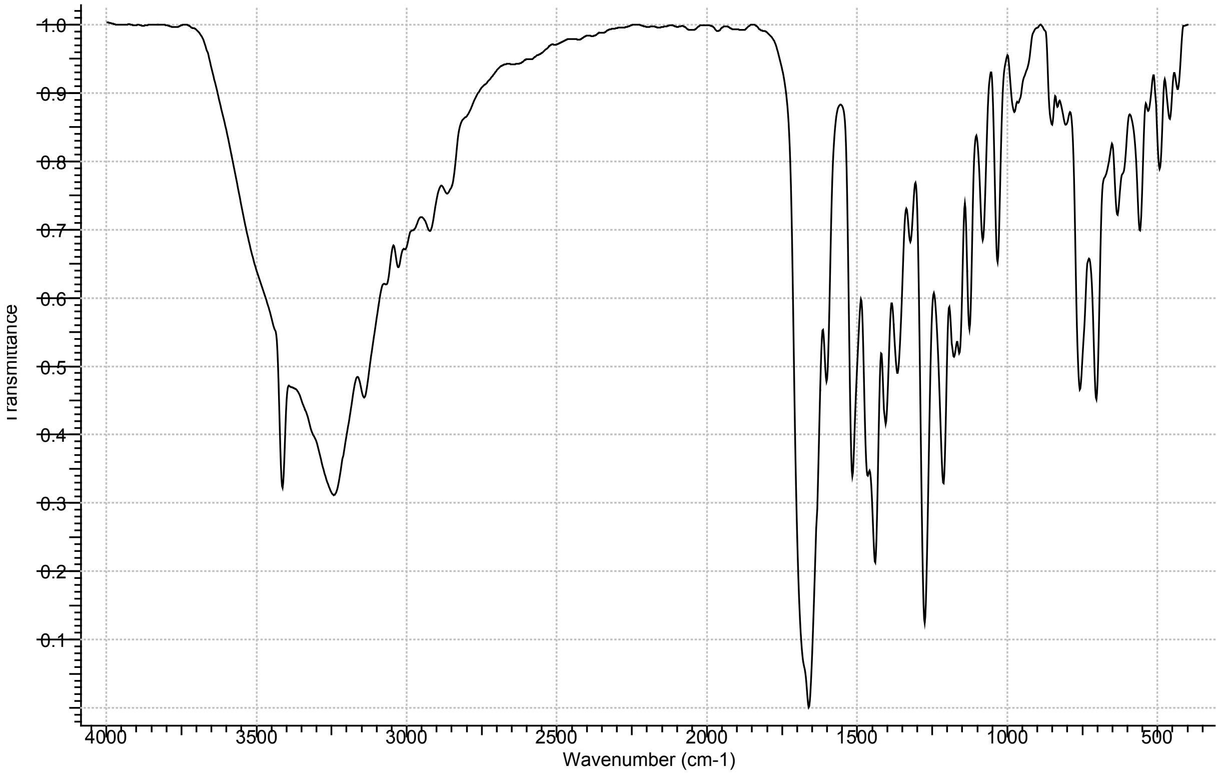

Mass Spectrometry

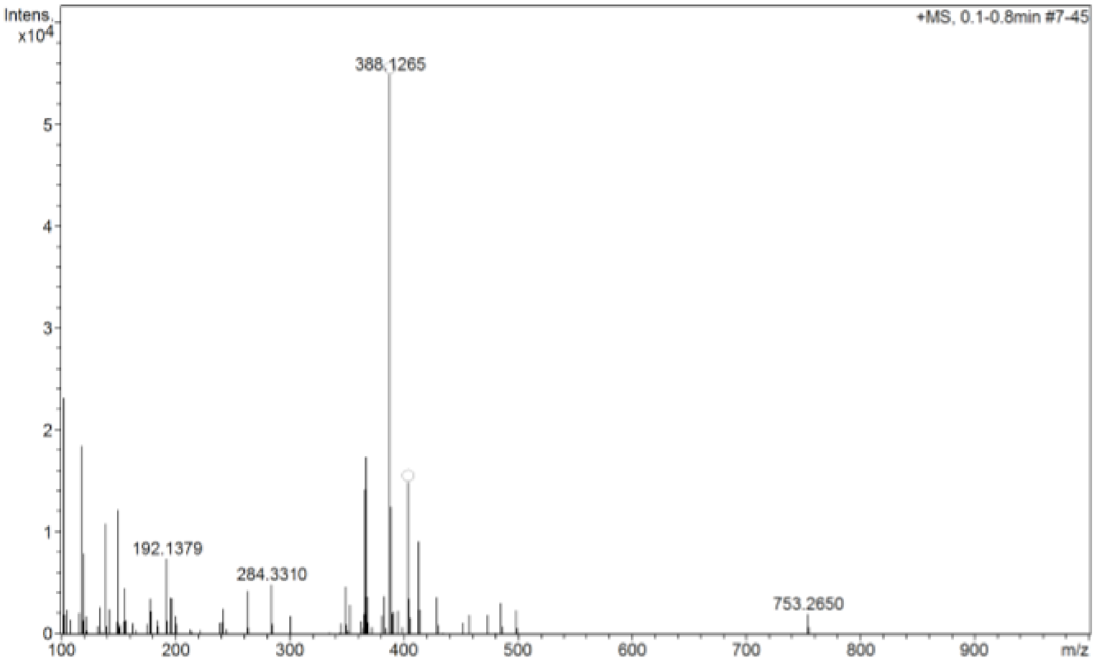

Meas. m/z	#	Ion Formula	m/z	err [ppm]	Mean err [ppm]	rdb	N-Rule	e⁻ Conf	mSigma	Std I	Std Mean m/z	Std I VarNorm	Std m/z Diff	Std Comb Dev
388.126494	1	C20H19N3NaO4	388.126777	0.7	-0.1	12.5	ok	even	2.5	3.4	n.a.	n.a.	n.a.	n.a.
	2	C16H15N9NaO2	388.124091	-6.2	-8.0	13.5	ok	even	13.0	24.3	n.a.	n.a.	n.a.	n.a.
	3	C21H15N7Na	388.128114	4.2	3.0	17.5	ok	even	15.8	22.6	n.a.	n.a.	n.a.	n.a.
	4	C15H19N5NaO6	388.122754	-9.6	-11.1	8.5	ok	even	25.1	43.4	n.a.	n.a.	n.a.	n.a.
404.100211	1	C20H19KN3O4	404.100714	1.2	1238.1	12.5	ok	even	10.3	18.1	n.a.	n.a.	n.a.	n.a.
	2	C21H15KN7	404.102052	4.6	1235.6	17.5	ok	even	15.3	28.5	n.a.	n.a.	n.a.	n.a.
	3	C16H15KN9O2	404.098029	-5.4	1236.4	13.5	ok	even	15.7	23.9	n.a.	n.a.	n.a.	n.a.
	4	C15H19KN5O6	404.096691	-8.7	1238.2	8.5	ok	even	25.4	40.4	n.a.	n.a.	n.a.	n.a.
