# Supplementary material for: Synthesis and molecular modelling studies of pyrimidinones and pyrrolo[3,4-d]-pyrimidinodiones as new antiplasmodial compounds
Source: Mem Inst Oswaldo Cruz. 2018 Jun 18;113(8):e170452. doi: 10.1590/0074-02760170452 (PMC6001580; doi:10.1590/0074-02760170452)
Supplement: Supplementary file 2 [file 0074-0276-mioc-113-8-e170452-suppl02.pdf]

PharmMapper predictions and their respective homologues in *Plasmodium falciparum*

PDB ID	Targert Name	Fit Score	Normalized Fit Score	Organism	Homologue in *P. falciparum*	Coverage	E value	Identity	Accession
1U4O	L-lactate dehydrogenase	3,541	0,3219	*Plasmodium falciparum*	L-lactate dehydrogenase	—	—	—	XP_001349989.1
1V0O	Protein kinase 5	2,52	0,252	*Plasmodium falciparum*	protein kinase 5	—	—	—	XP_001350280.1
1RDW	Actin, alpha skeletal muscle	2,514	0,2514	*Oryctolagus cuniculus*	actin I	99%	0	82%	XP_001350847.1
1OM1	Casein kinase II subunit alpha	5,057	0,3371	*Zea mays*	casein kinase II, alpha subunit	96%	1,00E-154	69%	XP_001347771.1
3BM9	Heat shock protein HSP 90-alpha	3,313	0,4141	*Homo sapiens*	heat shock protein 86	97%	0	64%	XP_001348998.1
1BOA	Methionine aminopeptidase 2	3,517	0,4397	*Homo sapiens*	methionine aminopeptidase	76%	7,00E-153	58%	XP_001348501.1
3F7Z	Glycogen synthase kinase-3 beta	4,223	0,4223	*Homo sapiens*	Glycogen synthase kinase-3 bet	80%	4,00E-136	55%	XP_001351197.1
2CSN	Casein kinase I homolog 1	2,554	0,4256	*Schizosaccharomyces pombe*	casein kinase 1	63%	6,00E-111	54%	XP_020438962.1
1VC2	Glyceraldehyde 3-phosphate dehydrogenase	3,639	0,2599	*Thermus thermophilus*	aldehyde-3-phosphate dehydro	96%	4,00E-107	52%	XP_001348772.1
2UZT	cAMP-dependent protein kinase catalytic [illegible]	3,716	0,3716	*Bos taurus*	ependent protein kinase catalyti	90%	2,00E-114	50%	XP_001352213.1

Homology modeling details

P. falciparum target	PDB (ID)/Homology model	Resoluion	Template ID	Template name	Coverage	Identity
L-lactate dehydrogenase	PDB (IXIV)	1,7	—	—	—	—
protein kinase 5	PDB (1V0O)	1,9	—	—	—	—
actin I	PDB (4CBU)	1,3	—	—	—	—
casein kinase II, alpha subunit	PDB (5XVU)	3,0	—	—	—	—
heat shock protein 86	Homology model	—	5fwl	Heat shock protein 90 beta	92%	69%
methionine aminopeptidase	Homology model	—	5cls	Methionine aminopeptidase 2	58%	59%
Glycogen synthase kinase-3 beta	Homology model	—	4e7w	Glycogen Synthase Kinase 3	81%	49%
casein kinase 1	Homology model	—	5fqd	Casein kinase I alpha	100%	63%
glyceraldehyde-3-phosphate dehydrogenase	PDB (2B4R)	2,25	—	—	—	—
MP-dependent protein kinase catalytic subu	Homology model	—	3l9m	cAMP-dependent protein kinase catalytic subunit alpha	96%	51%

Statistical validation of homology models

P. falciparum target	Poor rotamers	Favored rotamers	Ramachandran outliers	Ramachandran favored	Clashscore	MolProbity score
L-lactate dehydrogenase	—	—	—	—	—	—
protein kinase 5	—	—	—	—	—	—
actin I	—	—	—	—	—	—
casein kinase II, alpha subunit	—	—	—	—	—	—
heat shock protein 86	0,6%	98,3%	0,1%	97,6%	7,32	1,48
methionine aminopeptidase	0,4%	98,5%	0,3%	96,7%	5,98	1,53
Glycogen synthase kinase-3 beta	1,3%	97,7%	0,0%	98,5%	6,18	1,43
casein kinase 1	1,2%	97,3%	0,0%	97,6%	7,46	1,54
glyceraldehyde-3-phosphate dehydrogenase	—	—	—	—	—	—
MP-dependent protein kinase catalytic subu	0,4%	98,6%	0,0%	97,1%	7,28	1,26

Docking scores

P. falciparum target	Grid Dimensions (x, y, z)	28a	28b	28c	28d	28f	28h	28i	28k	28l
L-lactate dehydrogenase	35.51 x 11.74 x 17.30	-5,07	-3,79	-5,10	-4,36	-3,67	-4,55	-3,91	-3,49	-5,26
protein kinase 5	21.00 x 4.24 x 35.95	-8,76	-8,00	-8,87	-8,36	-8,33	-9,31	-6,25	-8,24	-9,10
actin I	10.25 x 19.62 x 19.69	-3,67	-3,71	-3,66	-3,75	-3,68	-3,89	-4,42	-4,12	-4,30
casein kinase II, alpha subunit	3.81 x -6.78 x -13.62	-6,33	-6,75	-7,06	-6,01	-5,59	-5,82	-5,20	-6,07	-7,94
heat shock protein 86	175.54 x 176.34 x 137.02	-5,38	-5,93	-5,48	-5,96	-5,20	-5,79	-5,48	-4,04	-5,84
methionine aminopeptidase	27.28 x 20.08 x 17.83	-7,89	-8,00	-7,38	-8,77	-7,15	-7,13	-7,41	-8,39	-7,60
Glycogen synthase kinase-3 beta	93.81 x 68.05 x 9.74	-7,95	-7,92	-7,91	-7,89	-8,00	-6,27	-6,56	-8,06	-8,07
casein kinase 1	3.84 x -7.08 x -14.00	-5,50	-7,10	-5,61	-4,78	-5,12	-5,86	-4,23	-4,77	-6,78
glyceraldehyde-3-phosphate dehydrogenase	16.93 x 21.00 x 14.07	-5,54	-6,15	-5,95	-5,92	-5,46	-6,26	-6,14	-5,87	-6,92
MP-dependent protein kinase catalytic subu	26.00 x -8.67 x 21.11	-5,91	-5,71	-6,68	-6,20	-5,95	-6,60	-5,38	-5,55	-7,13

CC BY
