# Supplementary figures and images for: Synthesis and molecular modelling studies of pyrimidinones and pyrrolo[3,4-d]-pyrimidinodiones as new antiplasmodial compounds
Source: Mem Inst Oswaldo Cruz. 2018 Jun 18;113(8):e170452. doi: 10.1590/0074-02760170452 (PMC6001580; doi:10.1590/0074-02760170452)

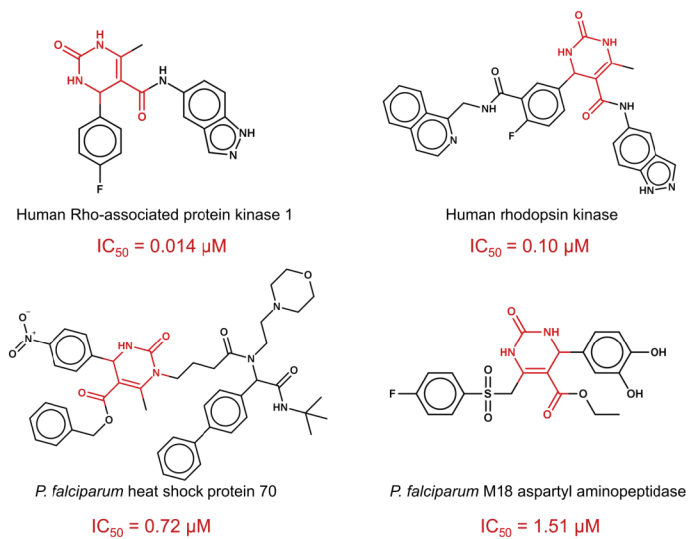

Known dihydropyrimidinones found in the ChEMBL database and their activity

Supplement: Supplementary file 3 [file 0074-0276-mioc-113-8-e170452-suppl03.pdf]
